# Supplementary figures and images for: Ubiquitination-activated TAB–TAK1–IKK–NF-κB axis modulates gene expression for cell survival in the lysosomal damage response (part 1 of 2)
Source: eLife. 2025 Sep 24;14:RP106901. doi: 10.7554/eLife.106901 (PMC12459955; doi:10.7554/eLife.106901)

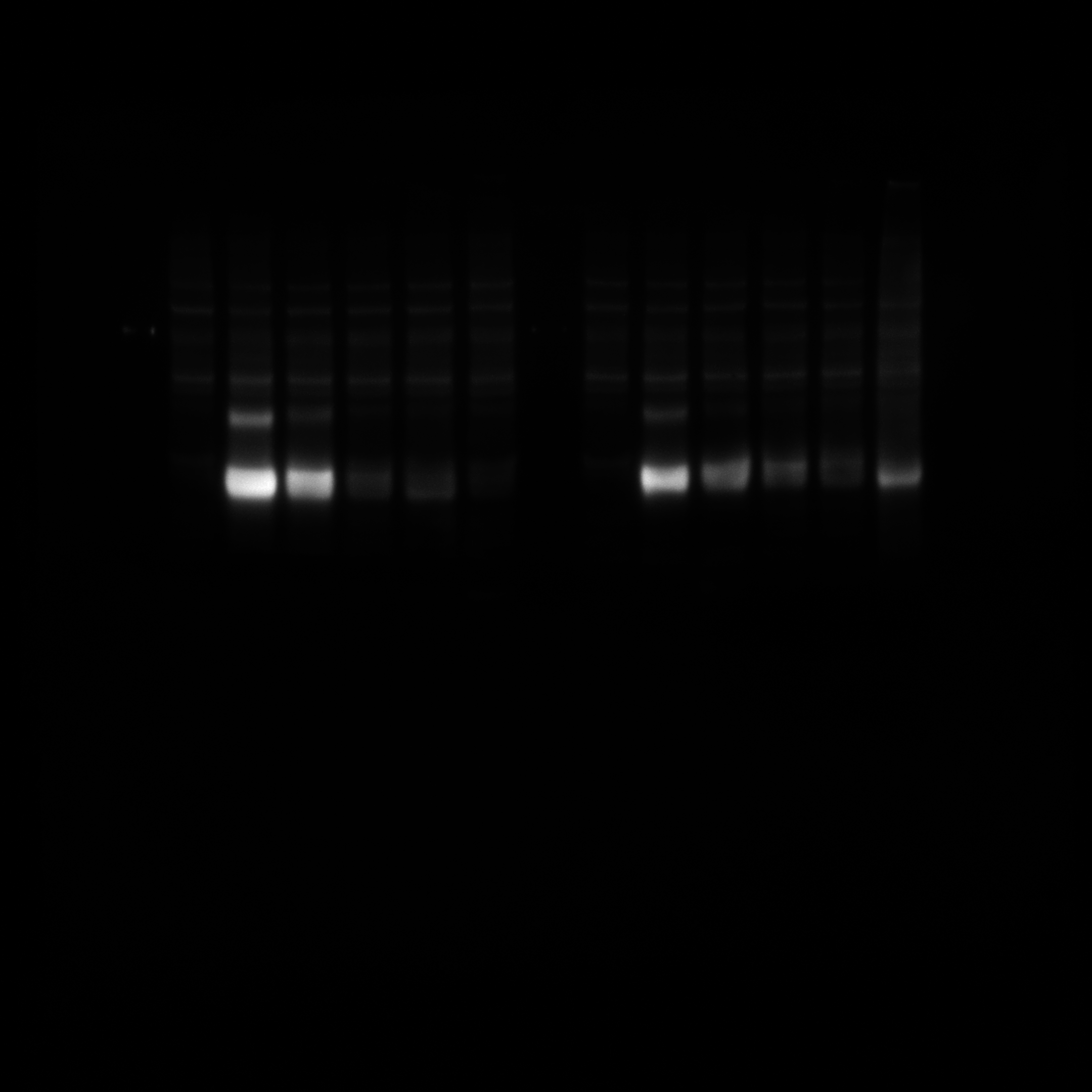

Supplement: Figure 1—source data 1. [file elife-106901-fig1-data1.zip › Figure1 source data 1/Figure1G Fos.Tif]

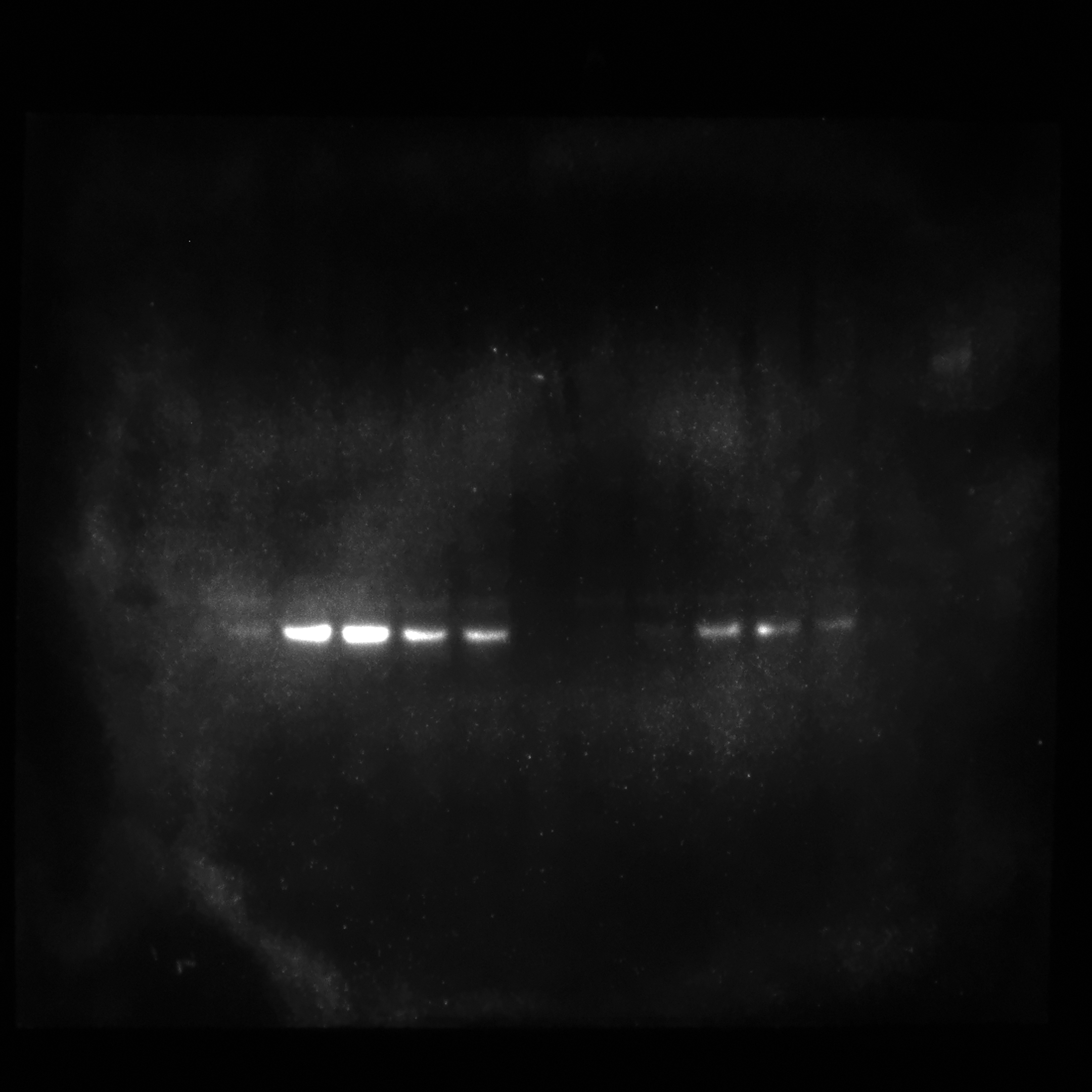

Supplement: Figure 1—source data 1. [file elife-106901-fig1-data1.zip › Figure1 source data 1/Figure1G IL1b.Tif]

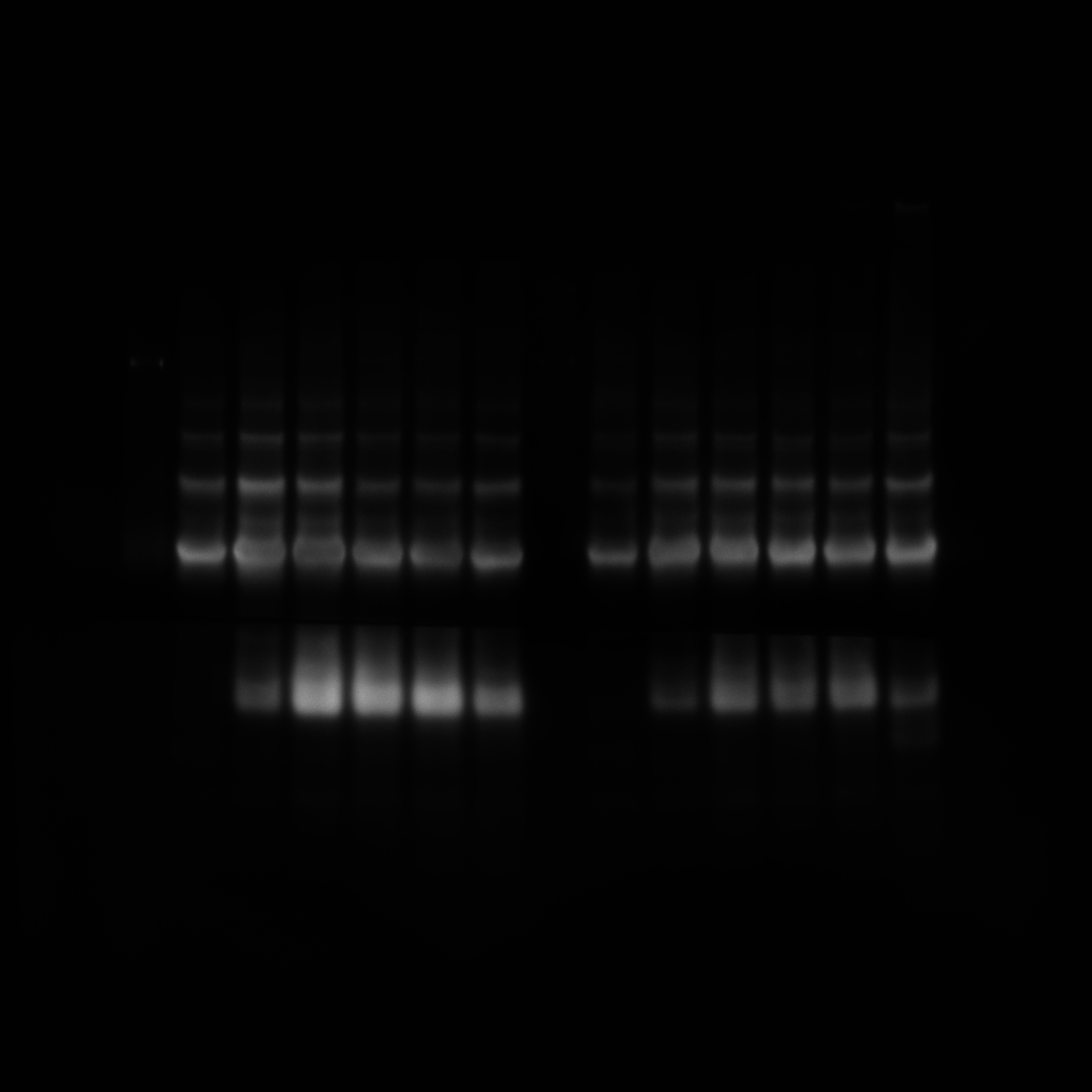

Supplement: Figure 1—source data 1. [file elife-106901-fig1-data1.zip › Figure1 source data 1/Figure1G IL6.Tif]

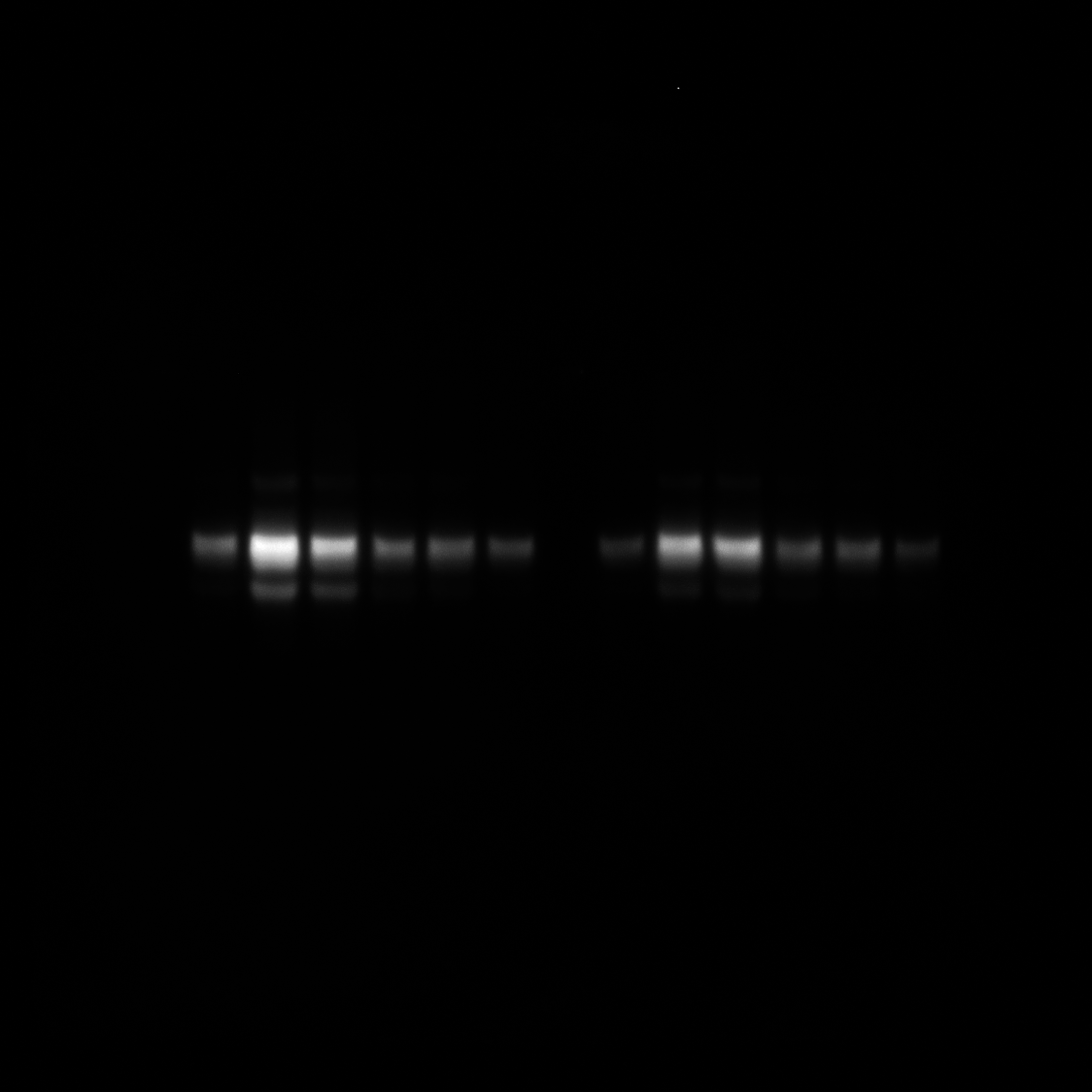

Supplement: Figure 1—source data 1. [file elife-106901-fig1-data1.zip › Figure1 source data 1/Figure1G IRF1.Tif]

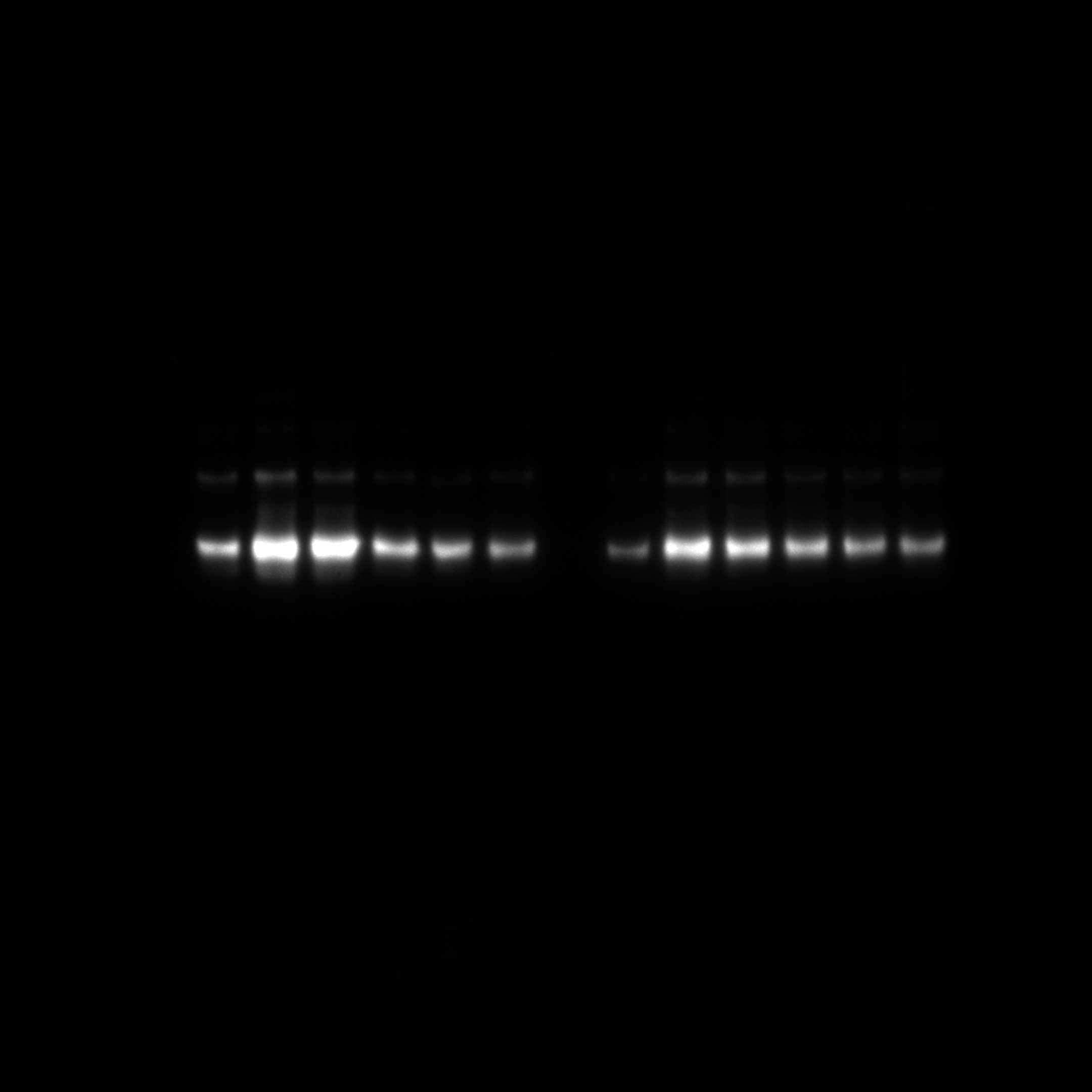

Supplement: Figure 1—source data 1. [file elife-106901-fig1-data1.zip › Figure1 source data 1/Figure1G Jun.Tif]

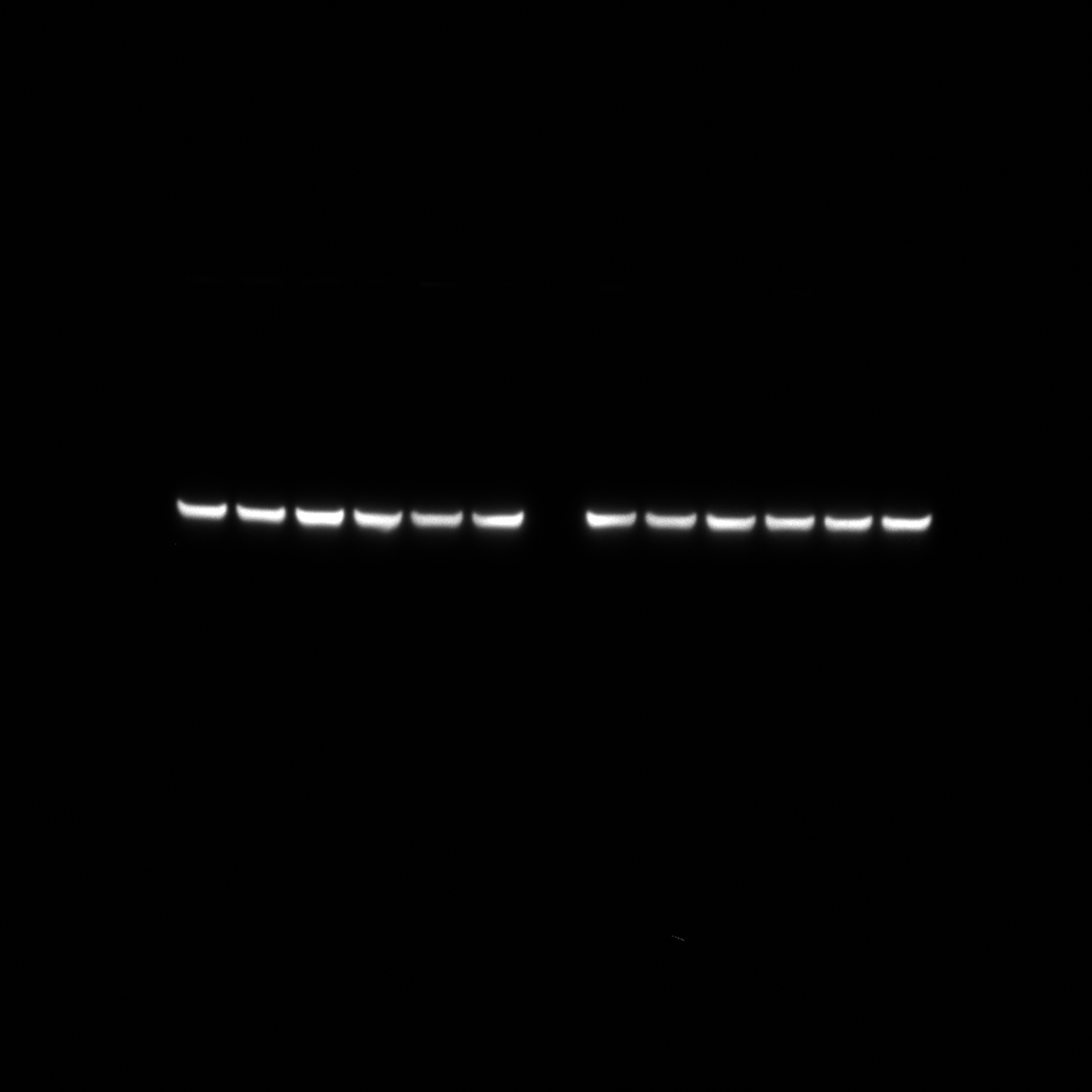

Supplement: Figure 1—source data 1. [file elife-106901-fig1-data1.zip › Figure1 source data 1/Figure1G Tubulin.Tif]

Figure 1G

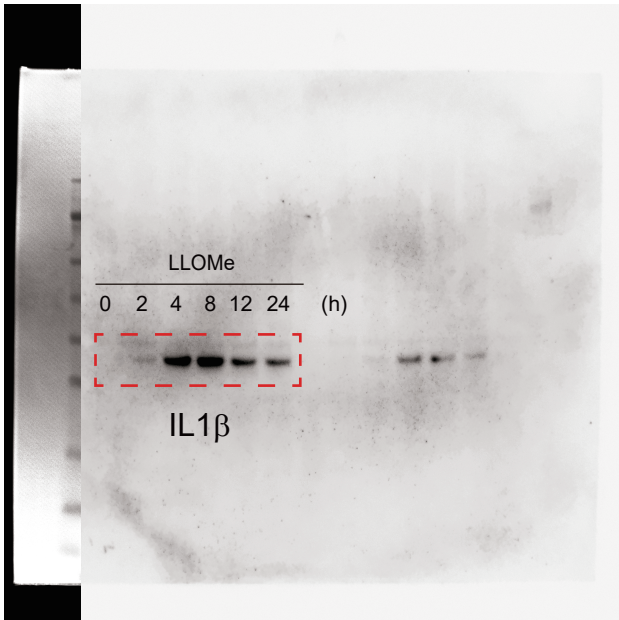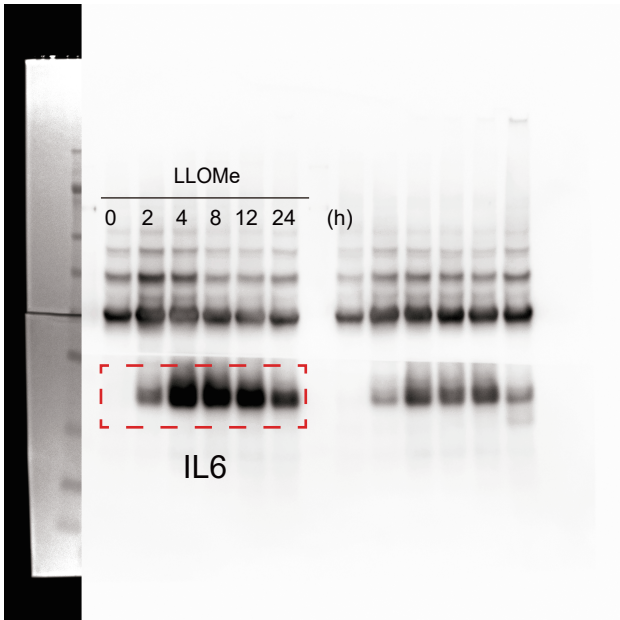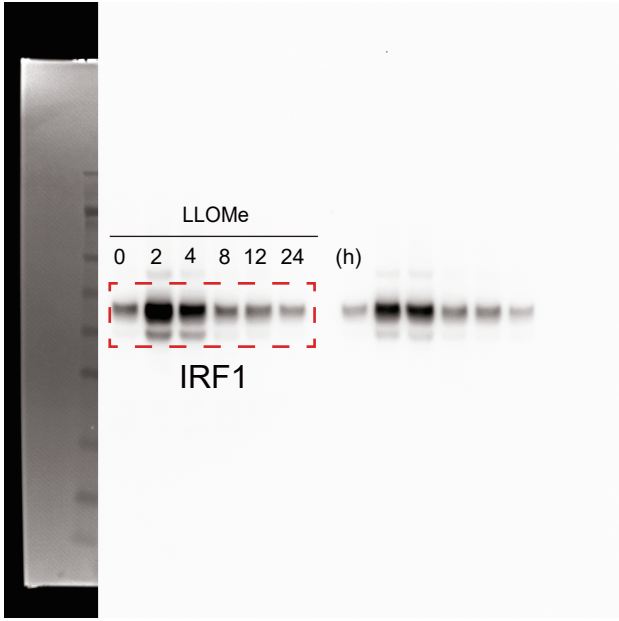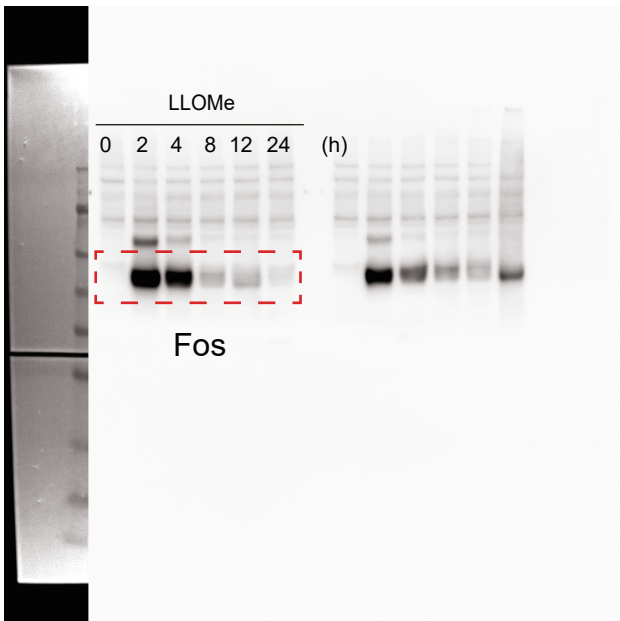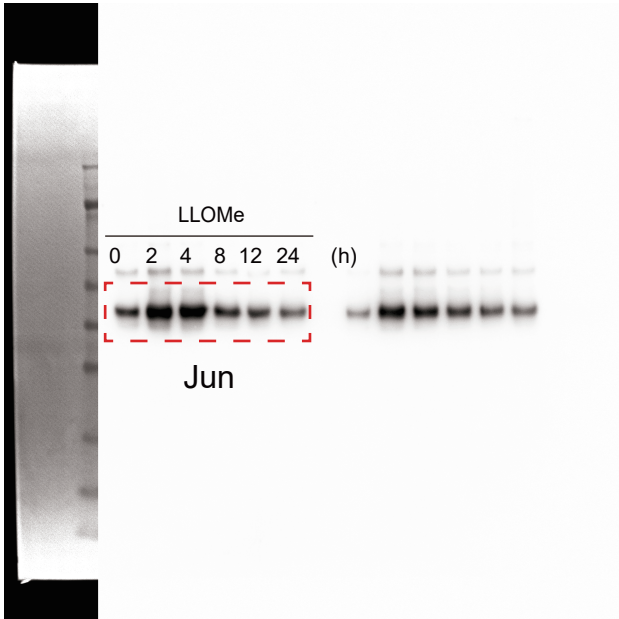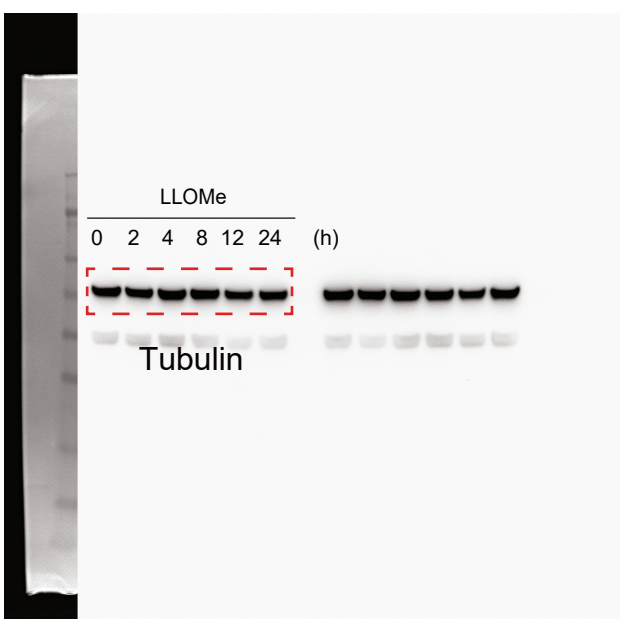

Supplement: Figure 1—source data 2. [file elife-106901-fig1-data2.pdf]

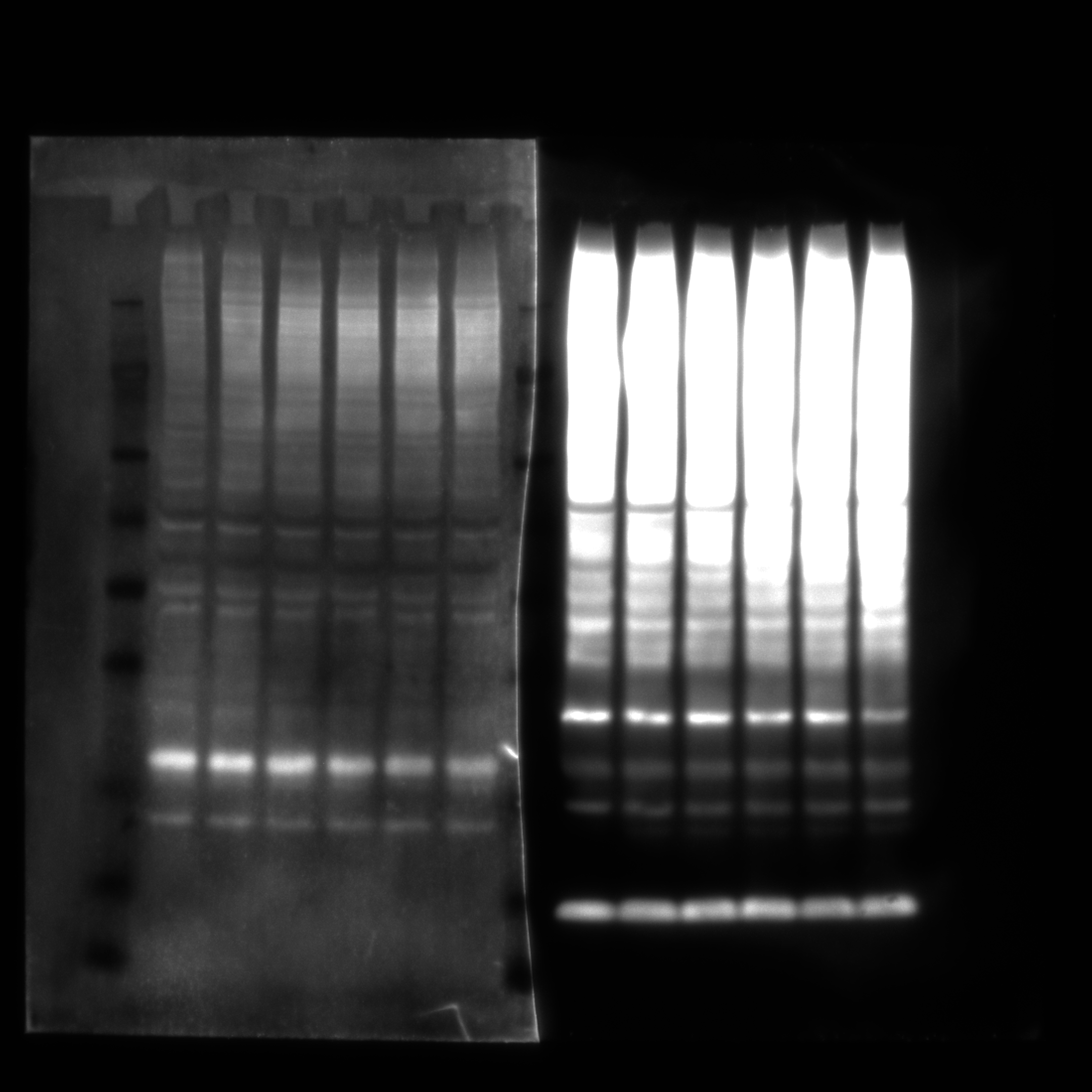

Supplement: Figure 2—source data 1. [file elife-106901-fig2-data1.zip › Figure2 source data 1/Figure 2B K63Ub.Tif]

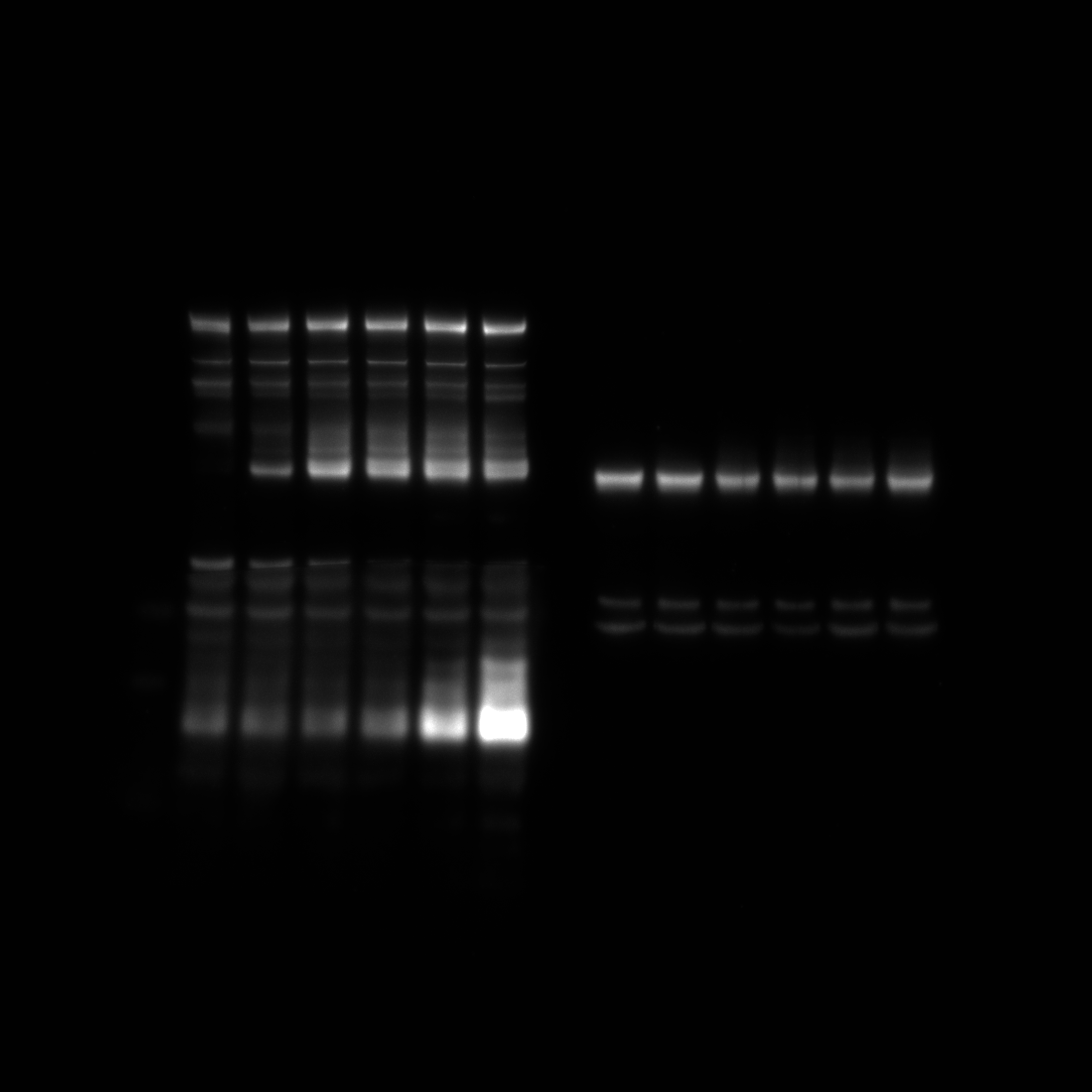

Supplement: Figure 2—source data 1. [file elife-106901-fig2-data1.zip › Figure2 source data 1/Figure 2B pTAK1.Tif]

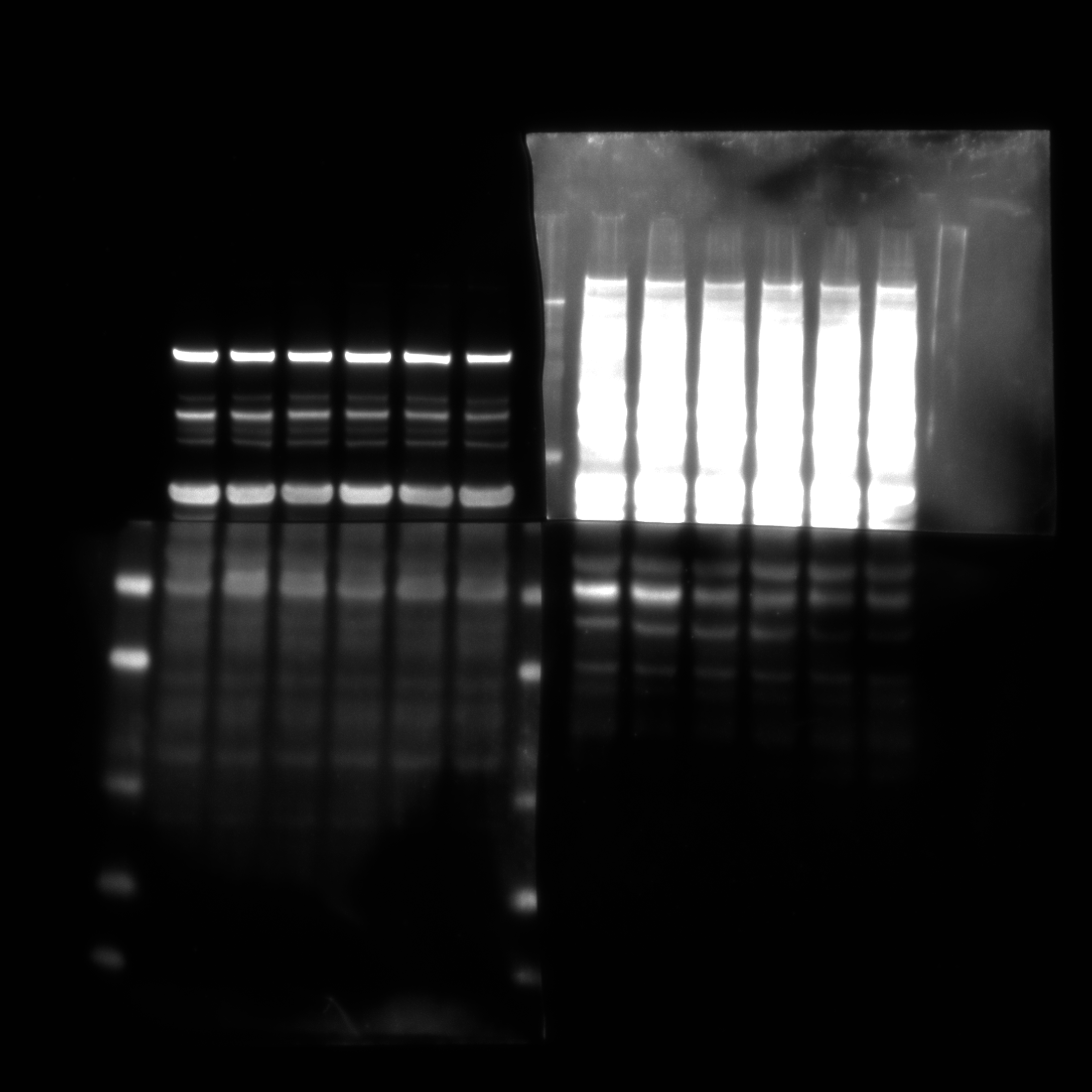

Supplement: Figure 2—source data 1. [file elife-106901-fig2-data1.zip › Figure2 source data 1/Figure 2B TAB2.Tif]

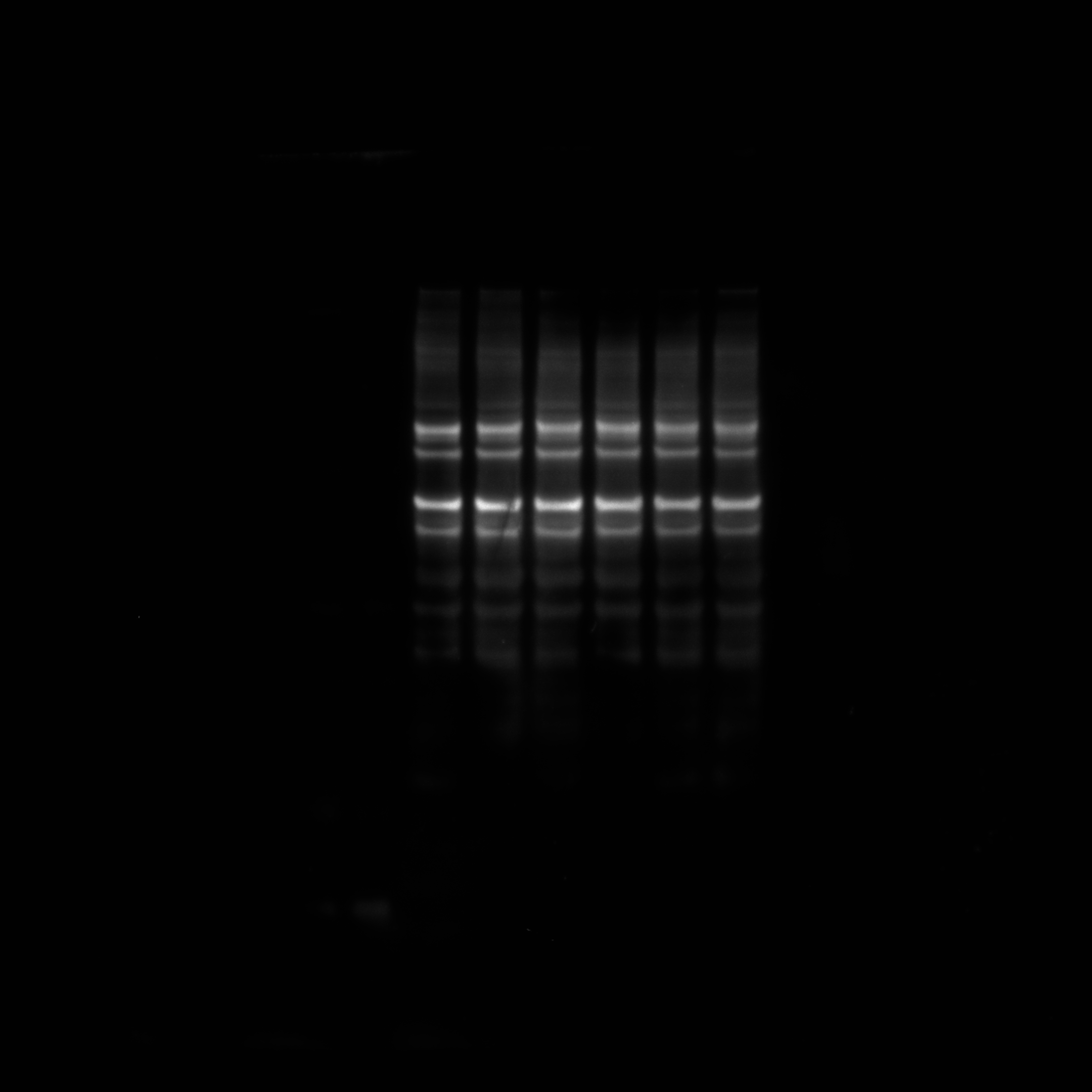

Supplement: Figure 2—source data 1. [file elife-106901-fig2-data1.zip › Figure2 source data 1/Figure 2B TAB3.Tif]

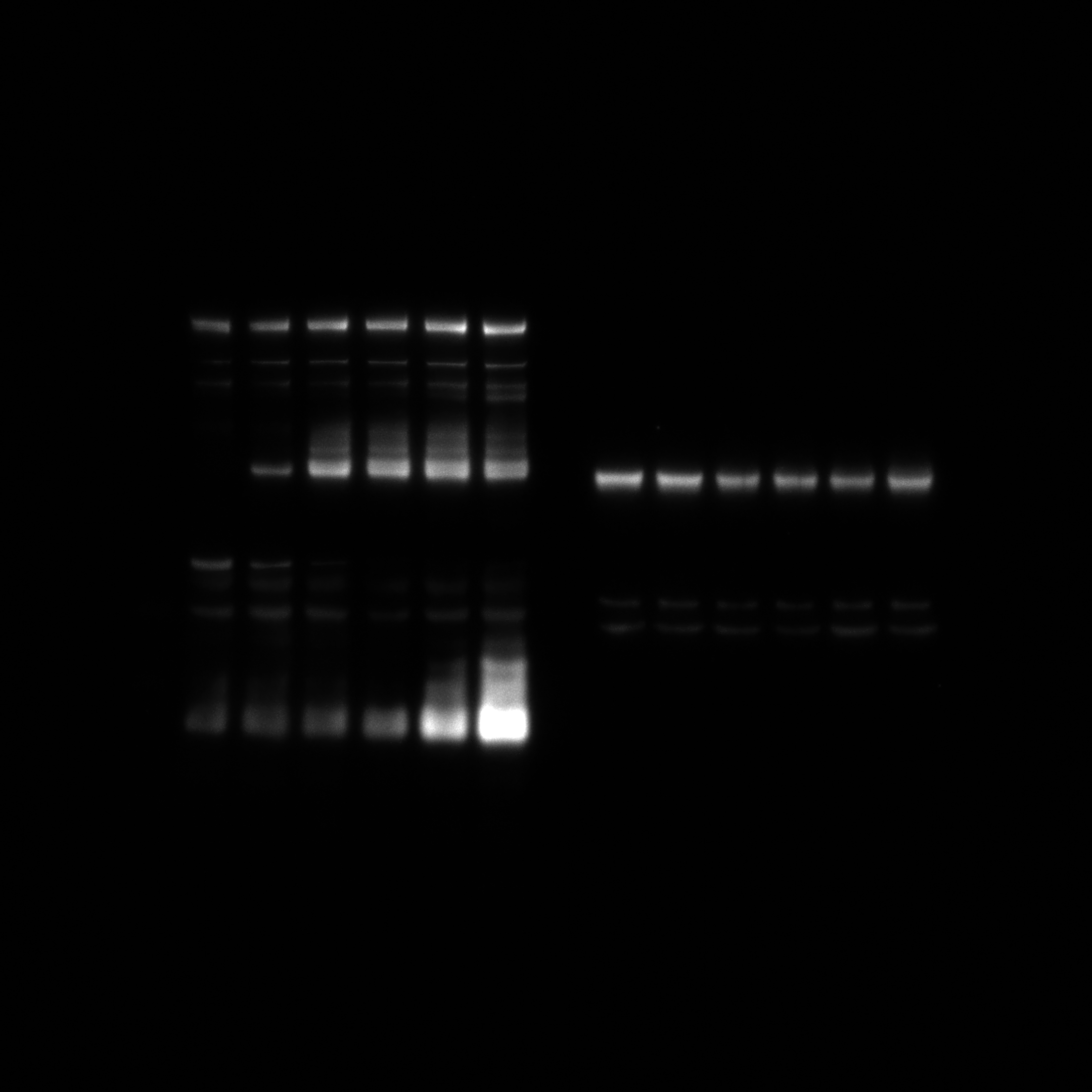

Supplement: Figure 2—source data 1. [file elife-106901-fig2-data1.zip › Figure2 source data 1/Figure 2B TAK1.Tif]

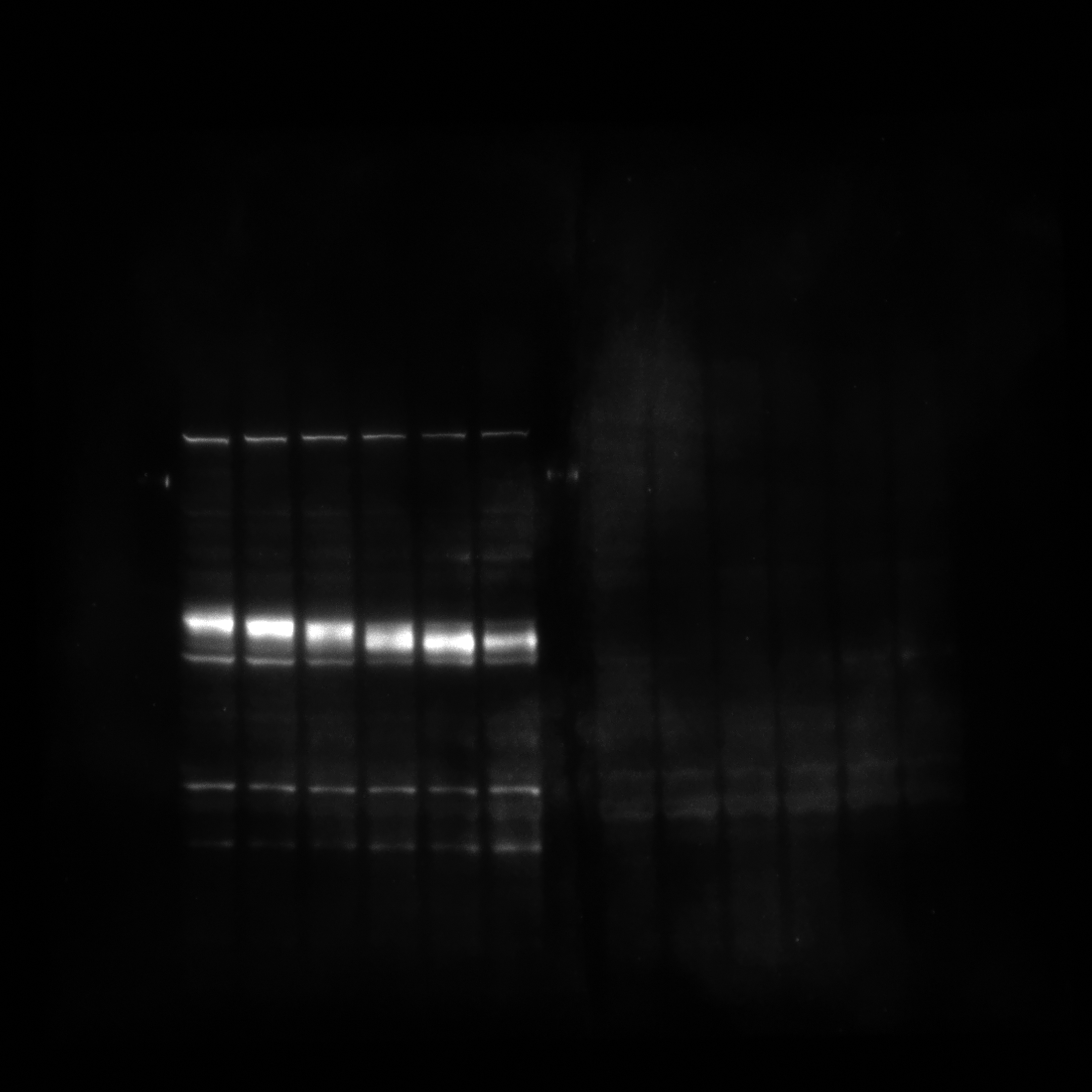

Supplement: Figure 2—source data 1. [file elife-106901-fig2-data1.zip › Figure2 source data 1/Figure 2B TFEB.Tif]

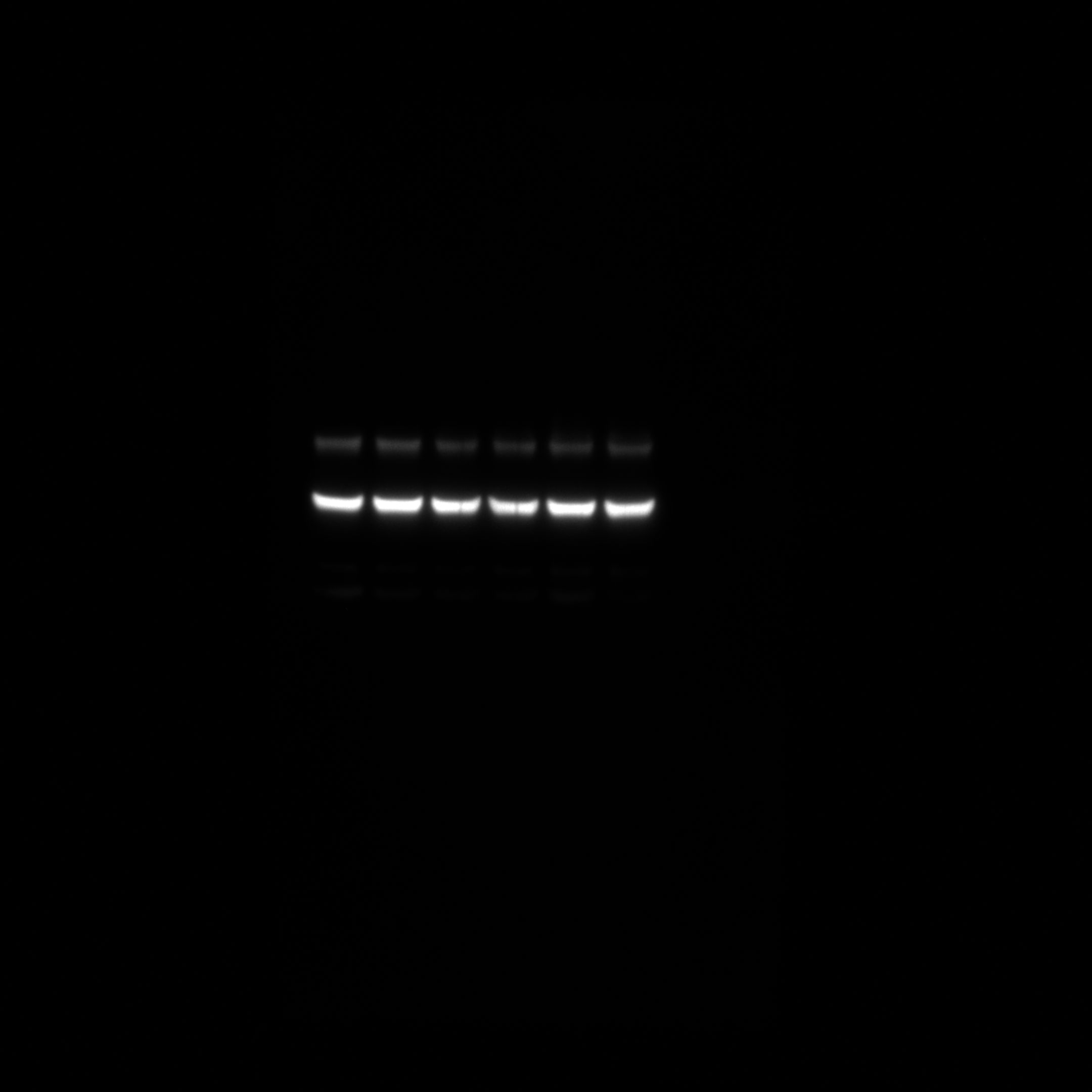

Supplement: Figure 2—source data 1. [file elife-106901-fig2-data1.zip › Figure2 source data 1/Figure 2B Tubulin.Tif]

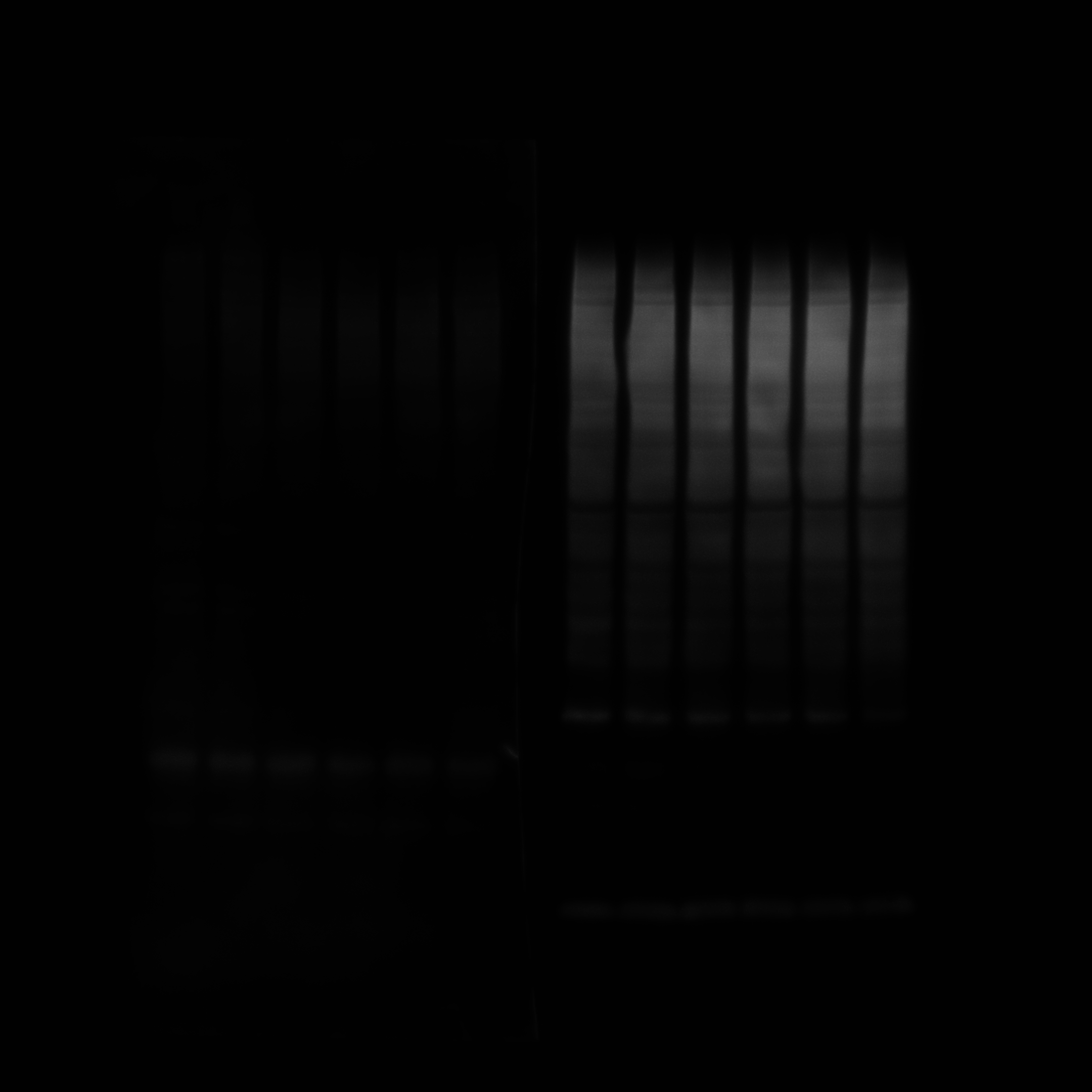

Supplement: Figure 2—source data 1. [file elife-106901-fig2-data1.zip › Figure2 source data 1/Figure 2B Ub.Tif]

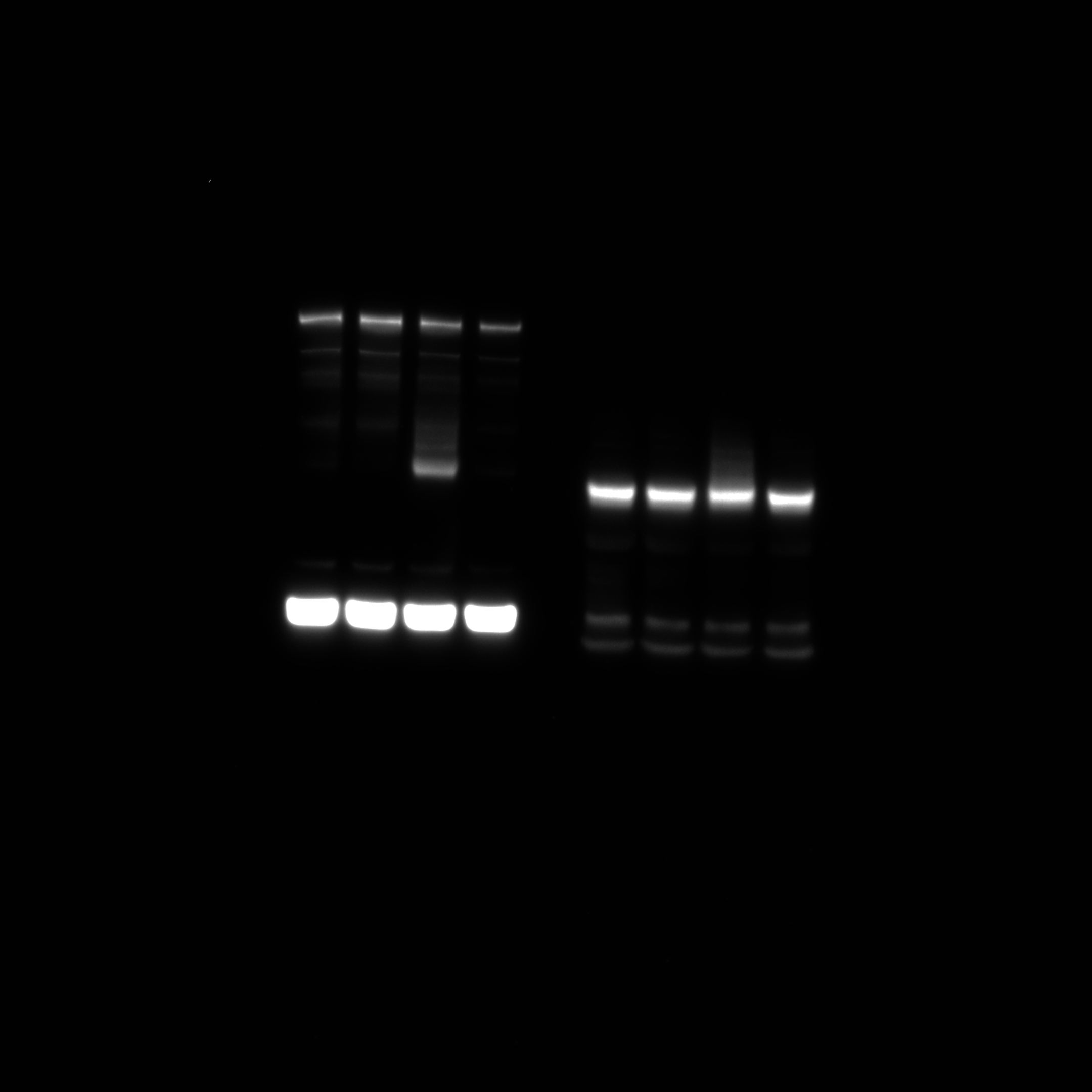

Supplement: Figure 2—source data 1. [file elife-106901-fig2-data1.zip › Figure2 source data 1/Figure 2C pTAK1.Tif]

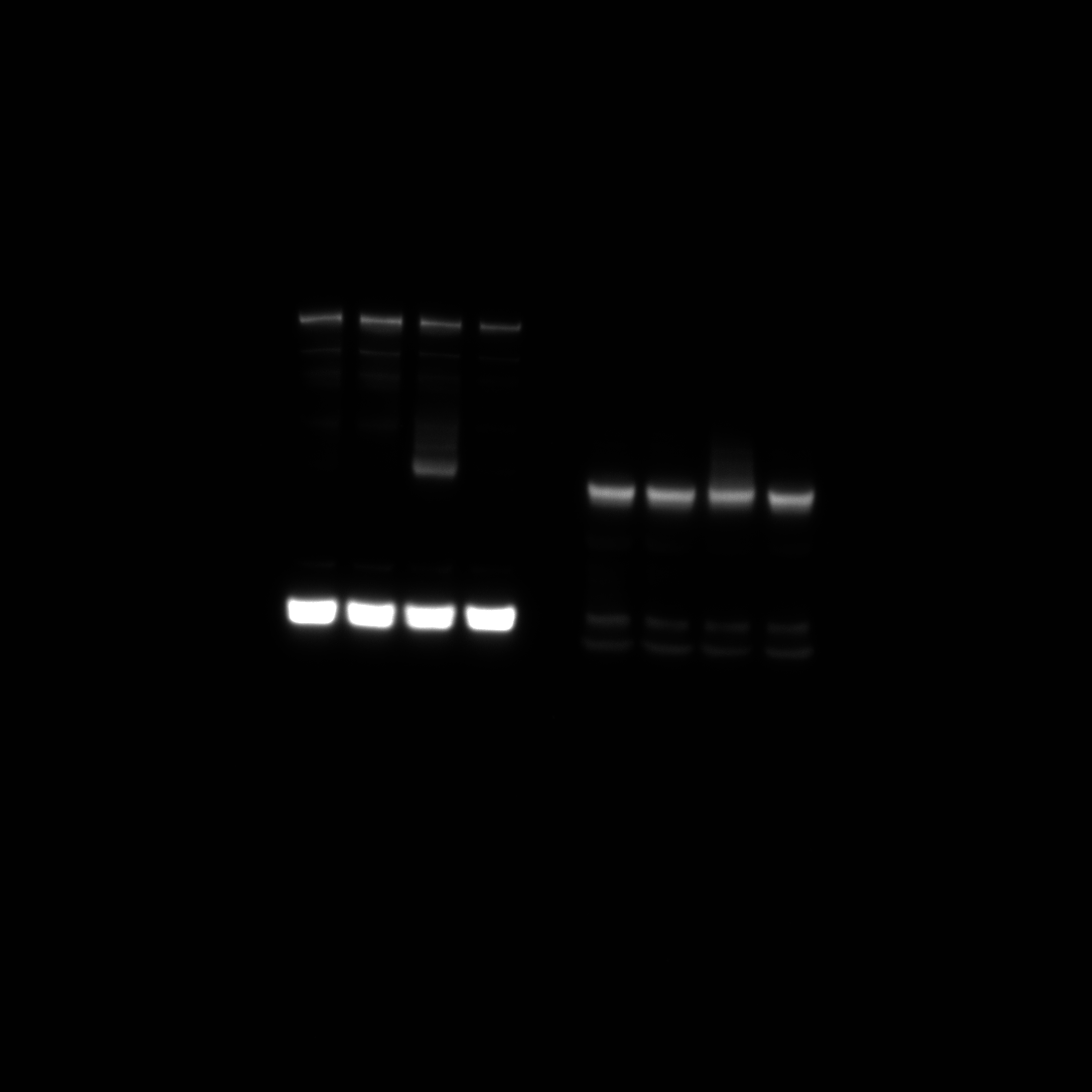

Supplement: Figure 2—source data 1. [file elife-106901-fig2-data1.zip › Figure2 source data 1/Figure 2C TAK1.Tif]

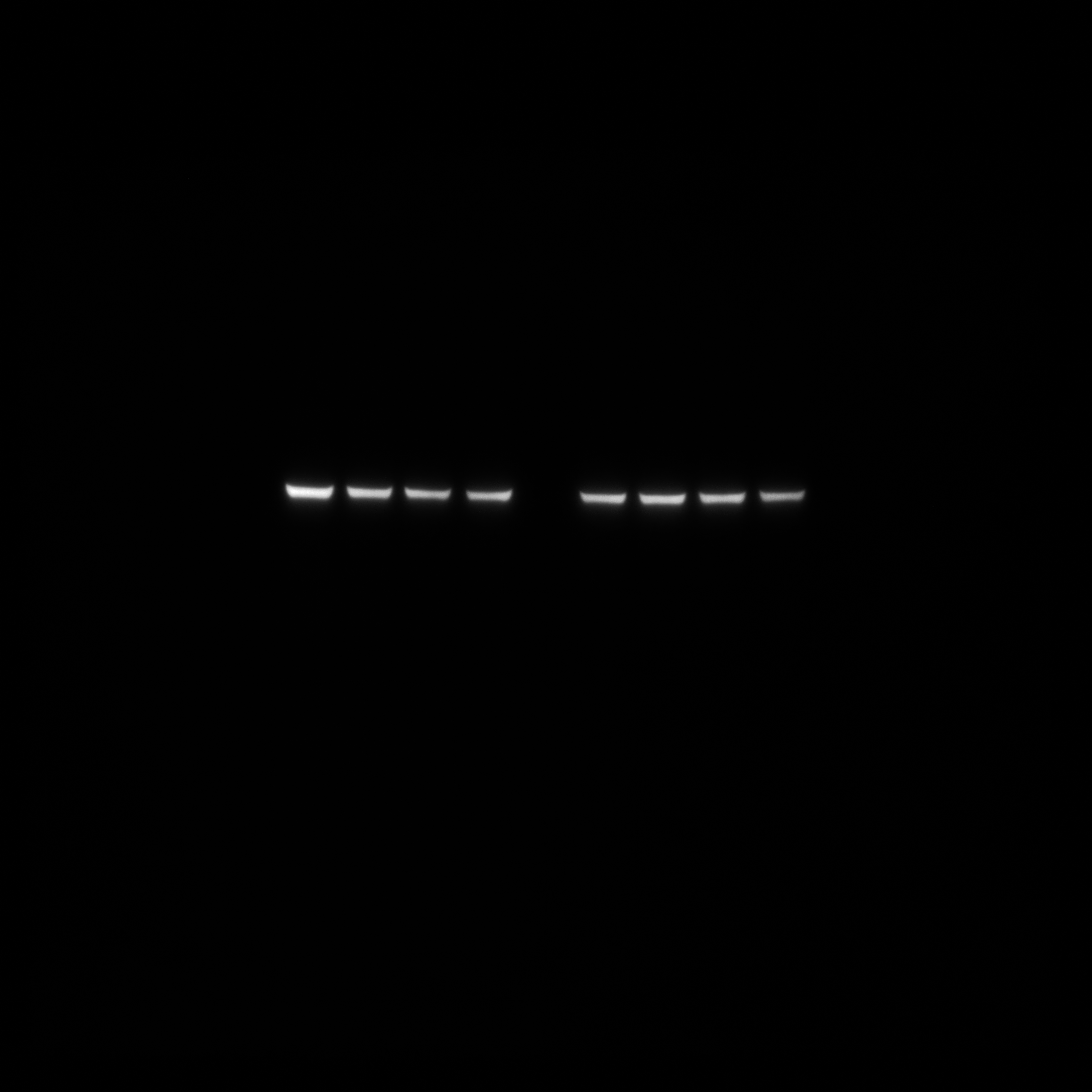

Supplement: Figure 2—source data 1. [file elife-106901-fig2-data1.zip › Figure2 source data 1/Figure 2C Tubulin.Tif]

**Figure 2B**

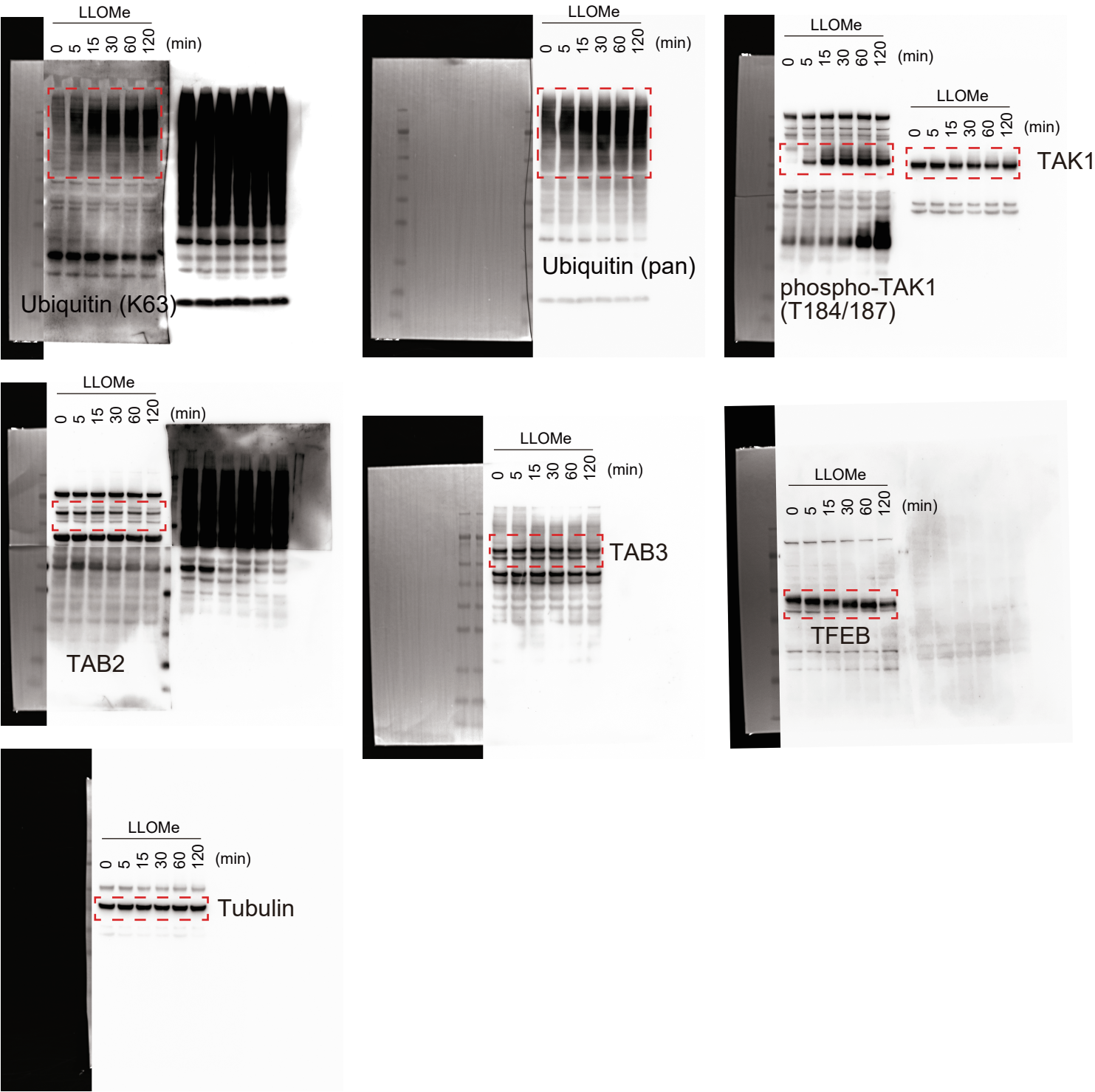

**Figure 2C**

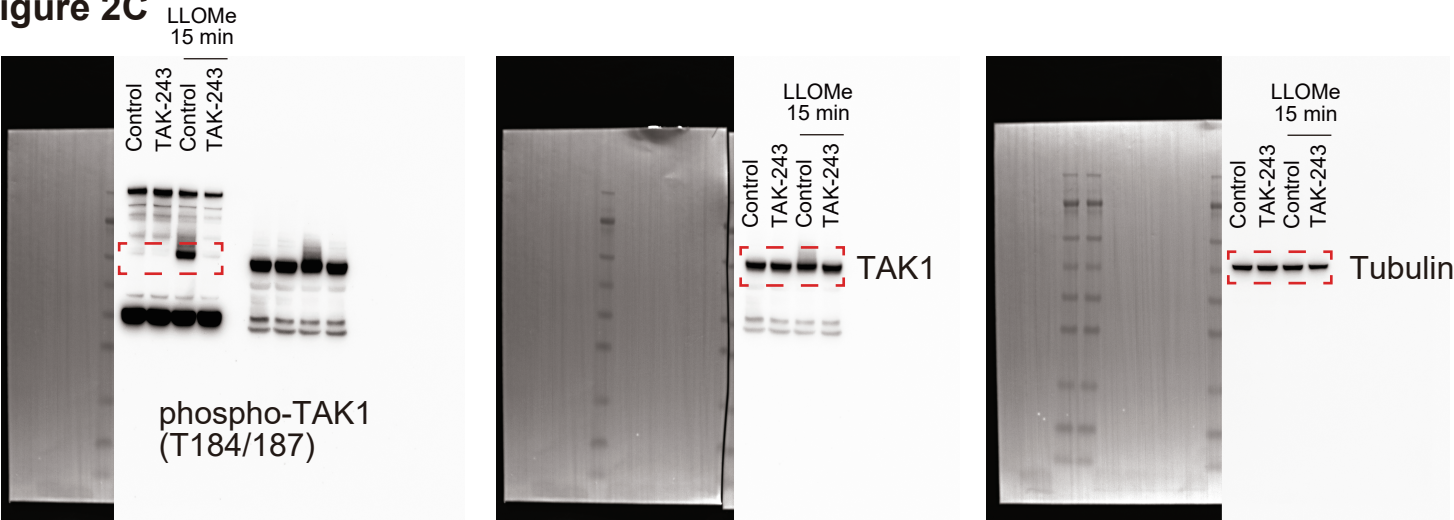

Supplement: Figure 2—source data 2. [file elife-106901-fig2-data2.pdf]

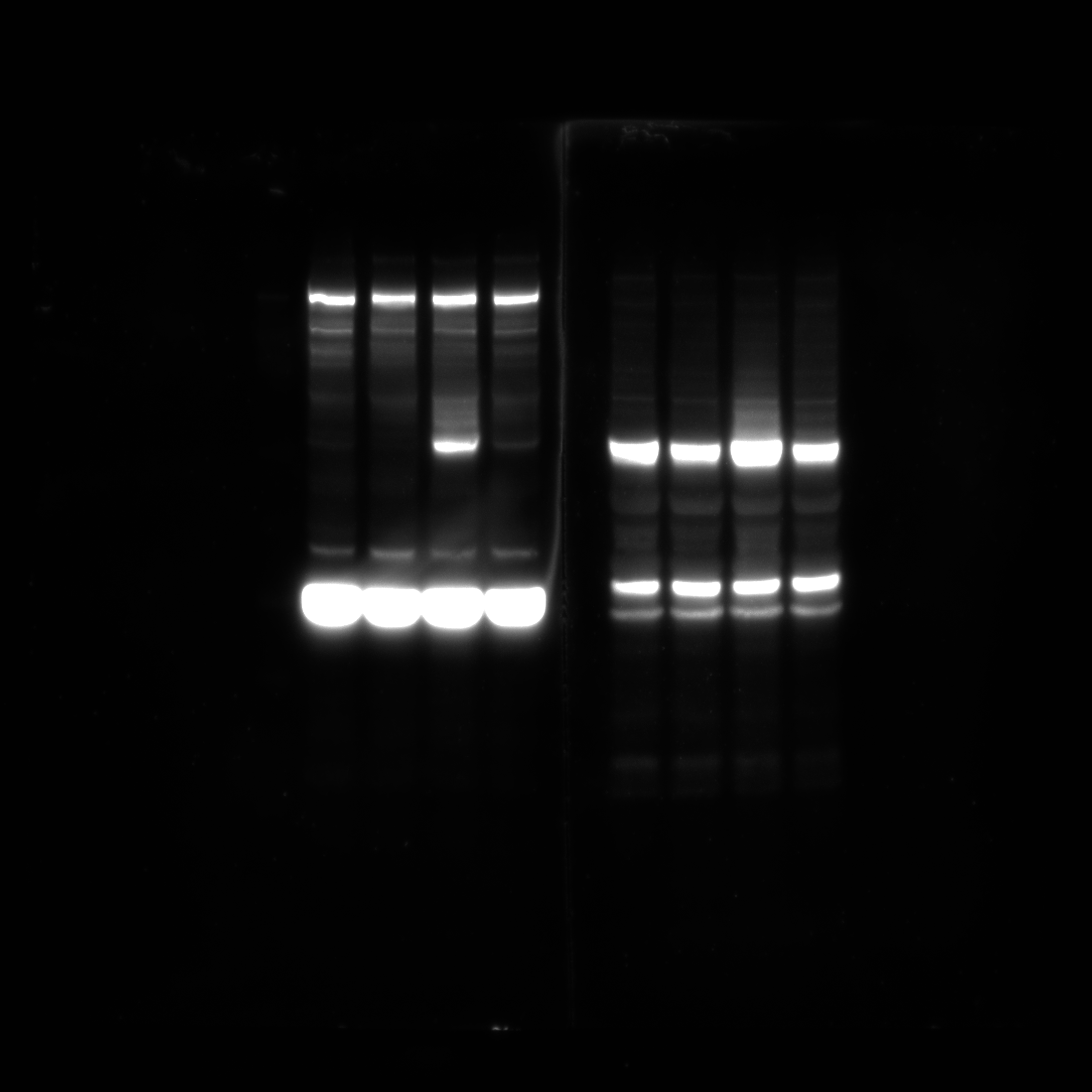

Supplement: Figure 2—source data 3. [file elife-106901-fig2-data3.zip › Figure2 source data 3/Figure 2D pTAK1.Tif]

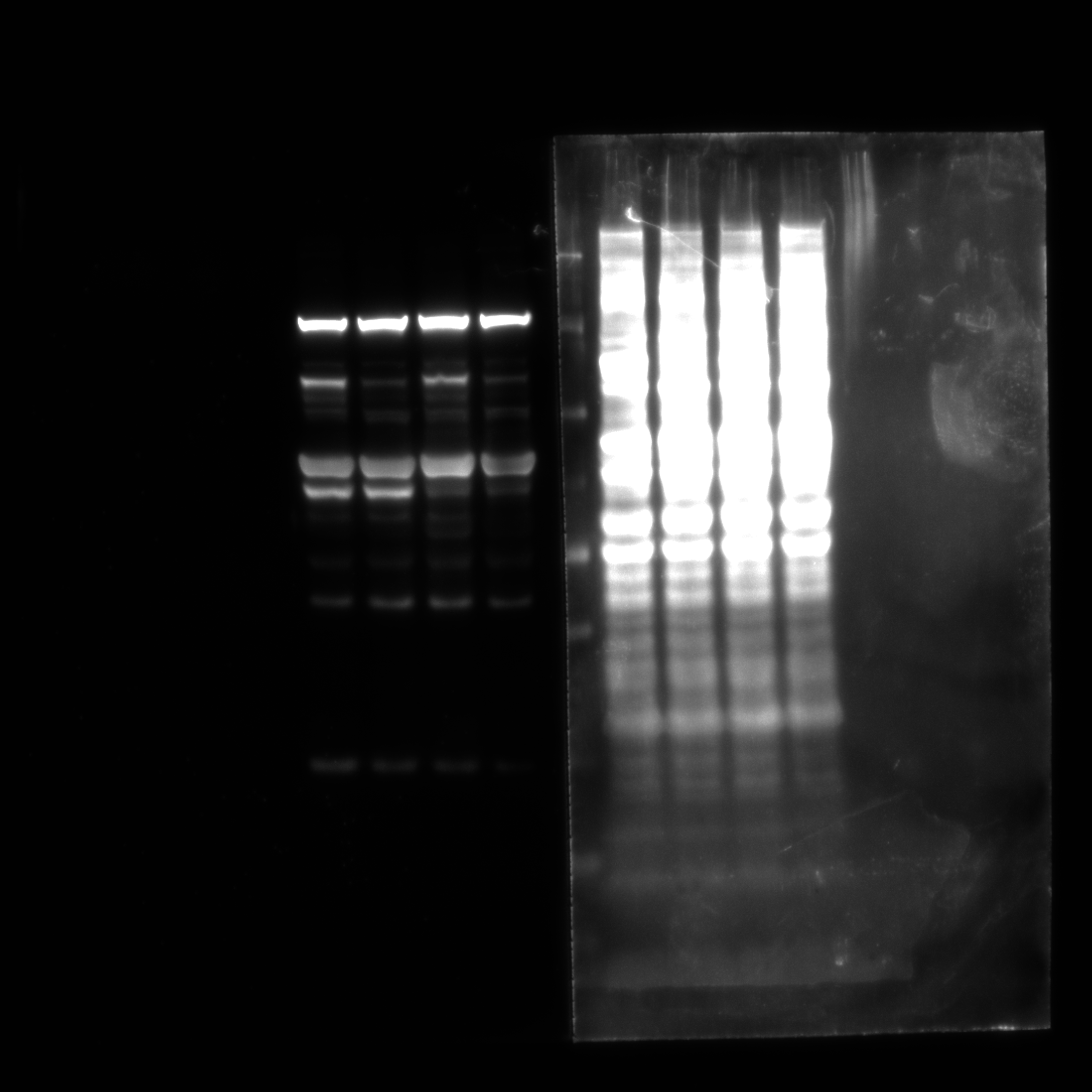

Supplement: Figure 2—source data 3. [file elife-106901-fig2-data3.zip › Figure2 source data 3/Figure 2D TAB2.Tif]

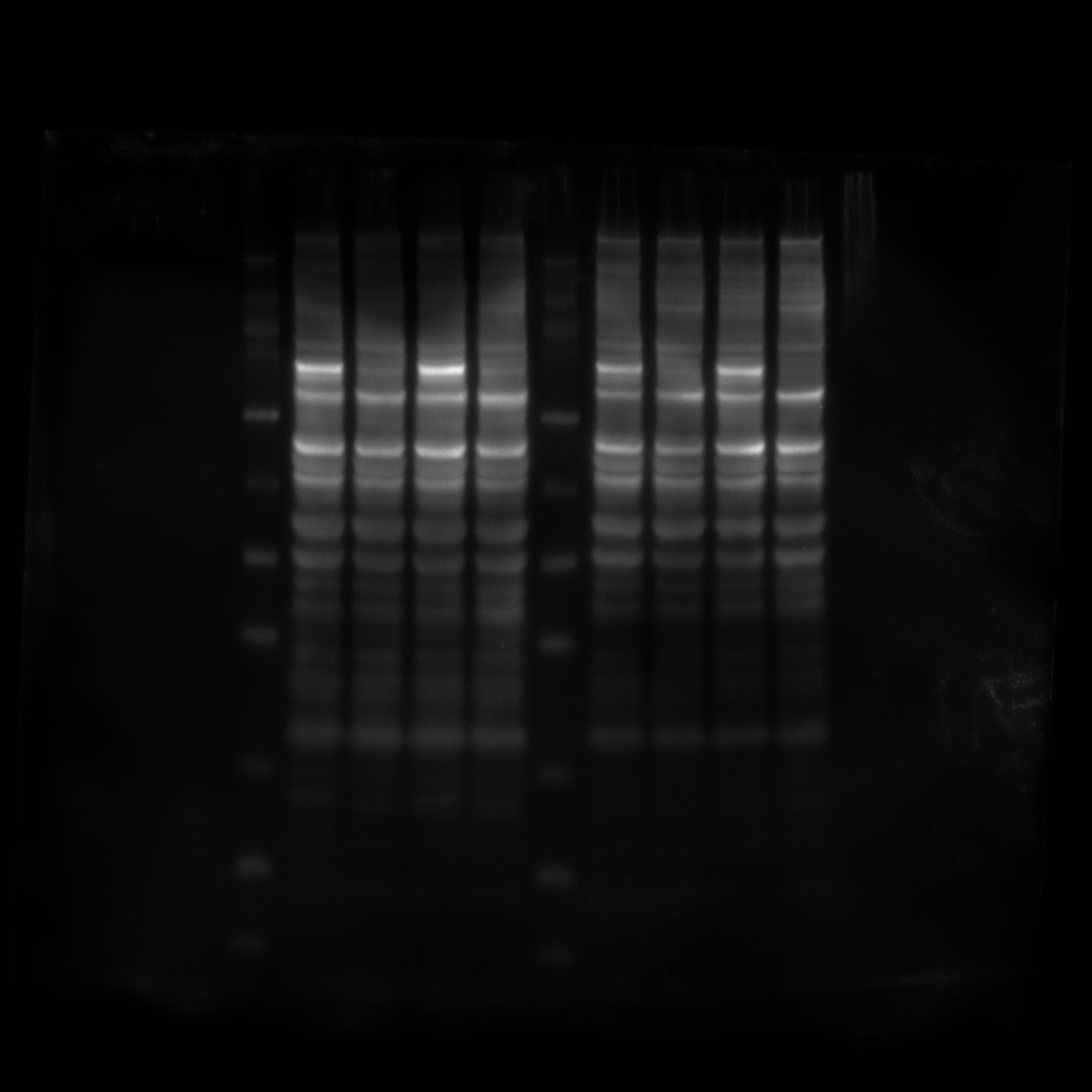

Supplement: Figure 2—source data 3. [file elife-106901-fig2-data3.zip › Figure2 source data 3/Figure 2D TAB3 .Tif]

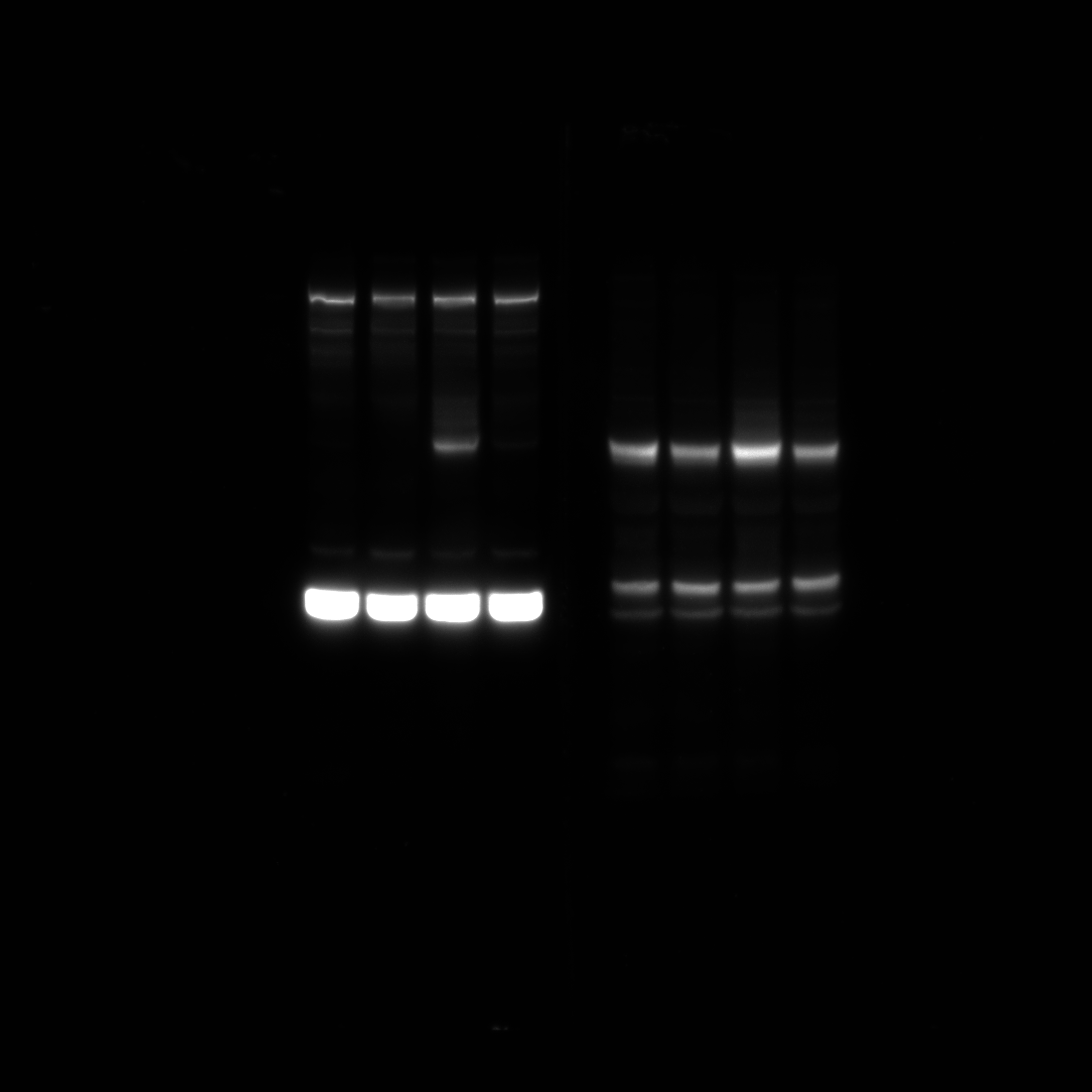

Supplement: Figure 2—source data 3. [file elife-106901-fig2-data3.zip › Figure2 source data 3/Figure 2D TAK1.Tif]

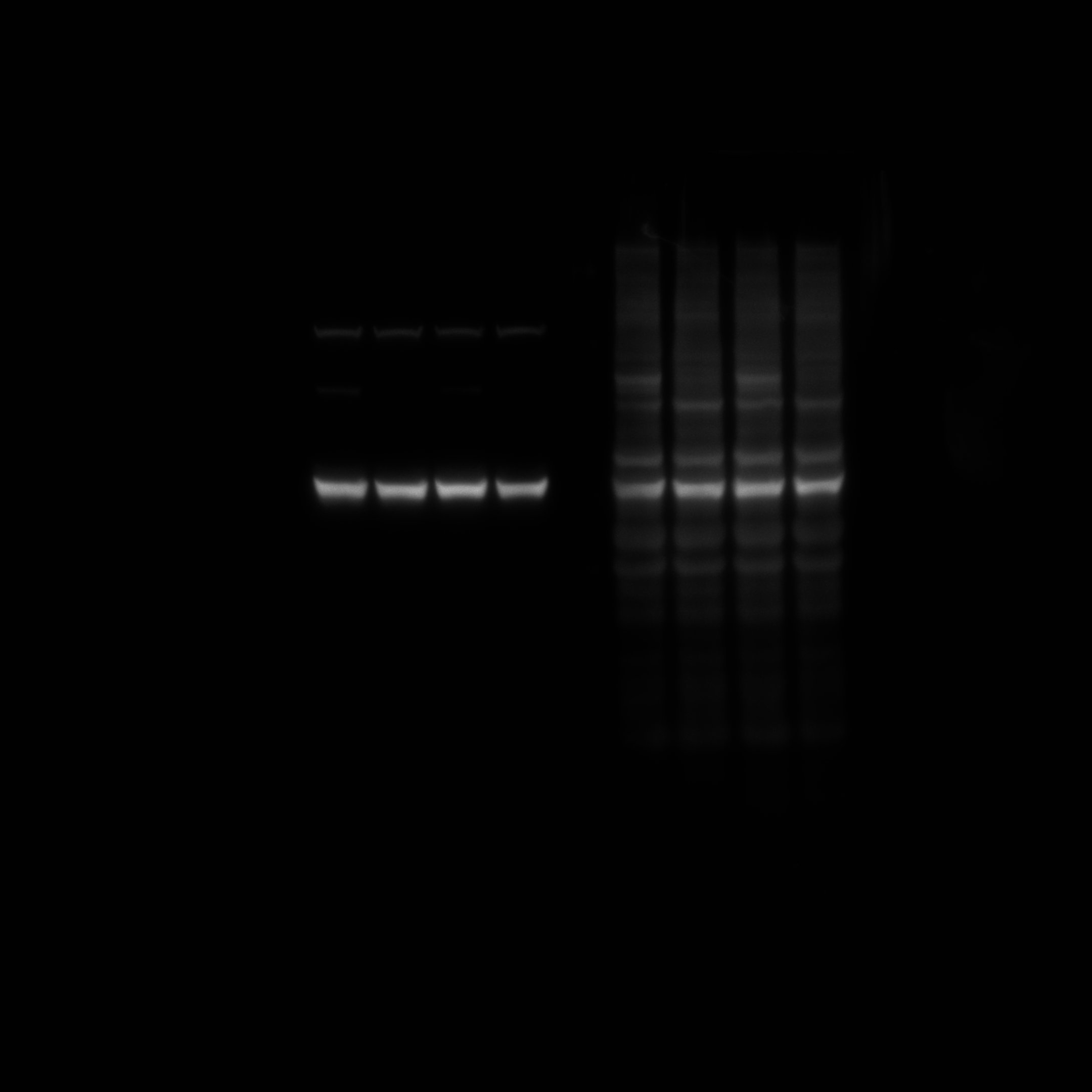

Supplement: Figure 2—source data 3. [file elife-106901-fig2-data3.zip › Figure2 source data 3/Figure 2D Tubulin.Tif]

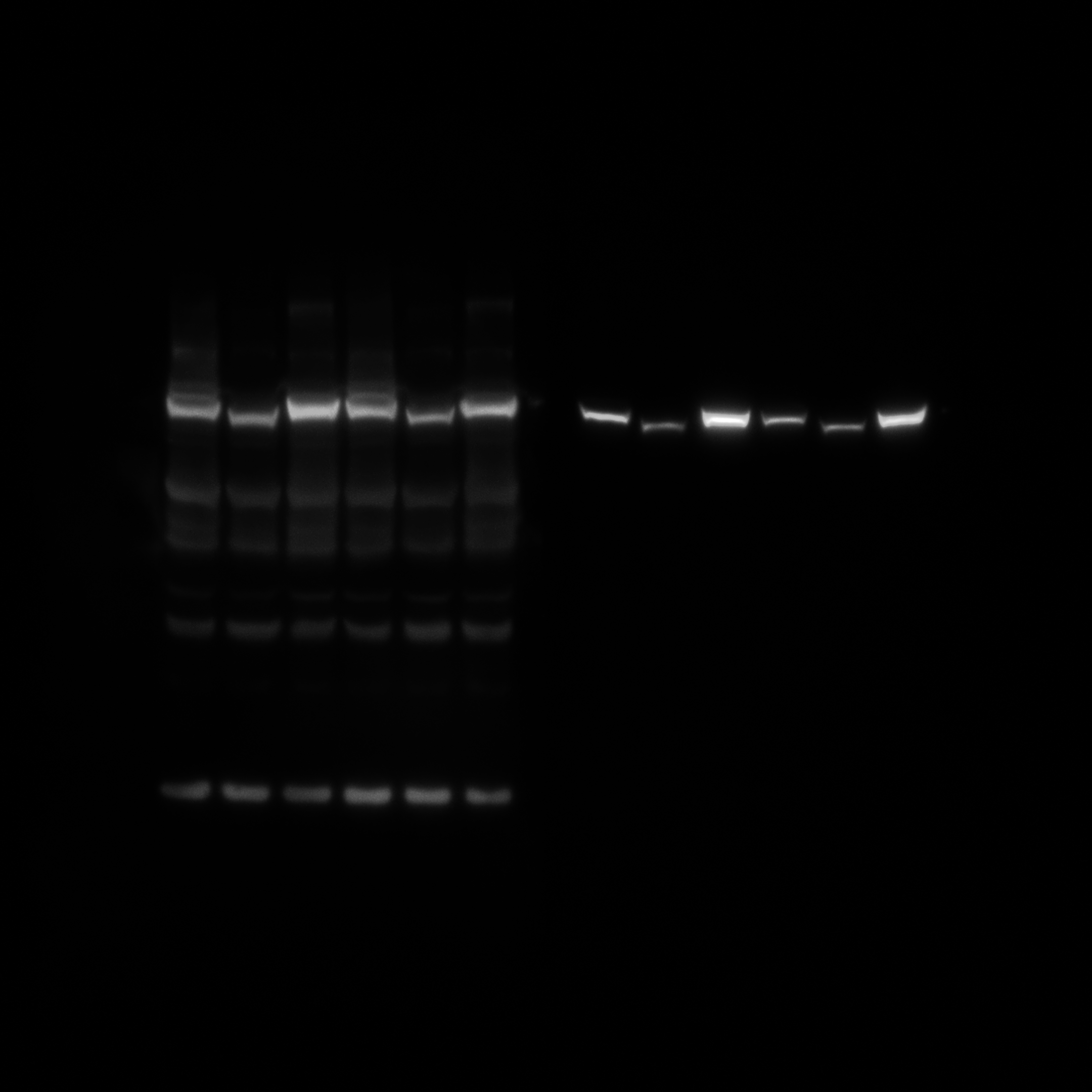

Supplement: Figure 2—source data 3. [file elife-106901-fig2-data3.zip › Figure2 source data 3/Figure 2E FLAG.Tif]

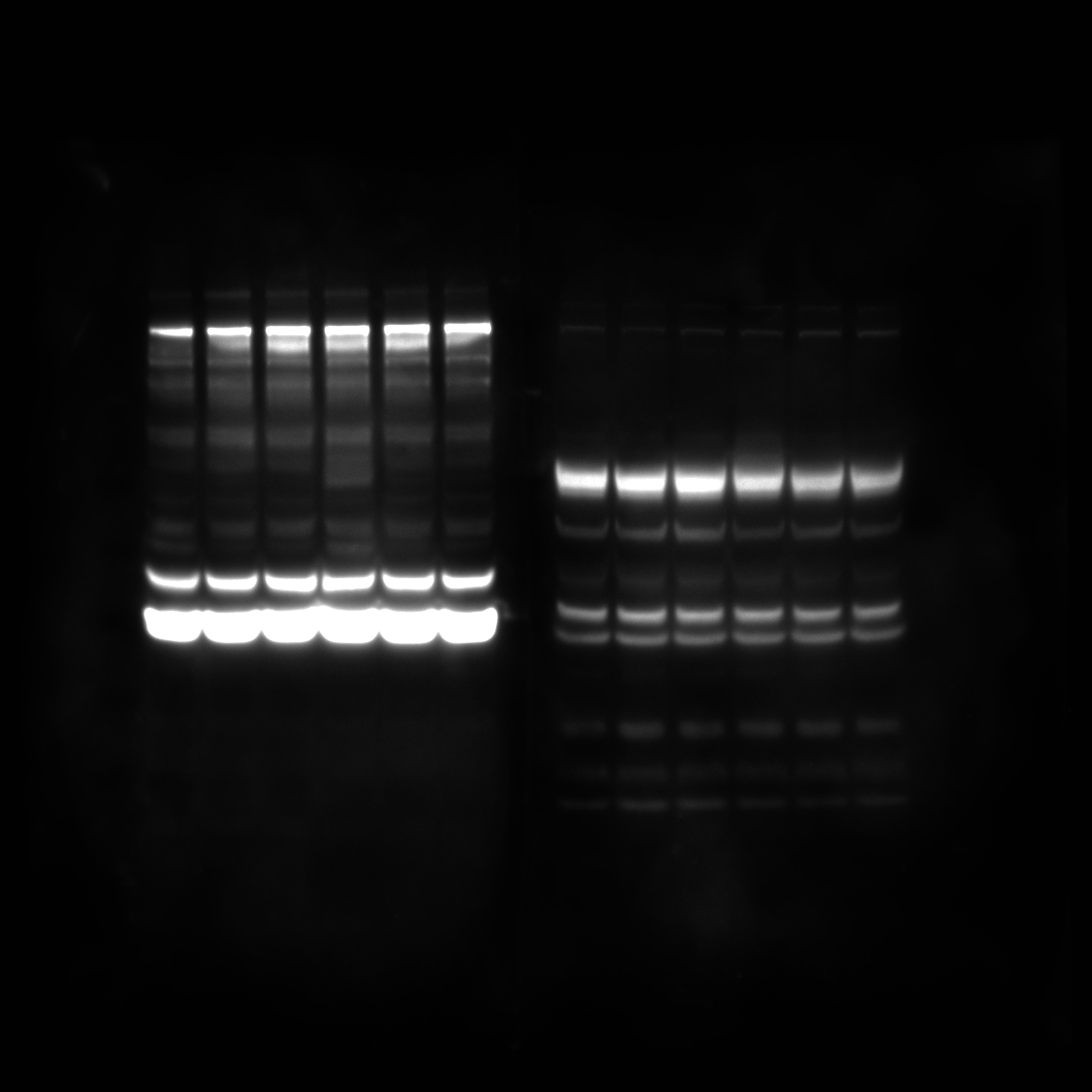

Supplement: Figure 2—source data 3. [file elife-106901-fig2-data3.zip › Figure2 source data 3/Figure 2E pTAK1.Tif]

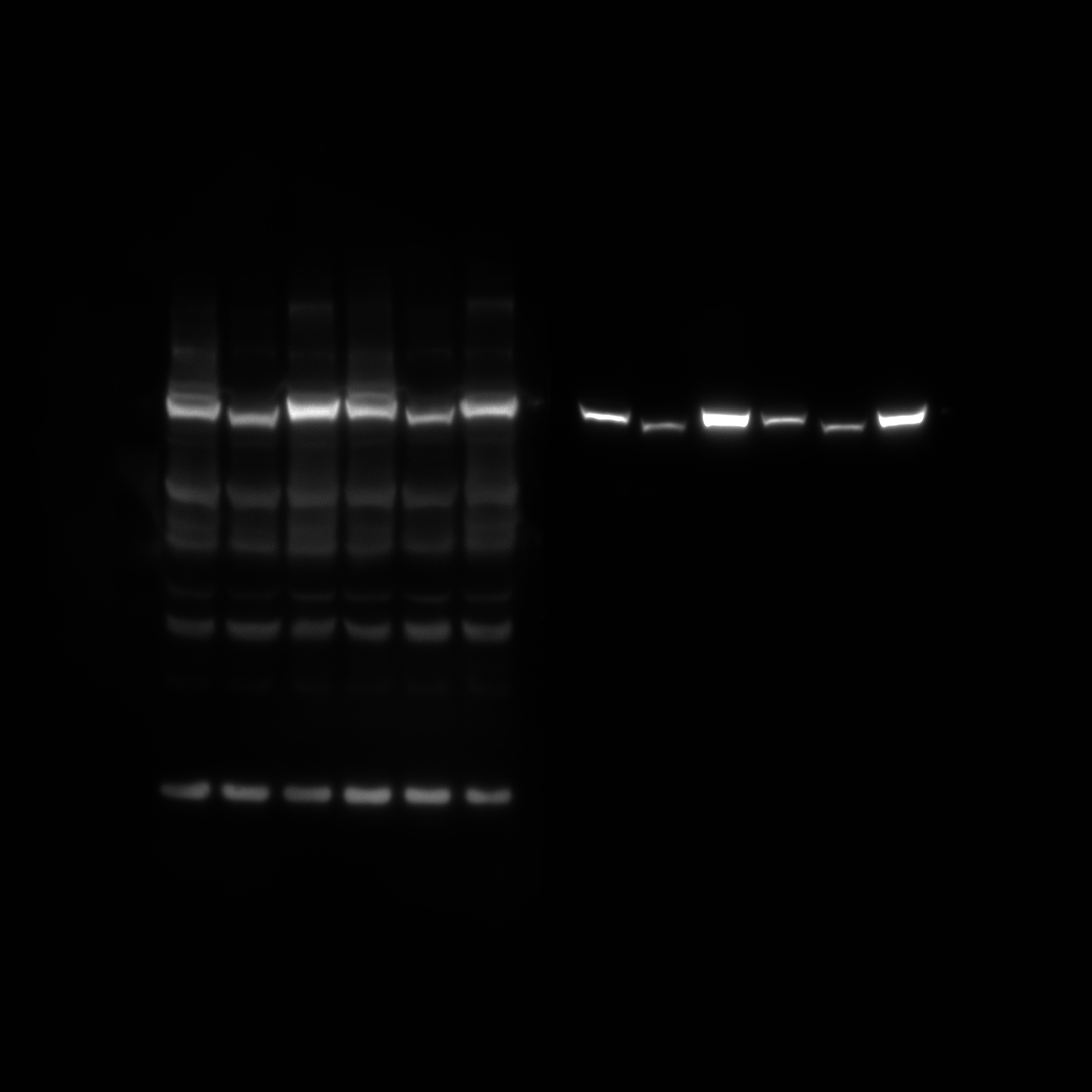

Supplement: Figure 2—source data 3. [file elife-106901-fig2-data3.zip › Figure2 source data 3/Figure 2E TAB2.Tif]

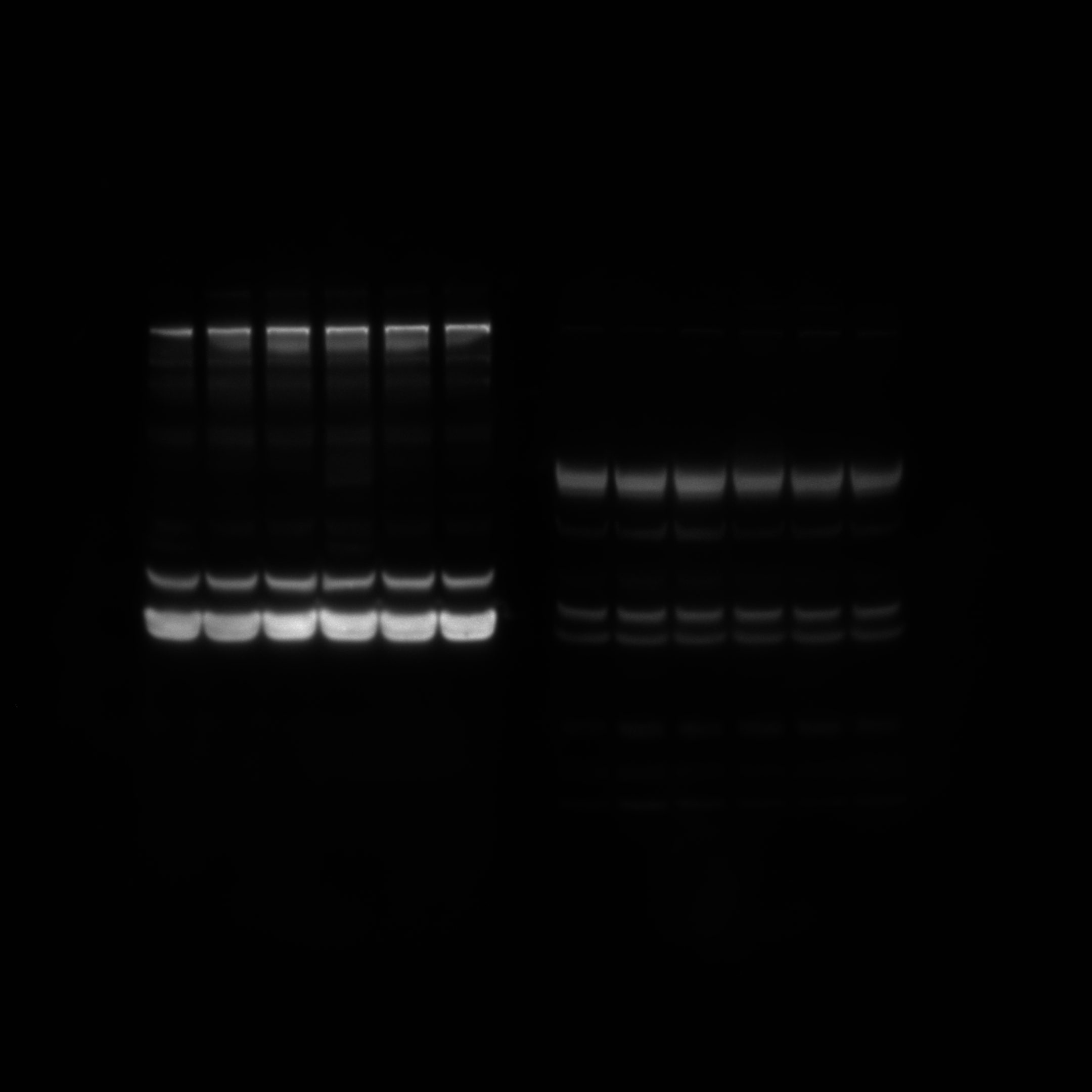

Supplement: Figure 2—source data 3. [file elife-106901-fig2-data3.zip › Figure2 source data 3/Figure 2E TAK1.Tif]

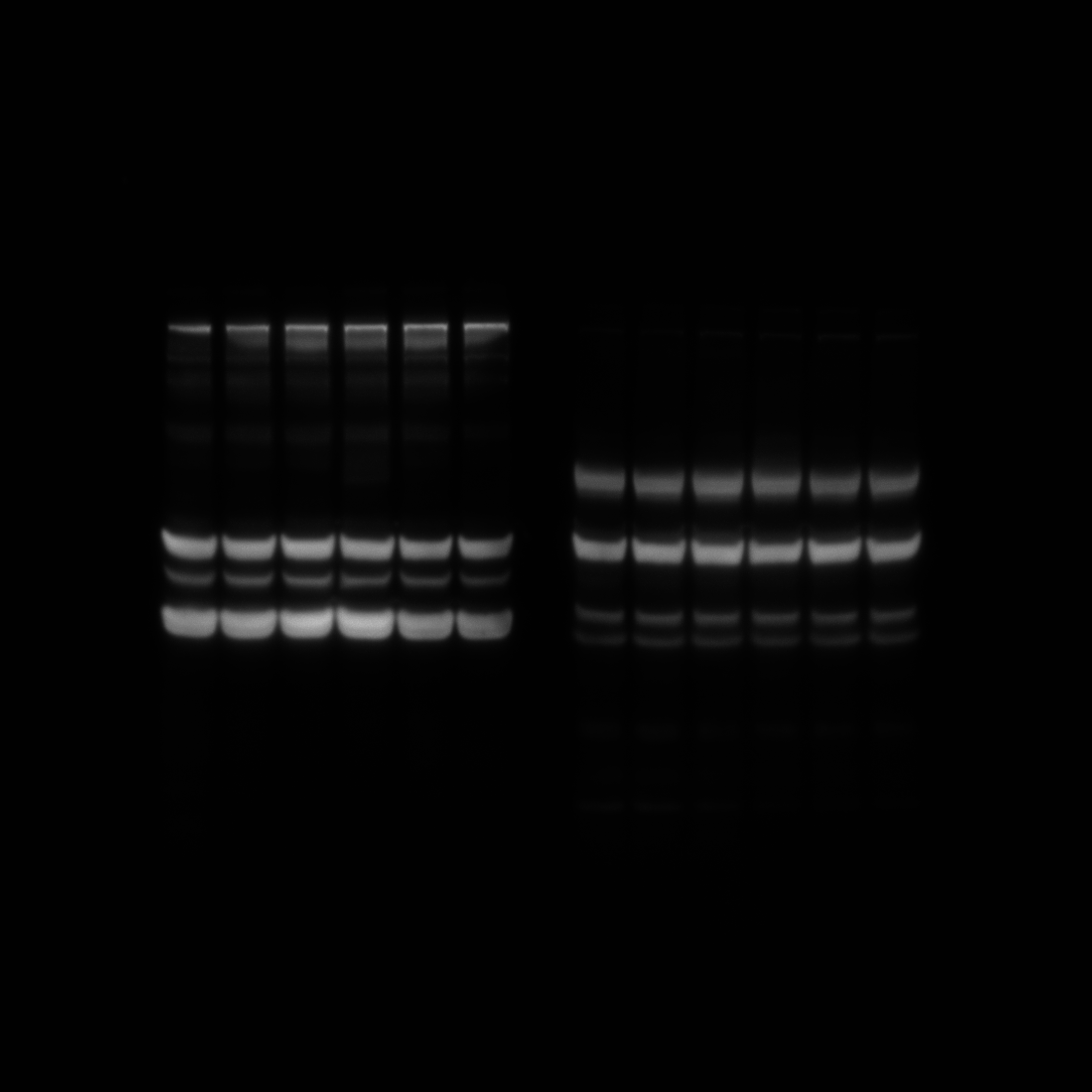

Supplement: Figure 2—source data 3. [file elife-106901-fig2-data3.zip › Figure2 source data 3/Figure 2E Tubulin.Tif]

**Figure 2D**

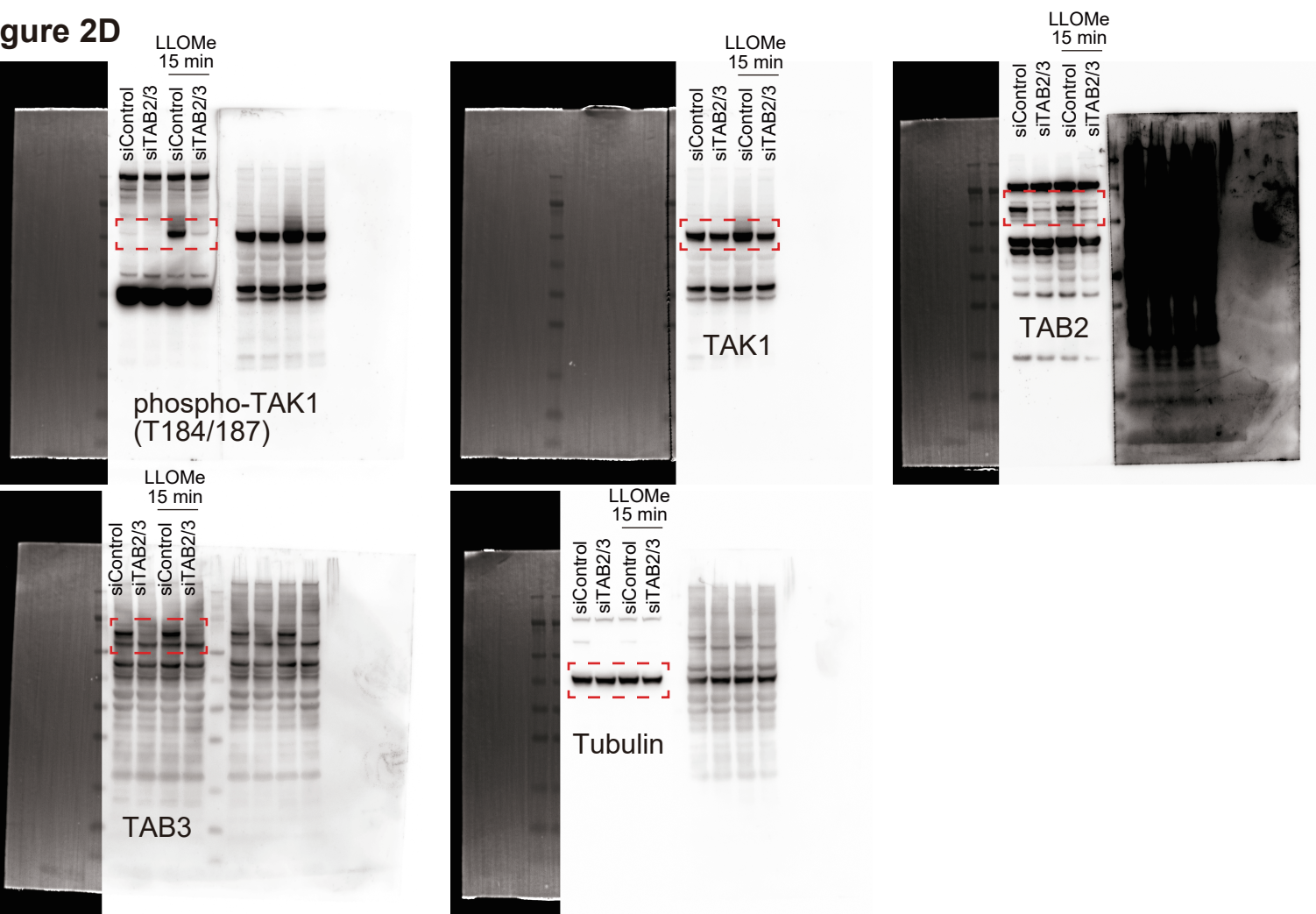

**Figure 2E**

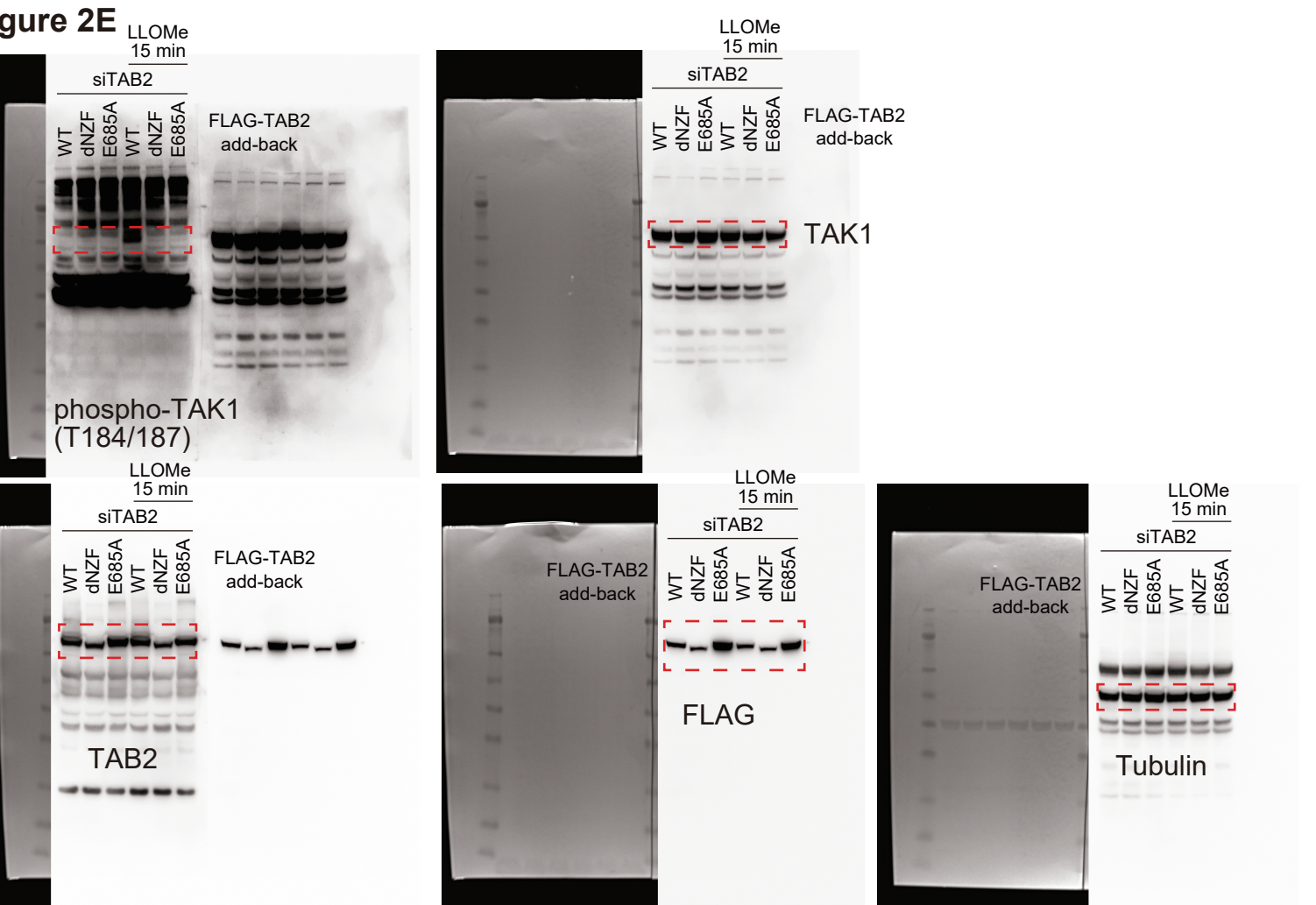

Supplement: Figure 2—source data 4. [file elife-106901-fig2-data4.pdf]

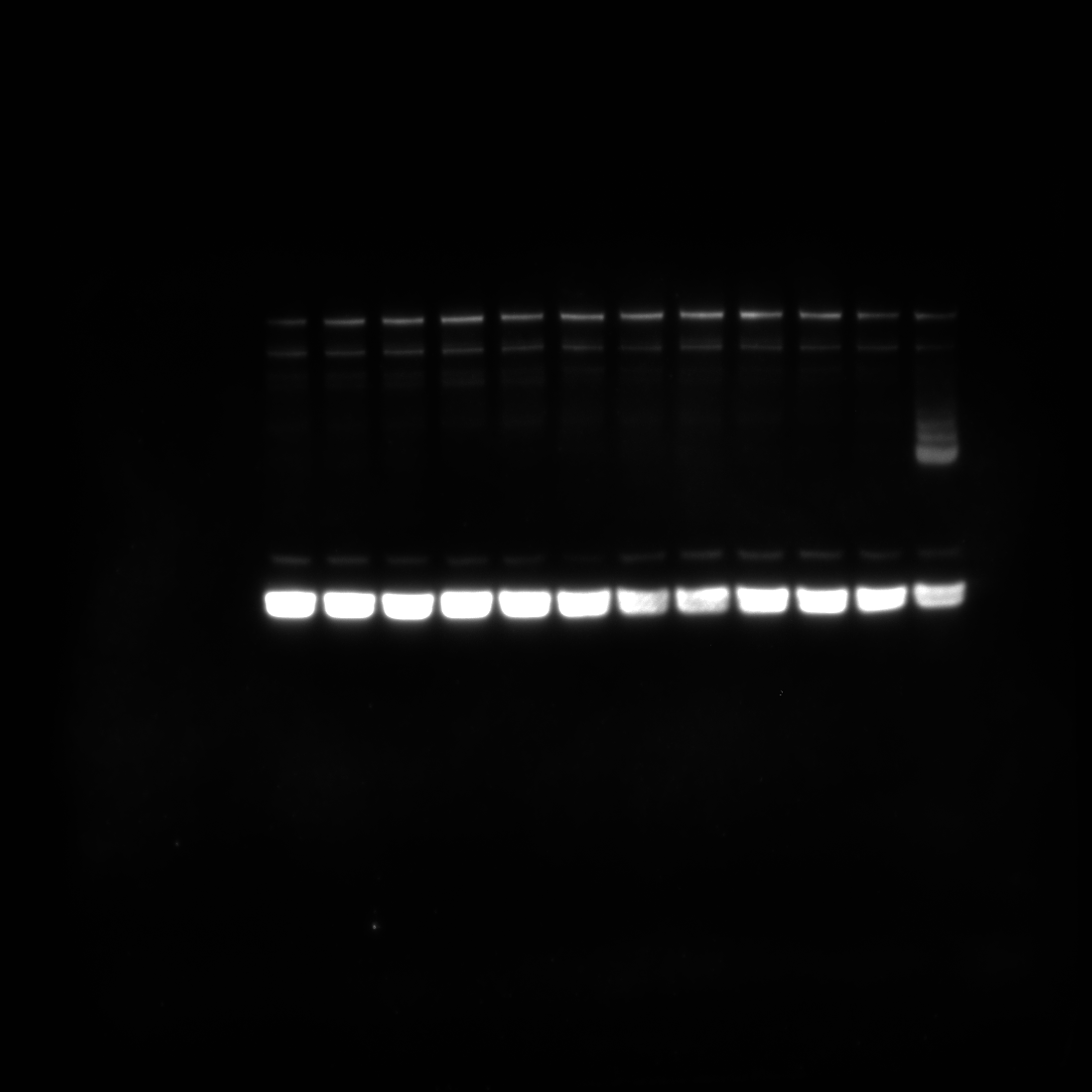

Supplement: Figure 2—figure supplement 1—source data 1. [file elife-106901-fig2-figsupp1-data1.zip › Figure2 figure supplement 1 source data 1/Figure S2E pTAK1.Tif]

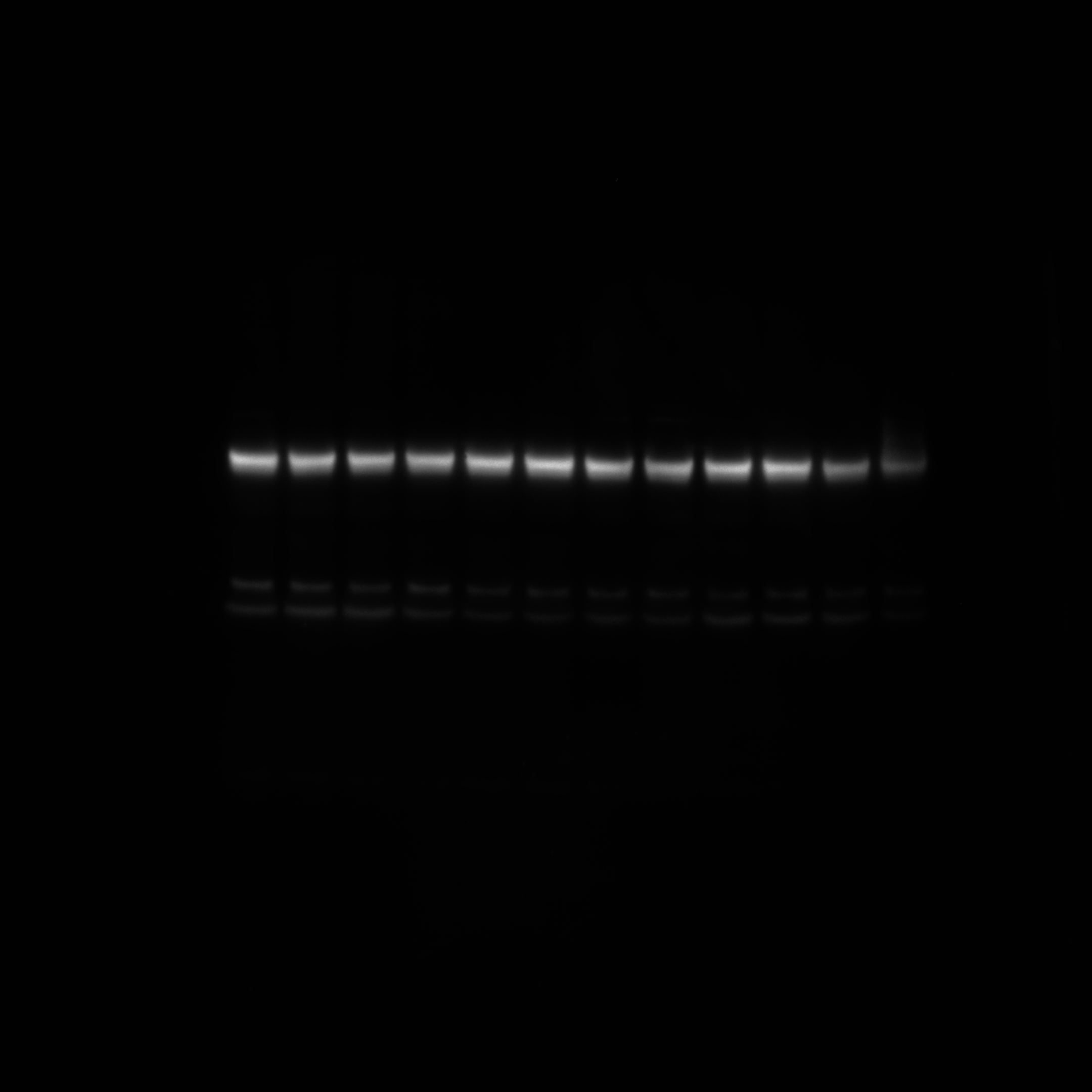

Supplement: Figure 2—figure supplement 1—source data 1. [file elife-106901-fig2-figsupp1-data1.zip › Figure2 figure supplement 1 source data 1/Figure S2E TAK1.Tif]

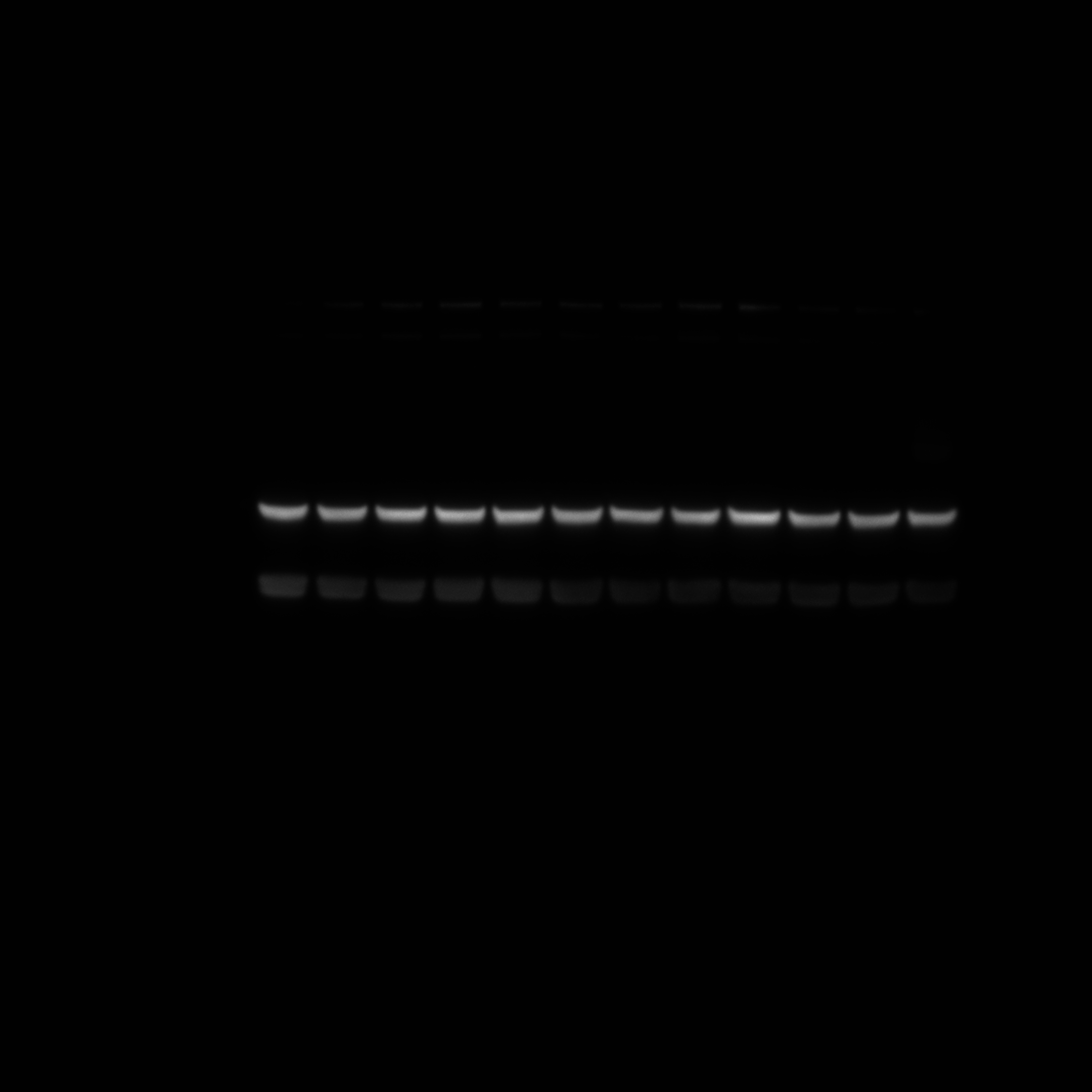

Supplement: Figure 2—figure supplement 1—source data 1. [file elife-106901-fig2-figsupp1-data1.zip › Figure2 figure supplement 1 source data 1/Figure S2E Tubulin.Tif]

### Figure S2E

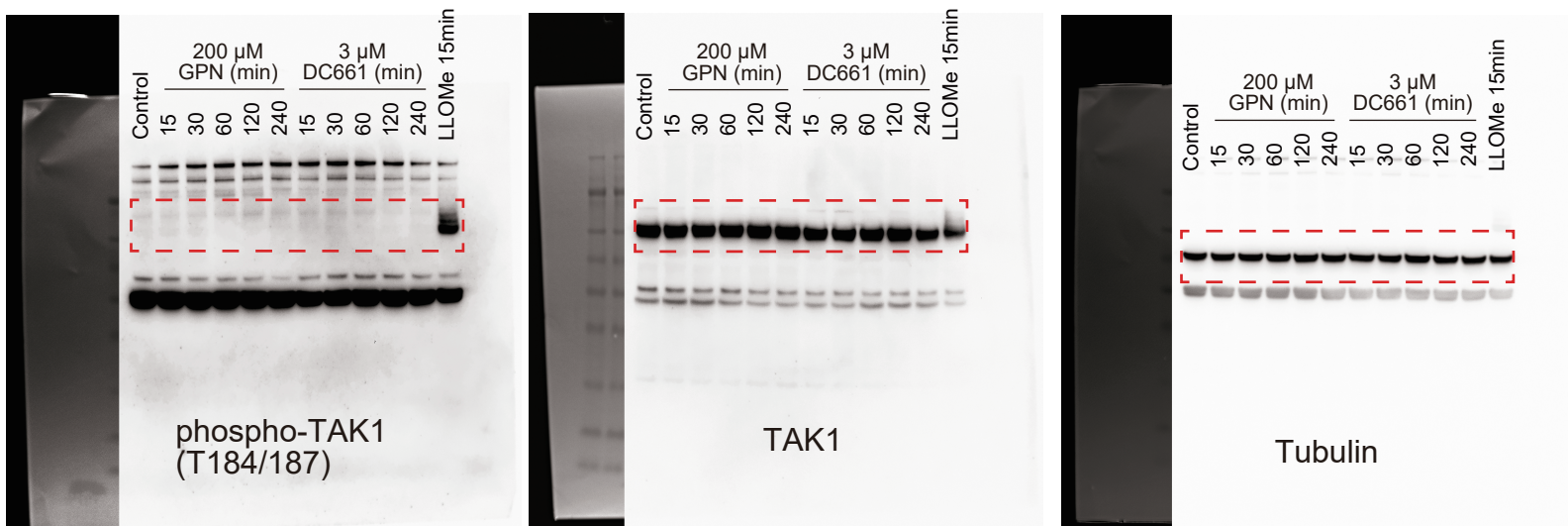

Supplement: Figure 2—figure supplement 1—source data 2. [file elife-106901-fig2-figsupp1-data2.pdf]

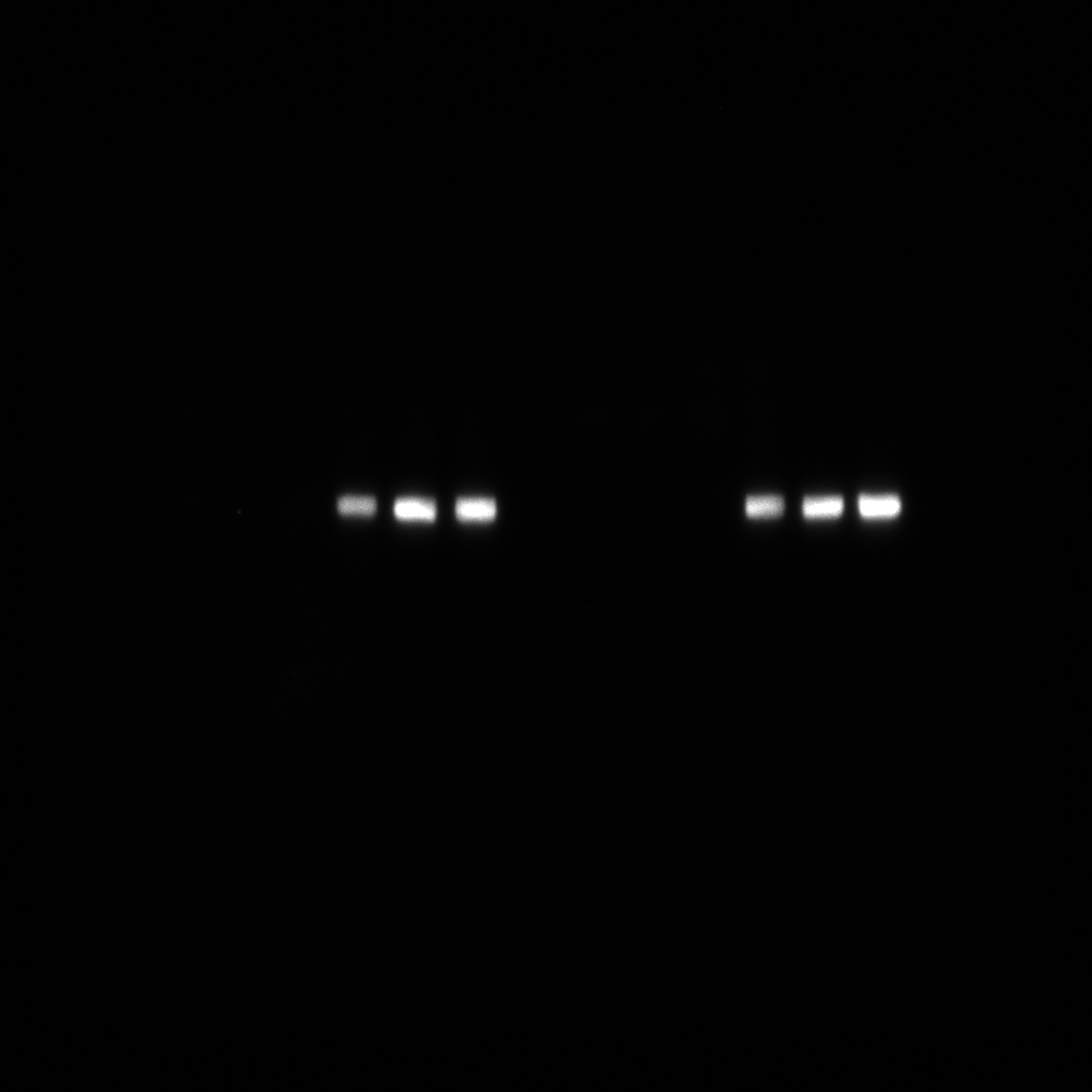

Supplement: Figure 3—source data 1. [file elife-106901-fig3-data1.zip › Figure3 source data 1/Figure 3F Fos.Tif]

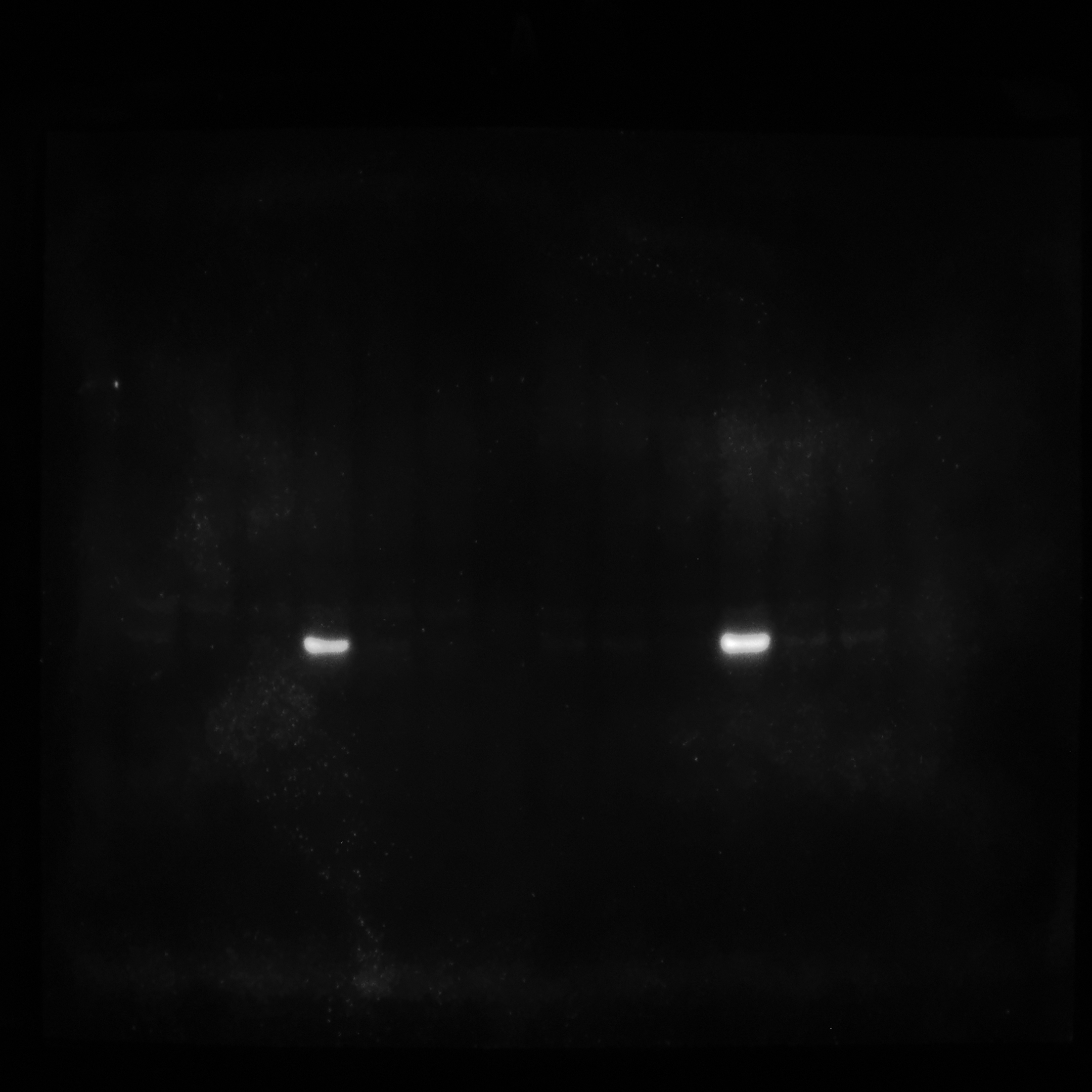

Supplement: Figure 3—source data 1. [file elife-106901-fig3-data1.zip › Figure3 source data 1/Figure 3F IL1b.Tif]

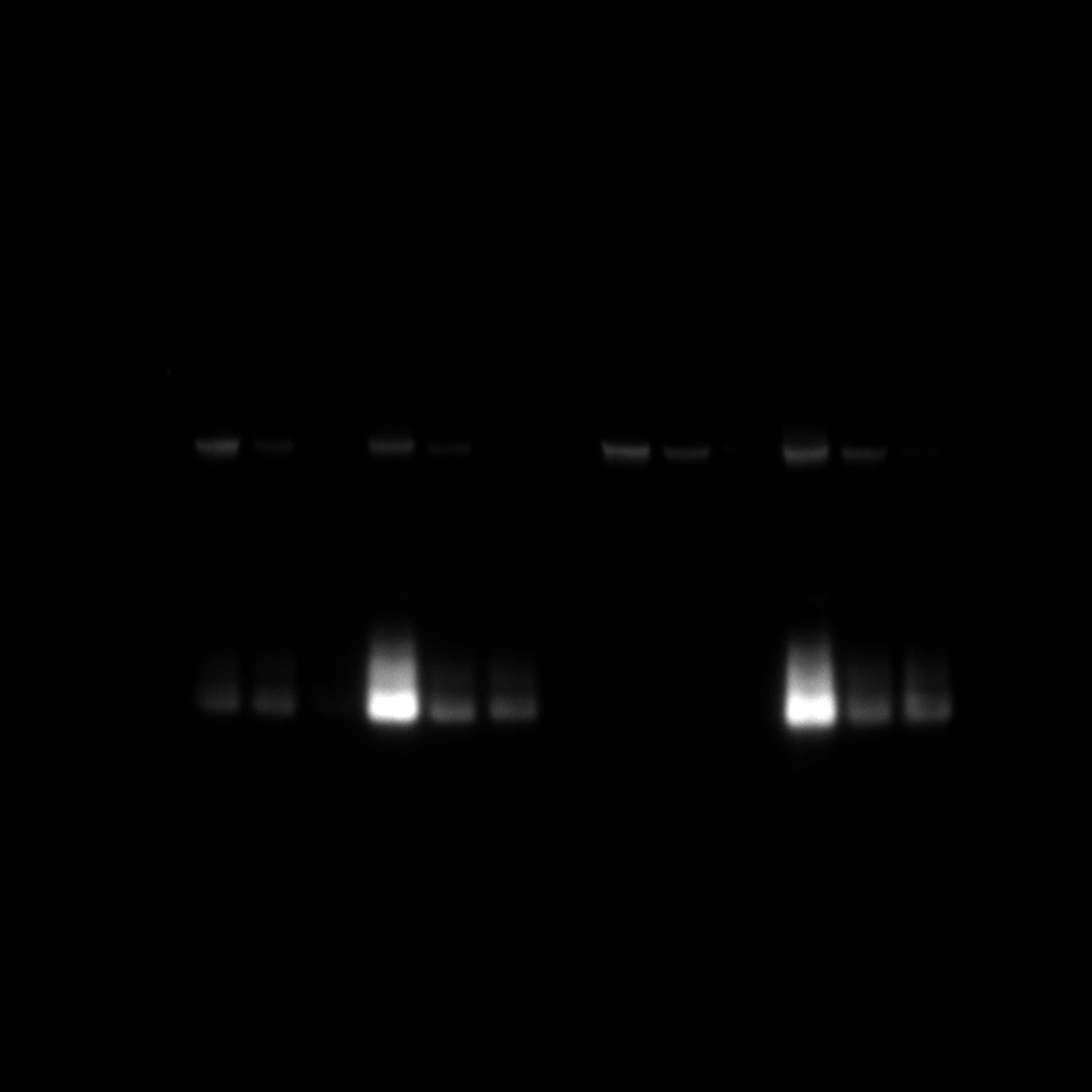

Supplement: Figure 3—source data 1. [file elife-106901-fig3-data1.zip › Figure3 source data 1/Figure 3F IL6.Tif]

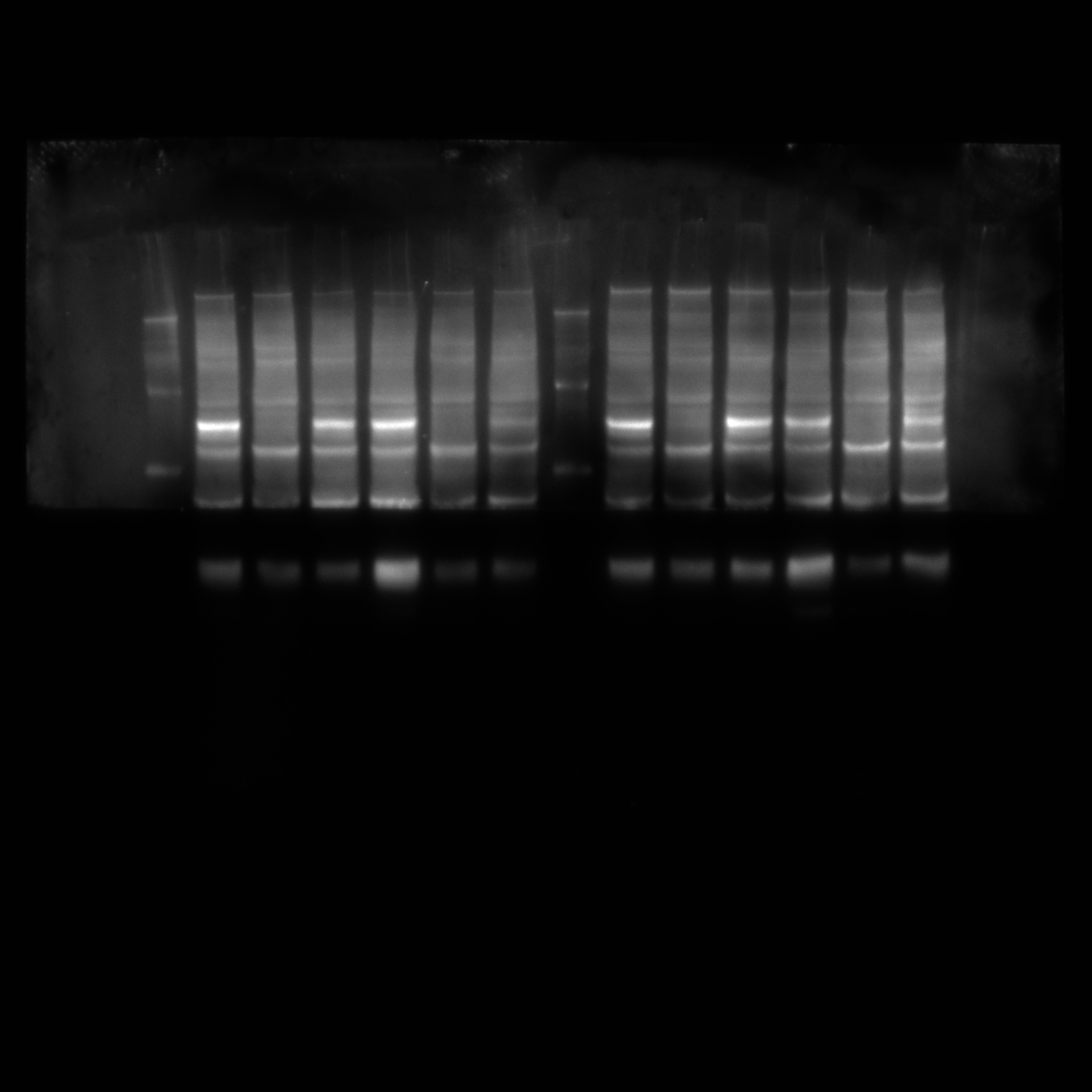

Supplement: Figure 3—source data 1. [file elife-106901-fig3-data1.zip › Figure3 source data 1/Figure 3F IRF1.Tif]

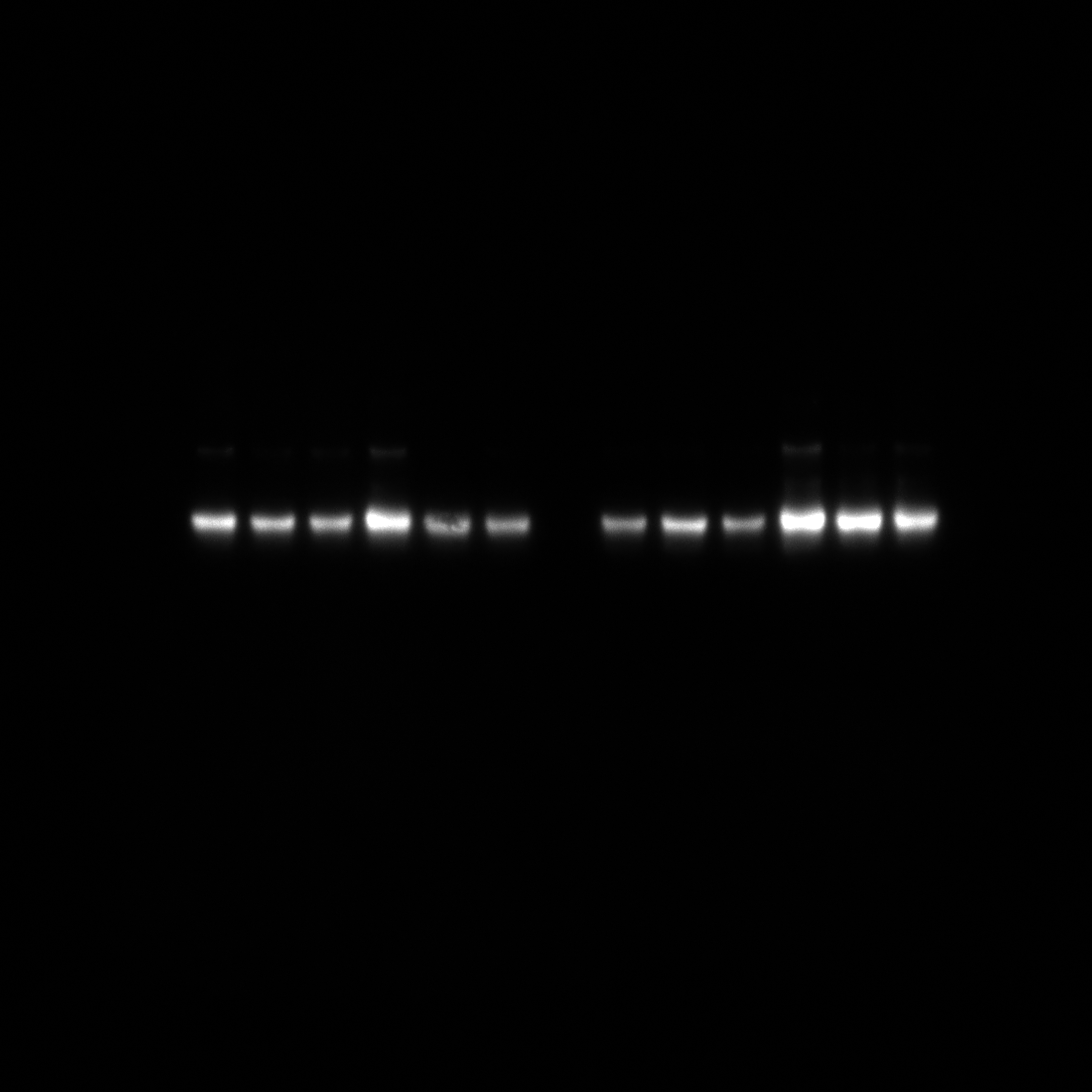

Supplement: Figure 3—source data 1. [file elife-106901-fig3-data1.zip › Figure3 source data 1/Figure 3F Jun.Tif]

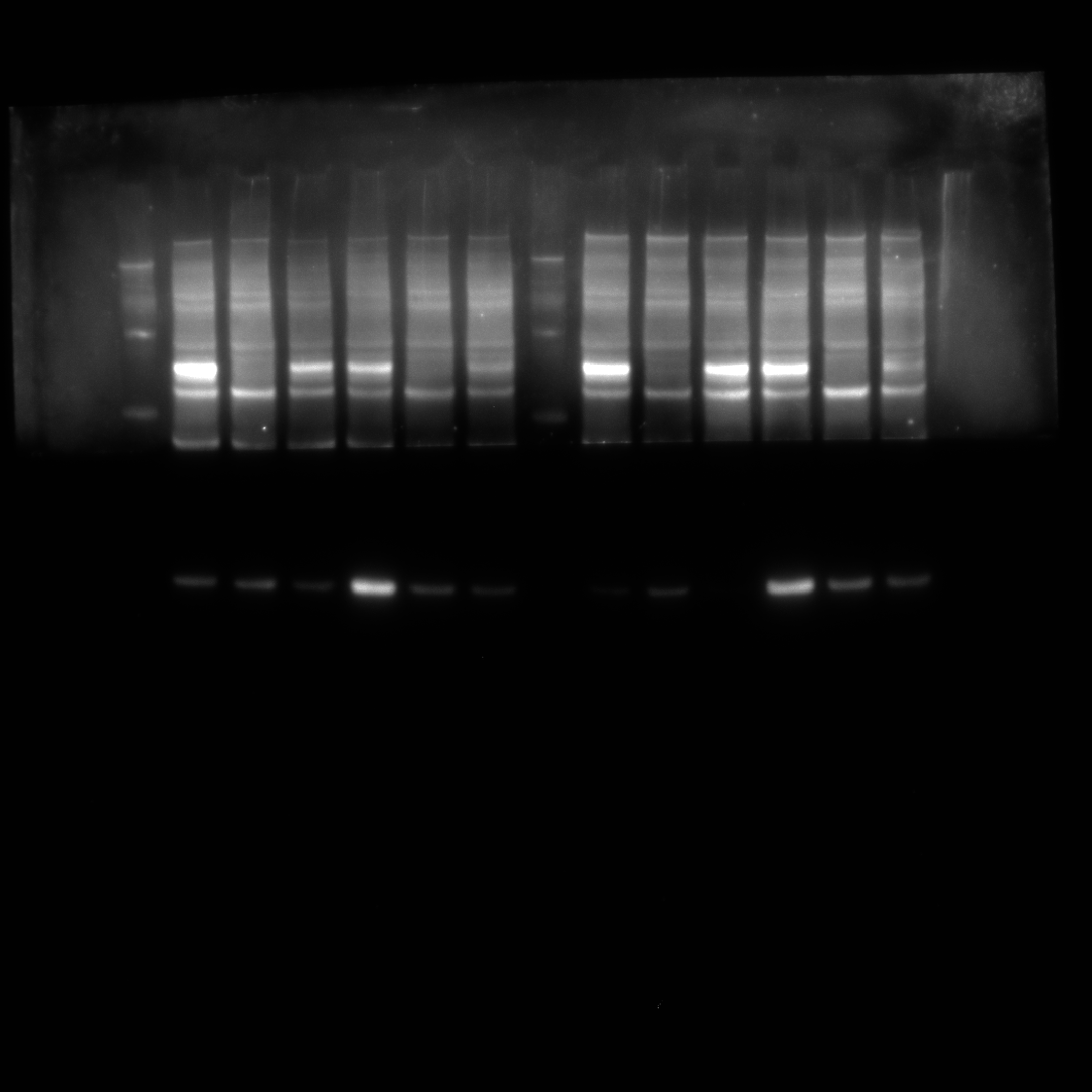

Supplement: Figure 3—source data 1. [file elife-106901-fig3-data1.zip › Figure3 source data 1/Figure 3F NKX31.Tif]

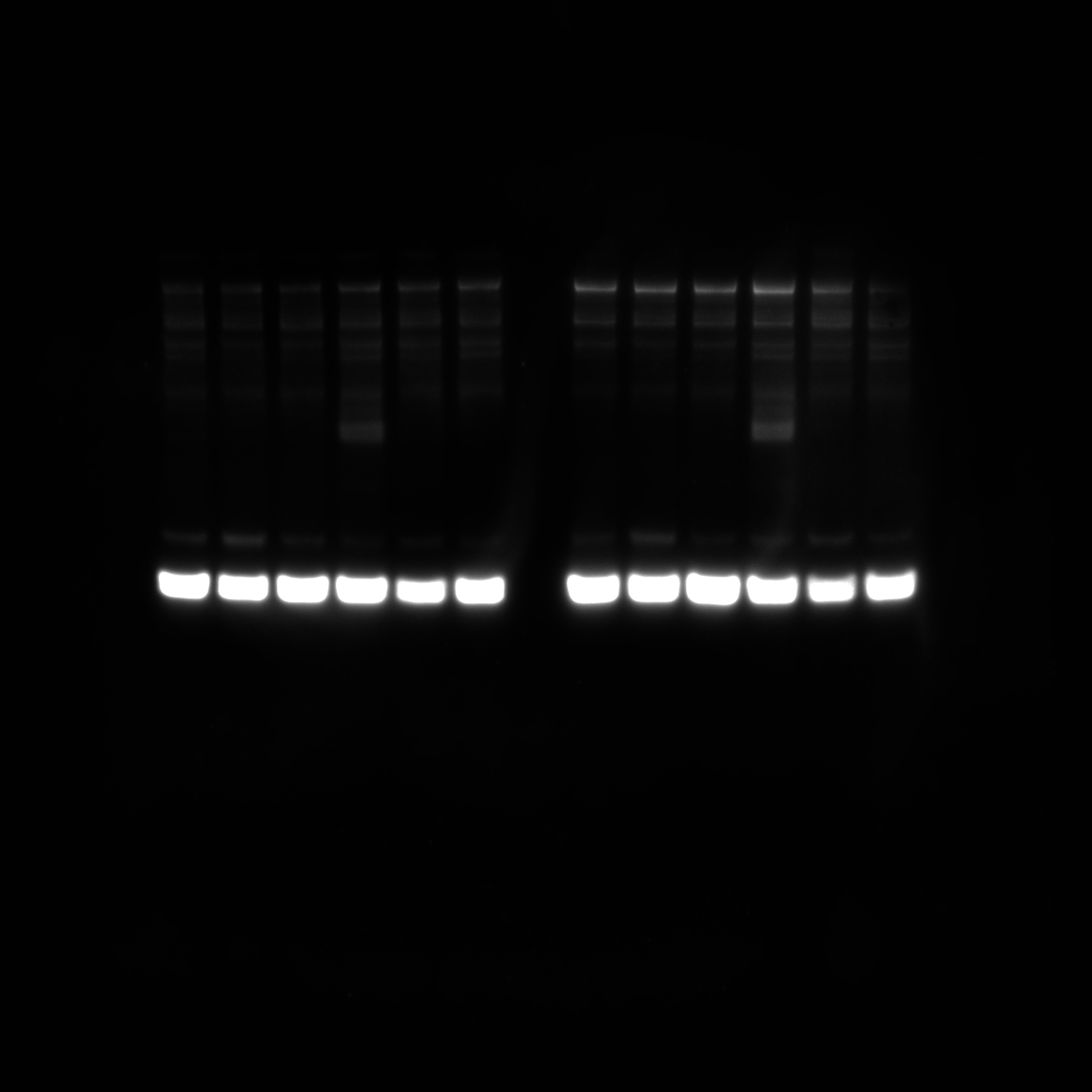

Supplement: Figure 3—source data 1. [file elife-106901-fig3-data1.zip › Figure3 source data 1/Figure 3F pTAK1.Tif]

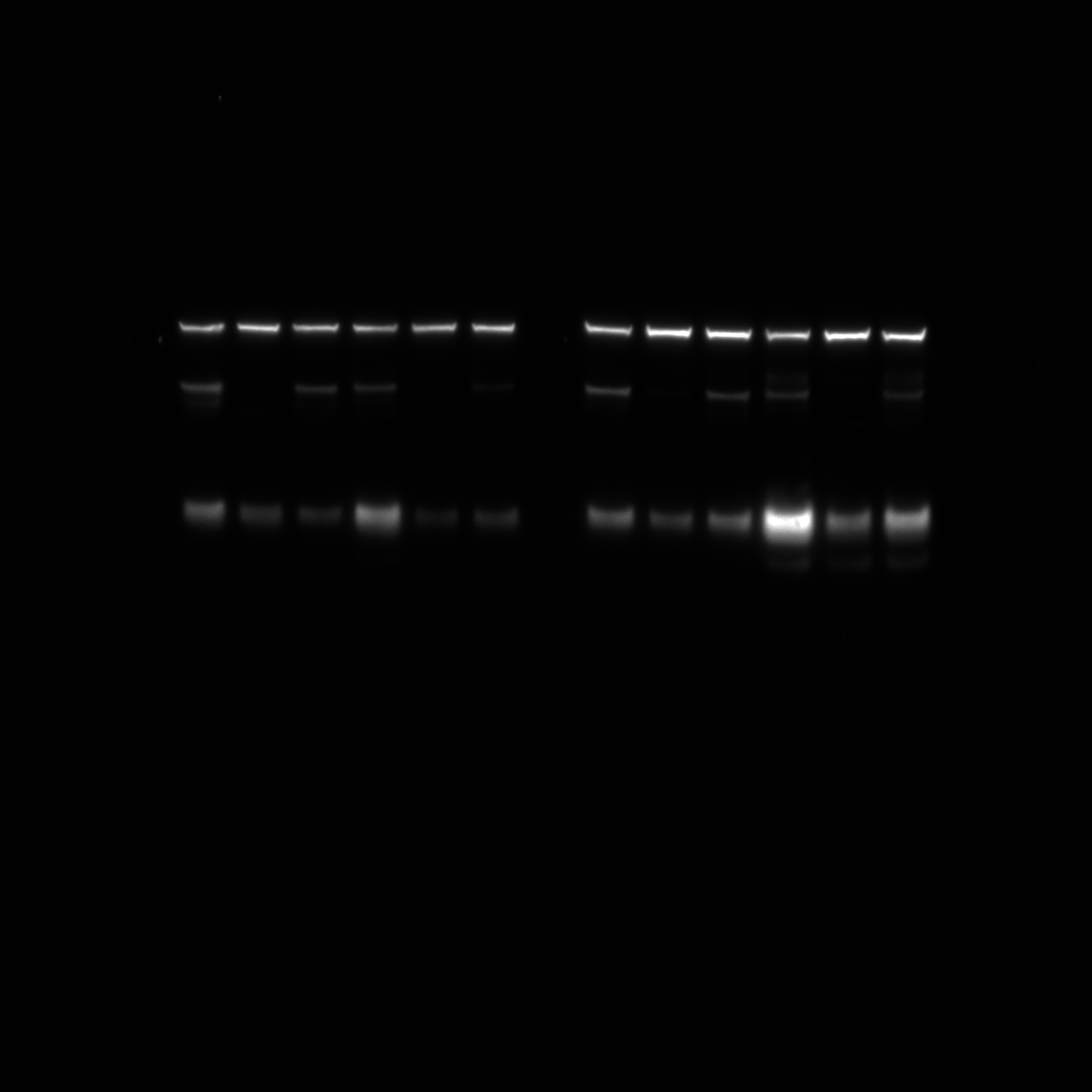

Supplement: Figure 3—source data 1. [file elife-106901-fig3-data1.zip › Figure3 source data 1/Figure 3F TAB2.Tif]

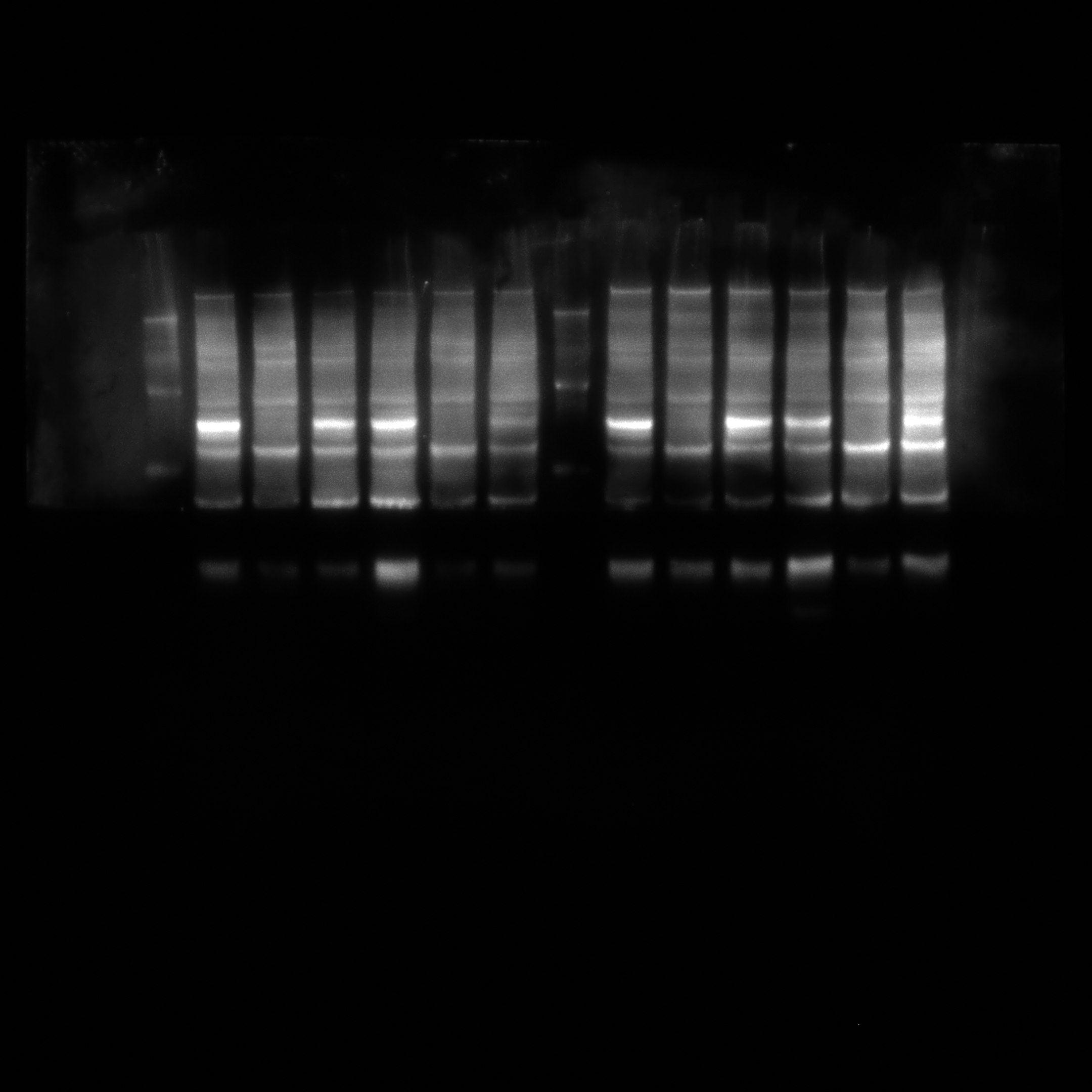

Supplement: Figure 3—source data 1. [file elife-106901-fig3-data1.zip › Figure3 source data 1/Figure 3F TAB3.Tif]

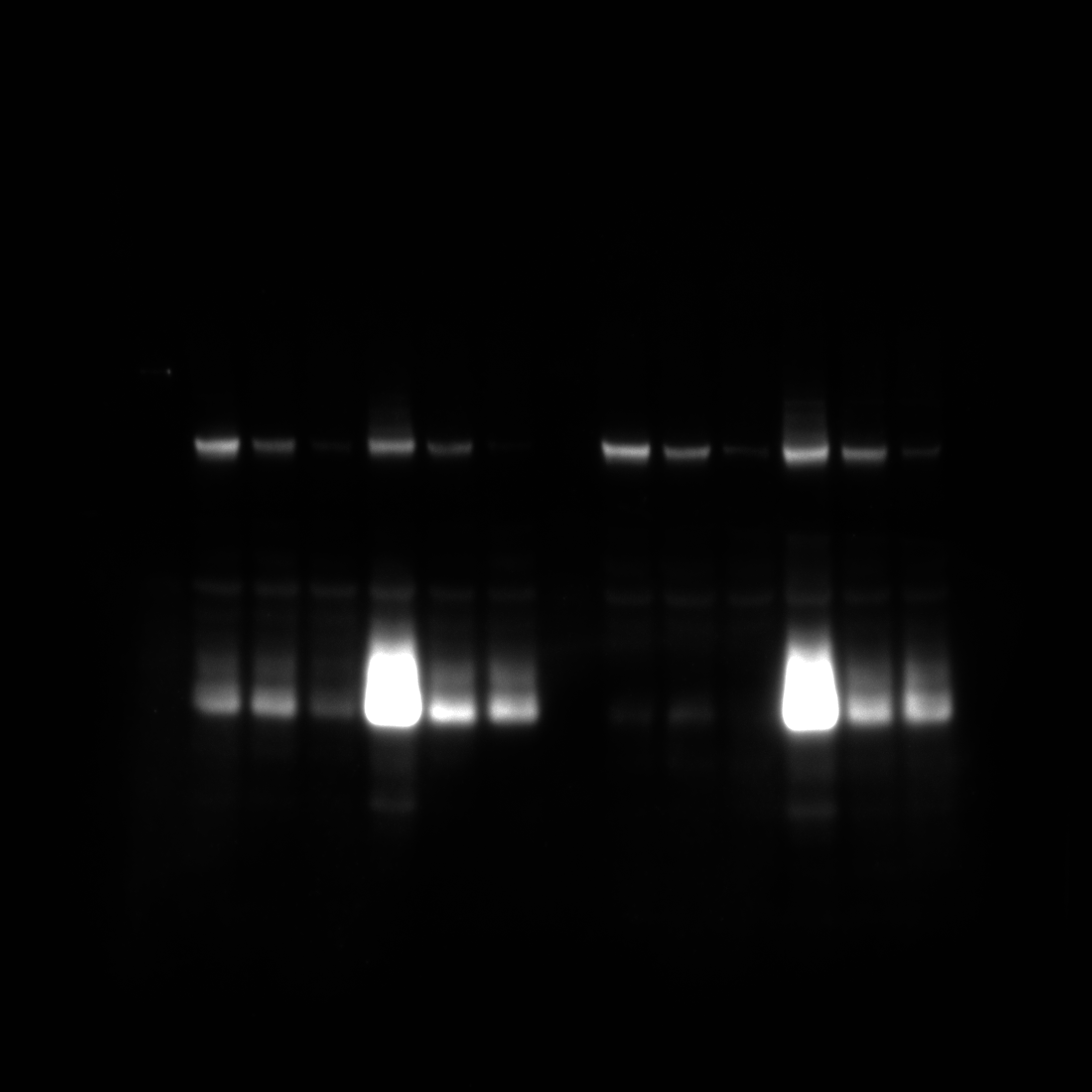

Supplement: Figure 3—source data 1. [file elife-106901-fig3-data1.zip › Figure3 source data 1/Figure 3F TAK1.Tif]

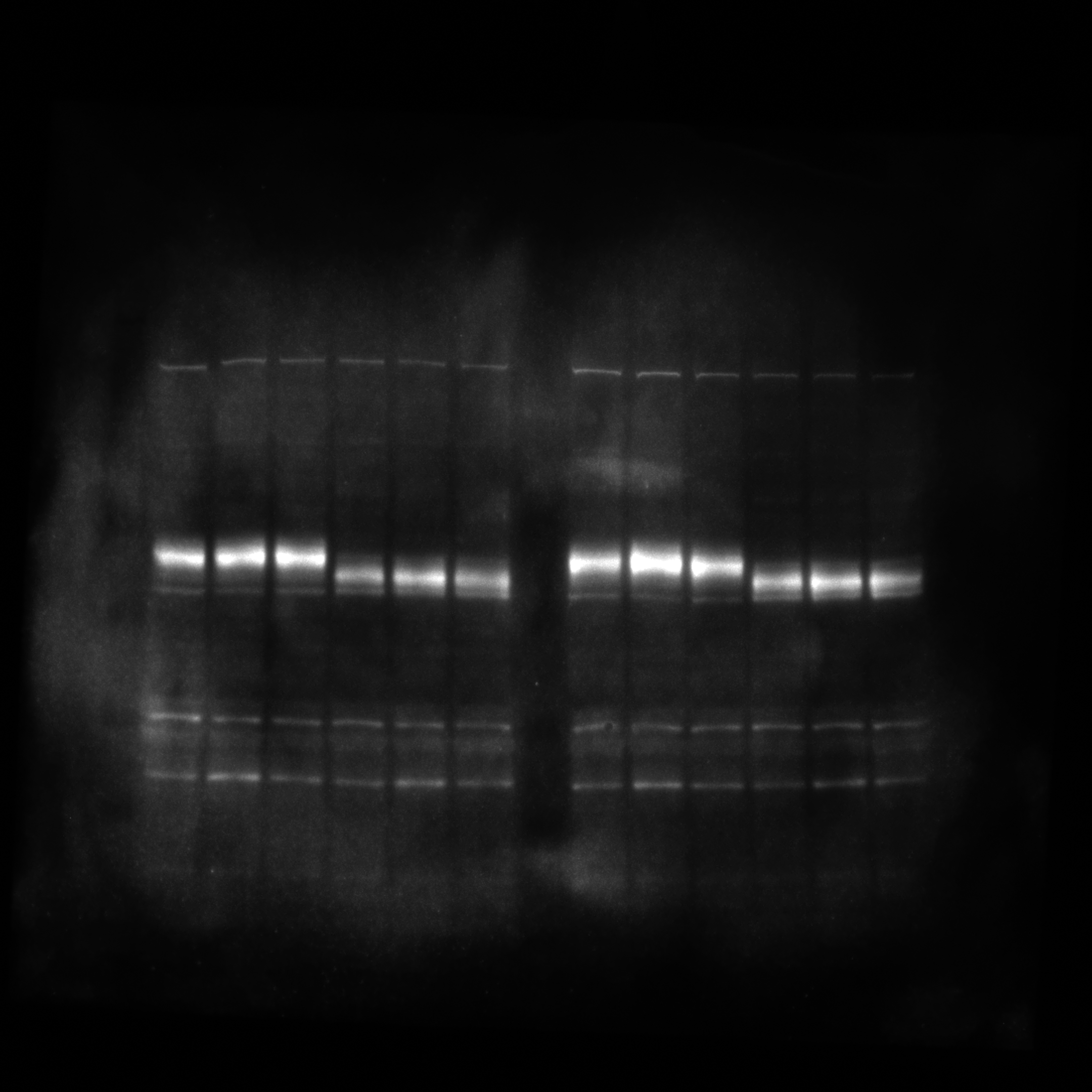

Supplement: Figure 3—source data 1. [file elife-106901-fig3-data1.zip › Figure3 source data 1/Figure 3F TFEB.Tif]

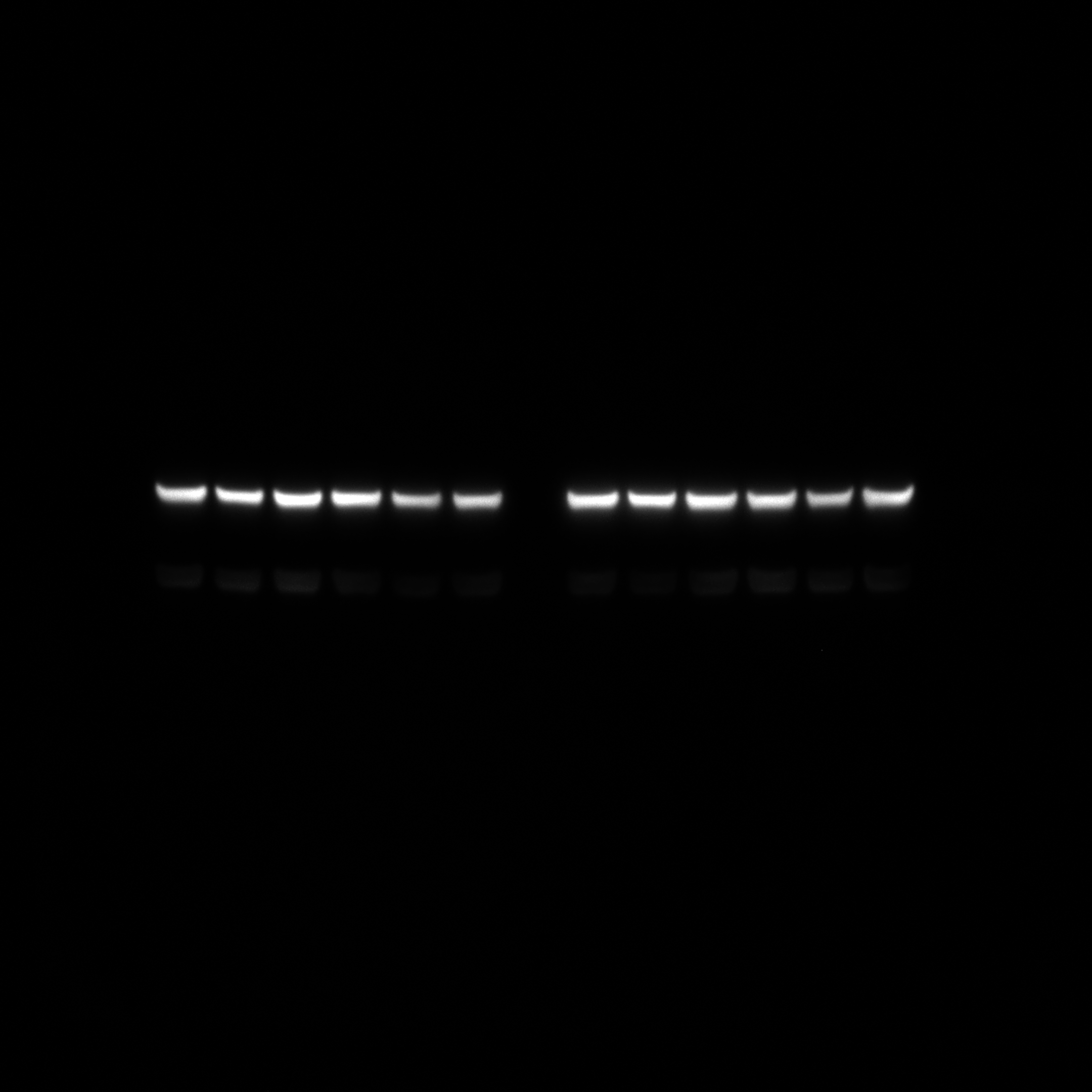

Supplement: Figure 3—source data 1. [file elife-106901-fig3-data1.zip › Figure3 source data 1/Figure 3F Tubulin.Tif]

**Figure 3F**

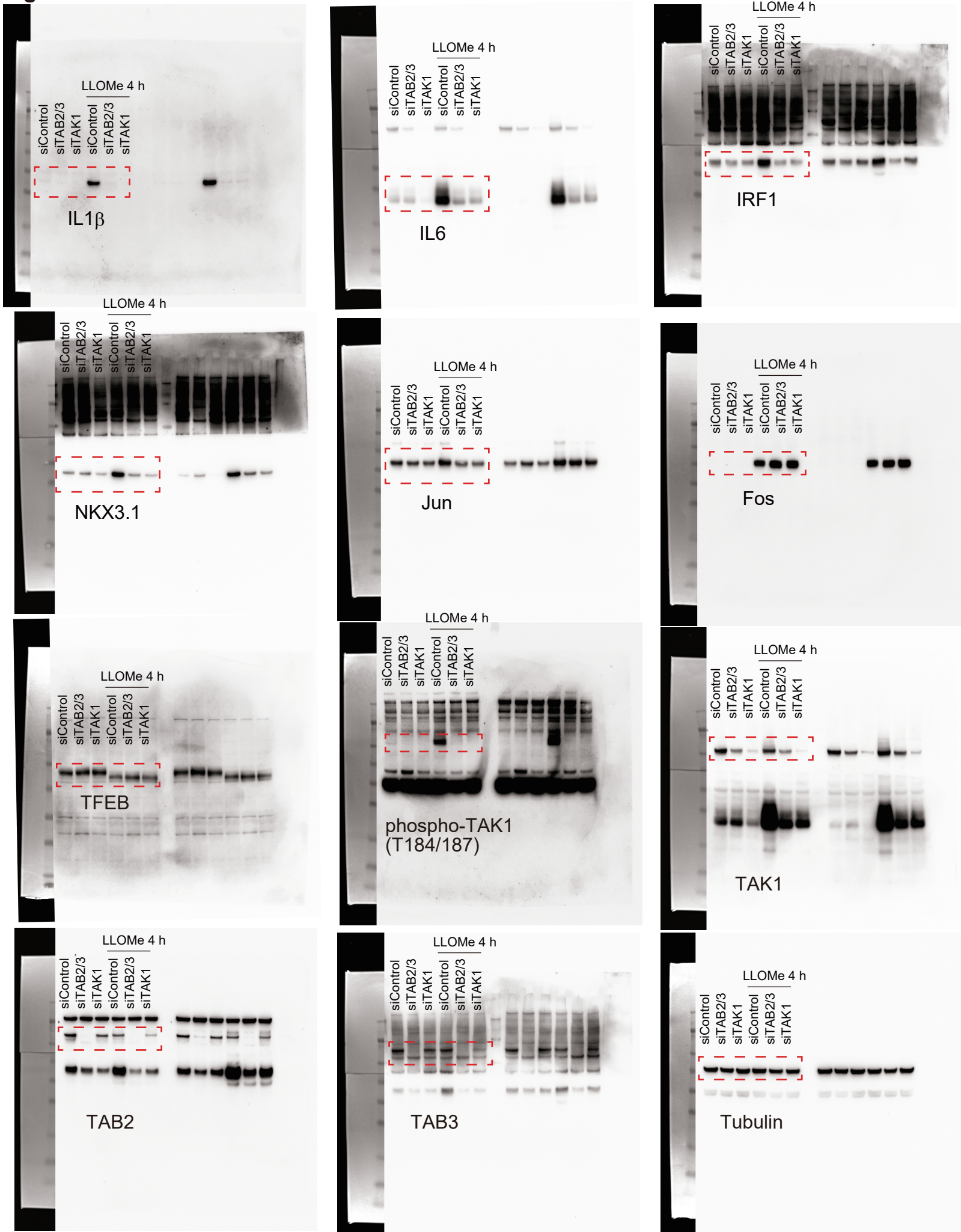

Supplement: Figure 3—source data 2. [file elife-106901-fig3-data2.pdf]

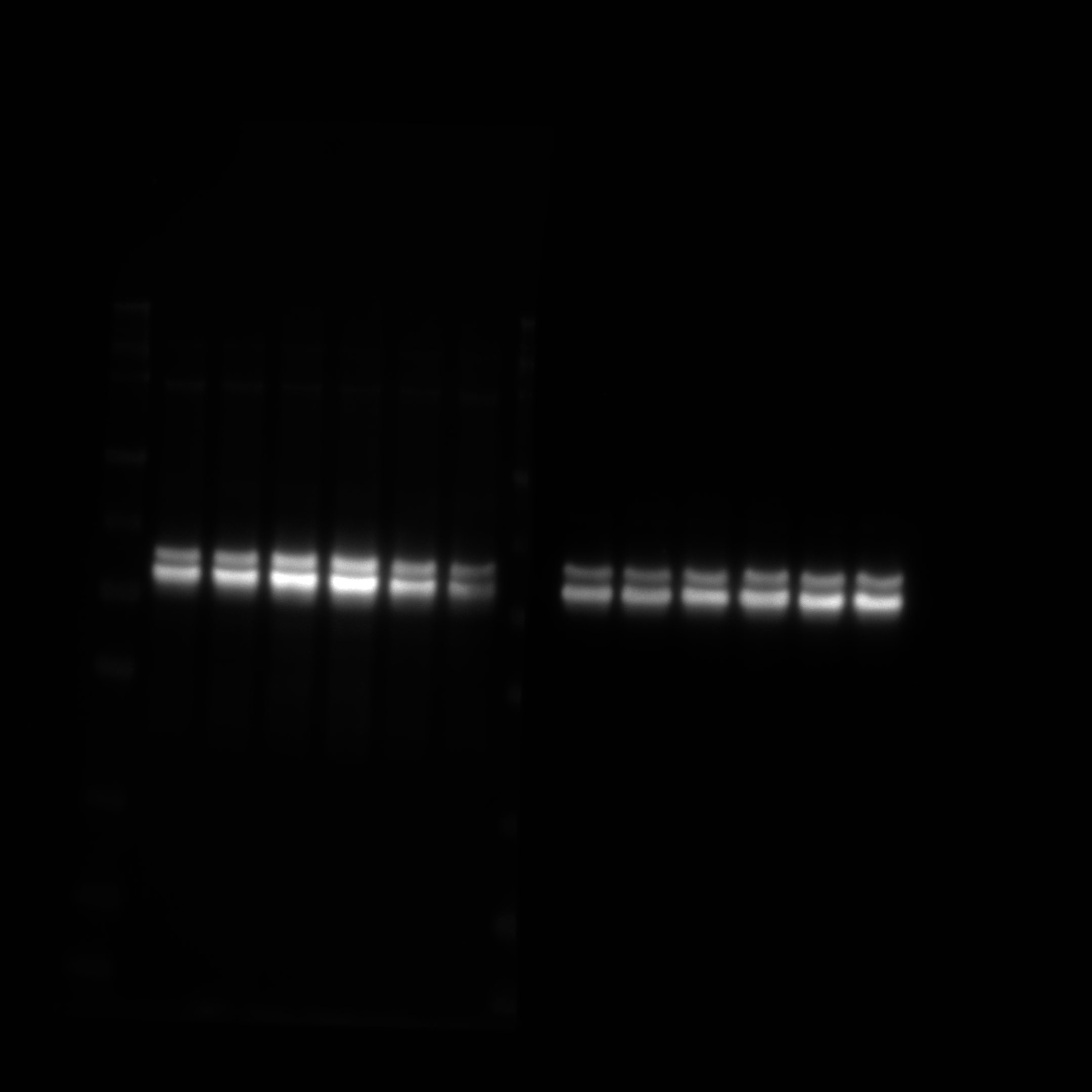

Supplement: Figure 4—source data 1. [file elife-106901-fig4-data1.zip › Figure4 source data 1/Figure 4C ERK.Tif]

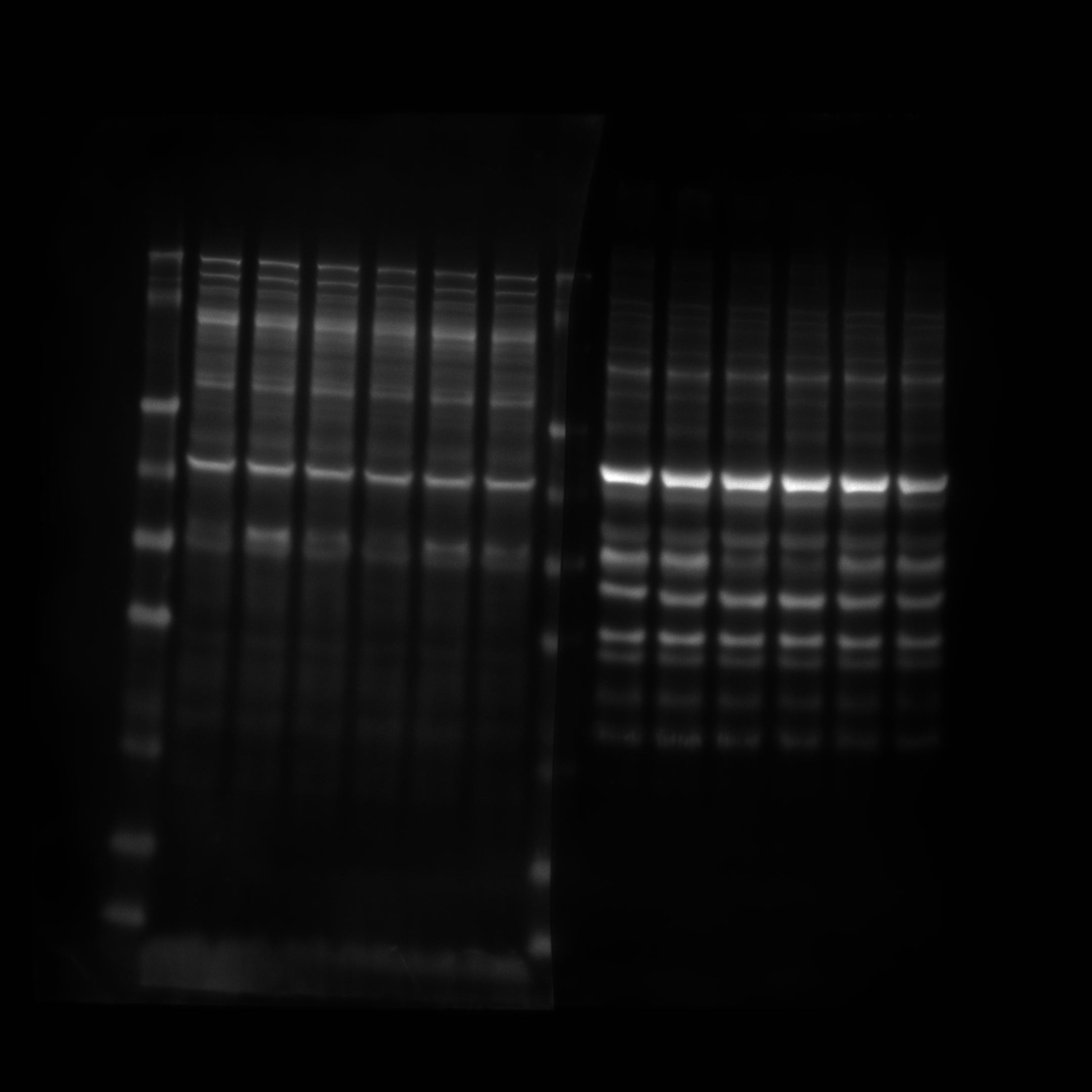

Supplement: Figure 4—source data 1. [file elife-106901-fig4-data1.zip › Figure4 source data 1/Figure 4C IkBa.Tif]

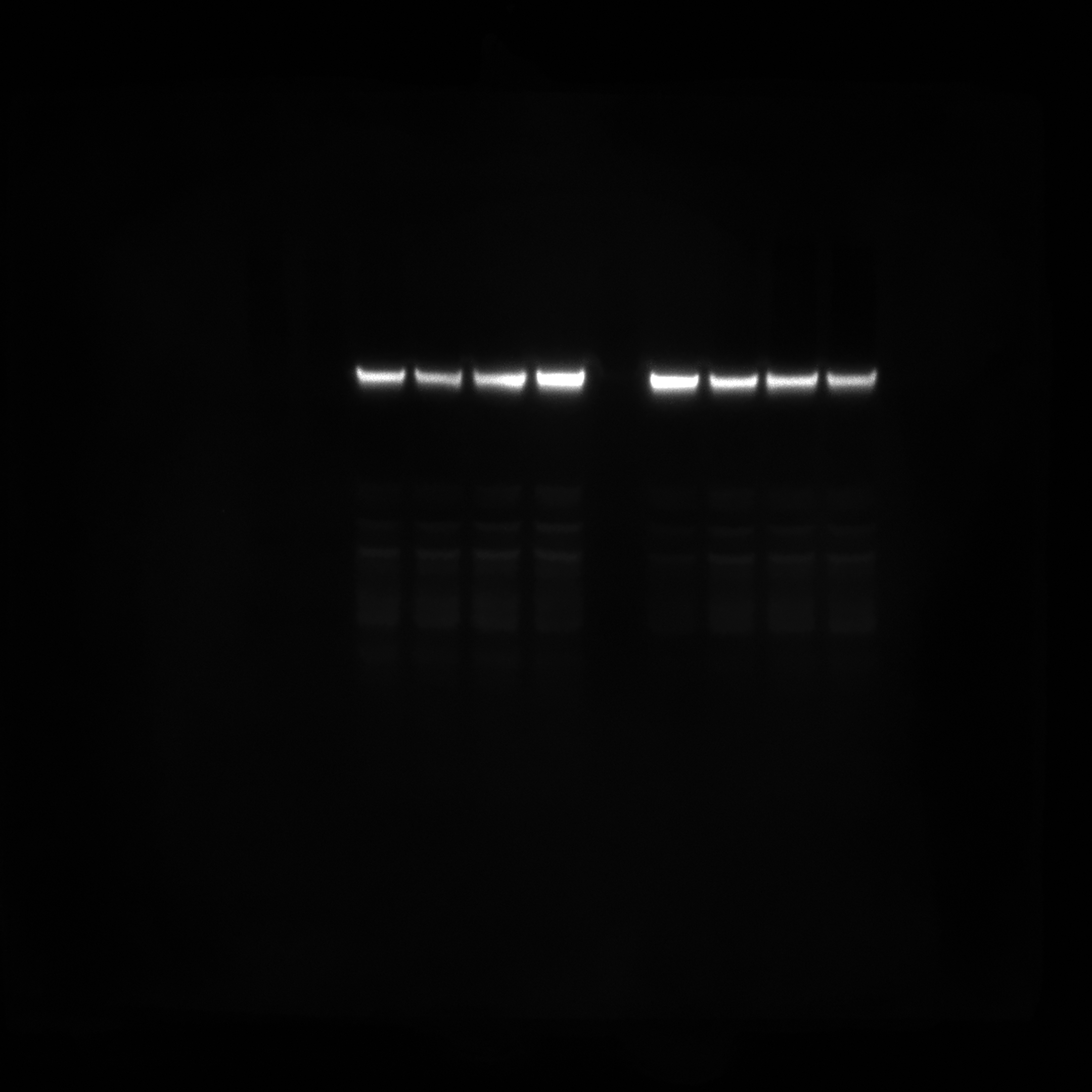

Supplement: Figure 4—source data 1. [file elife-106901-fig4-data1.zip › Figure4 source data 1/Figure 4C IKKa.Tif]

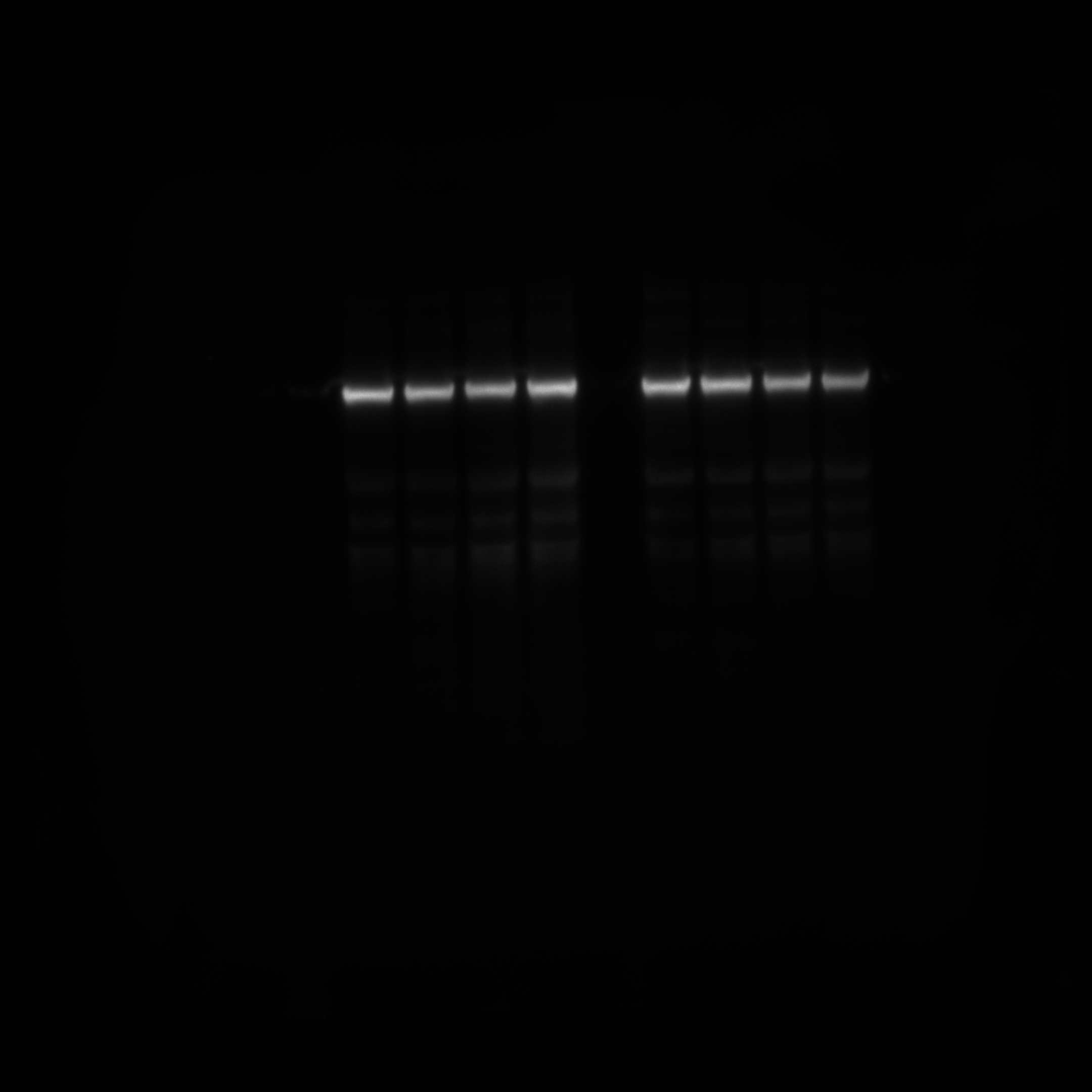

Supplement: Figure 4—source data 1. [file elife-106901-fig4-data1.zip › Figure4 source data 1/Figure 4C IKKb.Tif]

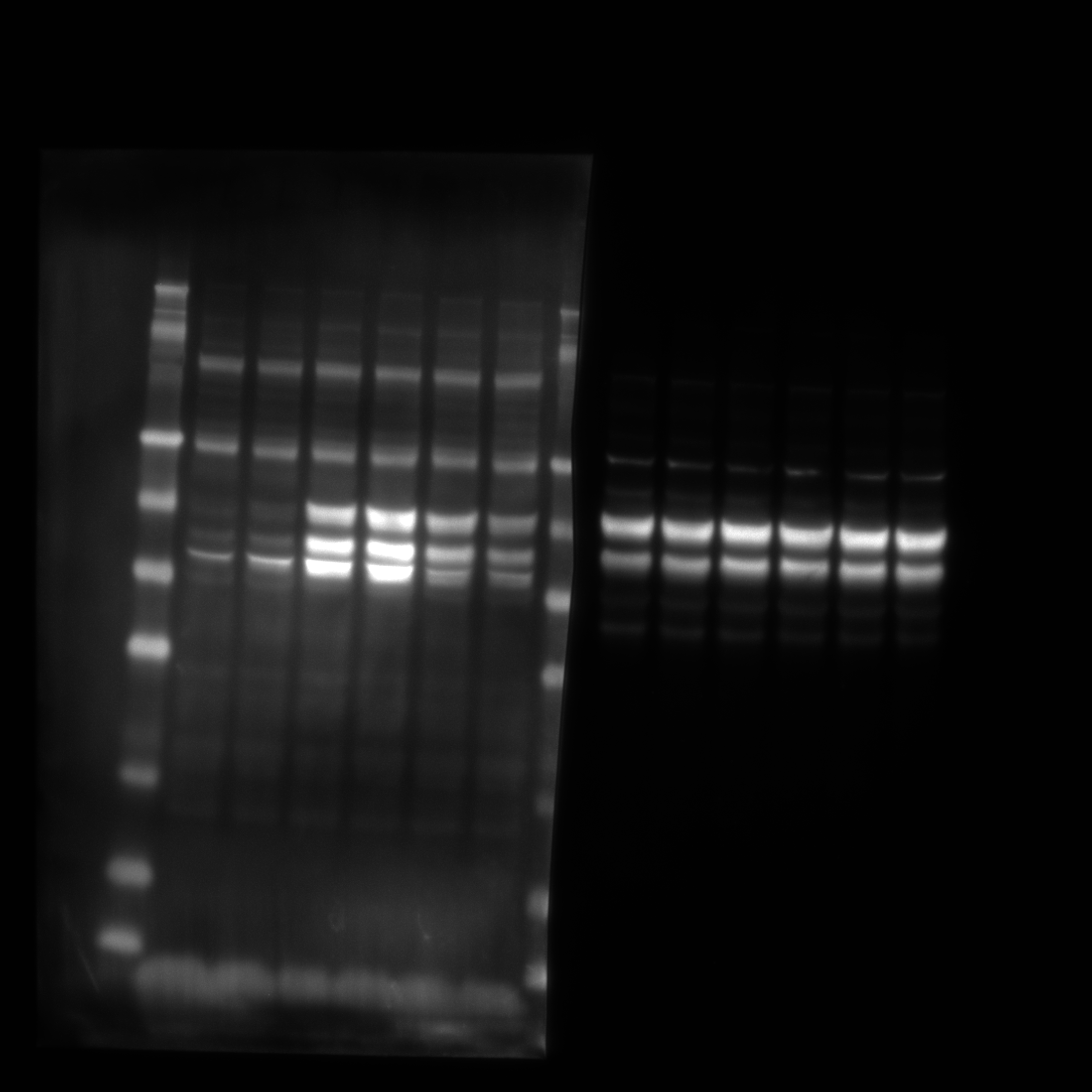

Supplement: Figure 4—source data 1. [file elife-106901-fig4-data1.zip › Figure4 source data 1/Figure 4C JNK.Tif]

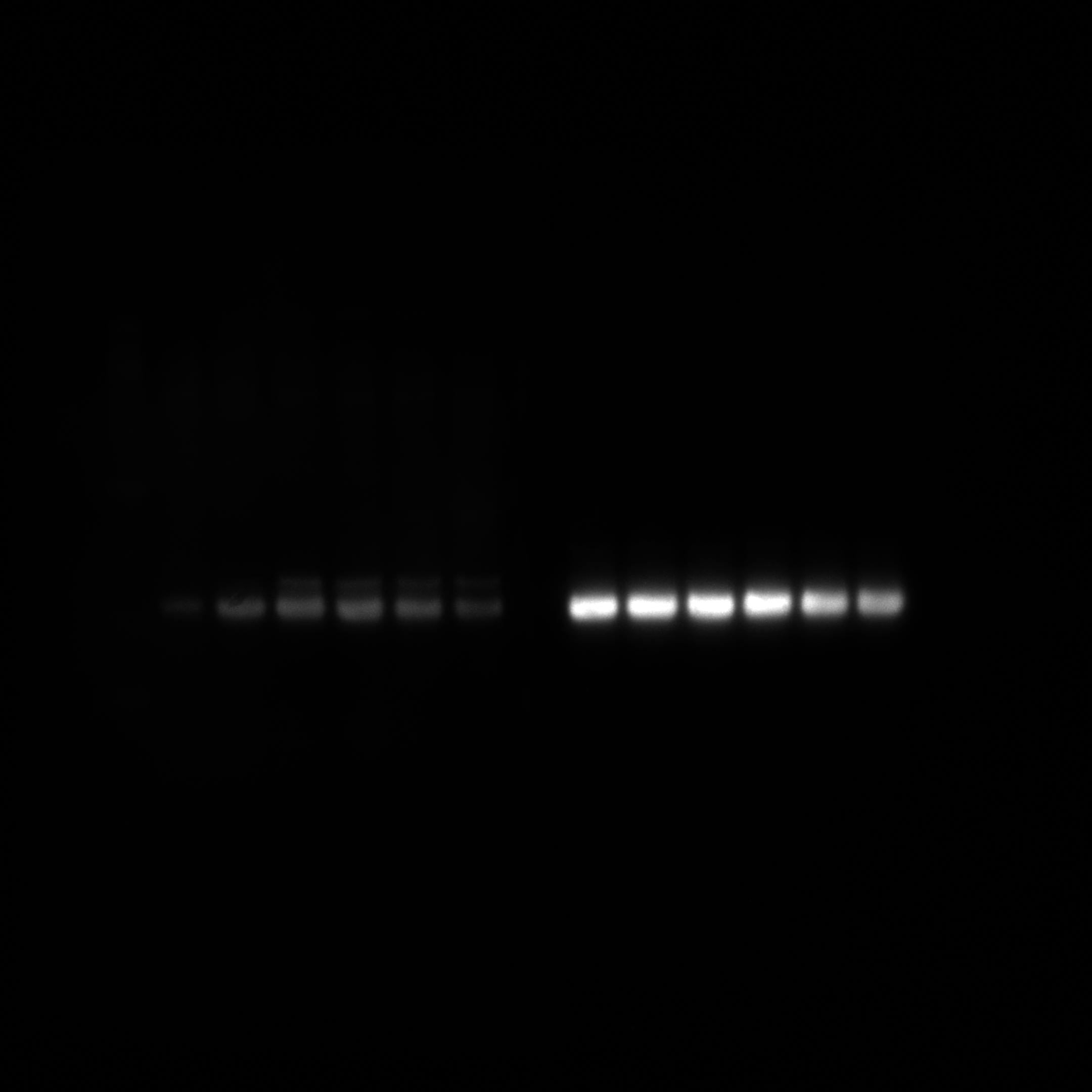

Supplement: Figure 4—source data 1. [file elife-106901-fig4-data1.zip › Figure4 source data 1/Figure 4C p38.Tif]

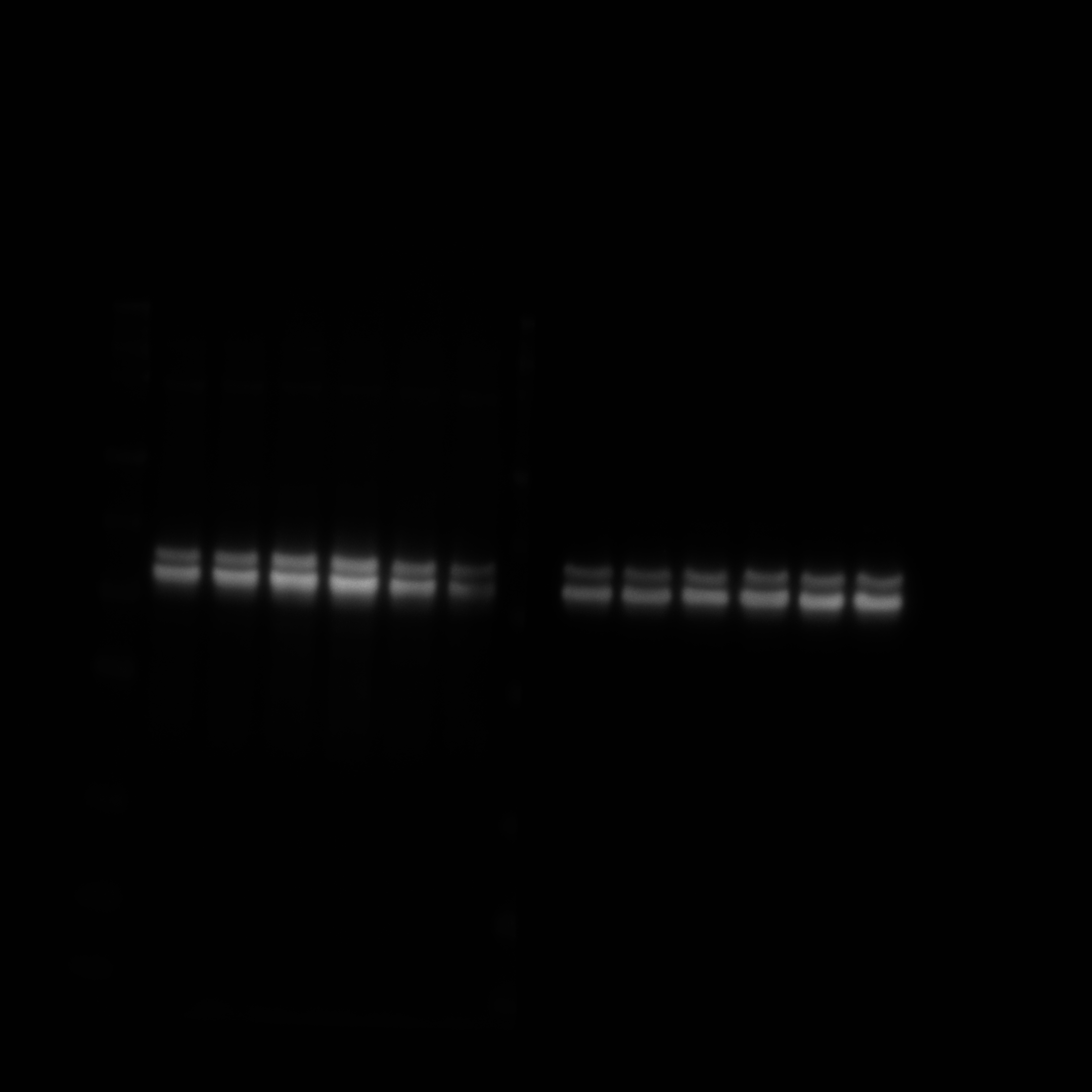

Supplement: Figure 4—source data 1. [file elife-106901-fig4-data1.zip › Figure4 source data 1/Figure 4C pERK.Tif]

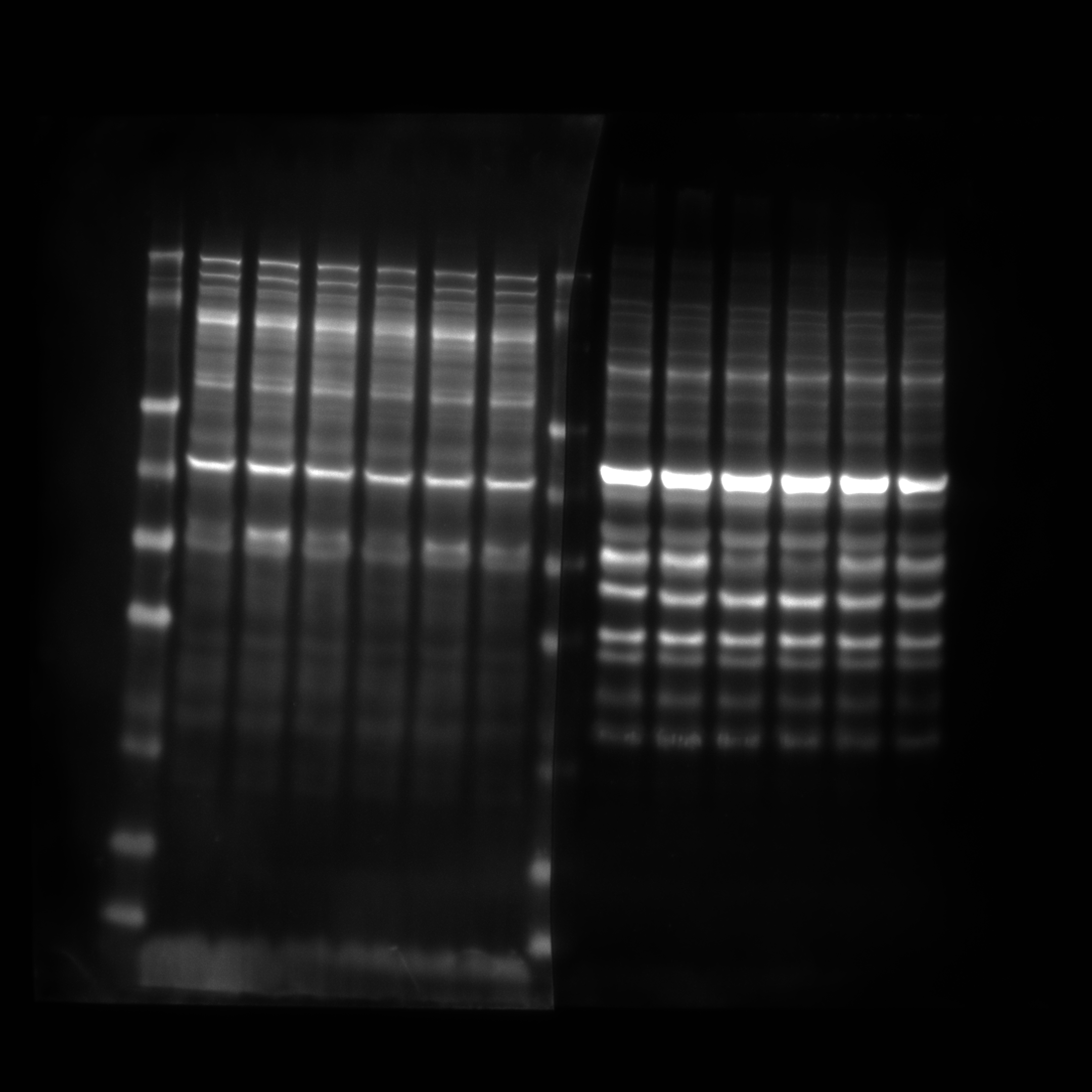

Supplement: Figure 4—source data 1. [file elife-106901-fig4-data1.zip › Figure4 source data 1/Figure 4C pIkBa .Tif]

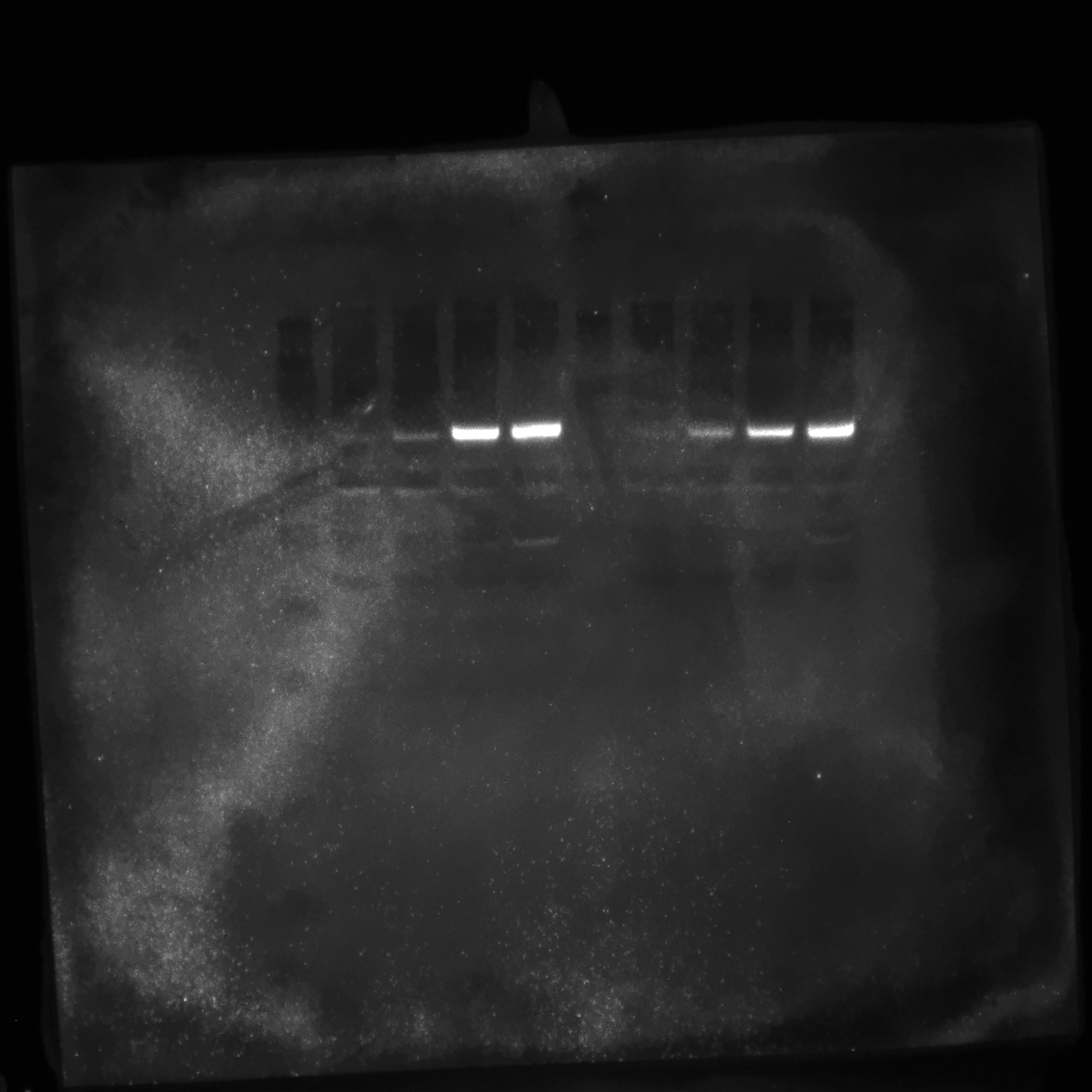

Supplement: Figure 4—source data 1. [file elife-106901-fig4-data1.zip › Figure4 source data 1/Figure 4C pIKKab.Tif]

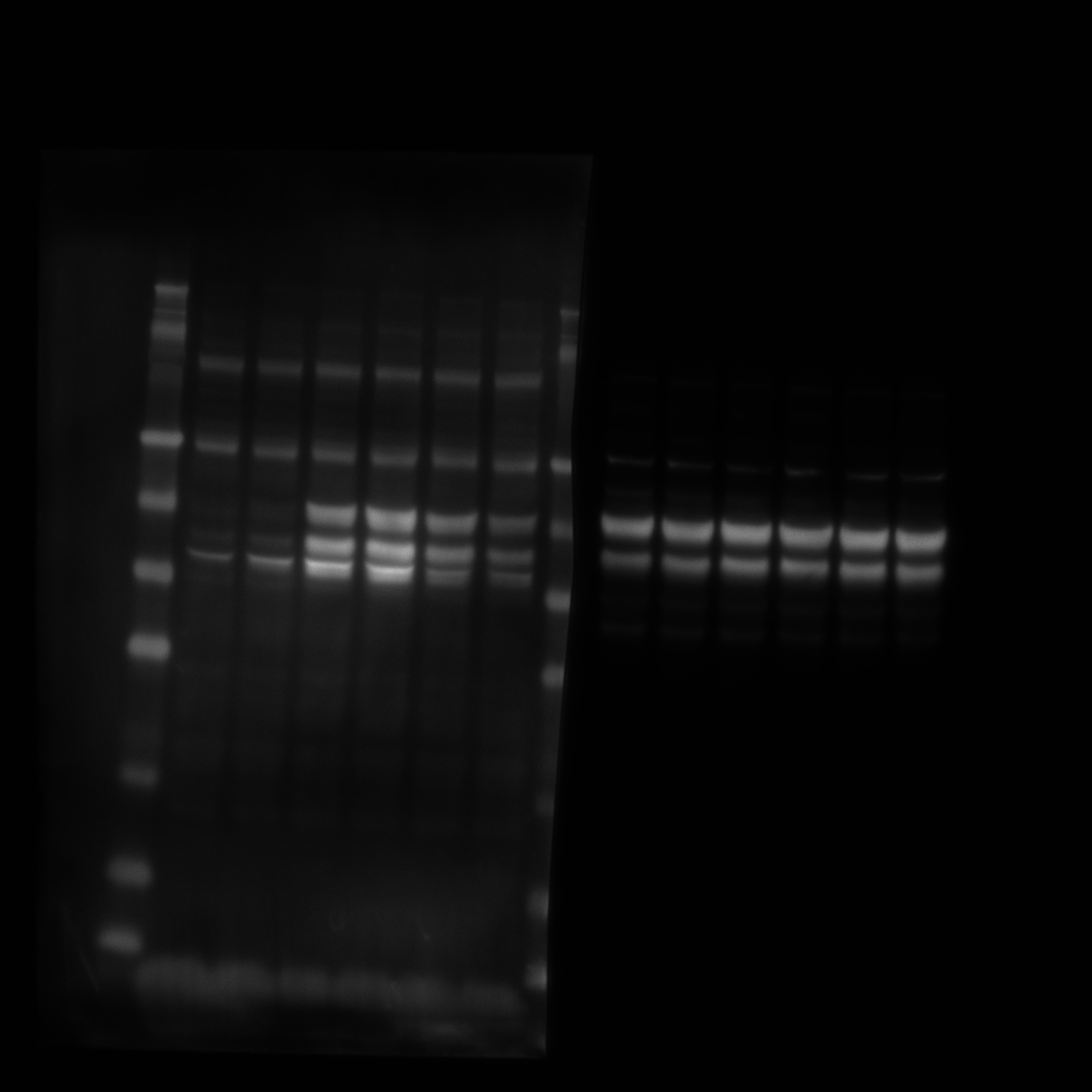

Supplement: Figure 4—source data 1. [file elife-106901-fig4-data1.zip › Figure4 source data 1/Figure 4C pJNK.Tif]

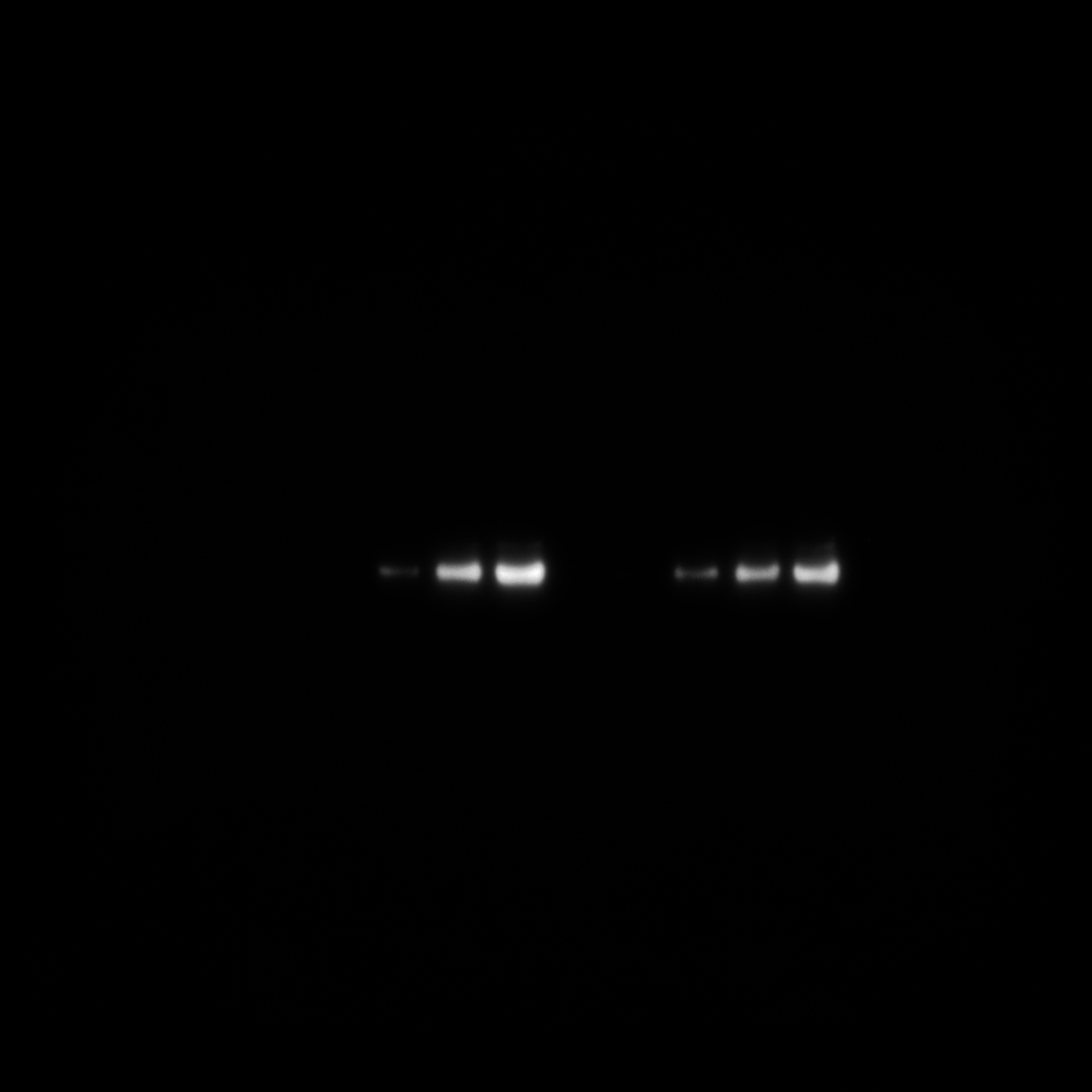

Supplement: Figure 4—source data 1. [file elife-106901-fig4-data1.zip › Figure4 source data 1/Figure 4C pp38.Tif]

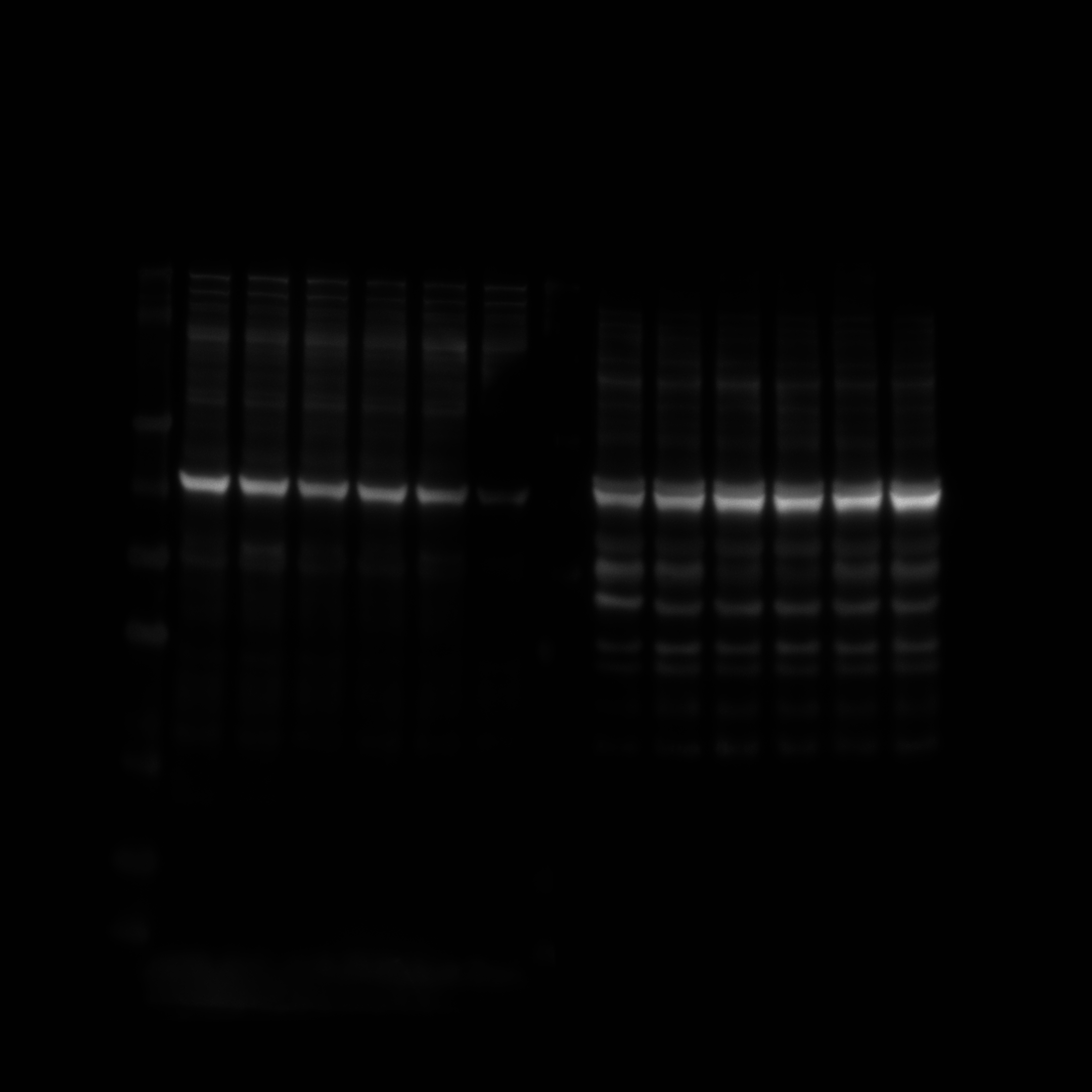

Supplement: Figure 4—source data 1. [file elife-106901-fig4-data1.zip › Figure4 source data 1/Figure 4C Tubulin.Tif]

**Figure 4C**

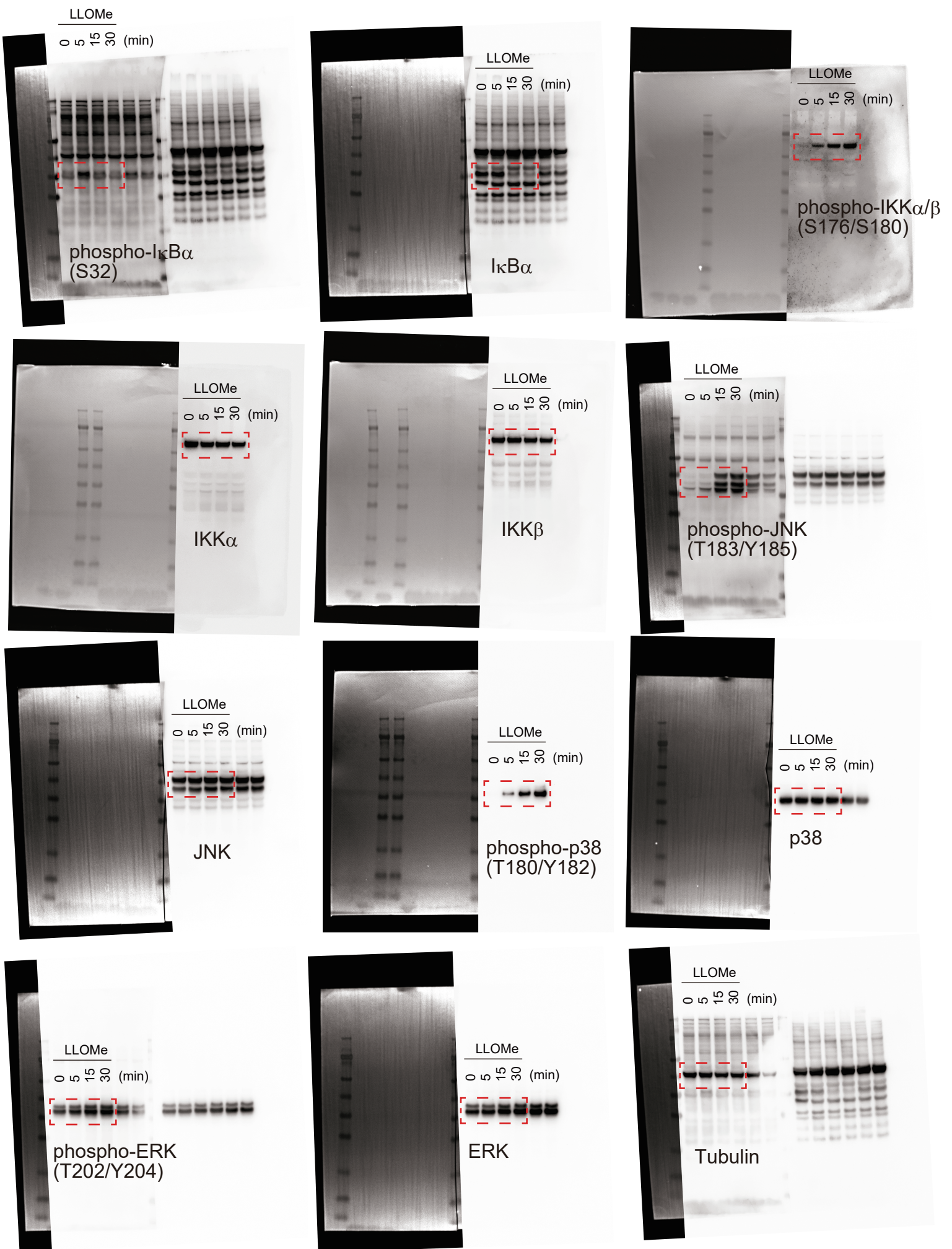

Supplement: Figure 4—source data 2. [file elife-106901-fig4-data2.pdf]

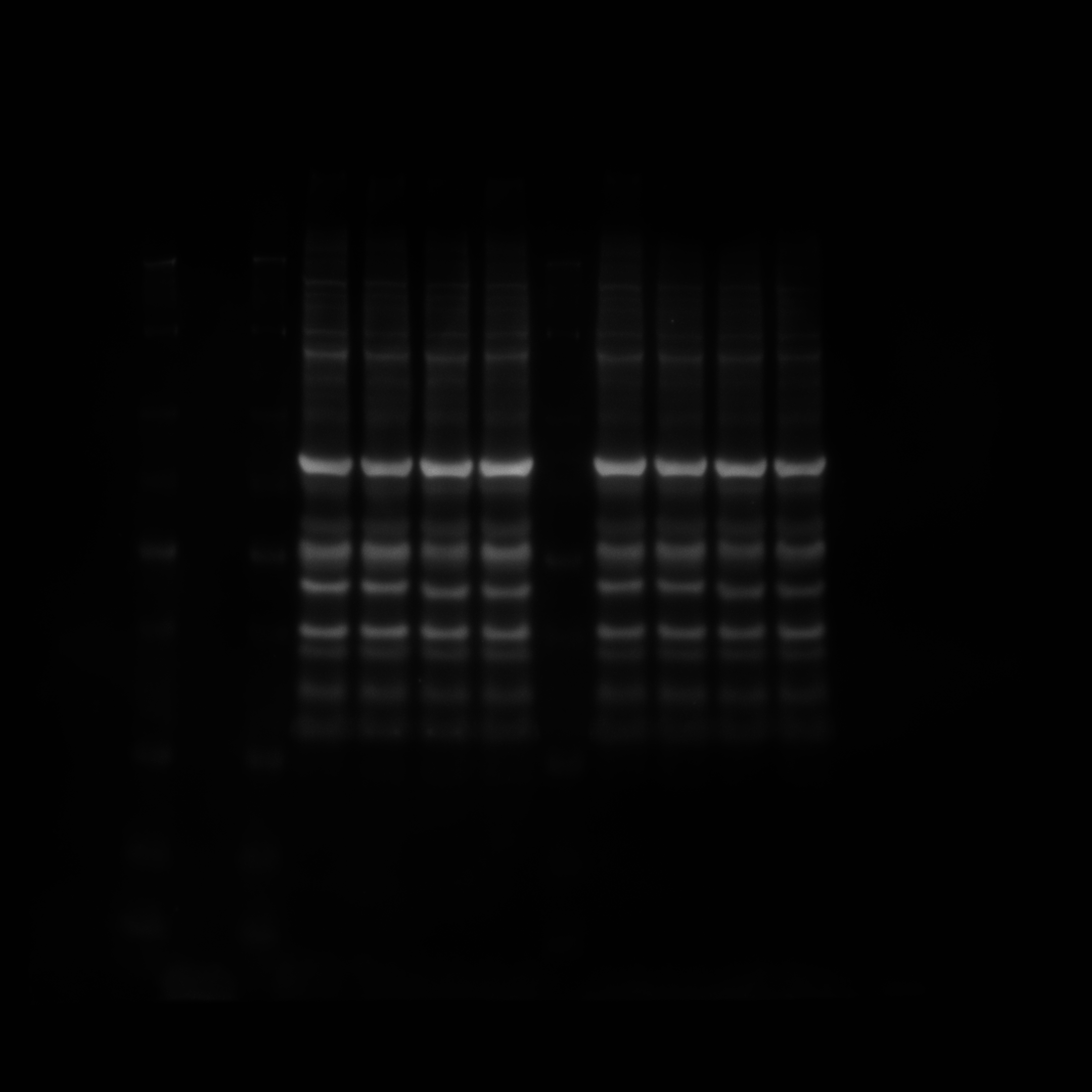

Supplement: Figure 4—source data 3. [file elife-106901-fig4-data3.zip › Figure4 source data 3/Figure 4D IkBa.Tif]

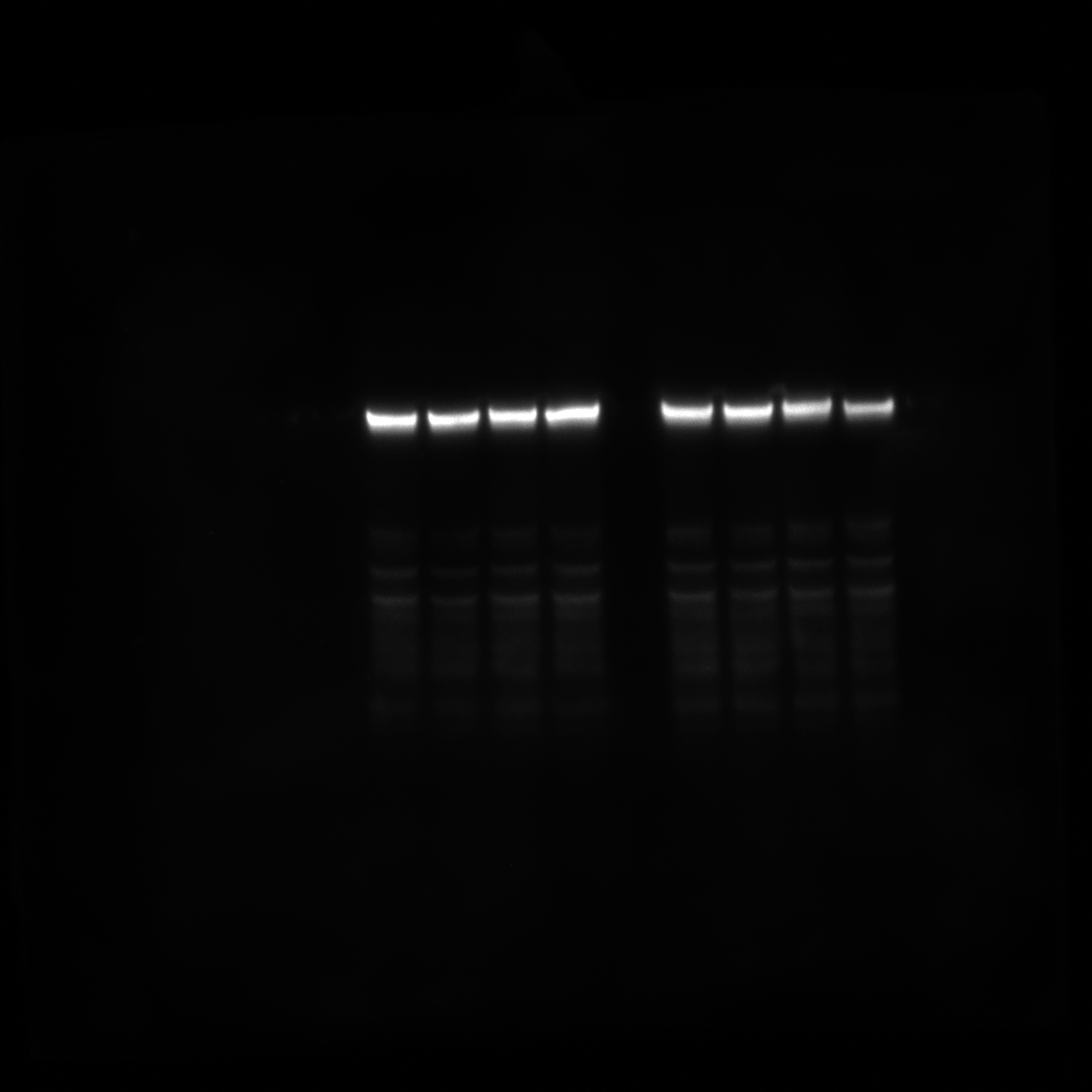

Supplement: Figure 4—source data 3. [file elife-106901-fig4-data3.zip › Figure4 source data 3/Figure 4D IKKa.Tif]

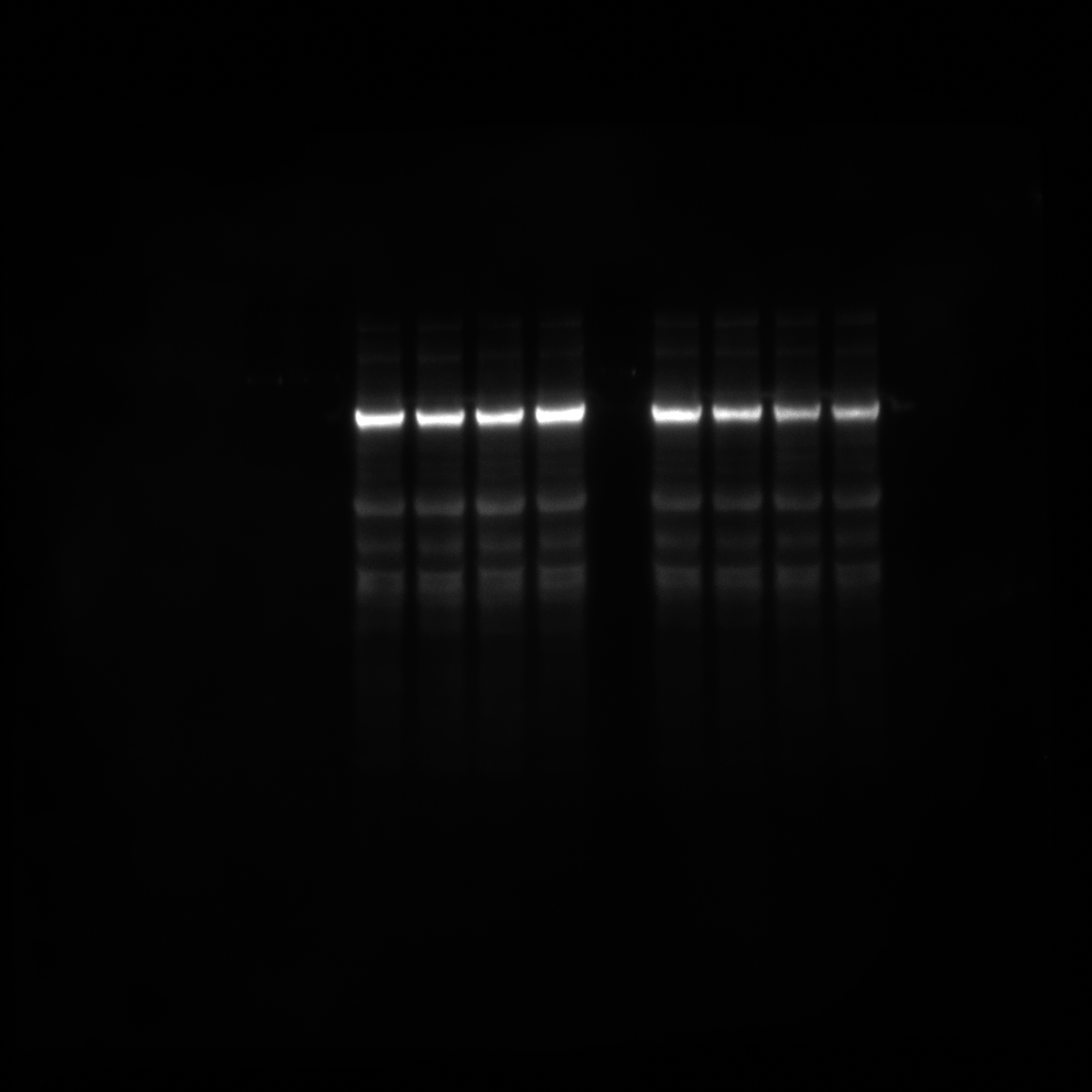

Supplement: Figure 4—source data 3. [file elife-106901-fig4-data3.zip › Figure4 source data 3/Figure 4D IKKb.Tif]

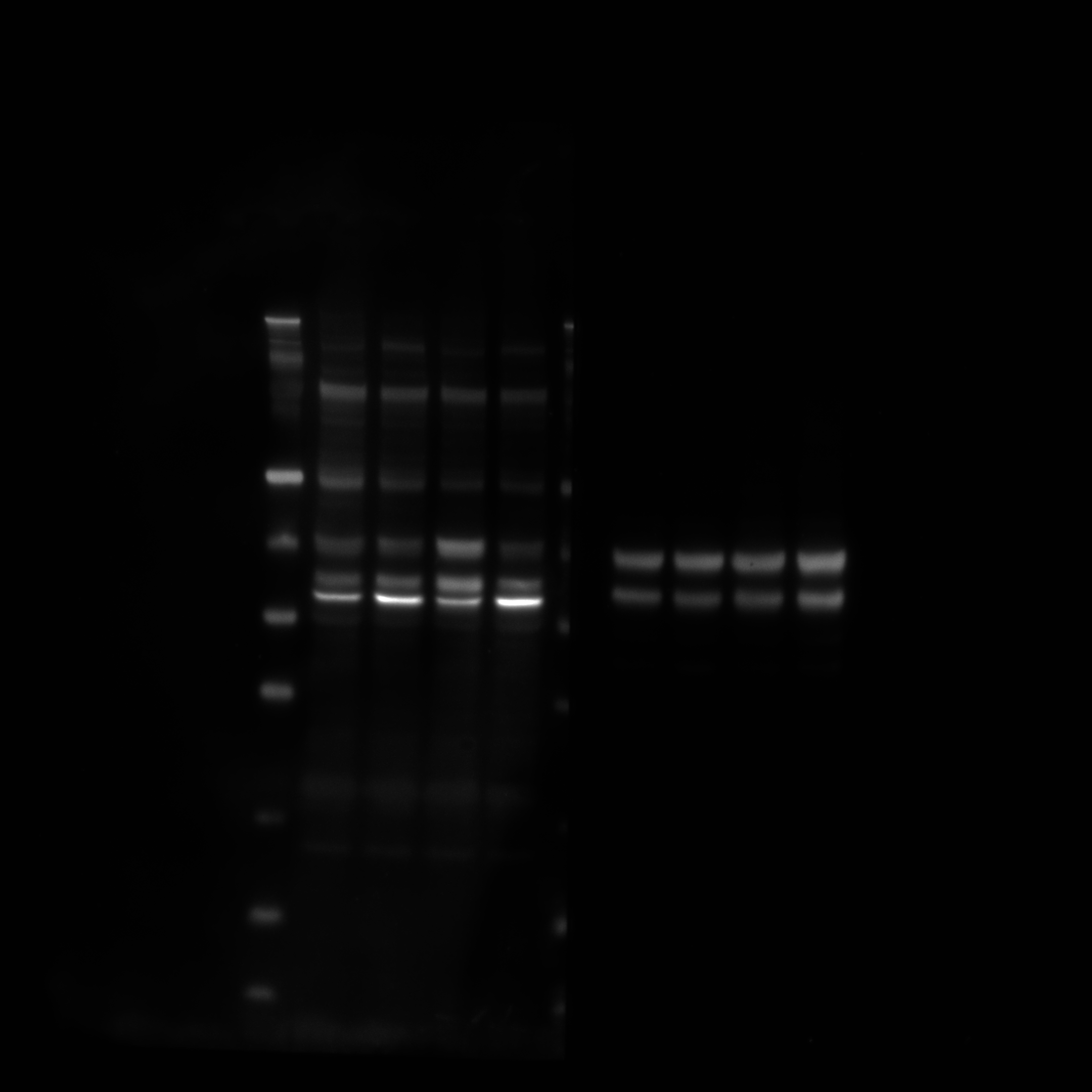

Supplement: Figure 4—source data 3. [file elife-106901-fig4-data3.zip › Figure4 source data 3/Figure 4D JNK.Tif]

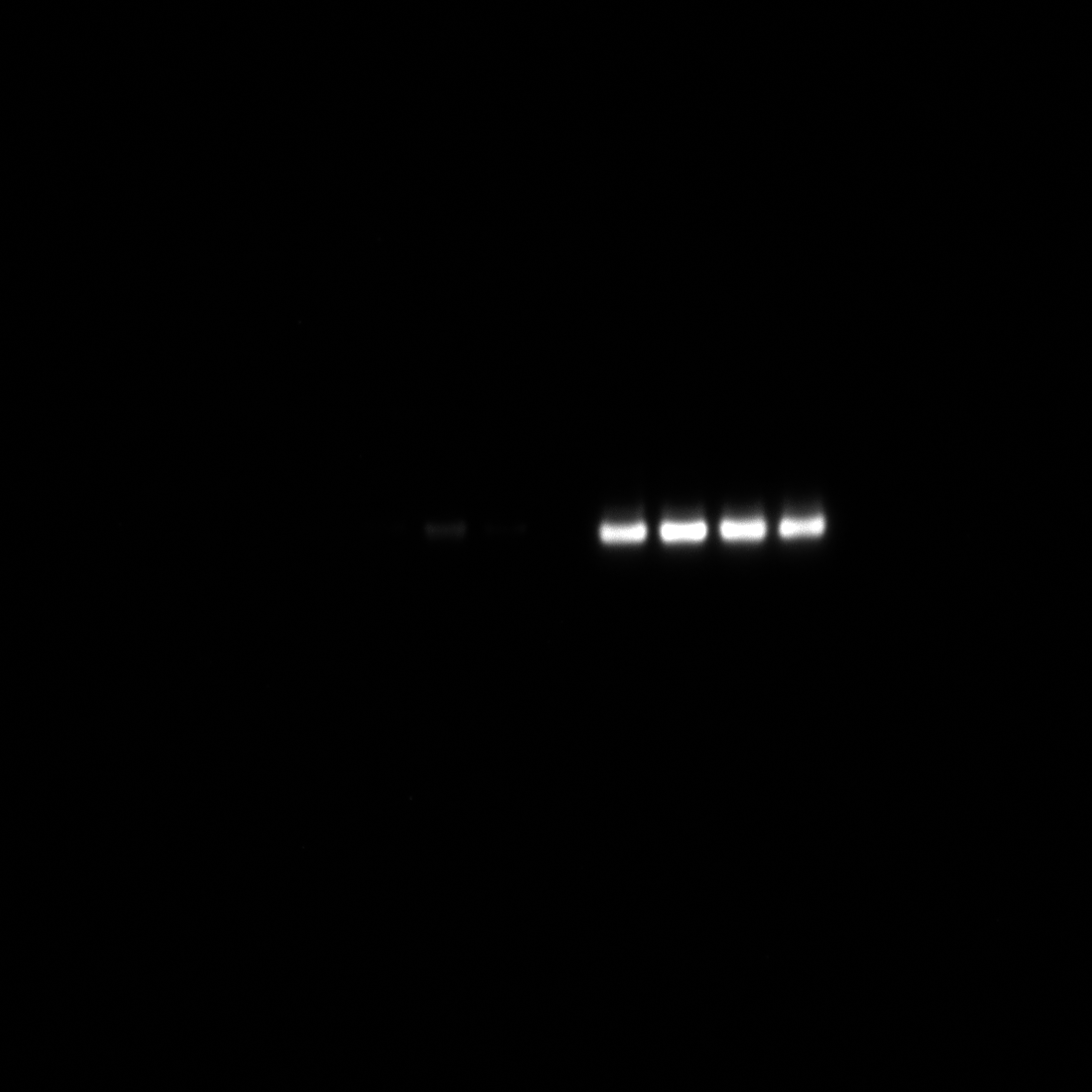

Supplement: Figure 4—source data 3. [file elife-106901-fig4-data3.zip › Figure4 source data 3/Figure 4D p38.Tif]

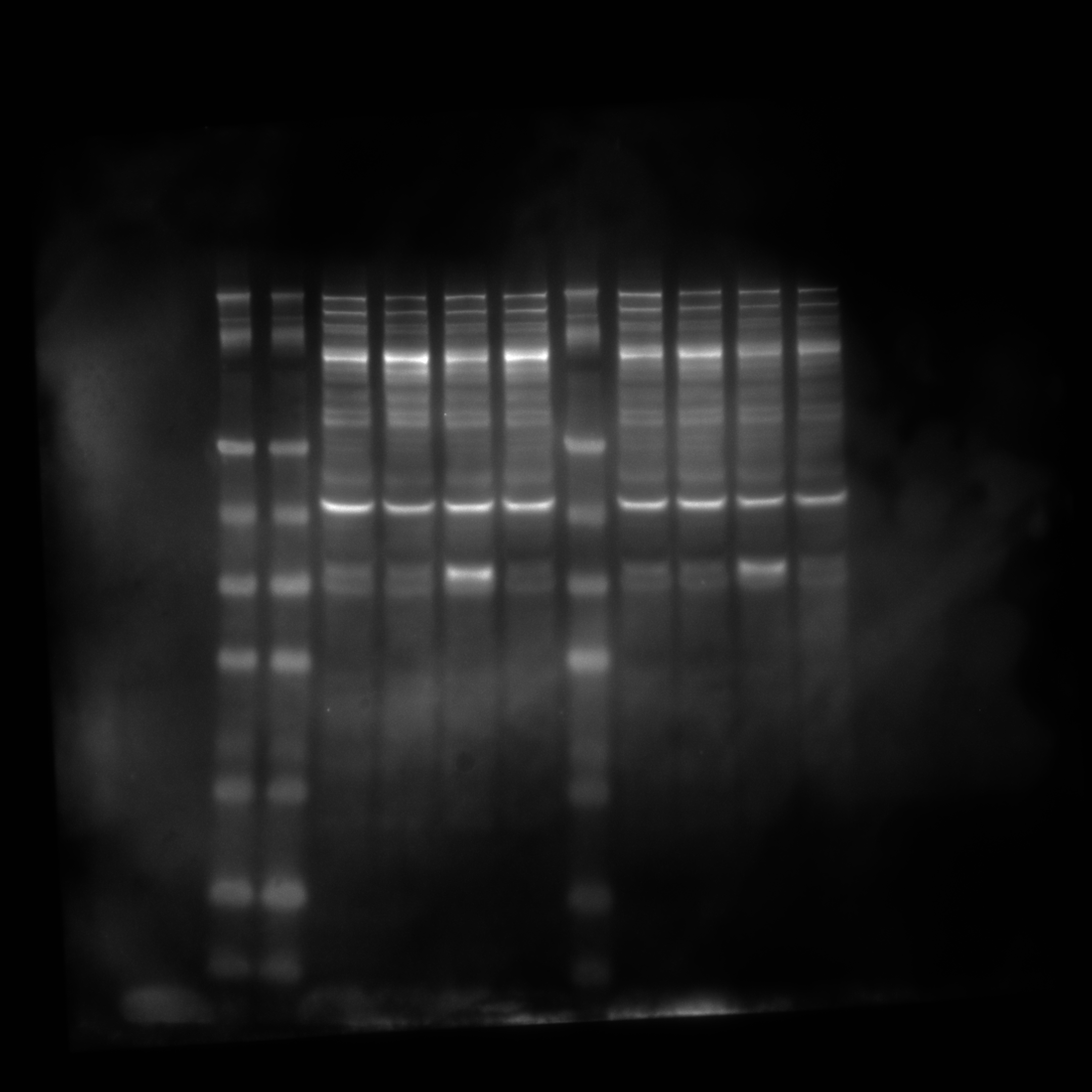

Supplement: Figure 4—source data 3. [file elife-106901-fig4-data3.zip › Figure4 source data 3/Figure 4D pIkBa.Tif]

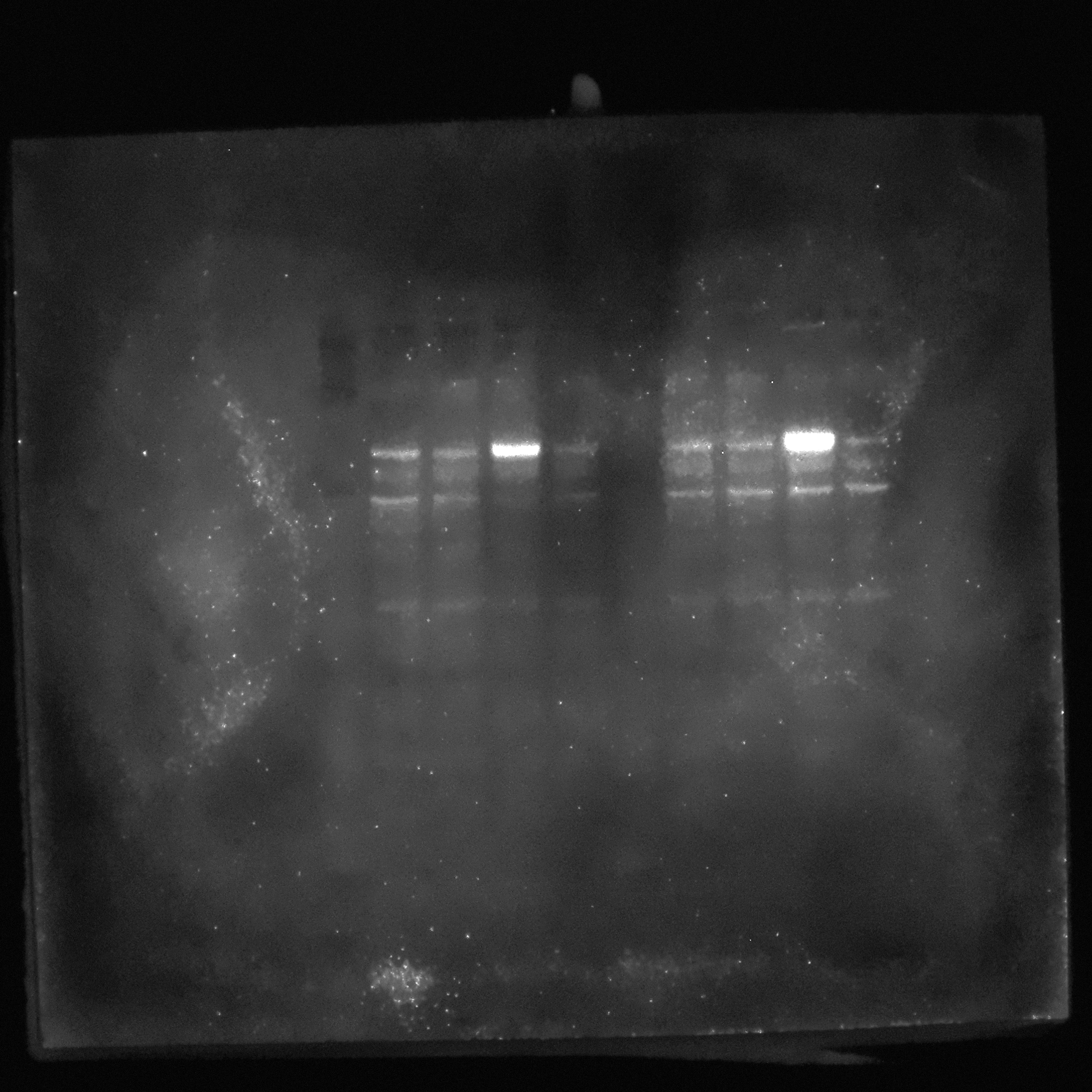

Supplement: Figure 4—source data 3. [file elife-106901-fig4-data3.zip › Figure4 source data 3/Figure 4D pIKKab.Tif]

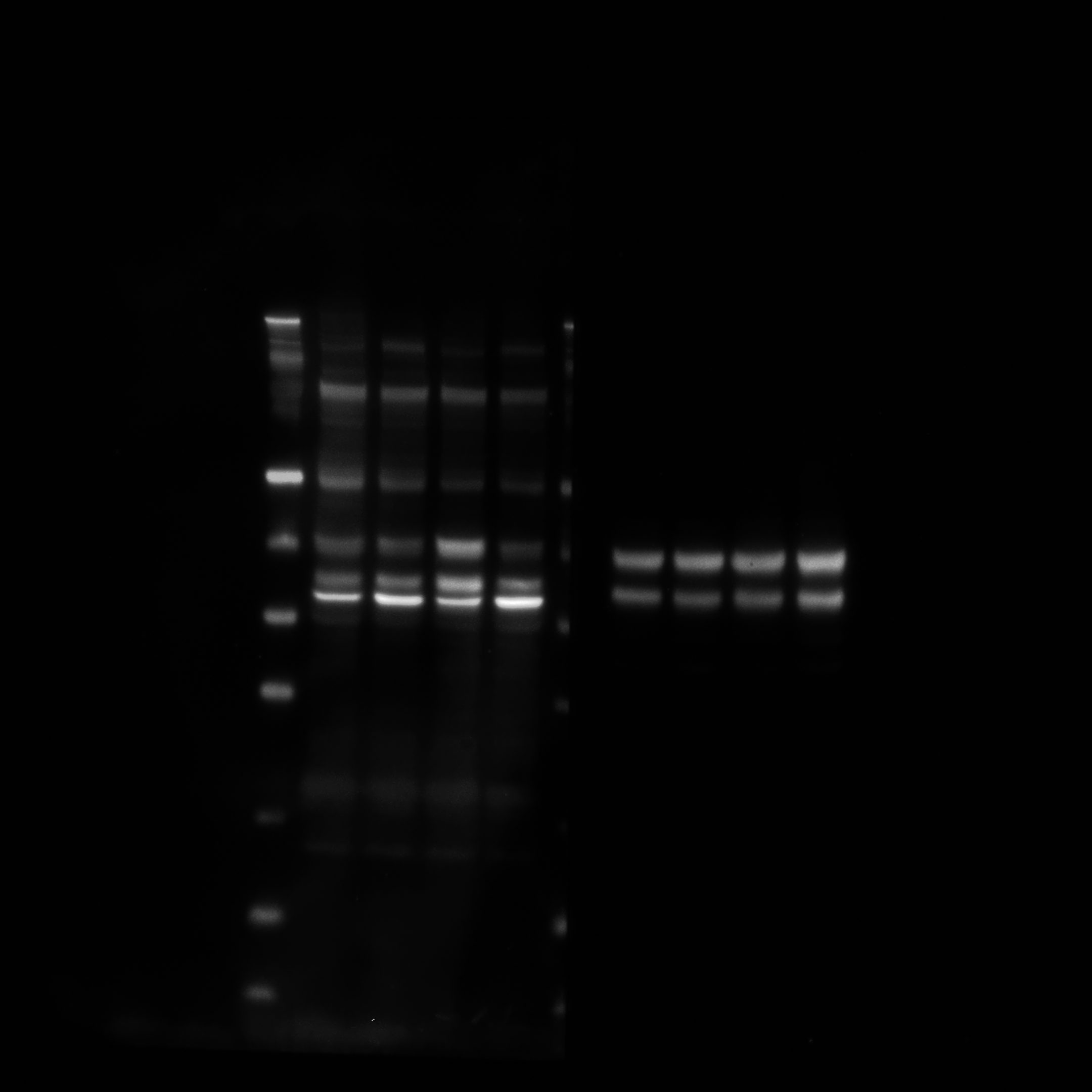

Supplement: Figure 4—source data 3. [file elife-106901-fig4-data3.zip › Figure4 source data 3/Figure 4D pJNK.Tif]

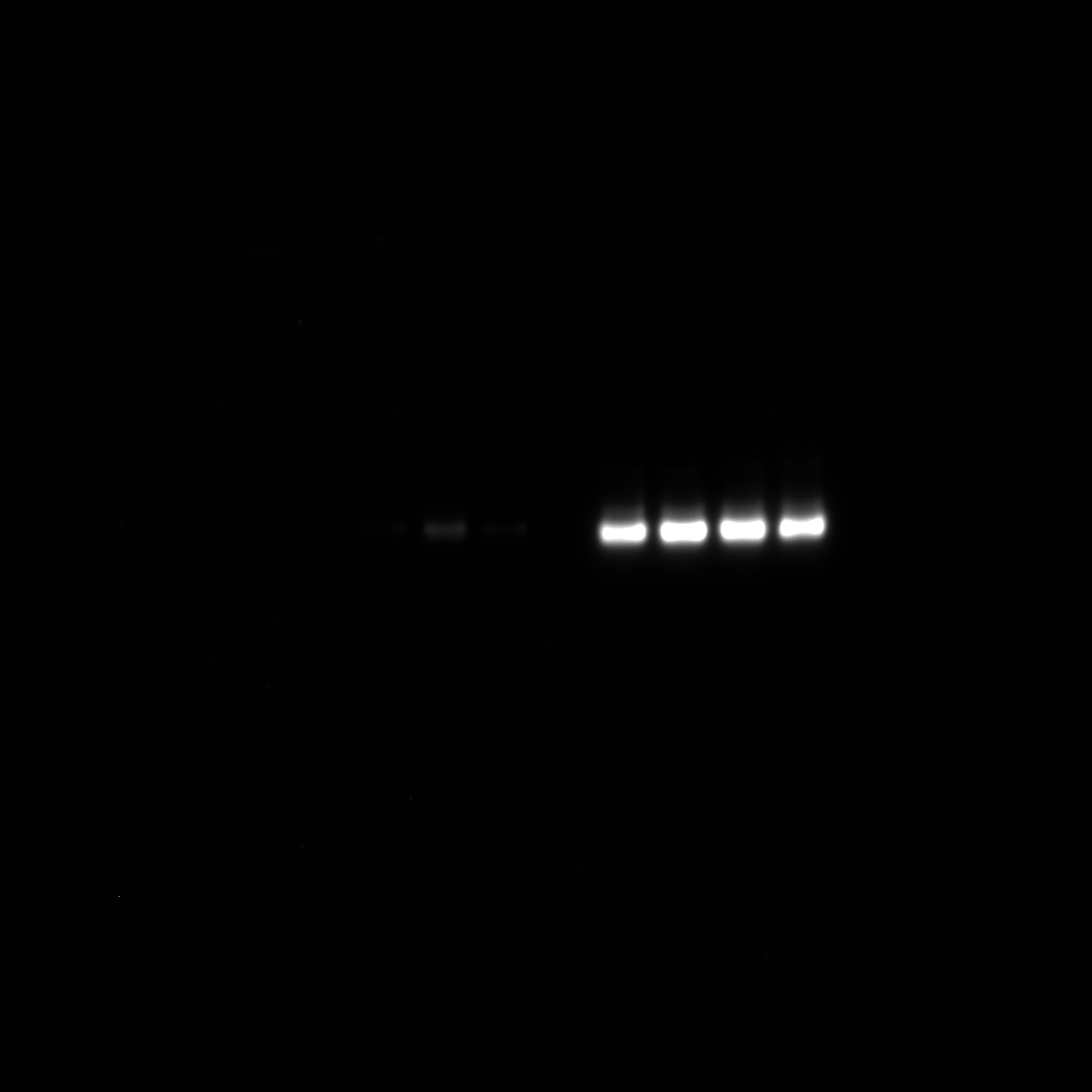

Supplement: Figure 4—source data 3. [file elife-106901-fig4-data3.zip › Figure4 source data 3/Figure 4D pp38.Tif]

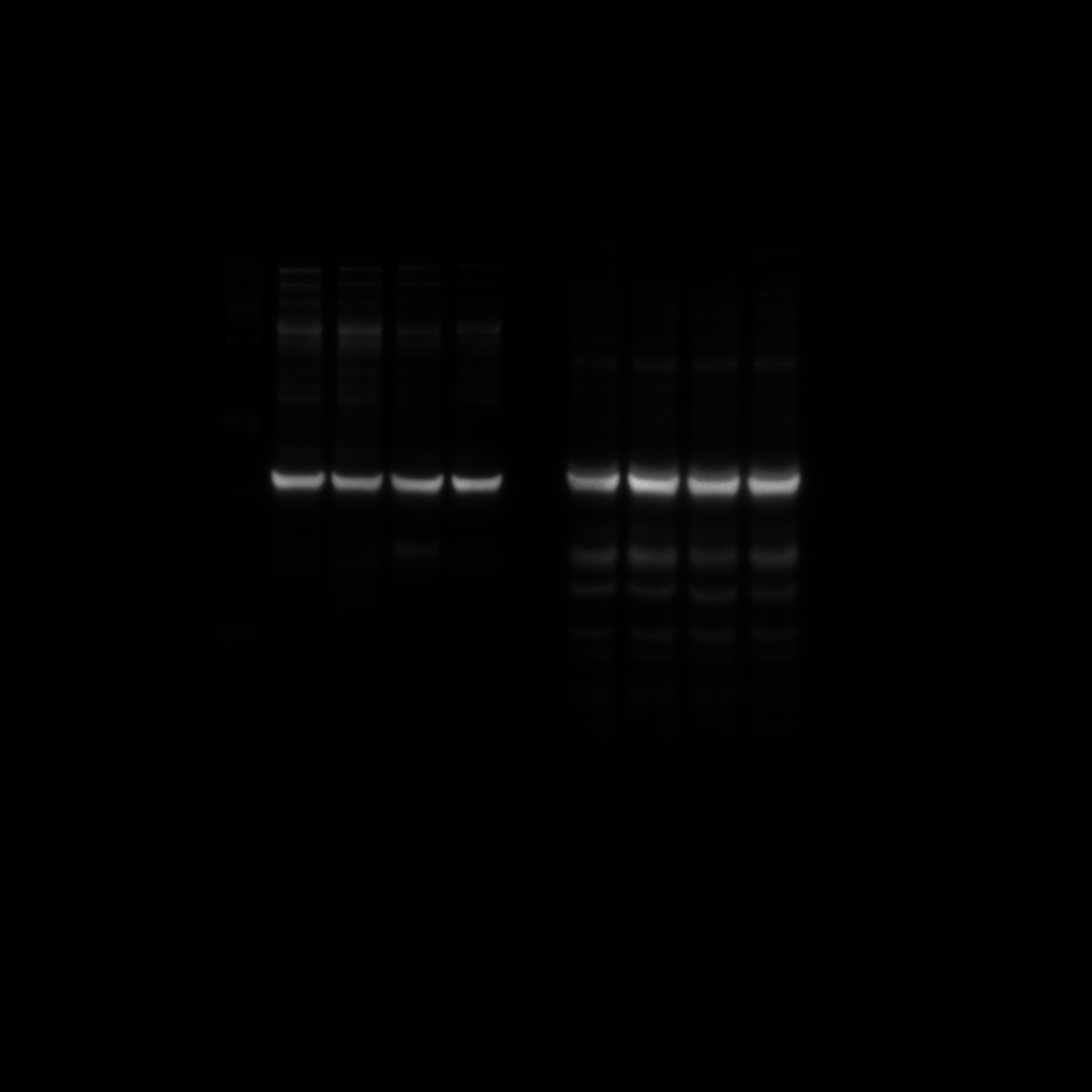

Supplement: Figure 4—source data 3. [file elife-106901-fig4-data3.zip › Figure4 source data 3/Figure 4D Tubulin.Tif]

**Figure 4D**

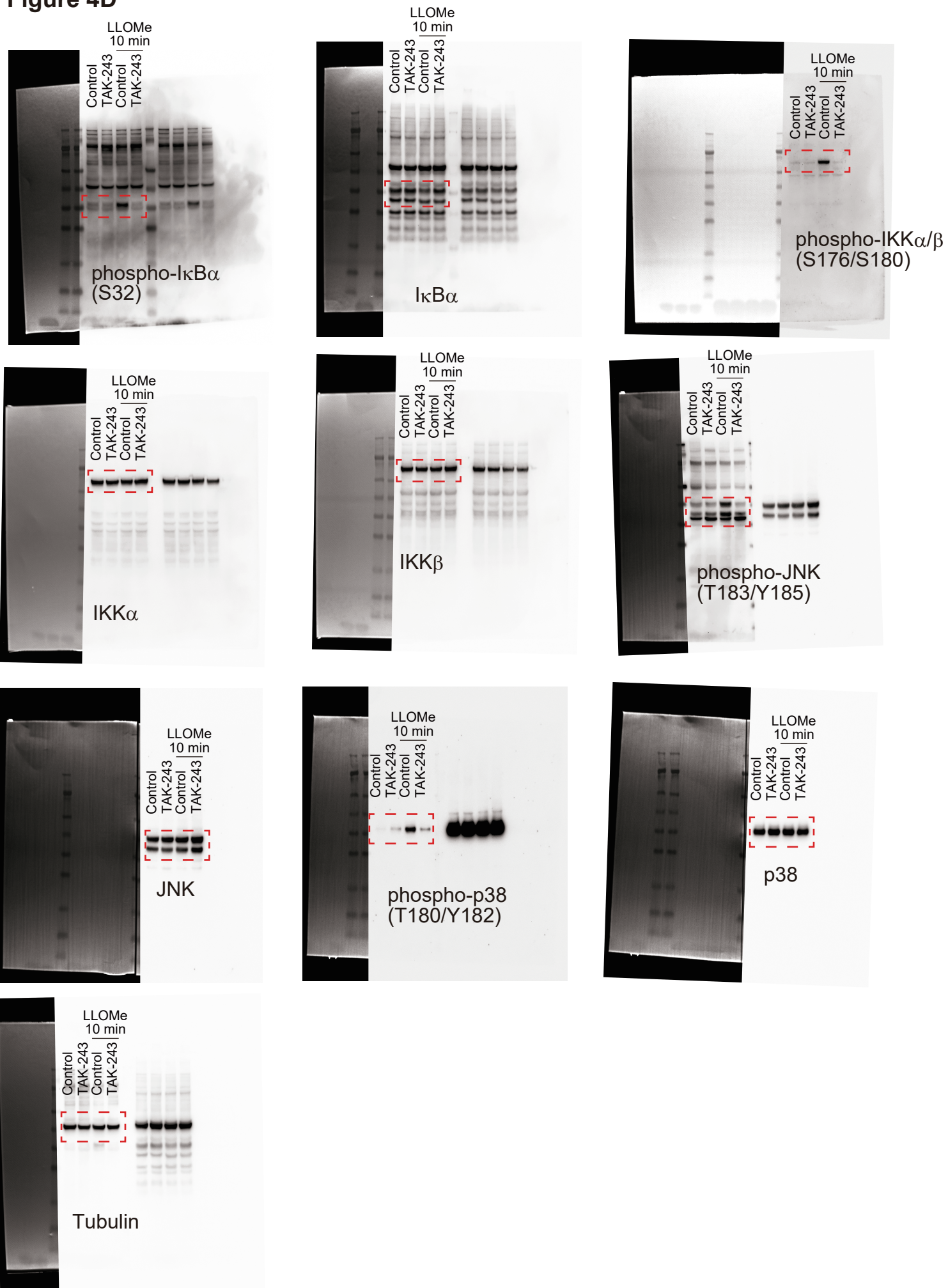

Supplement: Figure 4—source data 4. [file elife-106901-fig4-data4.pdf]

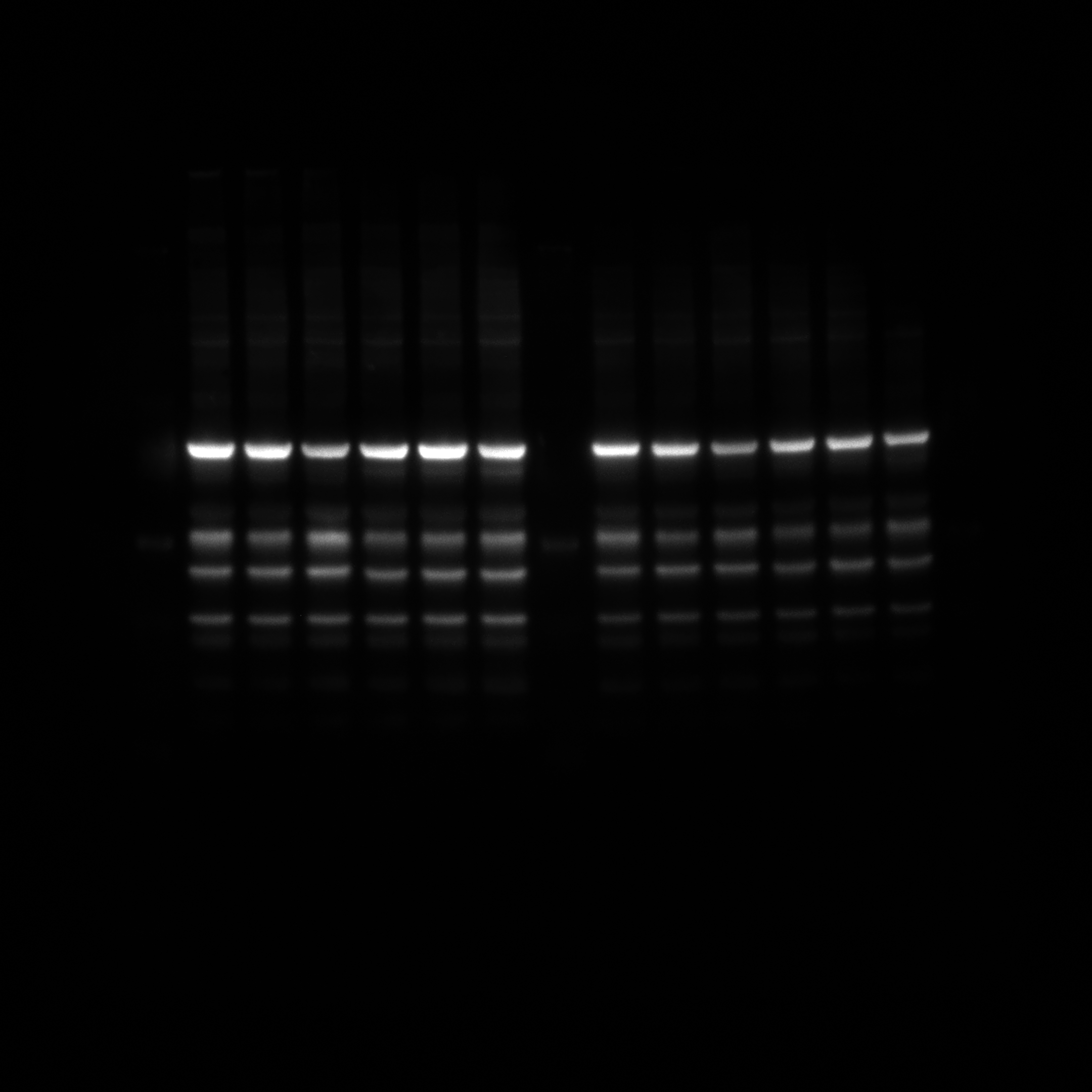

Supplement: Figure 4—source data 5. [file elife-106901-fig4-data5.zip › Figure4 source data 5/Figure 4E IkBa.Tif]

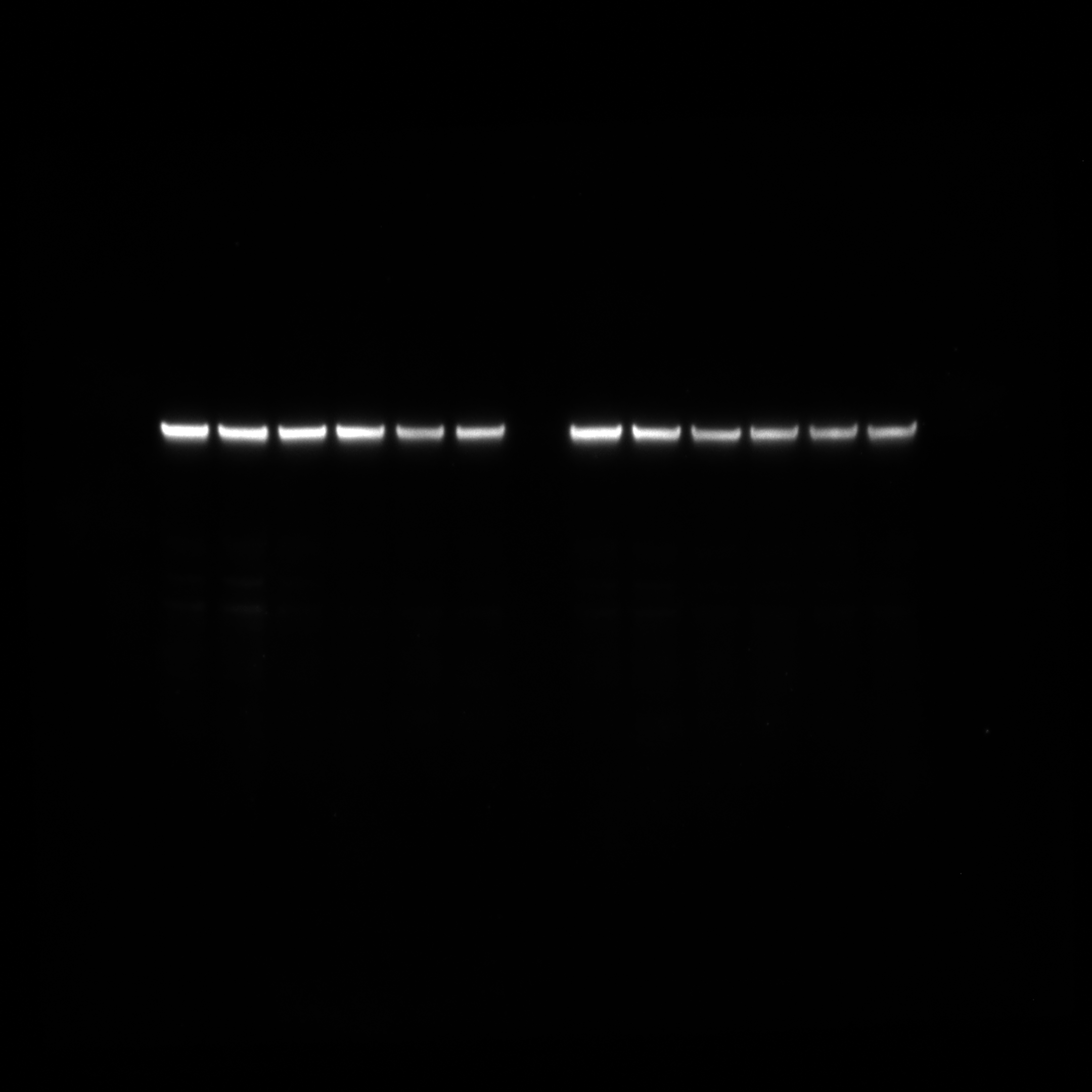

Supplement: Figure 4—source data 5. [file elife-106901-fig4-data5.zip › Figure4 source data 5/Figure 4E IKKa.Tif]

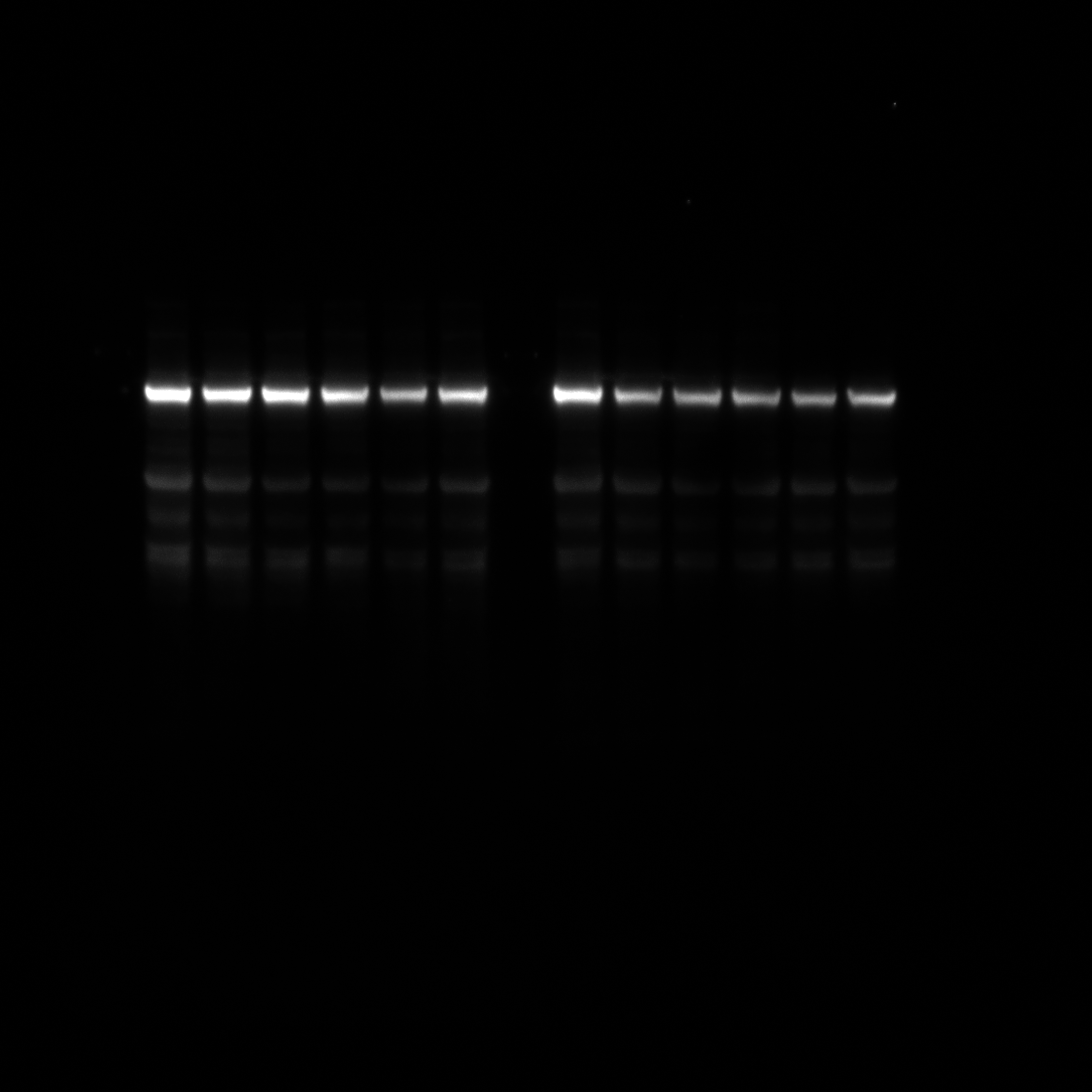

Supplement: Figure 4—source data 5. [file elife-106901-fig4-data5.zip › Figure4 source data 5/Figure 4E IKKb.Tif]

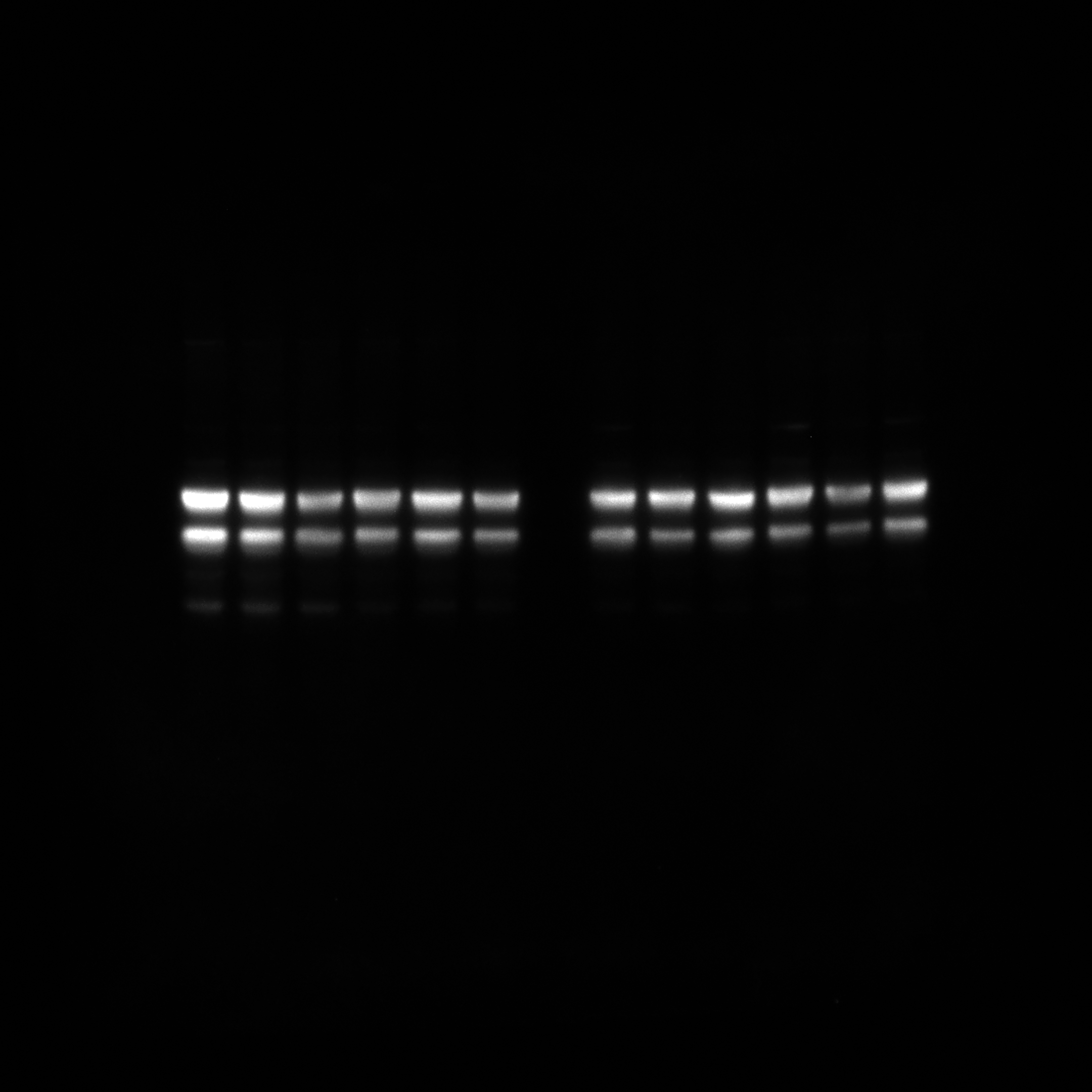

Supplement: Figure 4—source data 5. [file elife-106901-fig4-data5.zip › Figure4 source data 5/Figure 4E JNK.Tif]

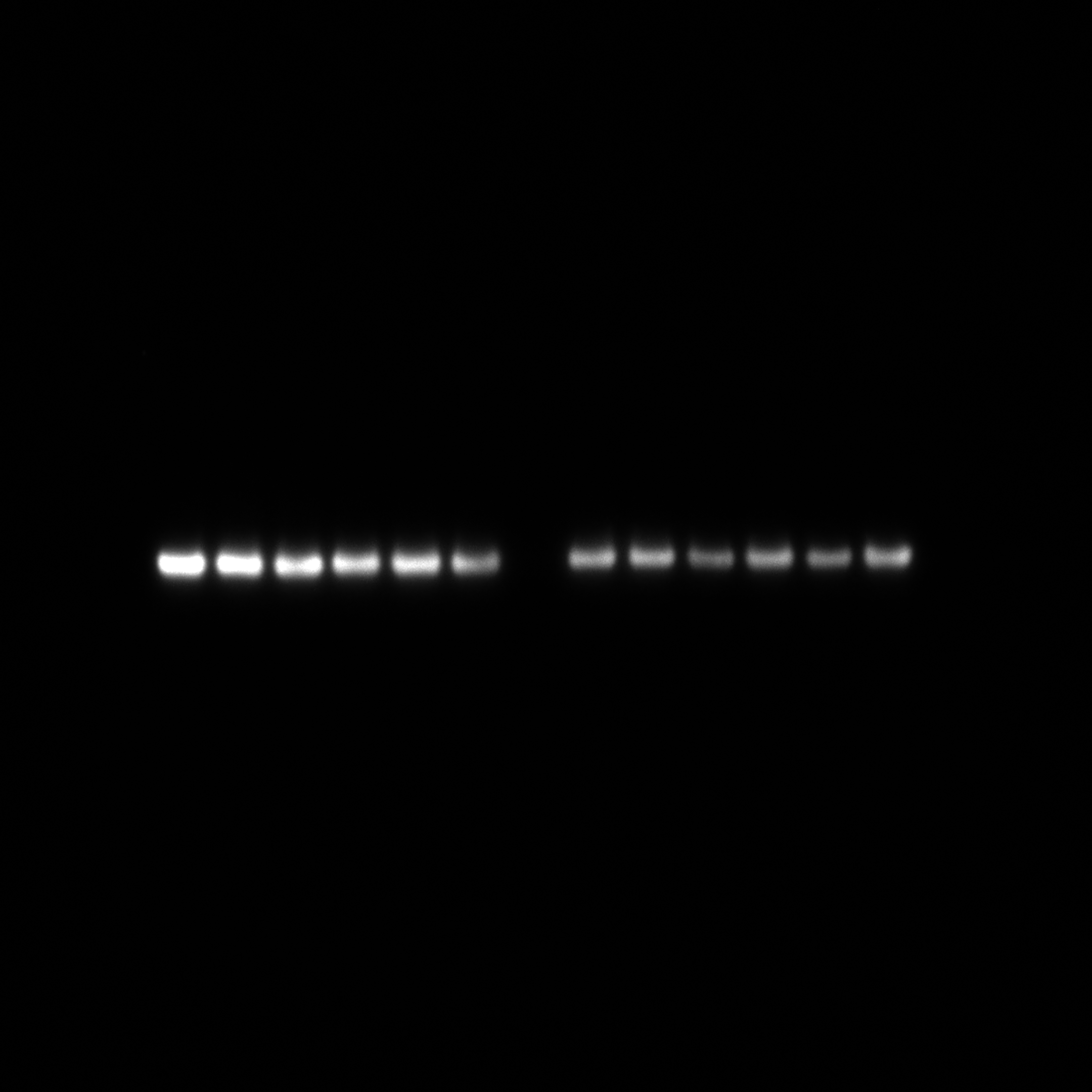

Supplement: Figure 4—source data 5. [file elife-106901-fig4-data5.zip › Figure4 source data 5/Figure 4E p38.Tif]

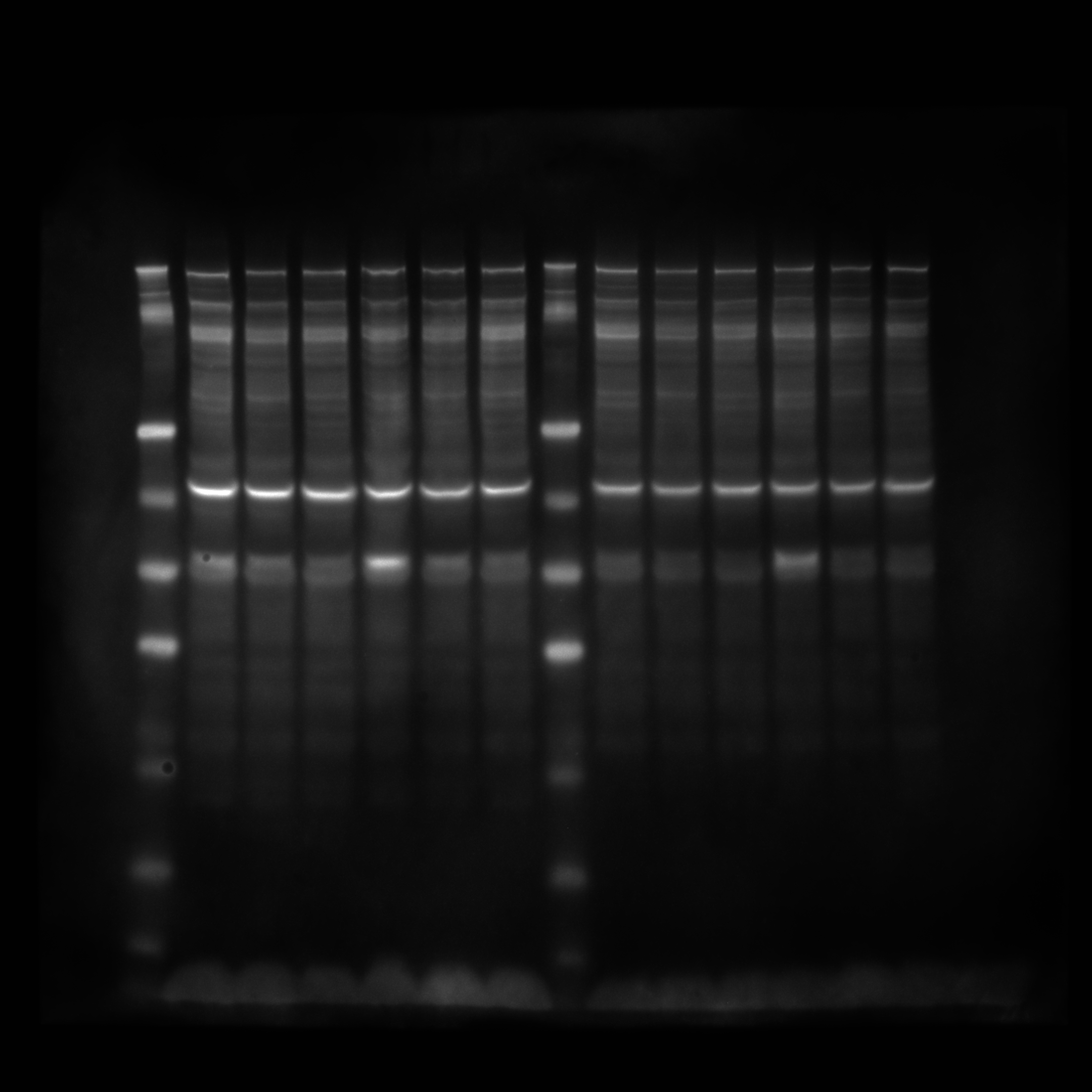

Supplement: Figure 4—source data 5. [file elife-106901-fig4-data5.zip › Figure4 source data 5/Figure 4E pIkBa.Tif]

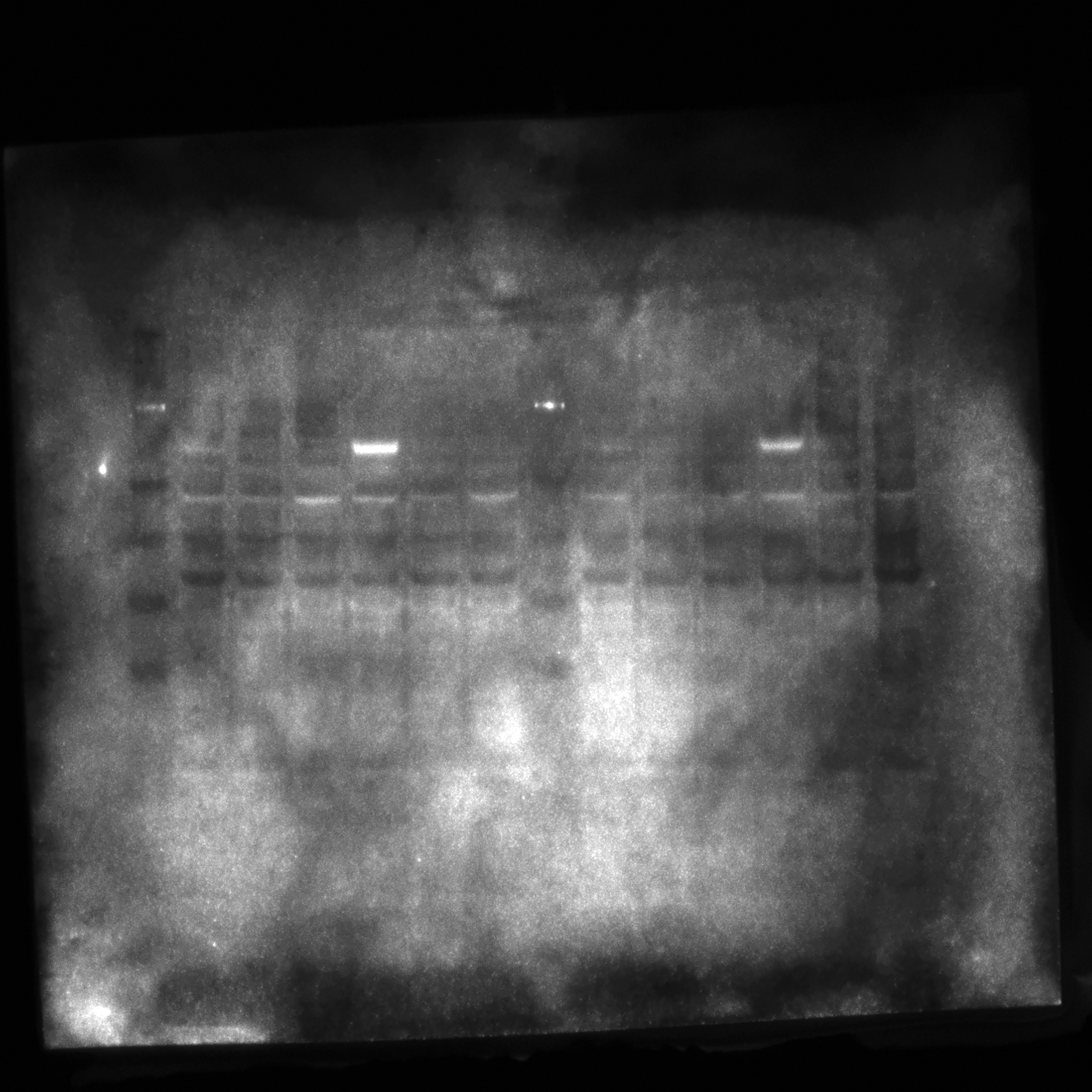

Supplement: Figure 4—source data 5. [file elife-106901-fig4-data5.zip › Figure4 source data 5/Figure 4E pIKKab.Tif]

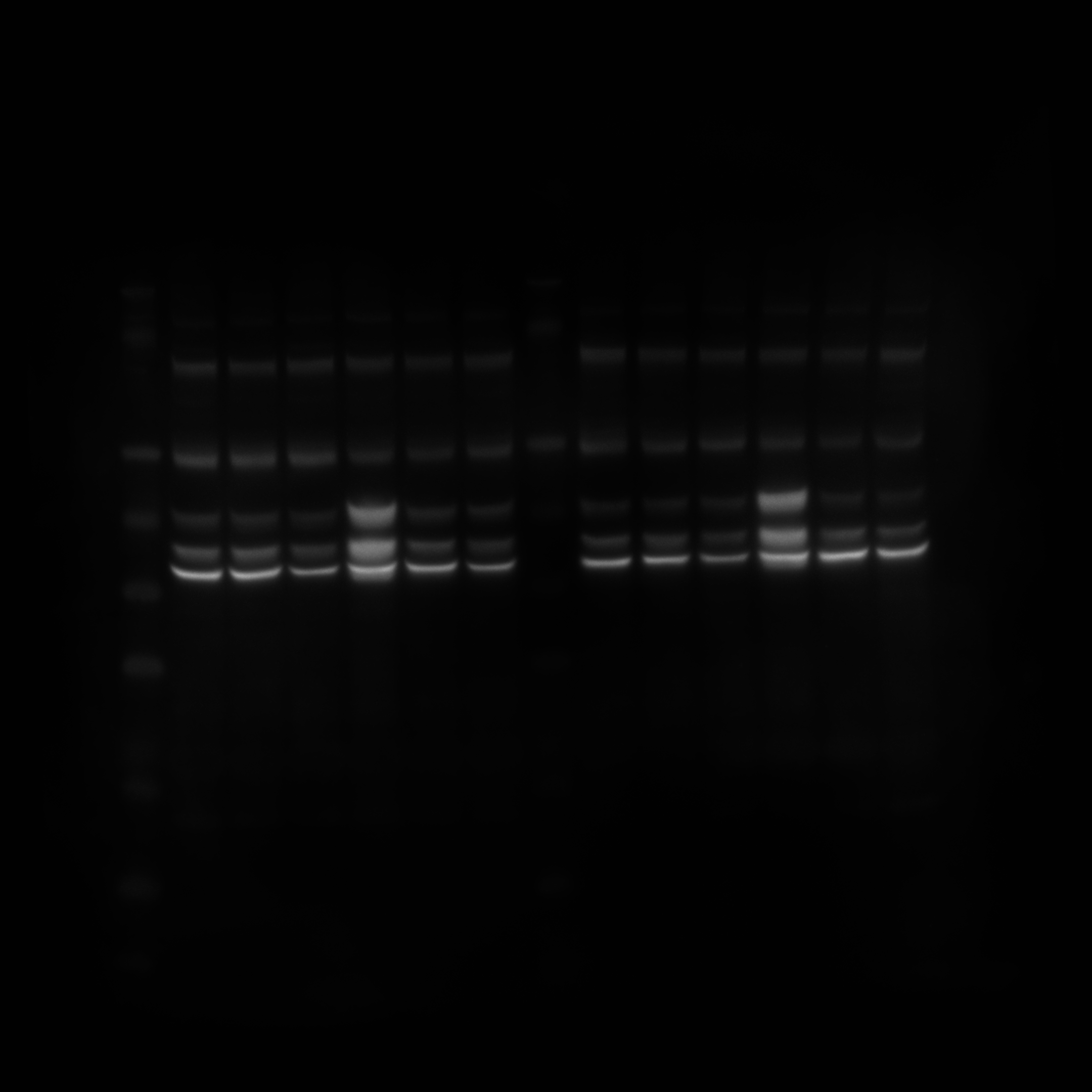

Supplement: Figure 4—source data 5. [file elife-106901-fig4-data5.zip › Figure4 source data 5/Figure 4E pJNK.Tif]

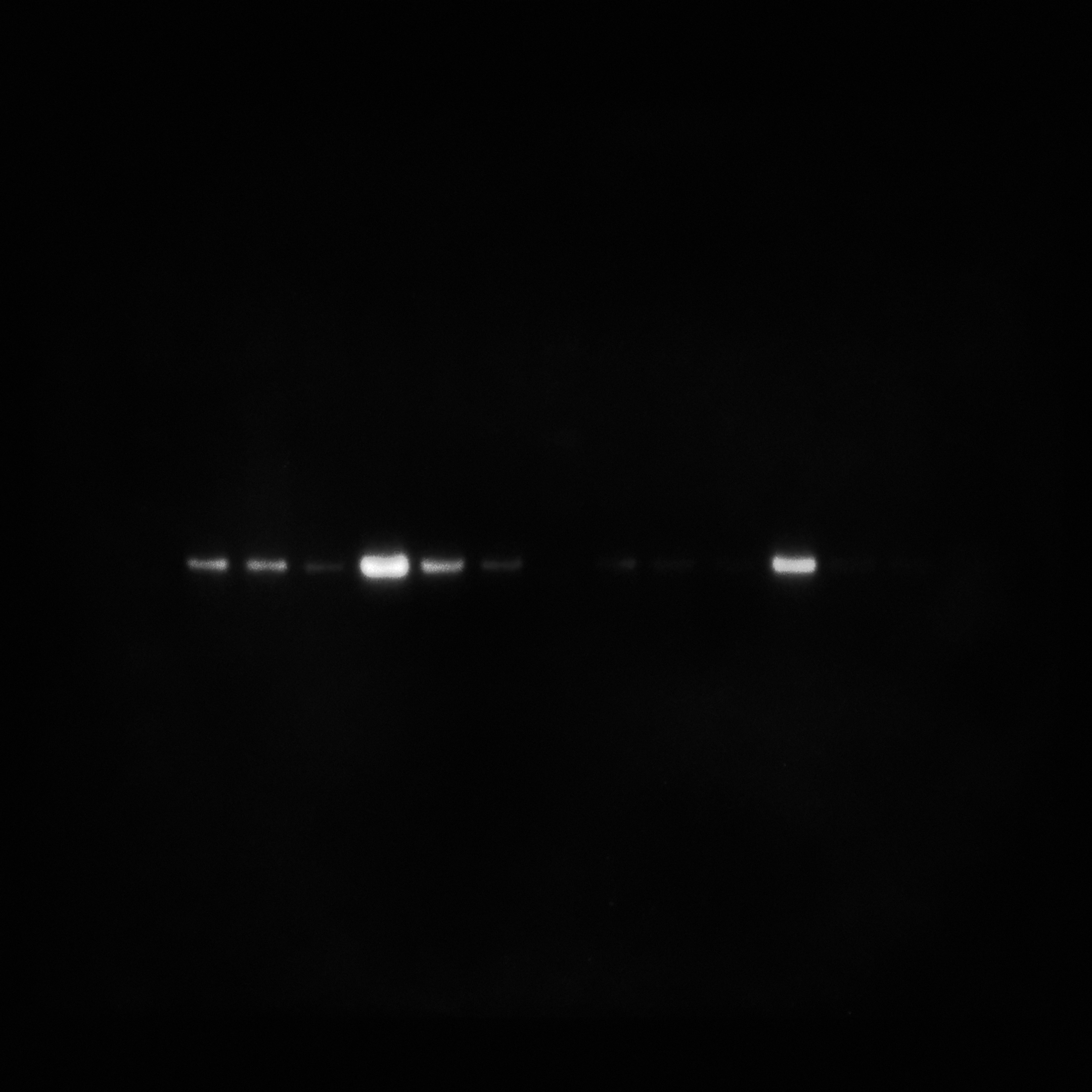

Supplement: Figure 4—source data 5. [file elife-106901-fig4-data5.zip › Figure4 source data 5/Figure 4E pp38.Tif]

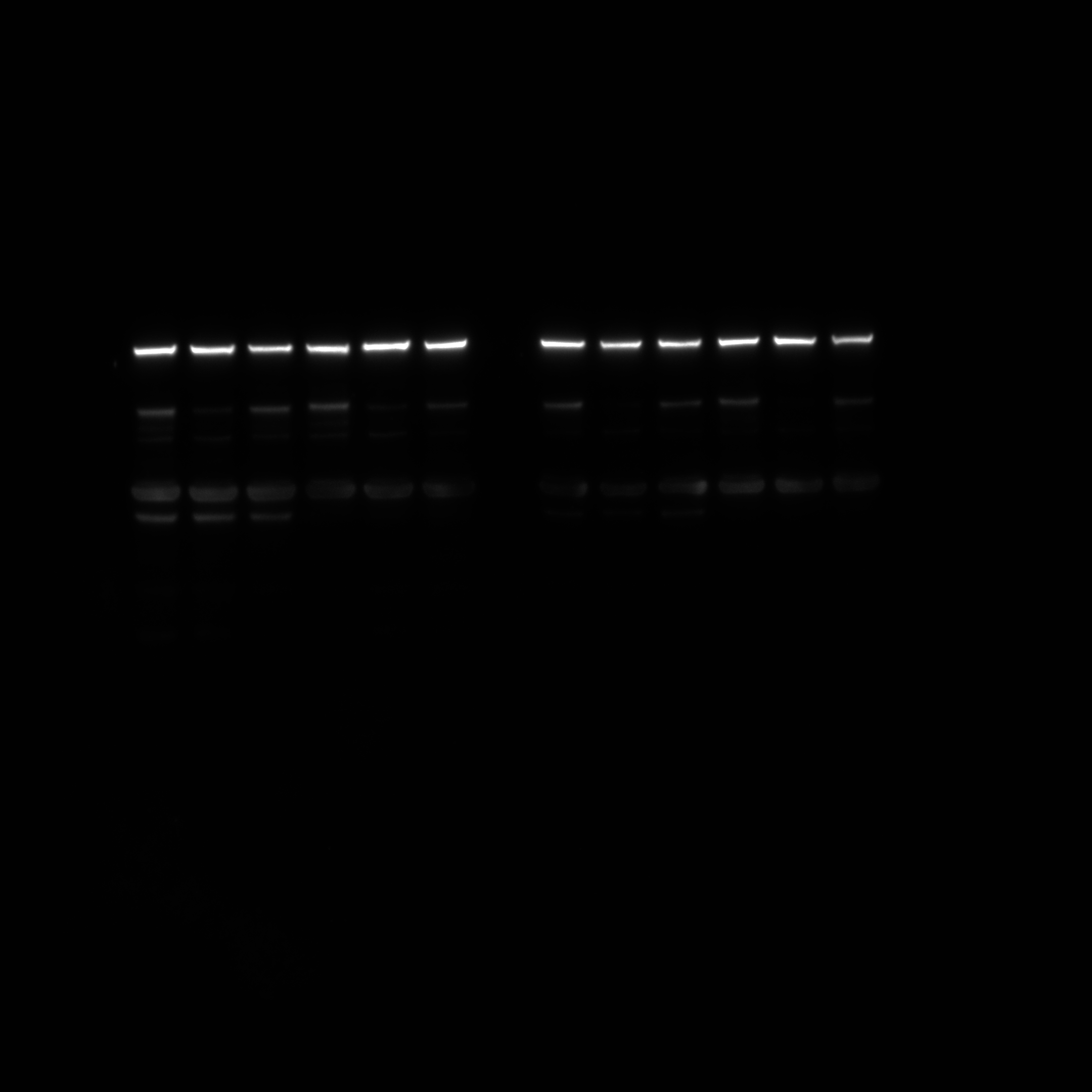

Supplement: Figure 4—source data 5. [file elife-106901-fig4-data5.zip › Figure4 source data 5/Figure 4E TAB2.Tif]

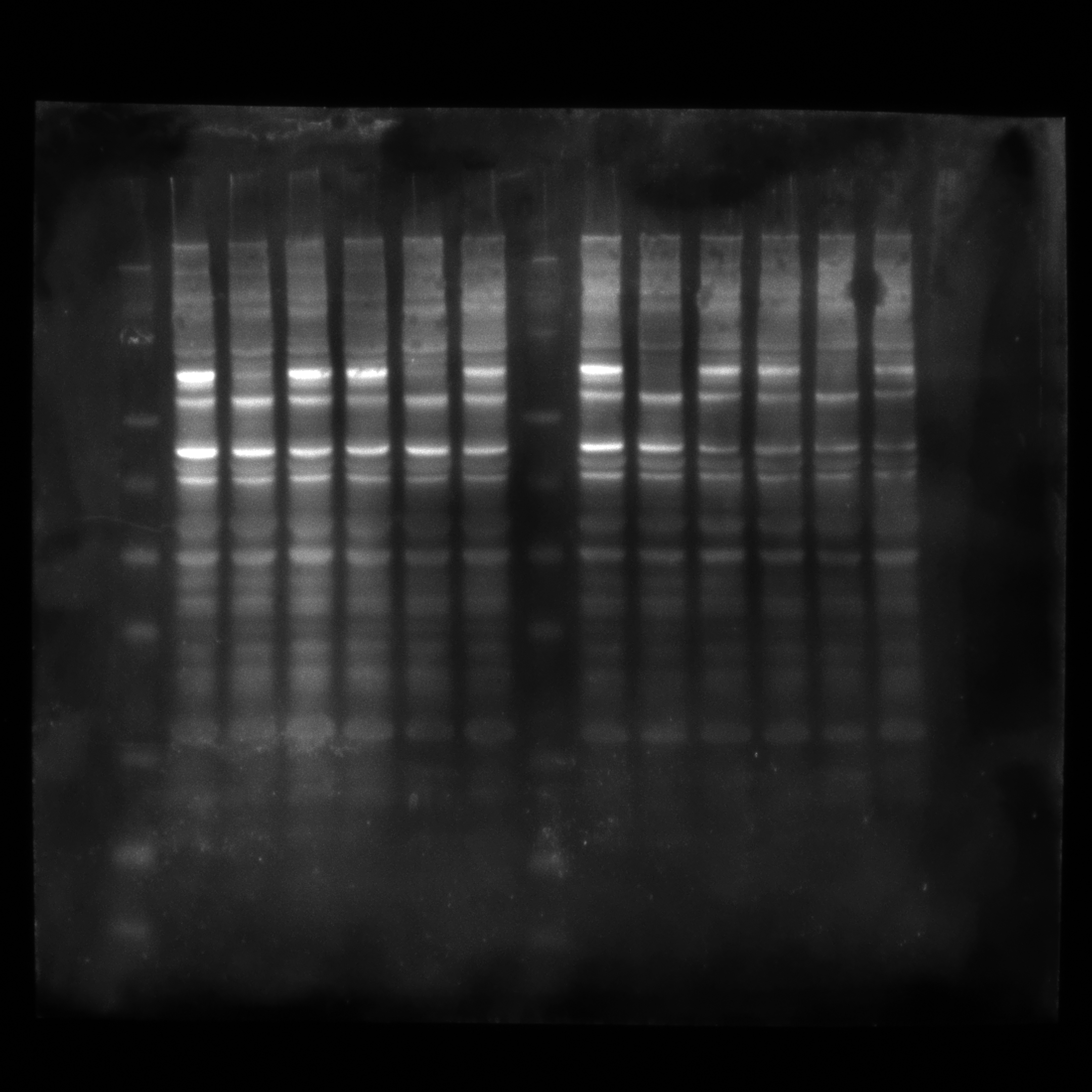

Supplement: Figure 4—source data 5. [file elife-106901-fig4-data5.zip › Figure4 source data 5/Figure 4E TAB3.Tif]

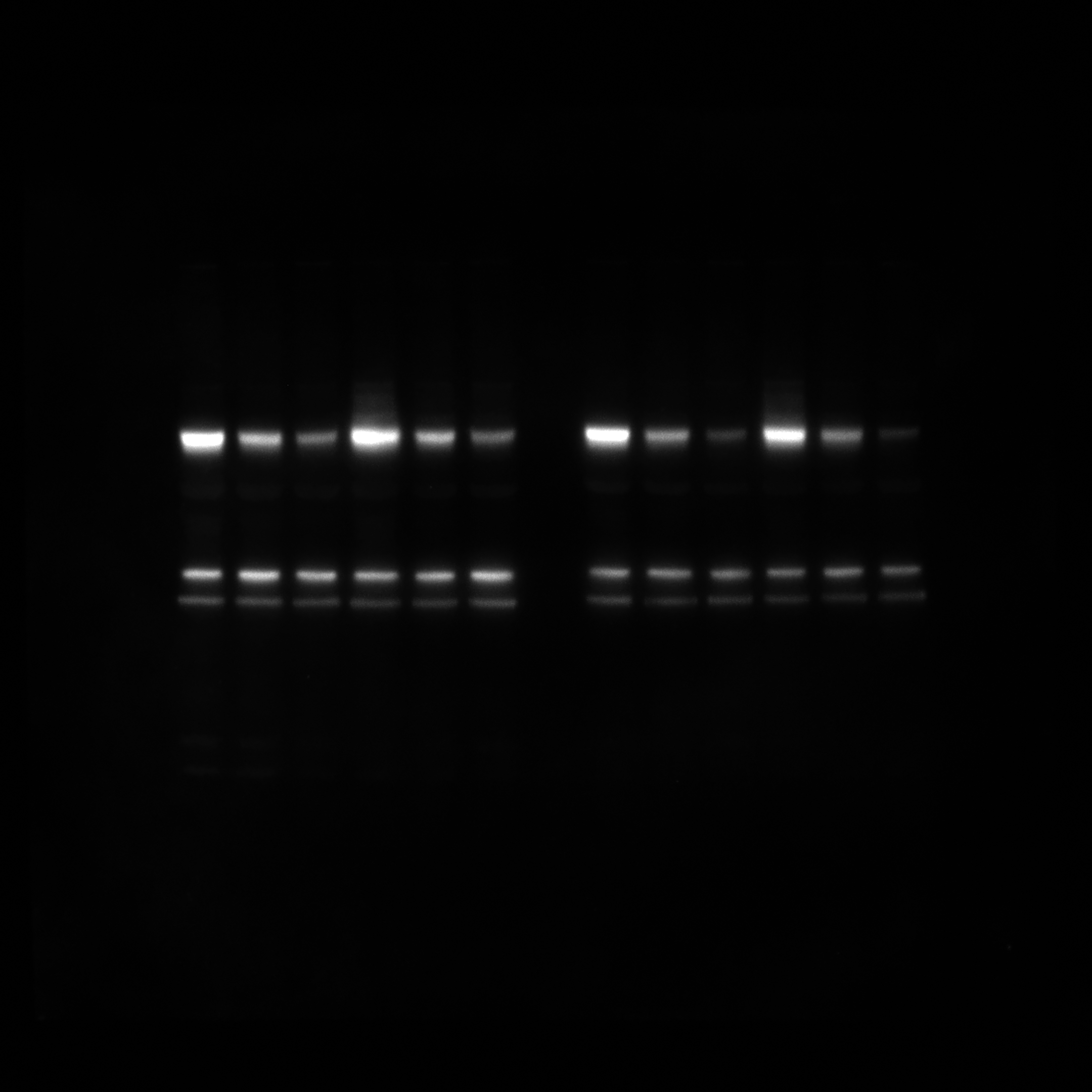

Supplement: Figure 4—source data 5. [file elife-106901-fig4-data5.zip › Figure4 source data 5/Figure 4E TAK1.Tif]

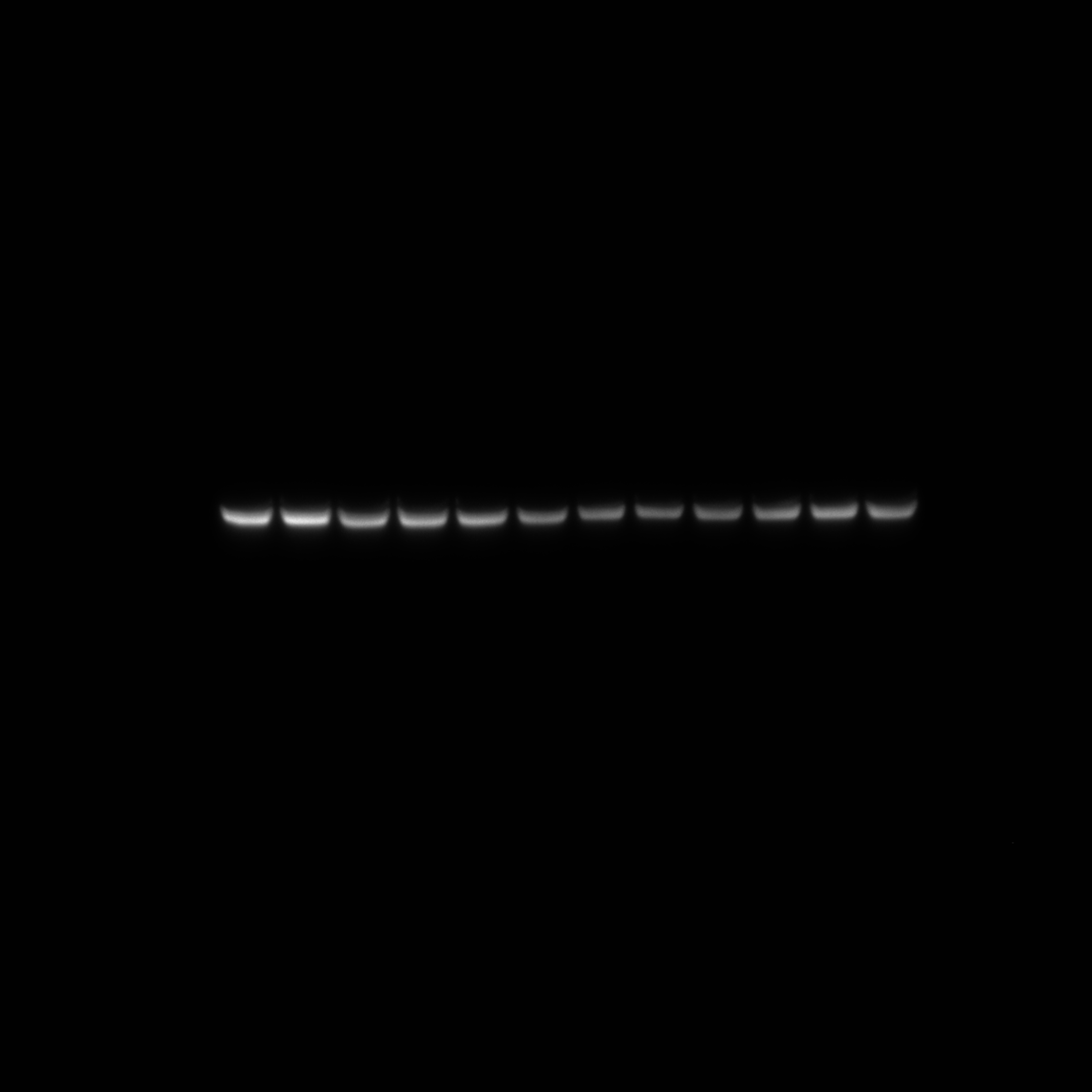

Supplement: Figure 4—source data 5. [file elife-106901-fig4-data5.zip › Figure4 source data 5/Figure 4E Tubulin.Tif]

**Figure 4E**

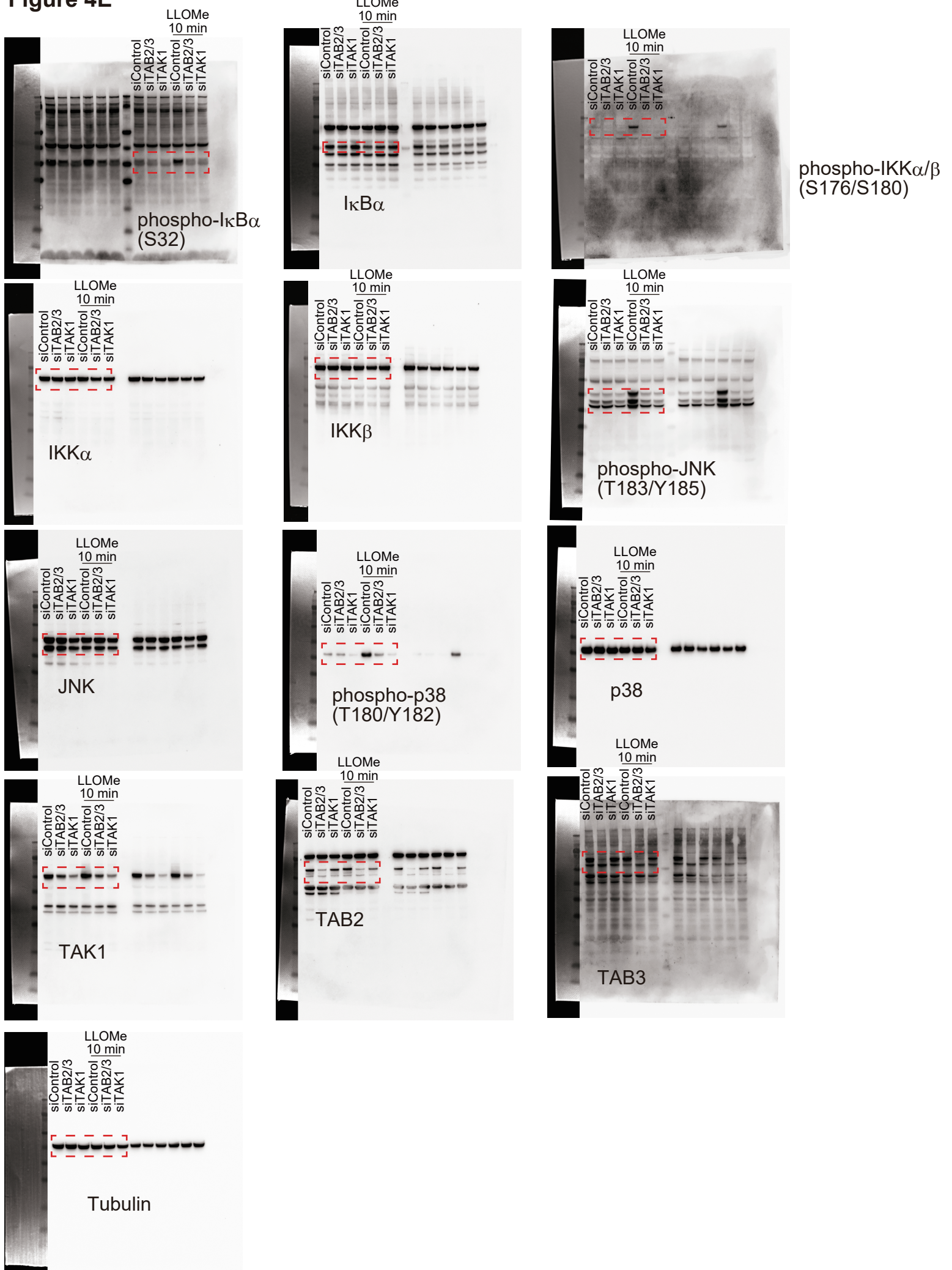

Supplement: Figure 4—source data 6. [file elife-106901-fig4-data6.pdf]

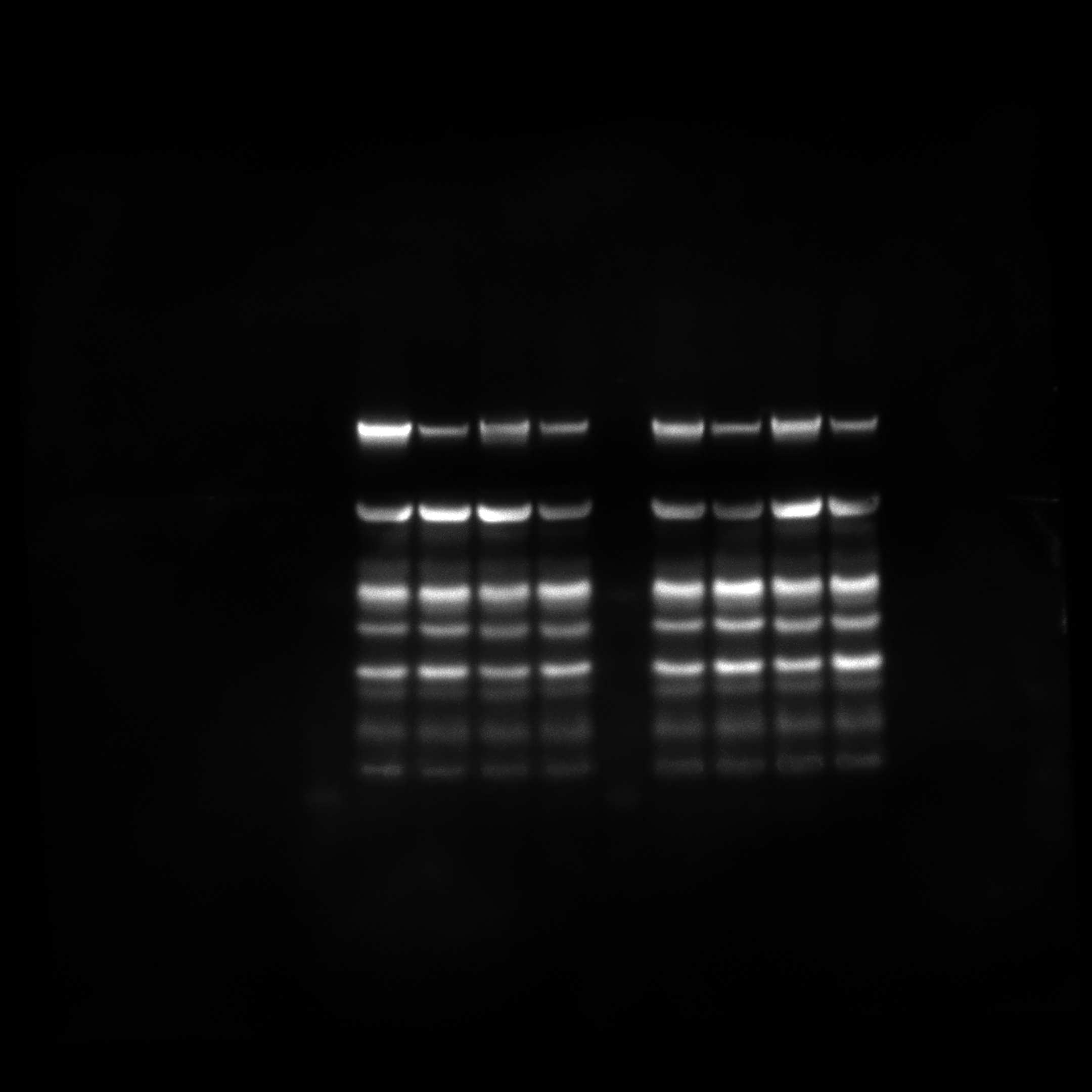

Supplement: Figure 4—source data 7. [file elife-106901-fig4-data7.zip › Figure4 source data 7/Figure 4F IkBa.Tif]

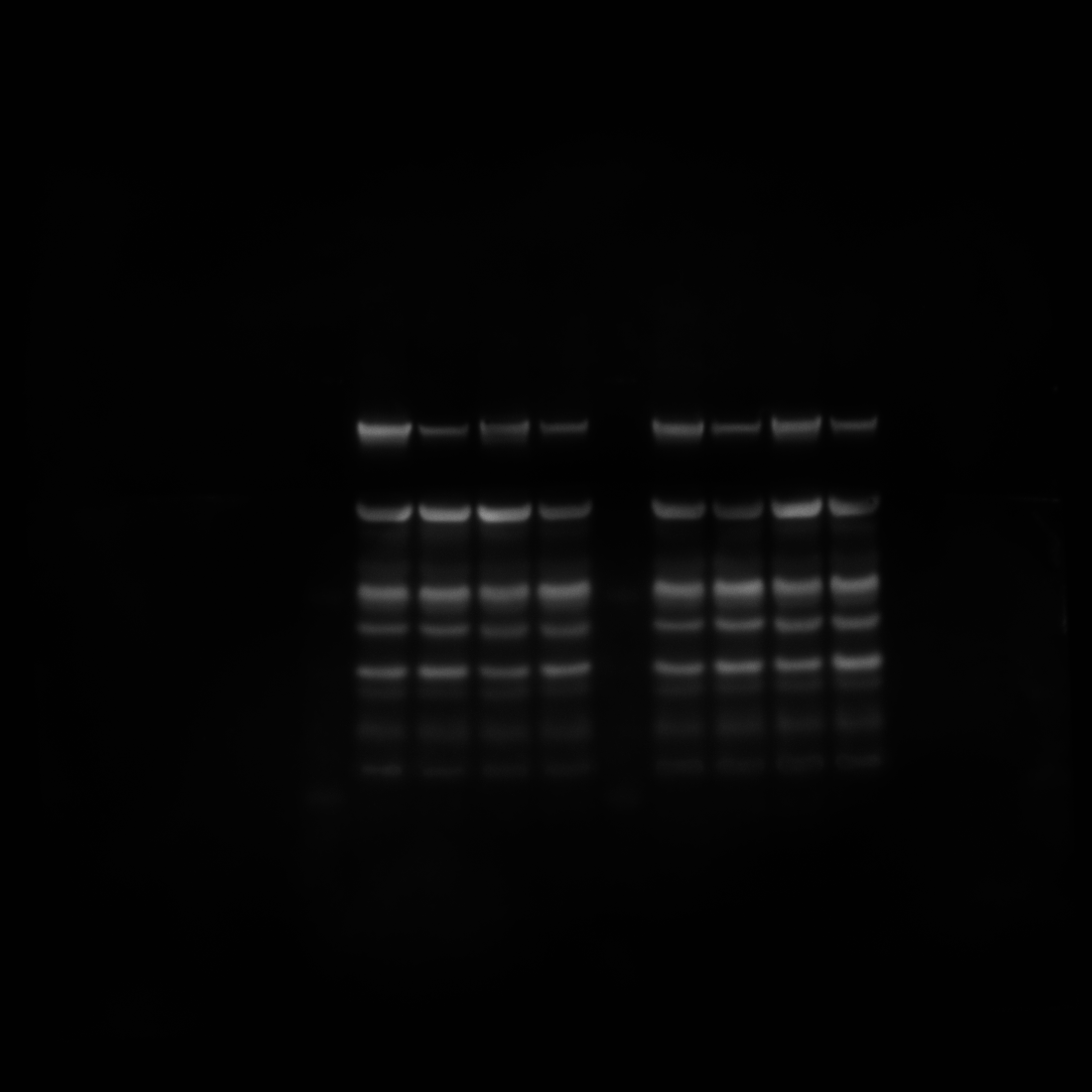

Supplement: Figure 4—source data 7. [file elife-106901-fig4-data7.zip › Figure4 source data 7/Figure 4F IKKa.Tif]

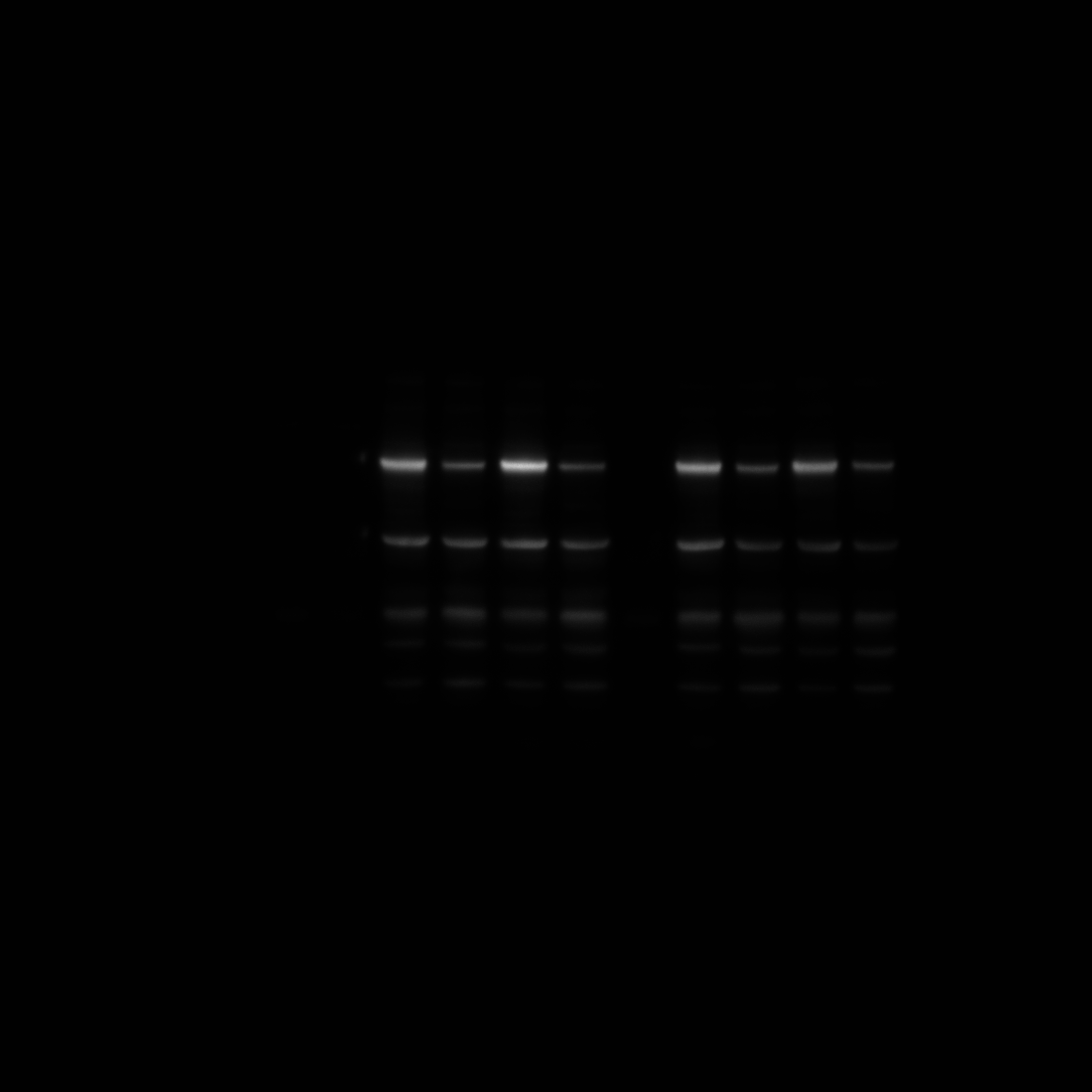

Supplement: Figure 4—source data 7. [file elife-106901-fig4-data7.zip › Figure4 source data 7/Figure 4F IKKb.Tif]

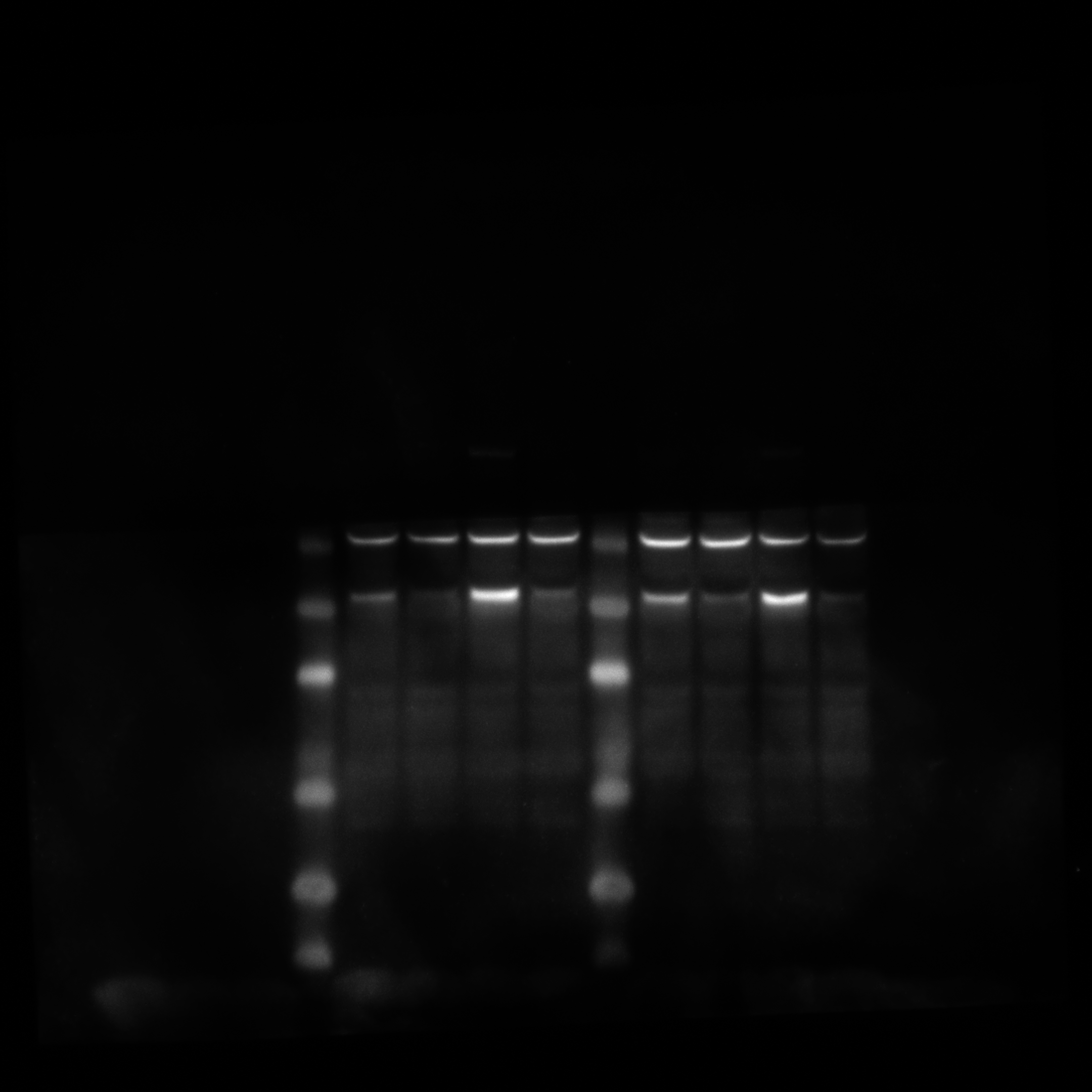

Supplement: Figure 4—source data 7. [file elife-106901-fig4-data7.zip › Figure4 source data 7/Figure 4F pIkBa.Tif]

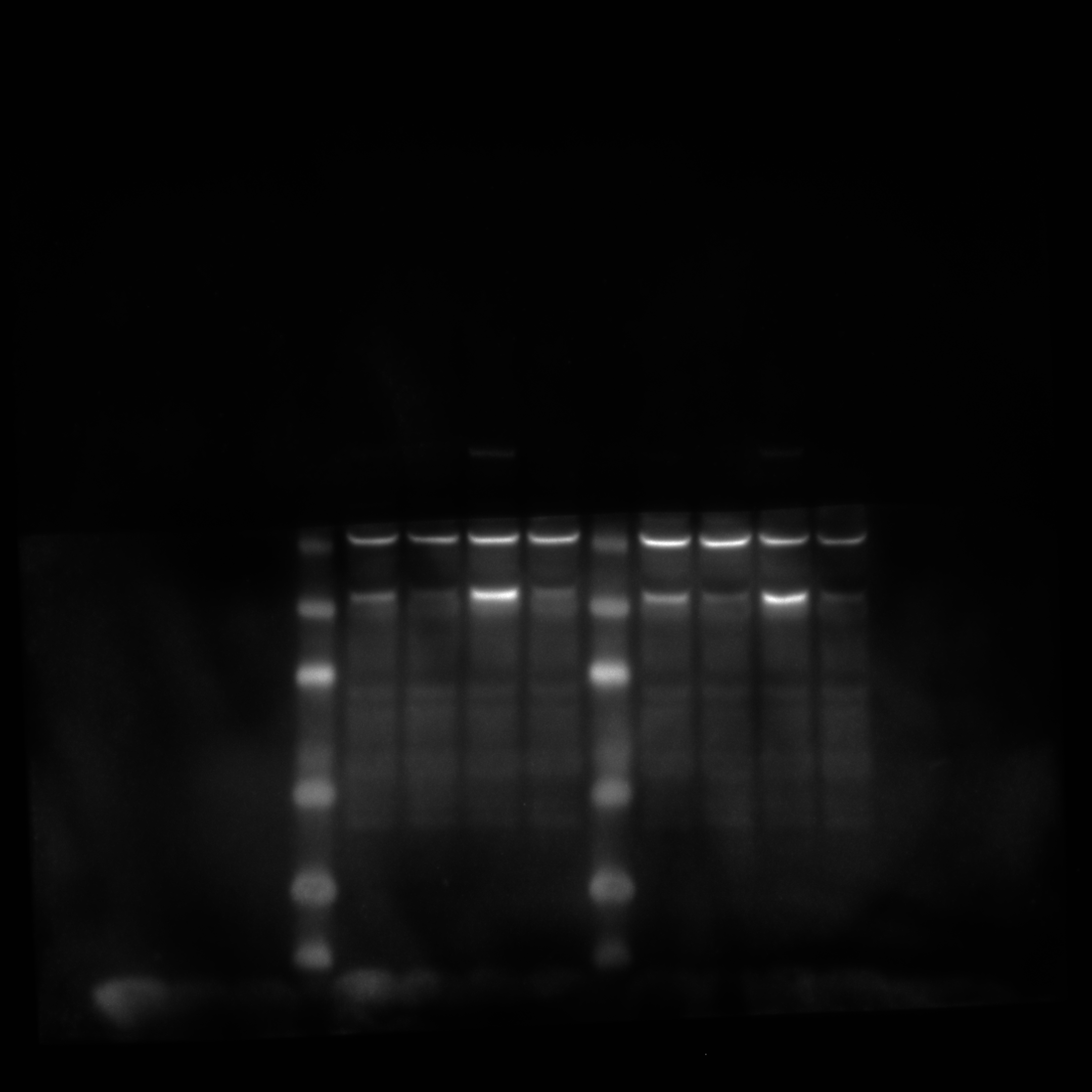

Supplement: Figure 4—source data 7. [file elife-106901-fig4-data7.zip › Figure4 source data 7/Figure 4F pIKKab.Tif]

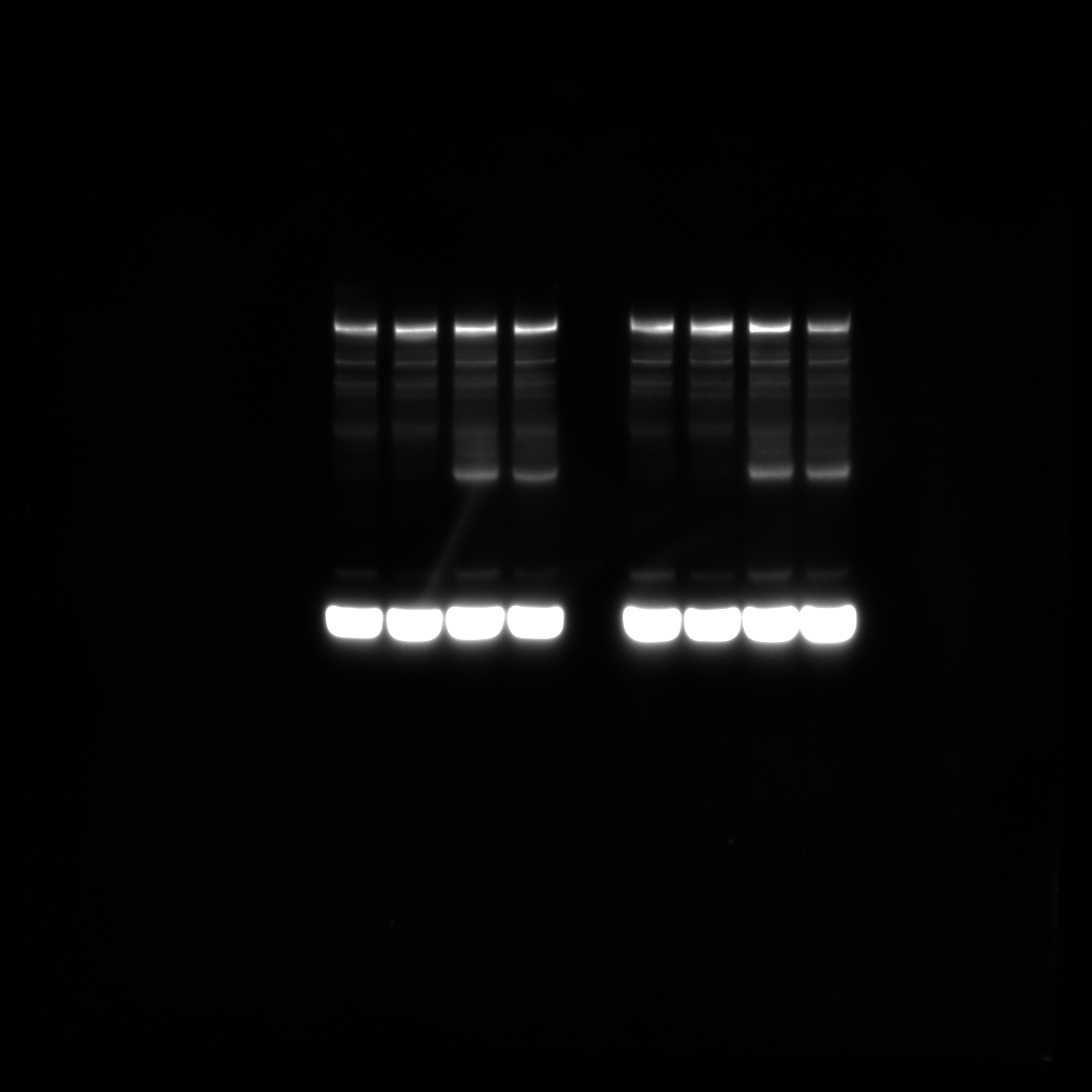

Supplement: Figure 4—source data 7. [file elife-106901-fig4-data7.zip › Figure4 source data 7/Figure 4F pTAK1.Tif]

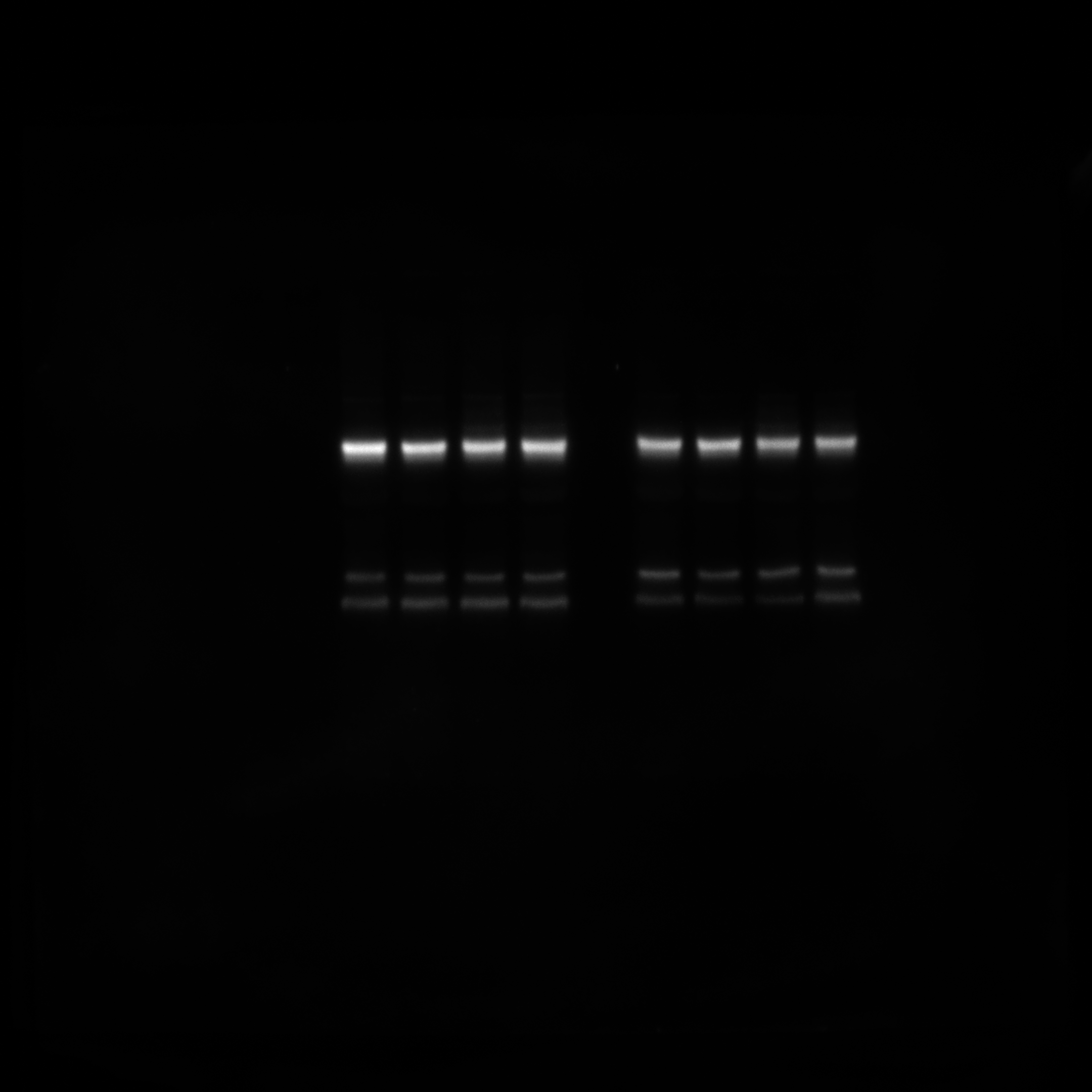

Supplement: Figure 4—source data 7. [file elife-106901-fig4-data7.zip › Figure4 source data 7/Figure 4F TAK1.Tif]

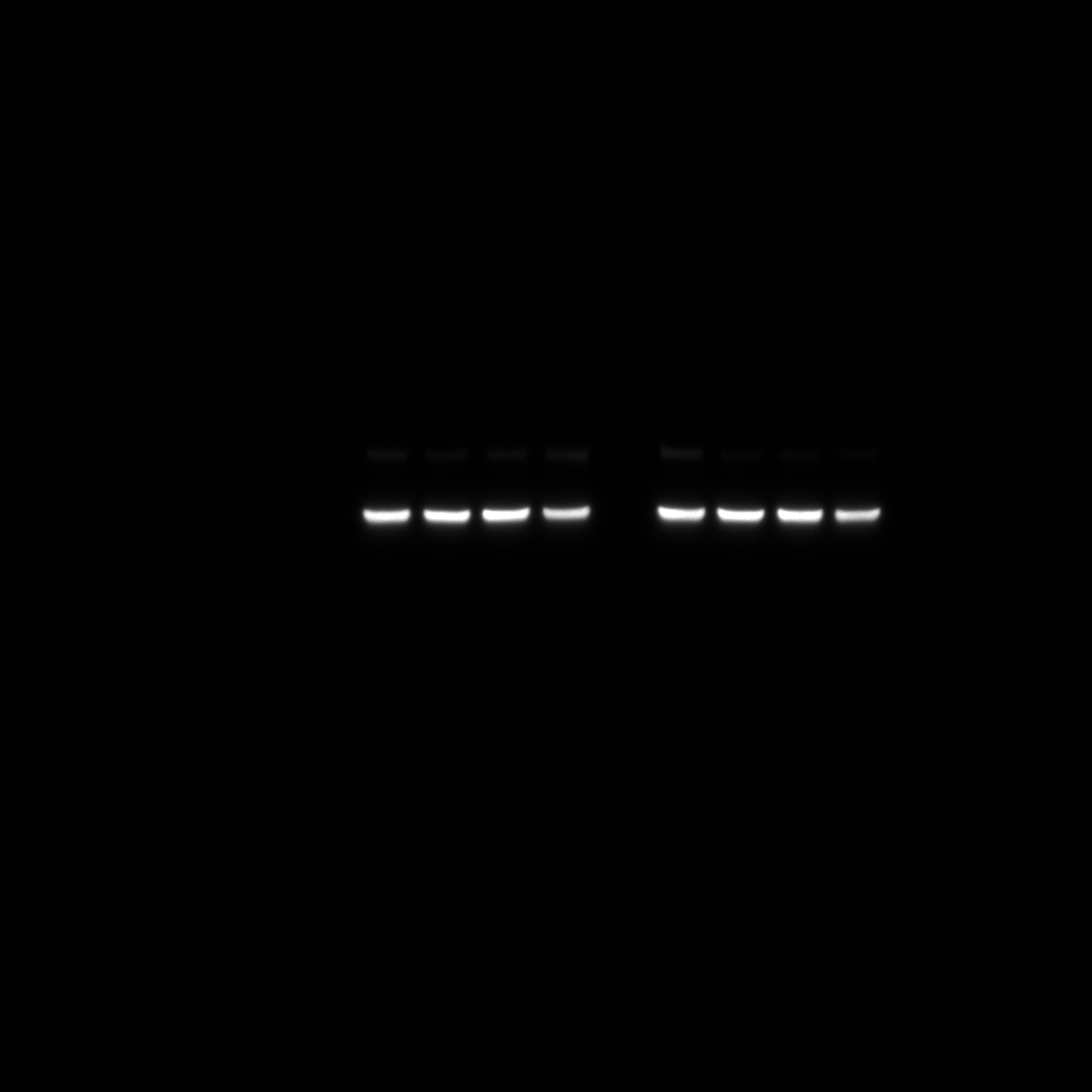

Supplement: Figure 4—source data 7. [file elife-106901-fig4-data7.zip › Figure4 source data 7/Figure 4F Tubulin.Tif]

Figure 4F

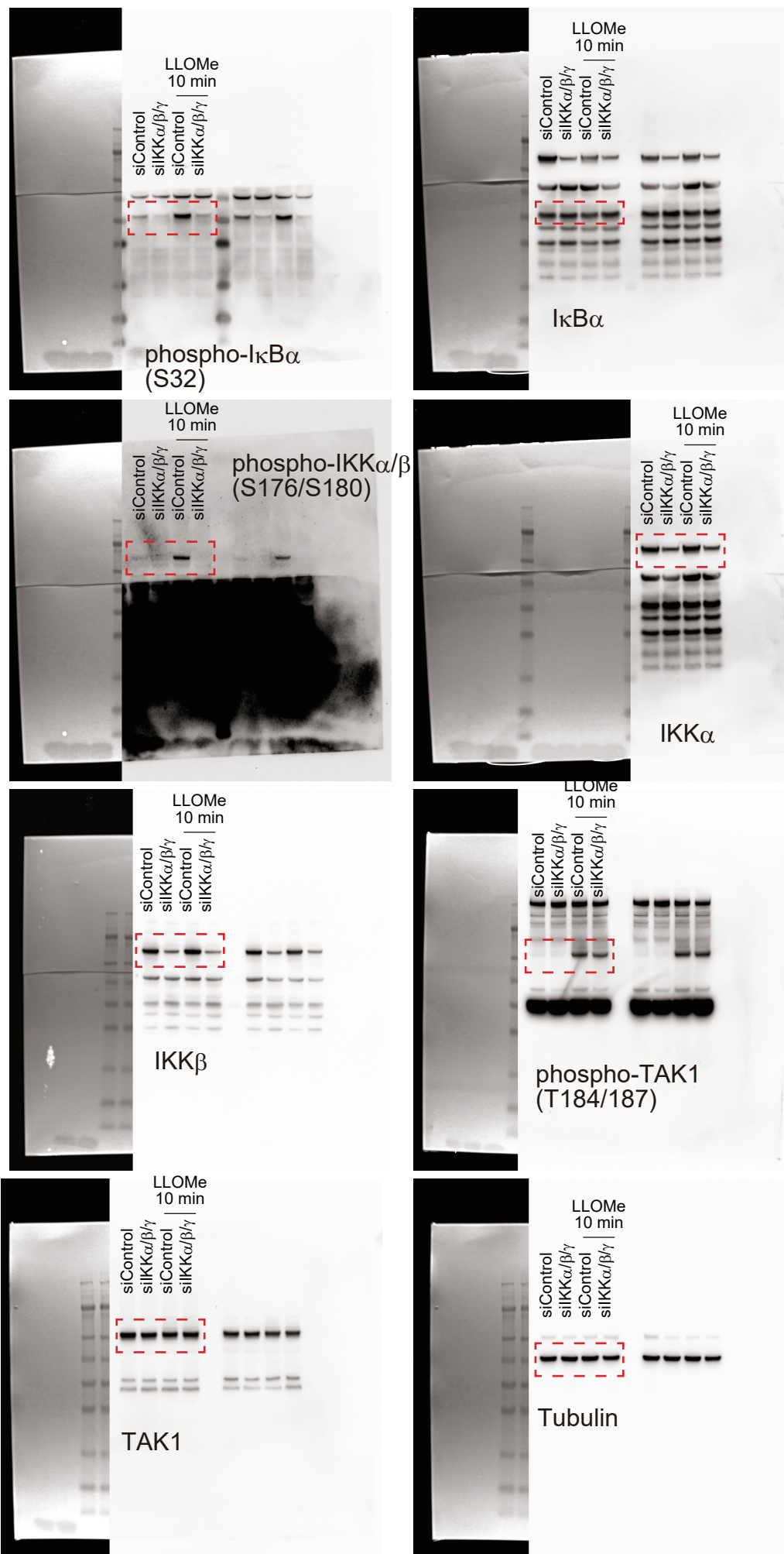

Supplement: Figure 4—source data 8. [file elife-106901-fig4-data8.pdf]

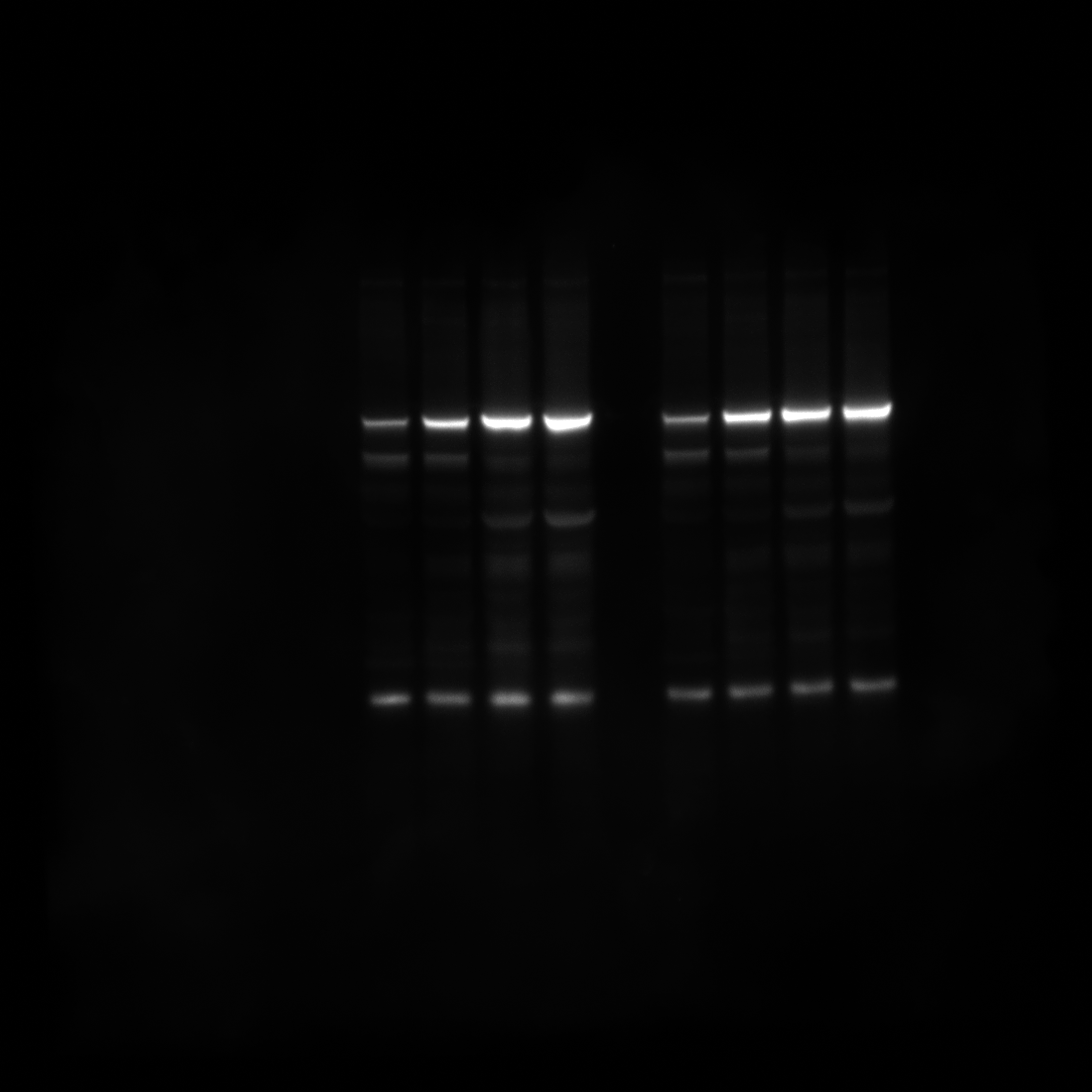

Supplement: Figure 4—figure supplement 1—source data 1. [file elife-106901-fig4-figsupp1-data1.zip › Figure4 figure supplement 1 source data 1/Figure S4D pTBK1.Tif]

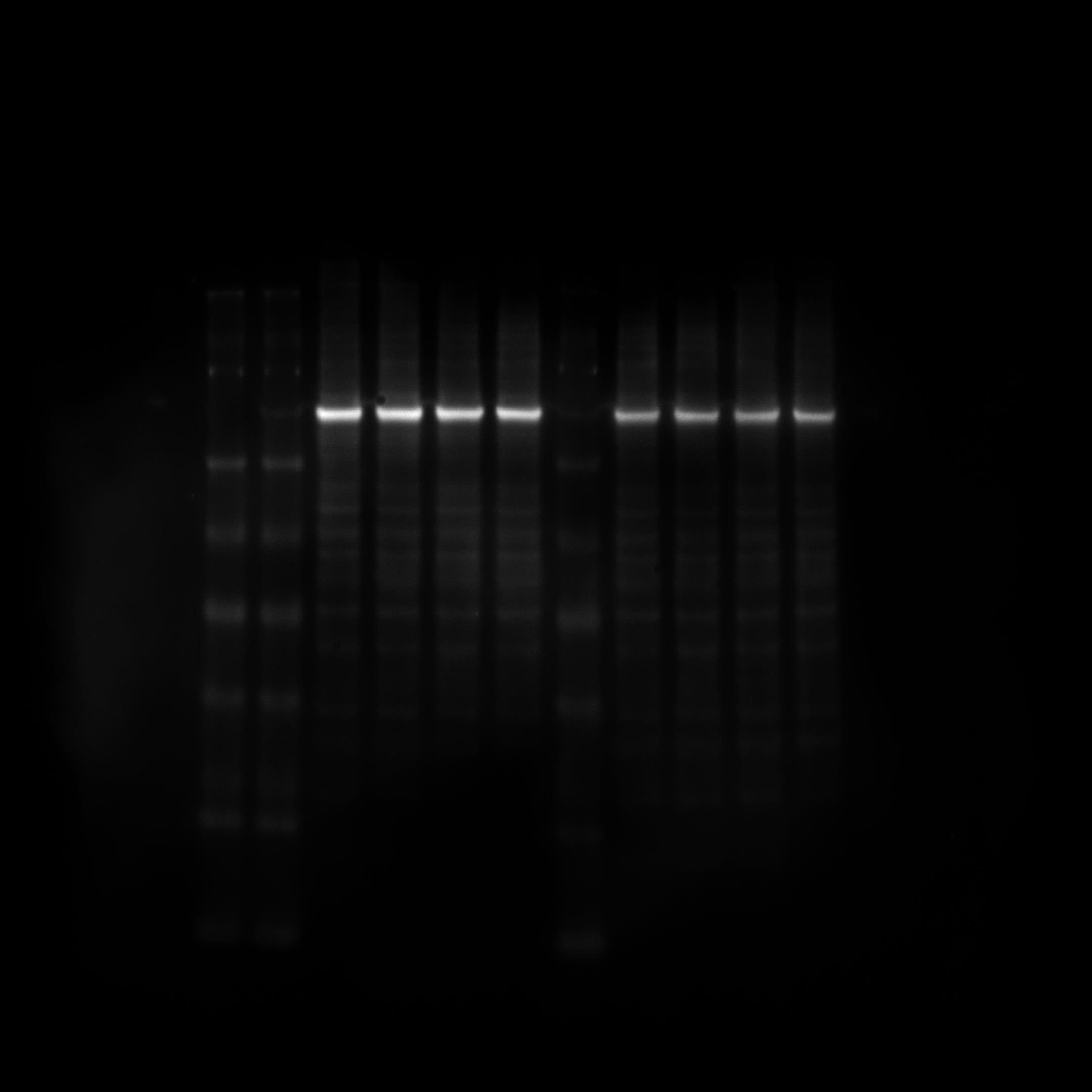

Supplement: Figure 4—figure supplement 1—source data 1. [file elife-106901-fig4-figsupp1-data1.zip › Figure4 figure supplement 1 source data 1/Figure S4D TBK1.Tif]

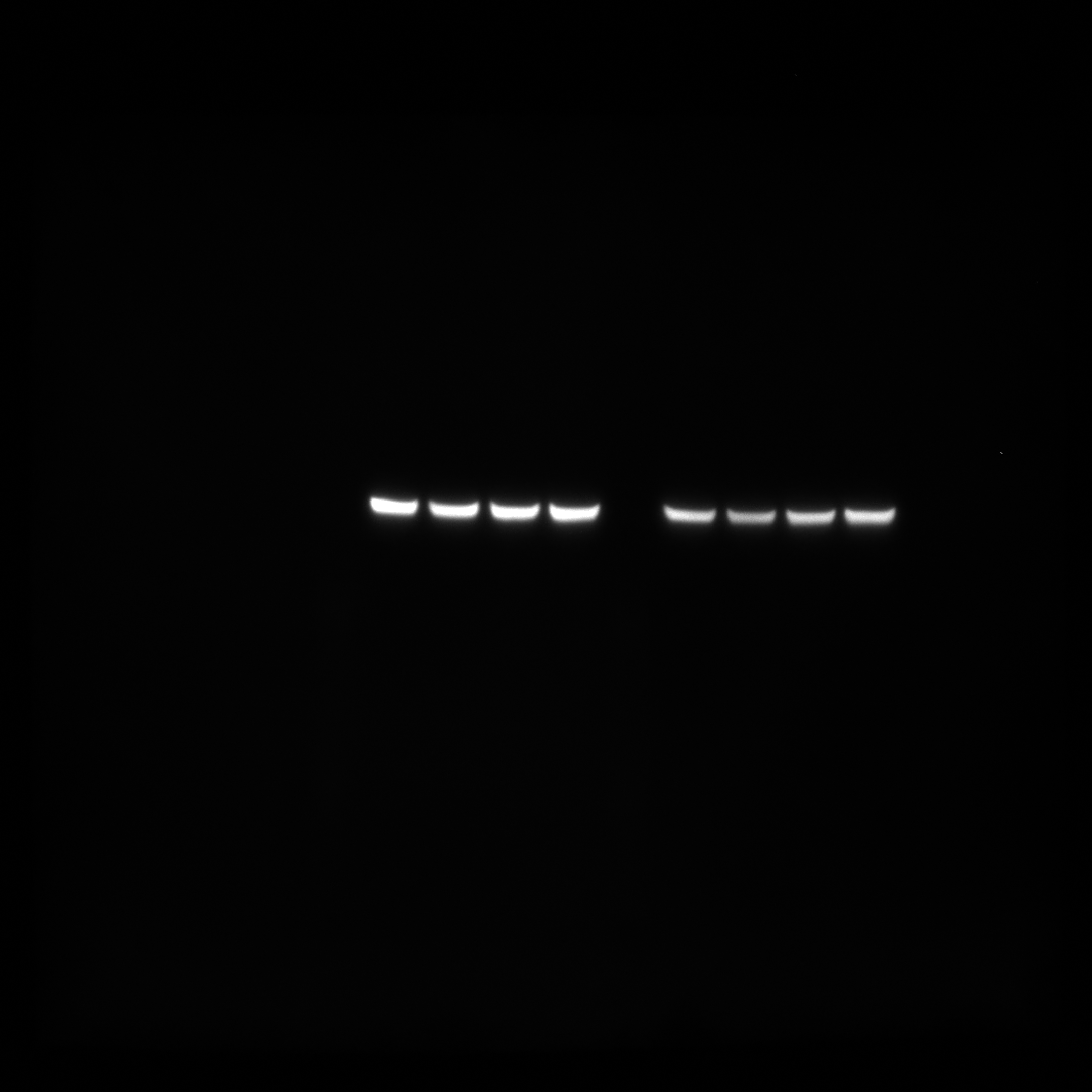

Supplement: Figure 4—figure supplement 1—source data 1. [file elife-106901-fig4-figsupp1-data1.zip › Figure4 figure supplement 1 source data 1/Figure S4D Tubulin.Tif]

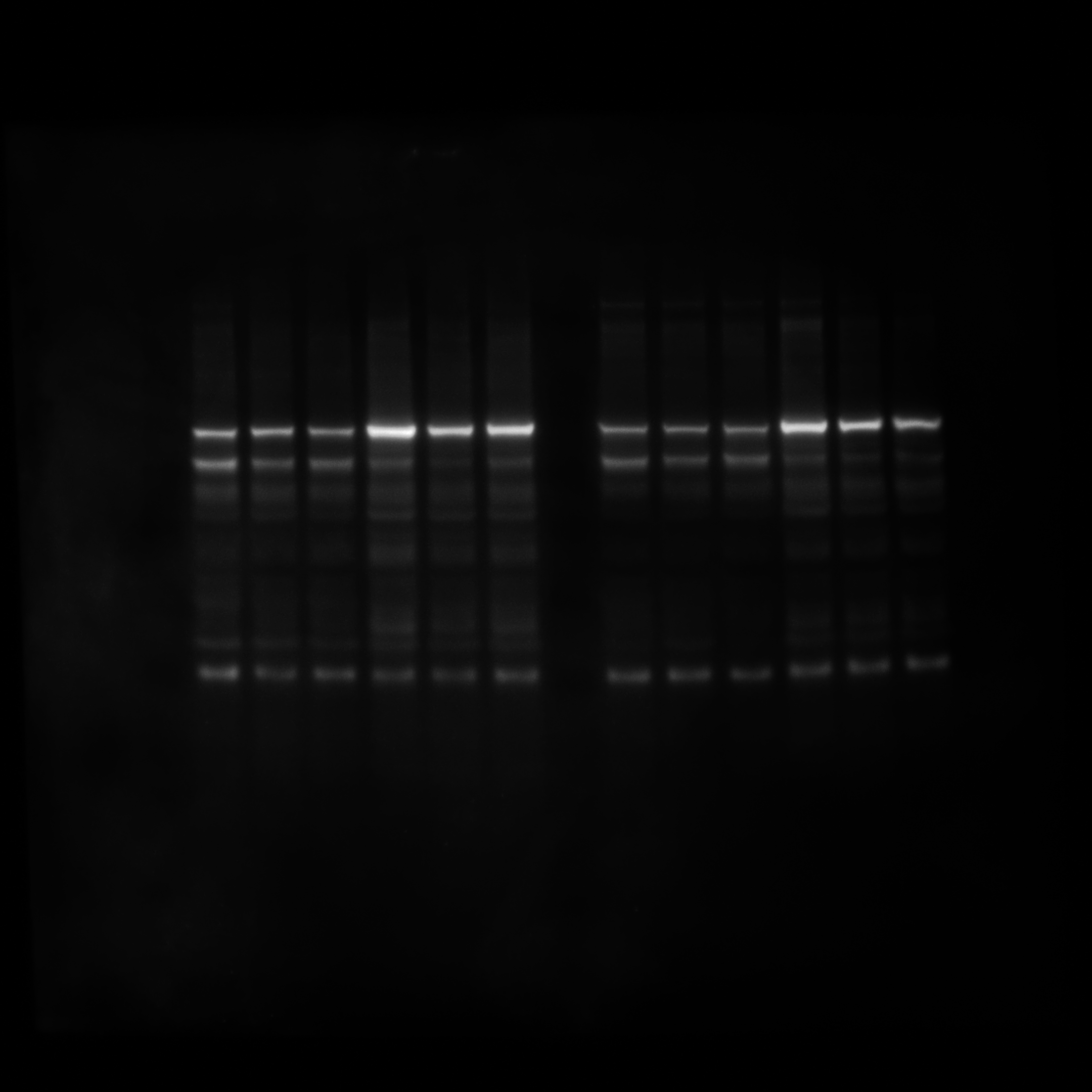

Supplement: Figure 4—figure supplement 1—source data 1. [file elife-106901-fig4-figsupp1-data1.zip › Figure4 figure supplement 1 source data 1/Figure S4E pTBK1.Tif]

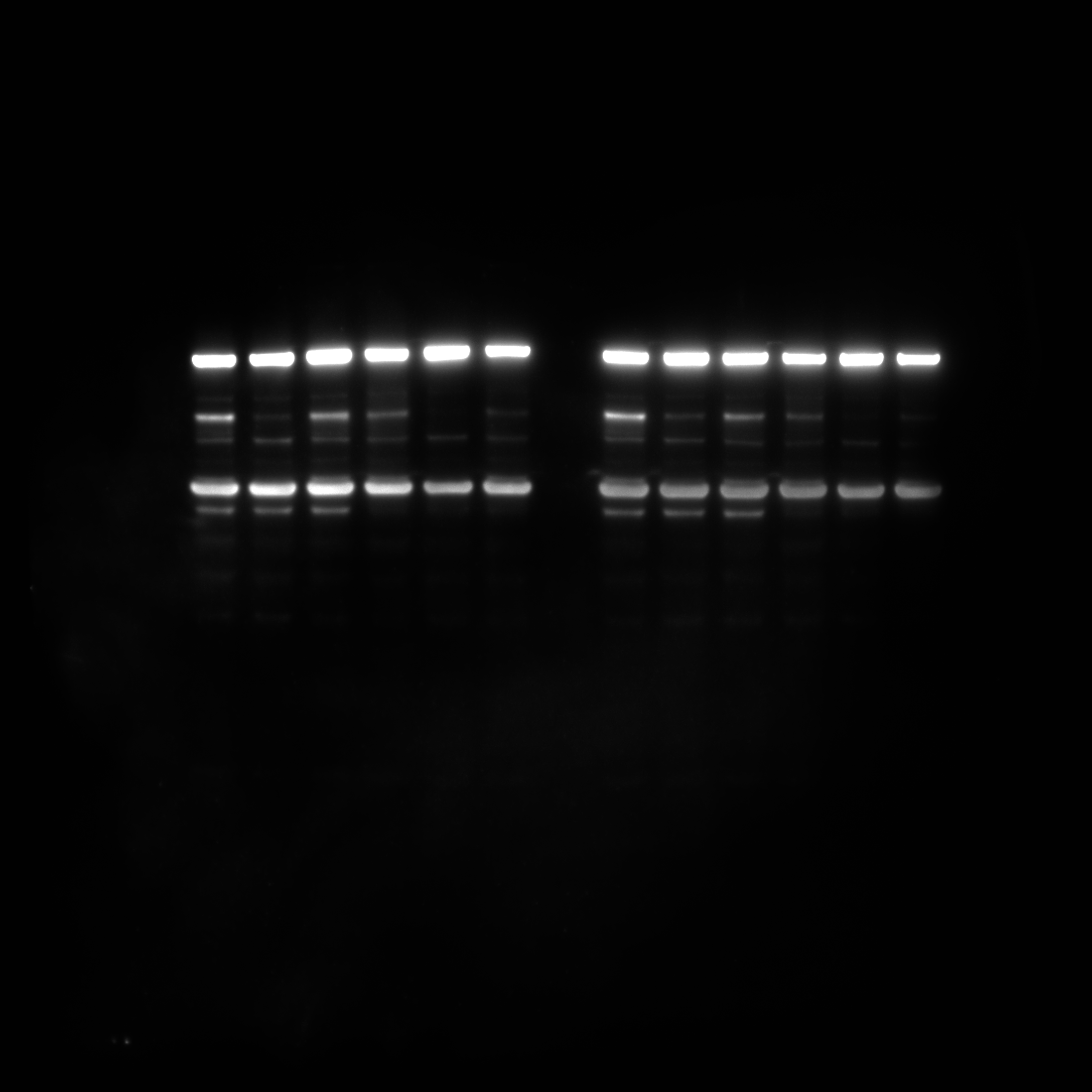

Supplement: Figure 4—figure supplement 1—source data 1. [file elife-106901-fig4-figsupp1-data1.zip › Figure4 figure supplement 1 source data 1/Figure S4E TAB2.Tif]

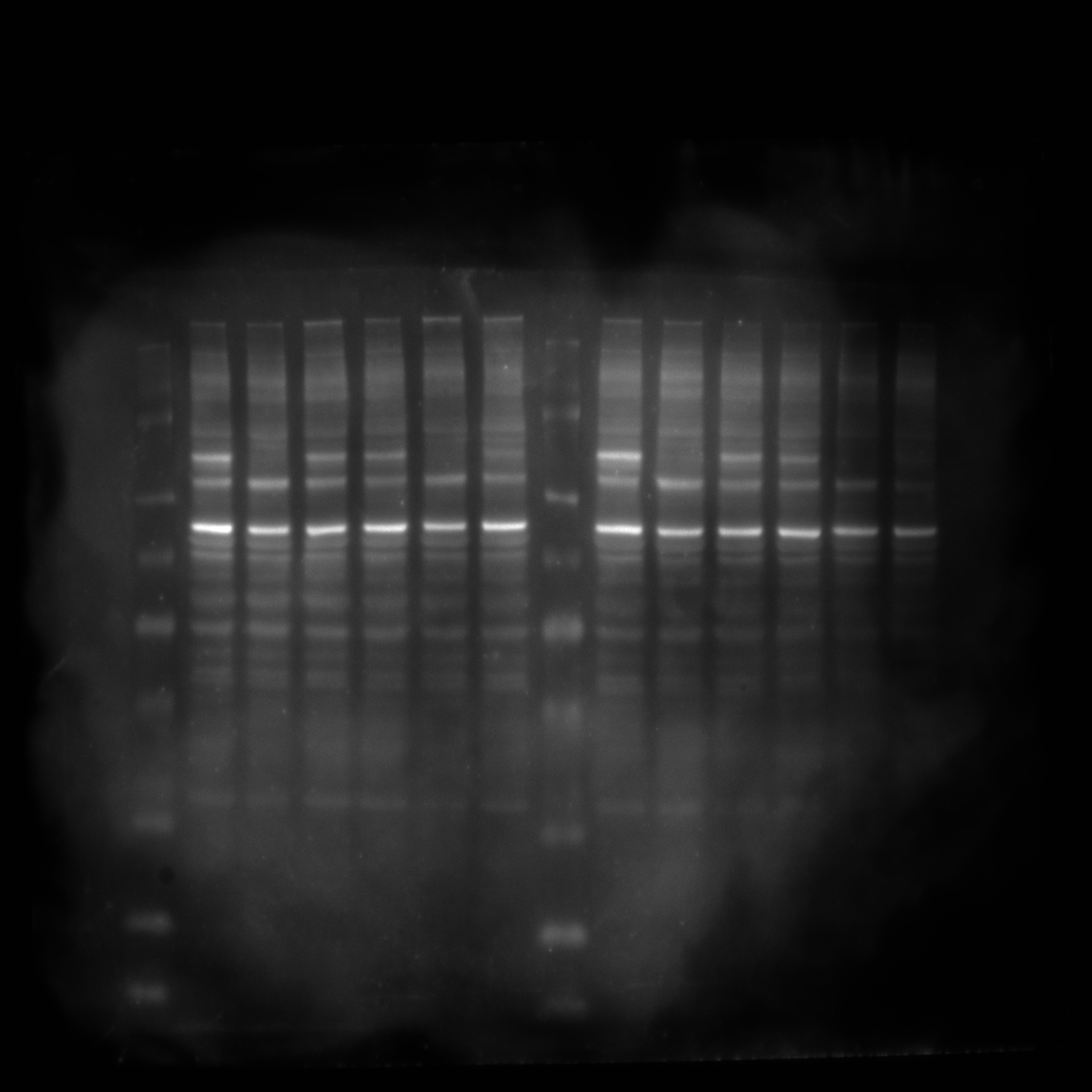

Supplement: Figure 4—figure supplement 1—source data 1. [file elife-106901-fig4-figsupp1-data1.zip › Figure4 figure supplement 1 source data 1/Figure S4E TAB3.Tif]
