# Supplementary figures and images for: Ubiquitination-activated TAB–TAK1–IKK–NF-κB axis modulates gene expression for cell survival in the lysosomal damage response (part 2 of 2)
Source: eLife. 2025 Sep 24;14:RP106901. doi: 10.7554/eLife.106901 (PMC12459955; doi:10.7554/eLife.106901)

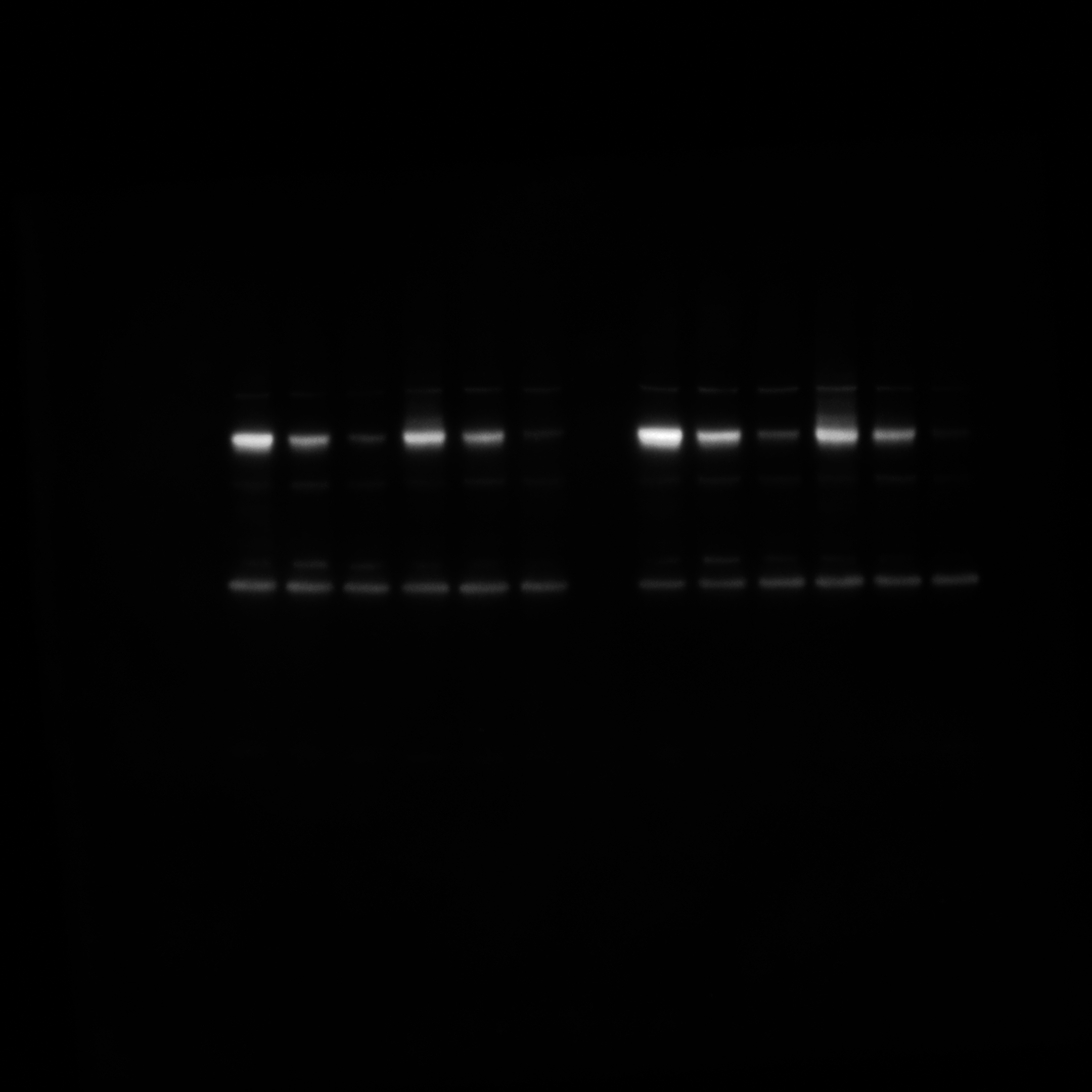

Supplement: Figure 4—figure supplement 1—source data 1. [file elife-106901-fig4-figsupp1-data1.zip › Figure4 figure supplement 1 source data 1/Figure S4E TAK1.Tif]

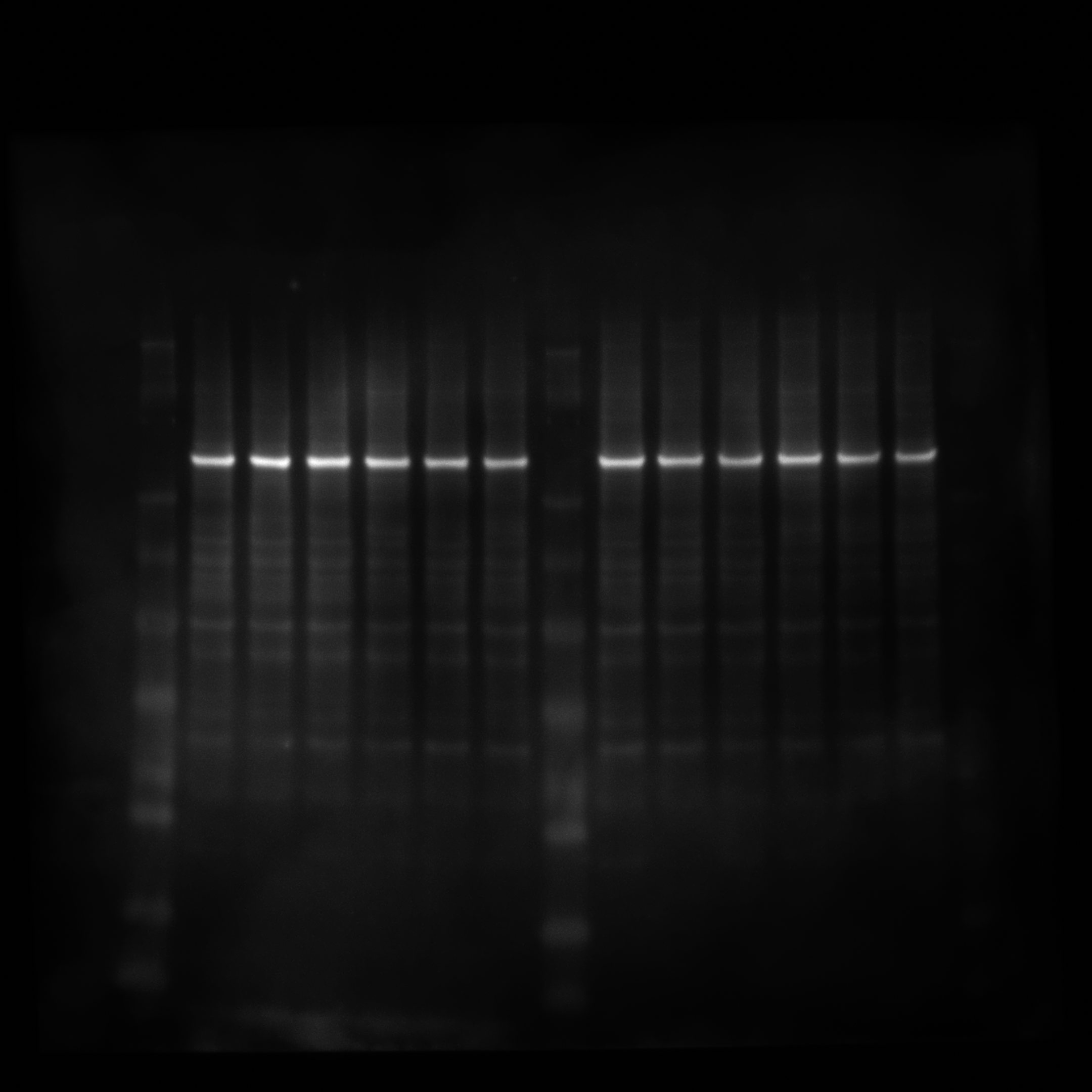

Supplement: Figure 4—figure supplement 1—source data 1. [file elife-106901-fig4-figsupp1-data1.zip › Figure4 figure supplement 1 source data 1/Figure S4E TBK1.Tif]

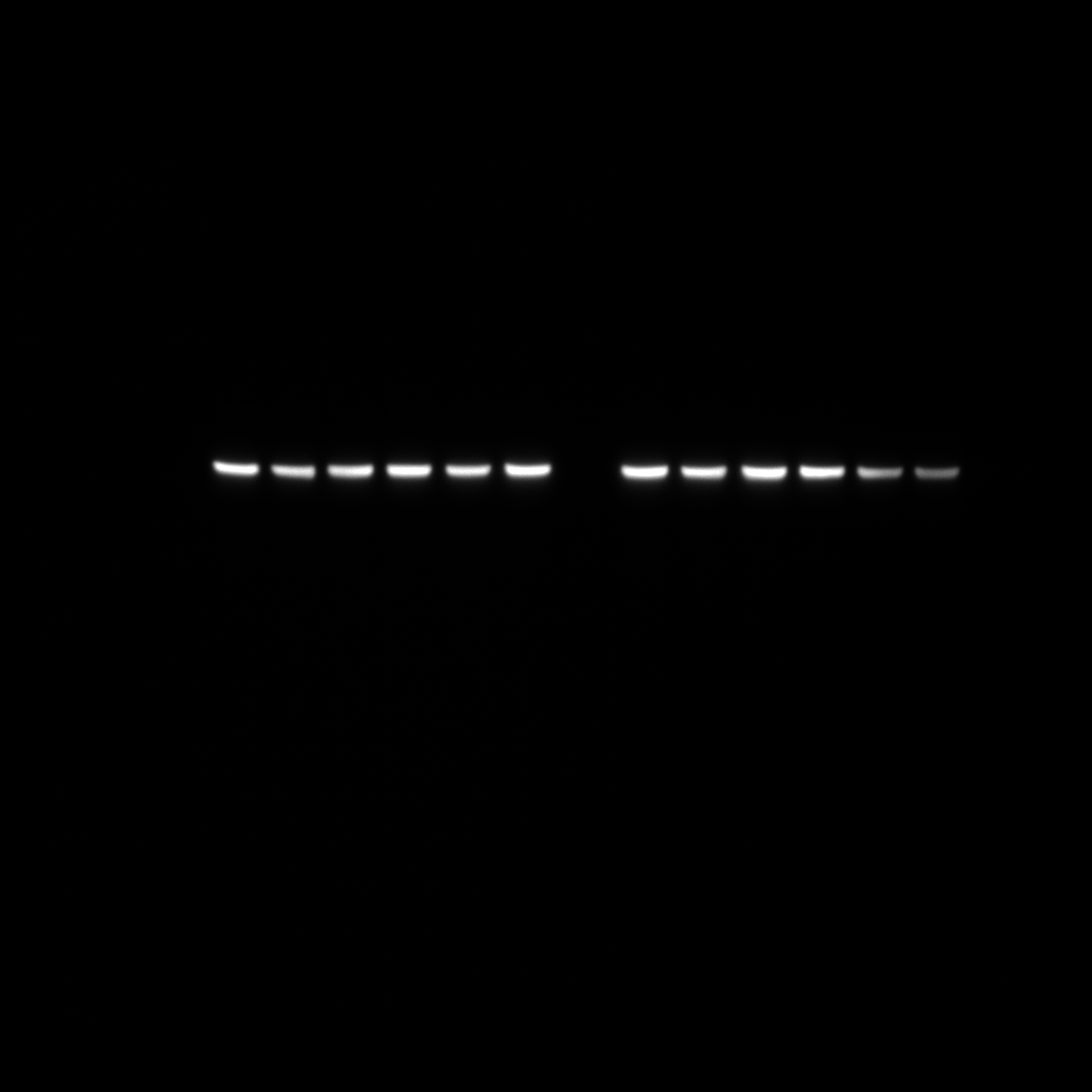

Supplement: Figure 4—figure supplement 1—source data 1. [file elife-106901-fig4-figsupp1-data1.zip › Figure4 figure supplement 1 source data 1/Figure S4E Tubulin.Tif]

Figure S4D

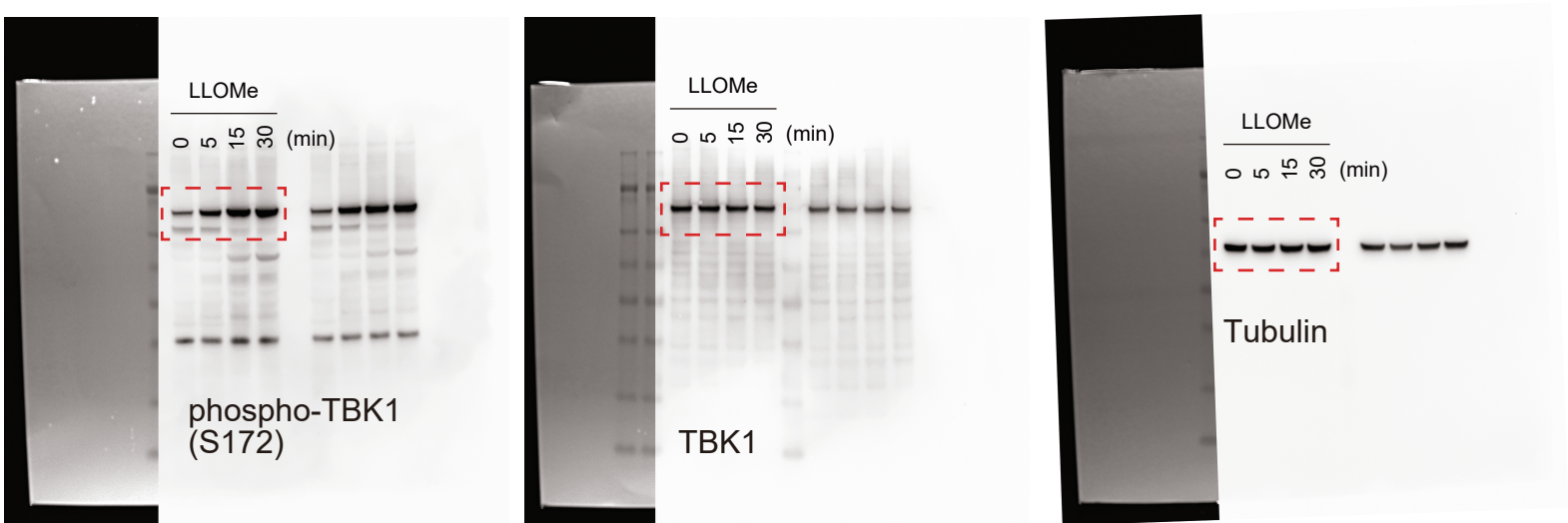

Figure S4E

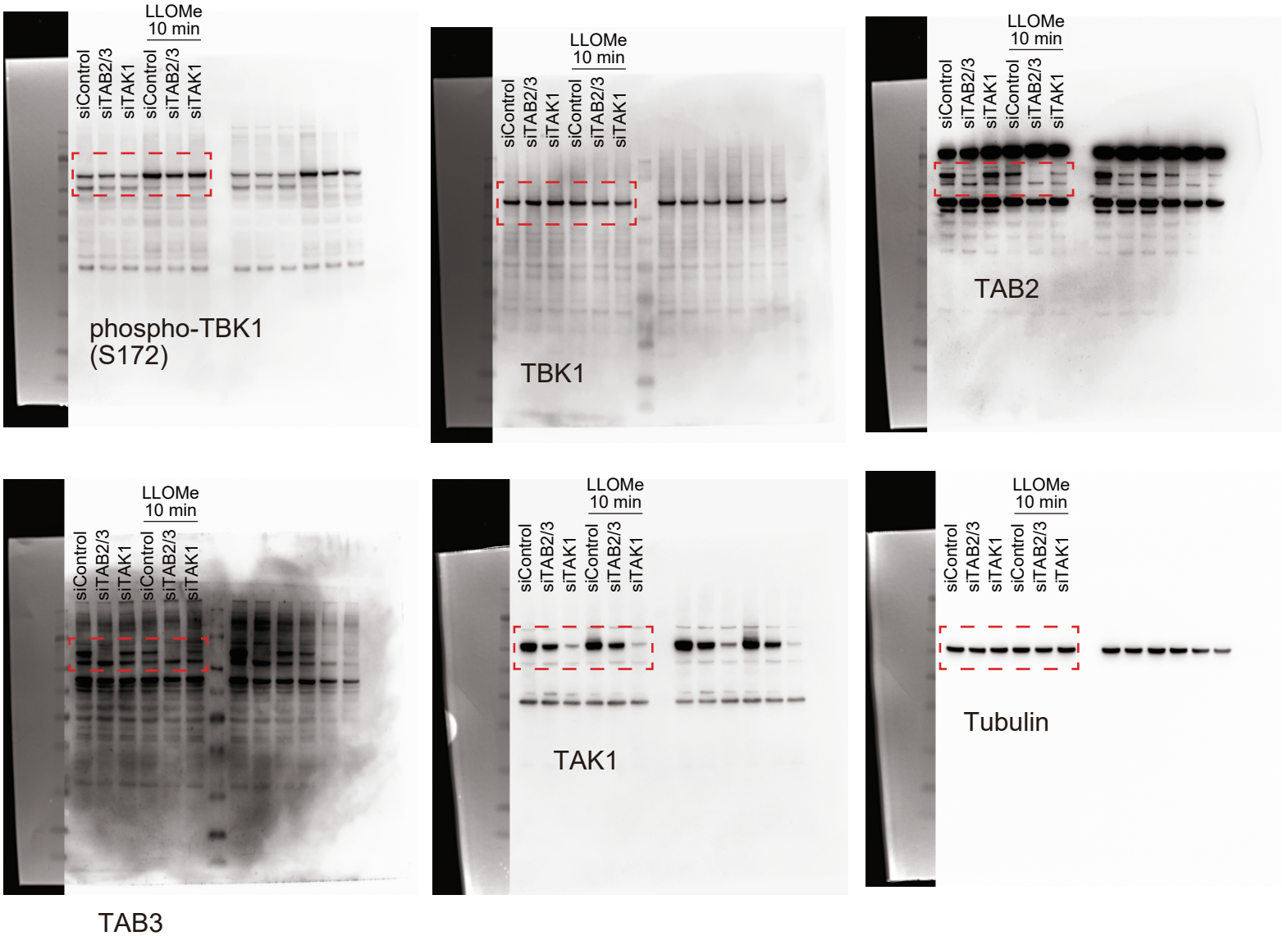

Supplement: Figure 4—figure supplement 1—source data 2. [file elife-106901-fig4-figsupp1-data2.pdf]

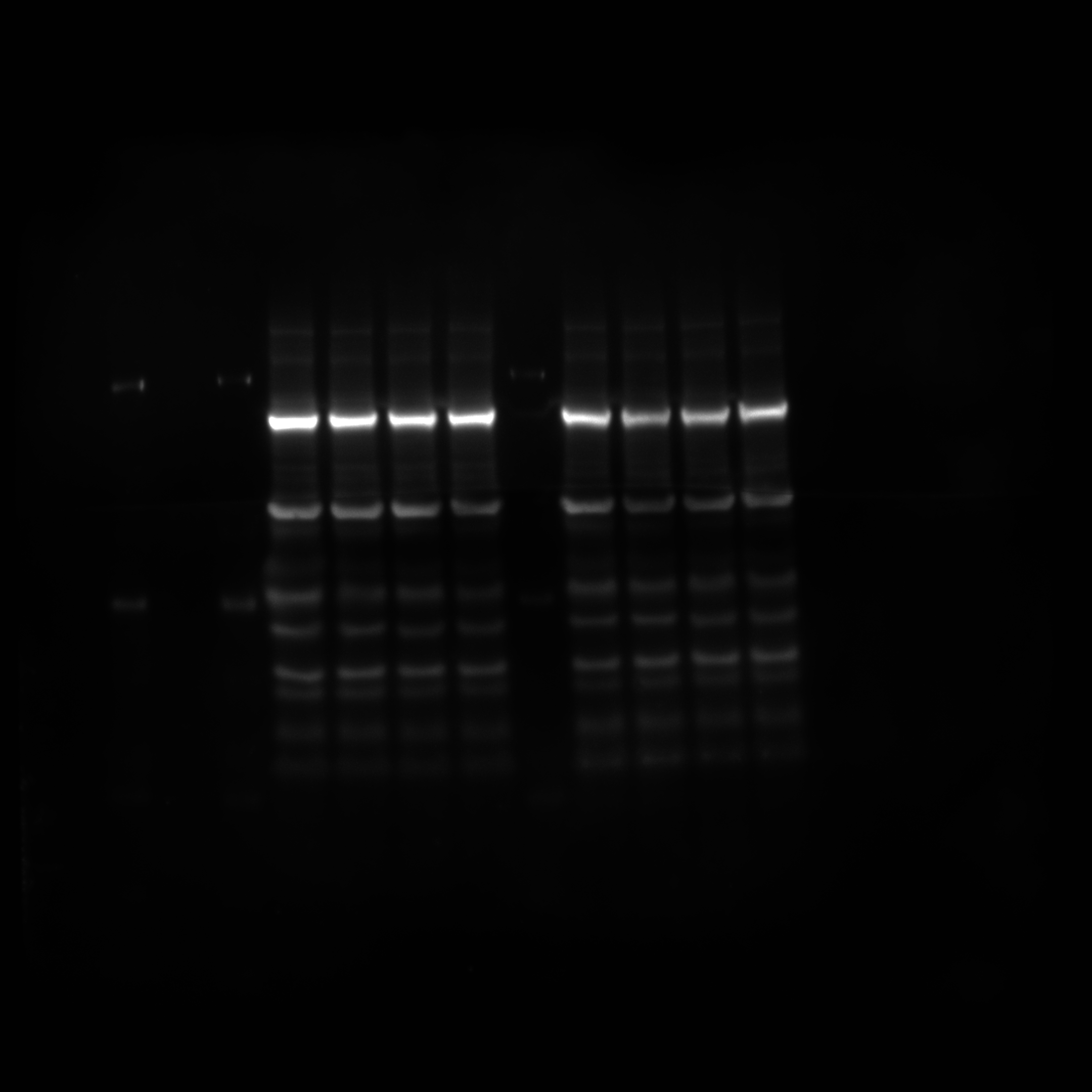

Supplement: Figure 4—figure supplement 1—source data 3. [file elife-106901-fig4-figsupp1-data3.zip › Figure4 figure supplement 1 source data 3/Figure S4F IkBa.Tif]

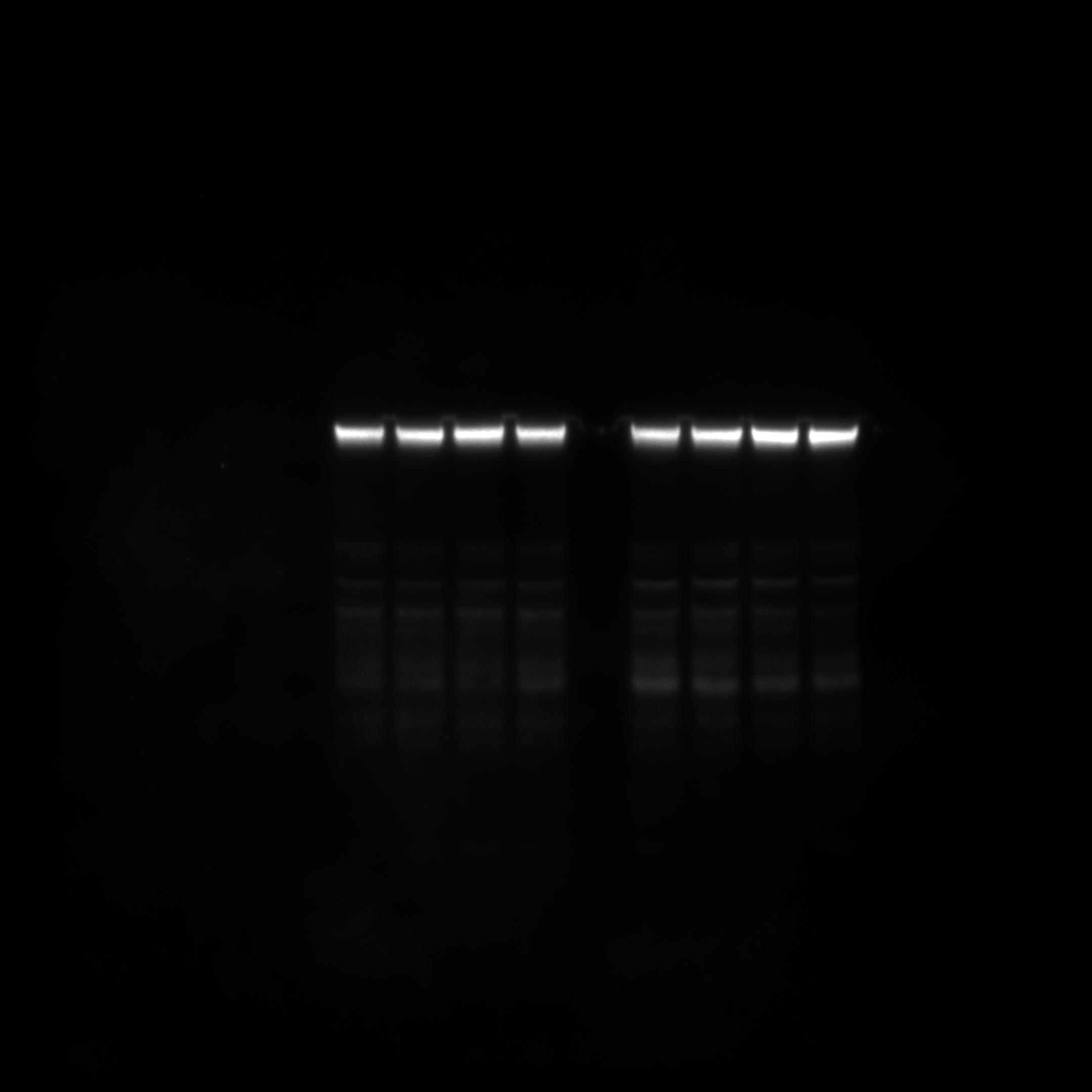

Supplement: Figure 4—figure supplement 1—source data 3. [file elife-106901-fig4-figsupp1-data3.zip › Figure4 figure supplement 1 source data 3/Figure S4F IKKa.Tif]

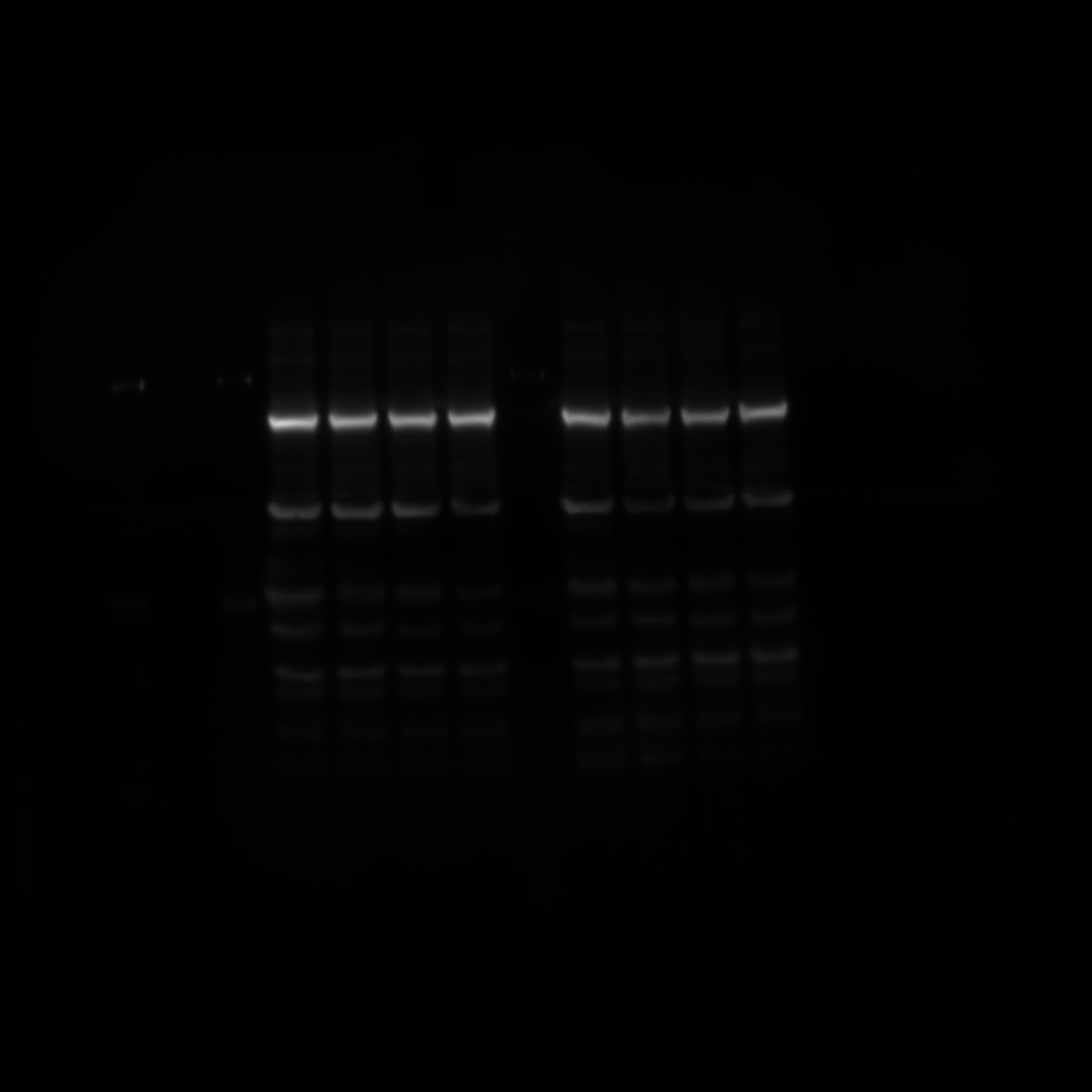

Supplement: Figure 4—figure supplement 1—source data 3. [file elife-106901-fig4-figsupp1-data3.zip › Figure4 figure supplement 1 source data 3/Figure S4F IKKb.Tif]

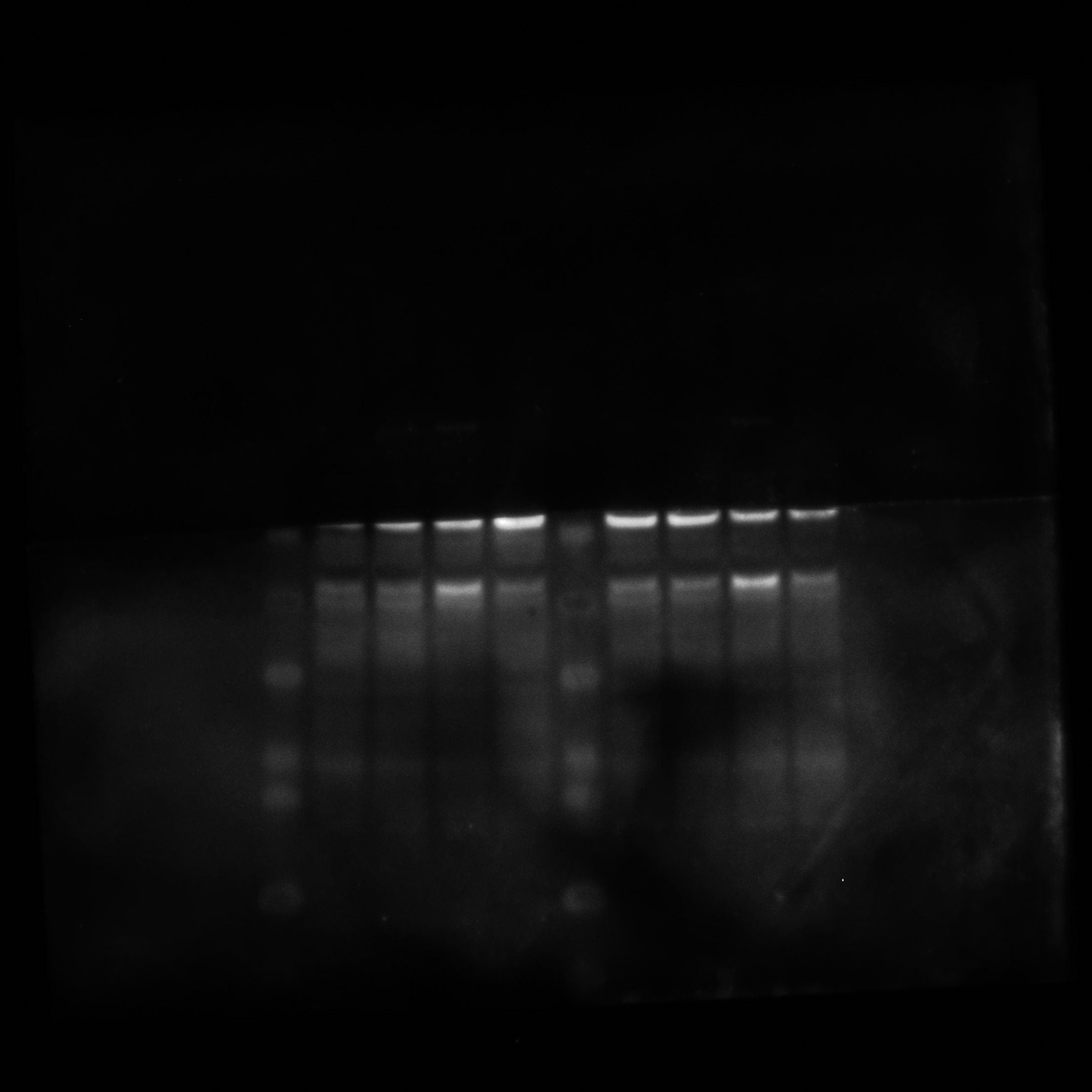

Supplement: Figure 4—figure supplement 1—source data 3. [file elife-106901-fig4-figsupp1-data3.zip › Figure4 figure supplement 1 source data 3/Figure S4F pIkBa.Tif]

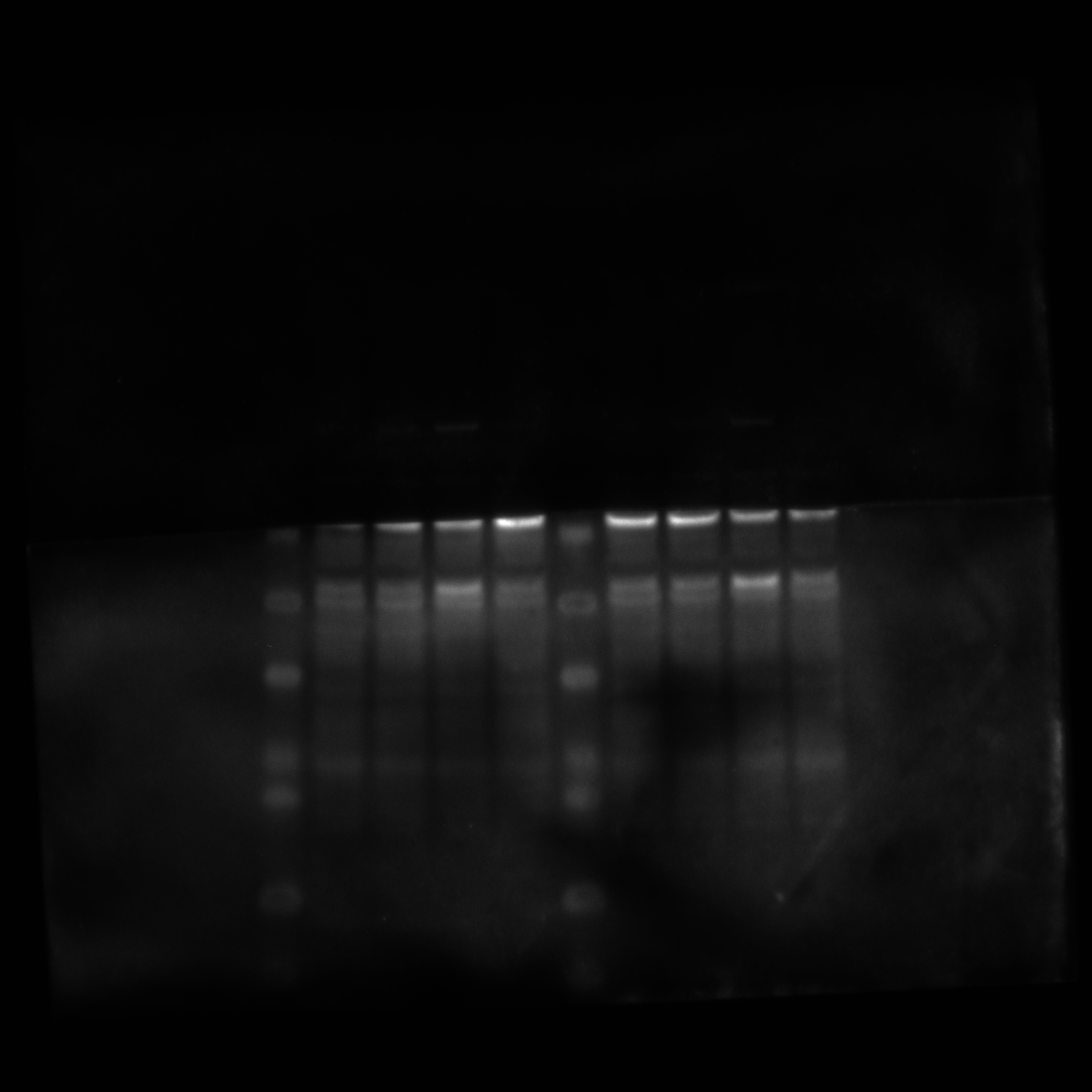

Supplement: Figure 4—figure supplement 1—source data 3. [file elife-106901-fig4-figsupp1-data3.zip › Figure4 figure supplement 1 source data 3/Figure S4F pIKKab.Tif]

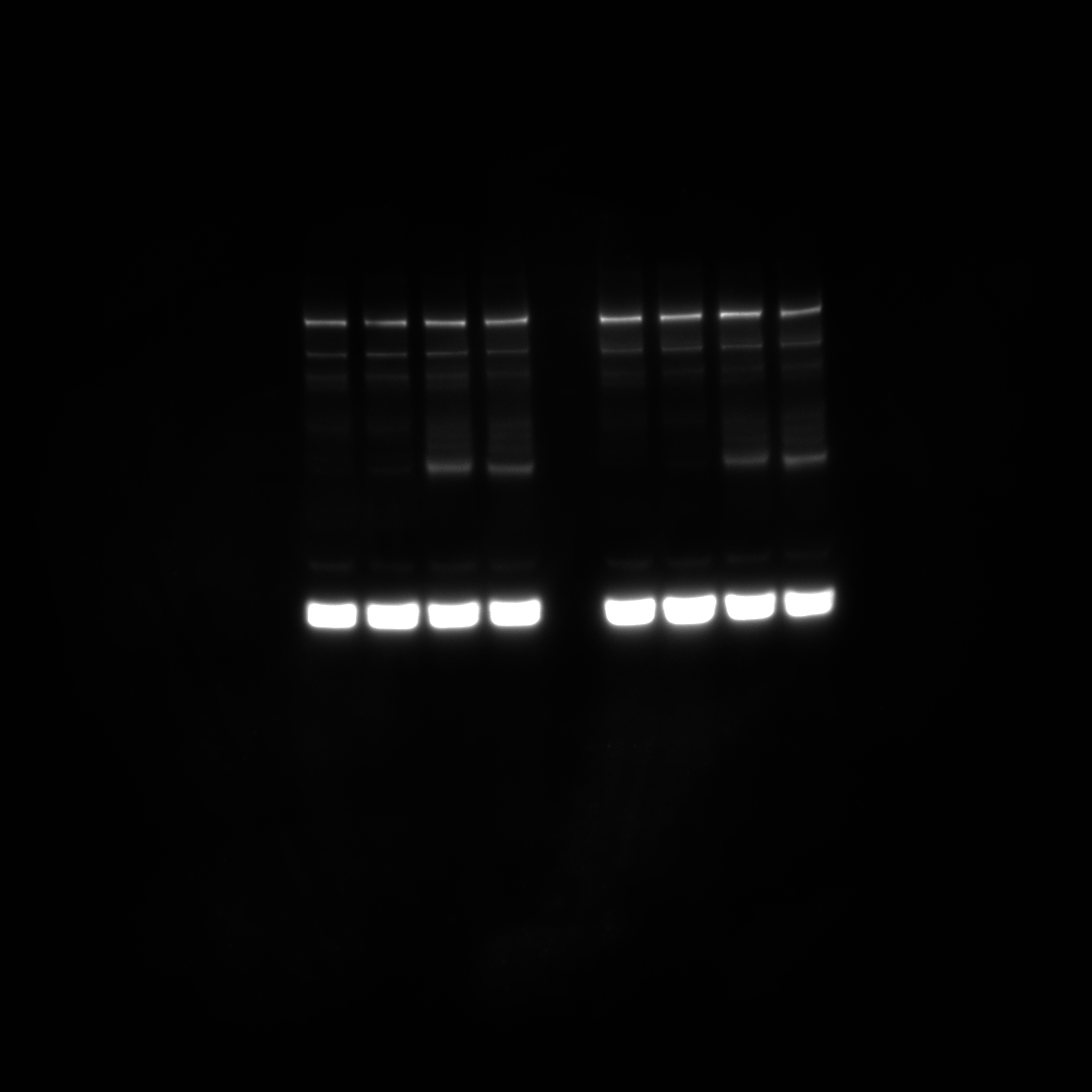

Supplement: Figure 4—figure supplement 1—source data 3. [file elife-106901-fig4-figsupp1-data3.zip › Figure4 figure supplement 1 source data 3/Figure S4F pTAK1.Tif]

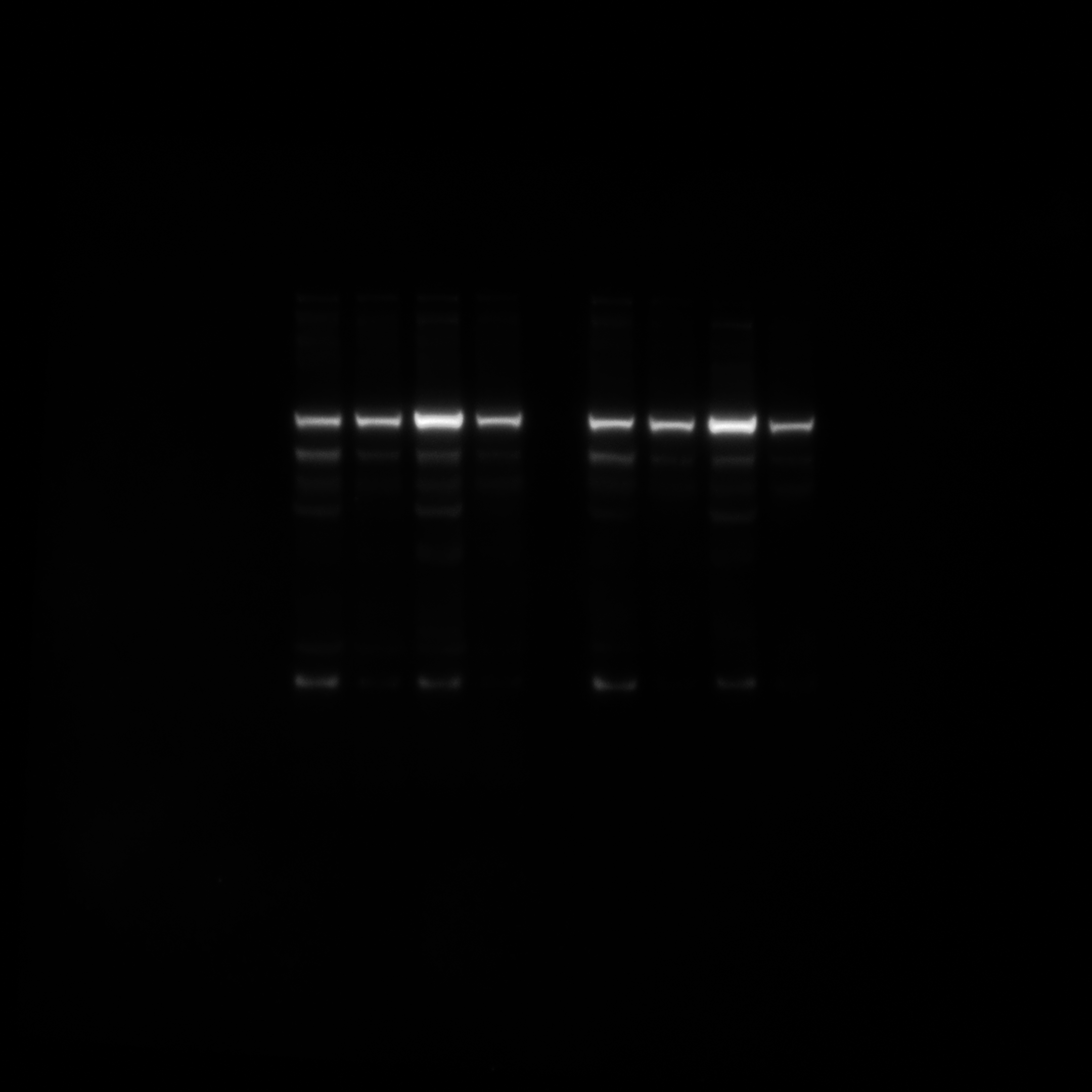

Supplement: Figure 4—figure supplement 1—source data 3. [file elife-106901-fig4-figsupp1-data3.zip › Figure4 figure supplement 1 source data 3/Figure S4F pTBK1.Tif]

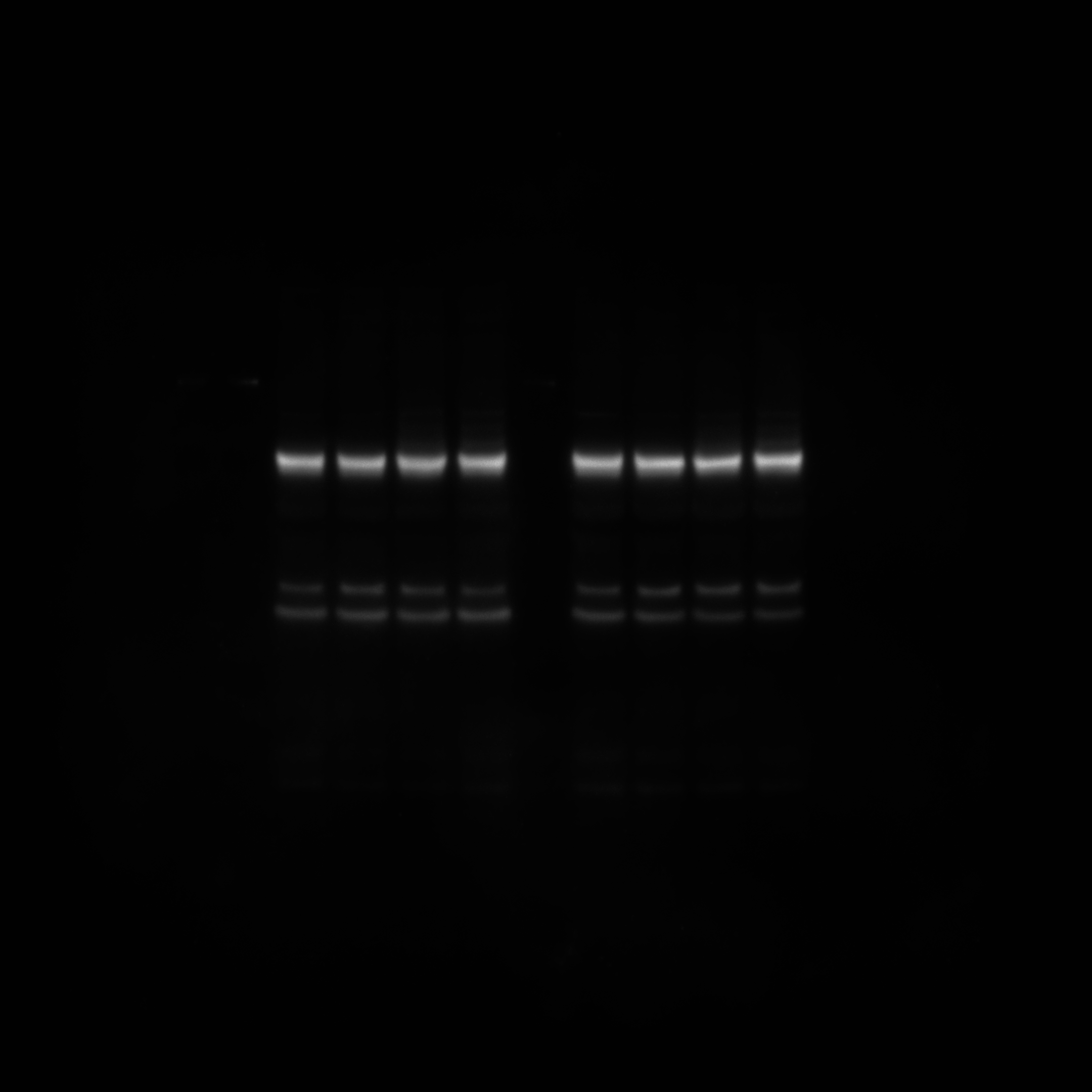

Supplement: Figure 4—figure supplement 1—source data 3. [file elife-106901-fig4-figsupp1-data3.zip › Figure4 figure supplement 1 source data 3/Figure S4F TAK1.Tif]

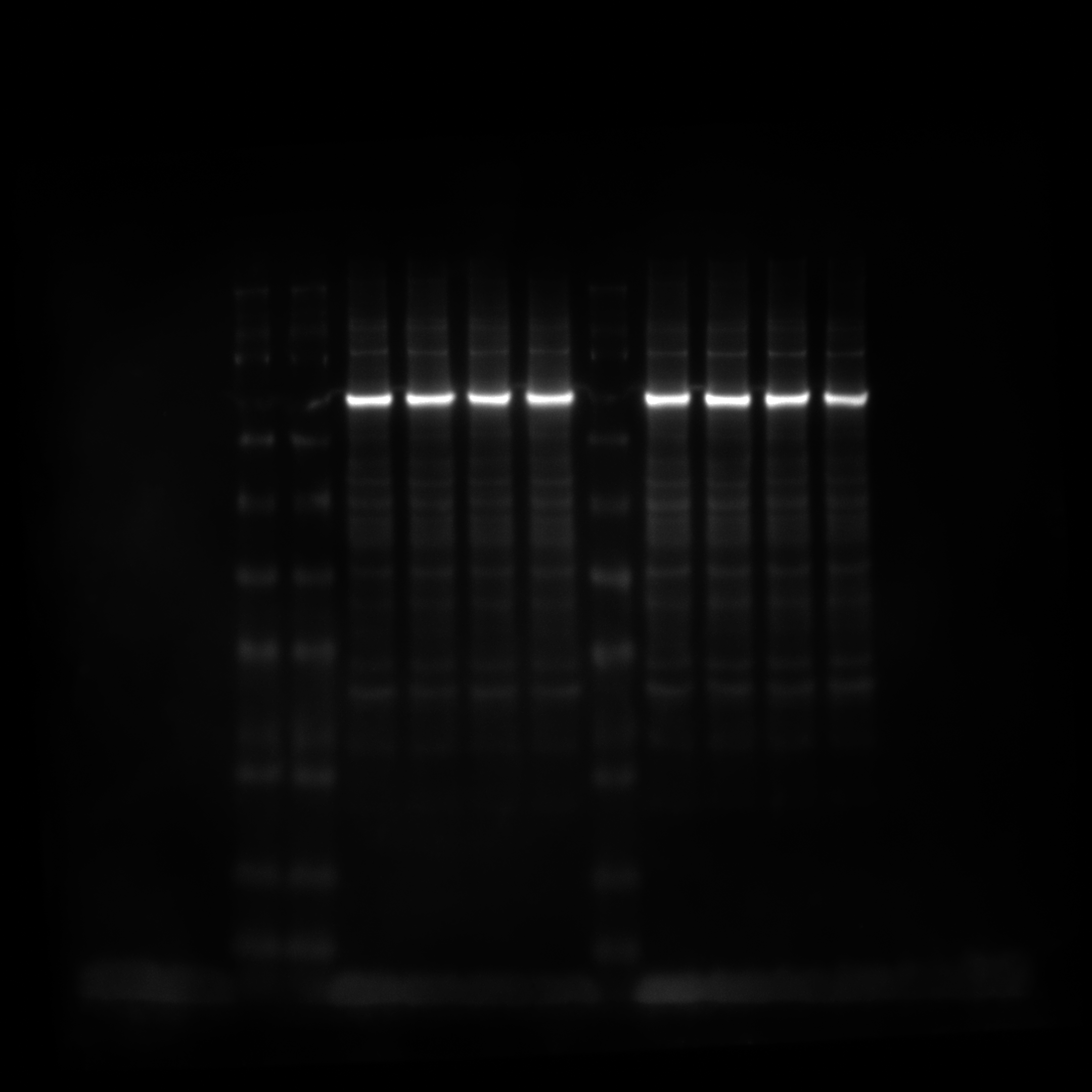

Supplement: Figure 4—figure supplement 1—source data 3. [file elife-106901-fig4-figsupp1-data3.zip › Figure4 figure supplement 1 source data 3/Figure S4F TBK1.Tif]

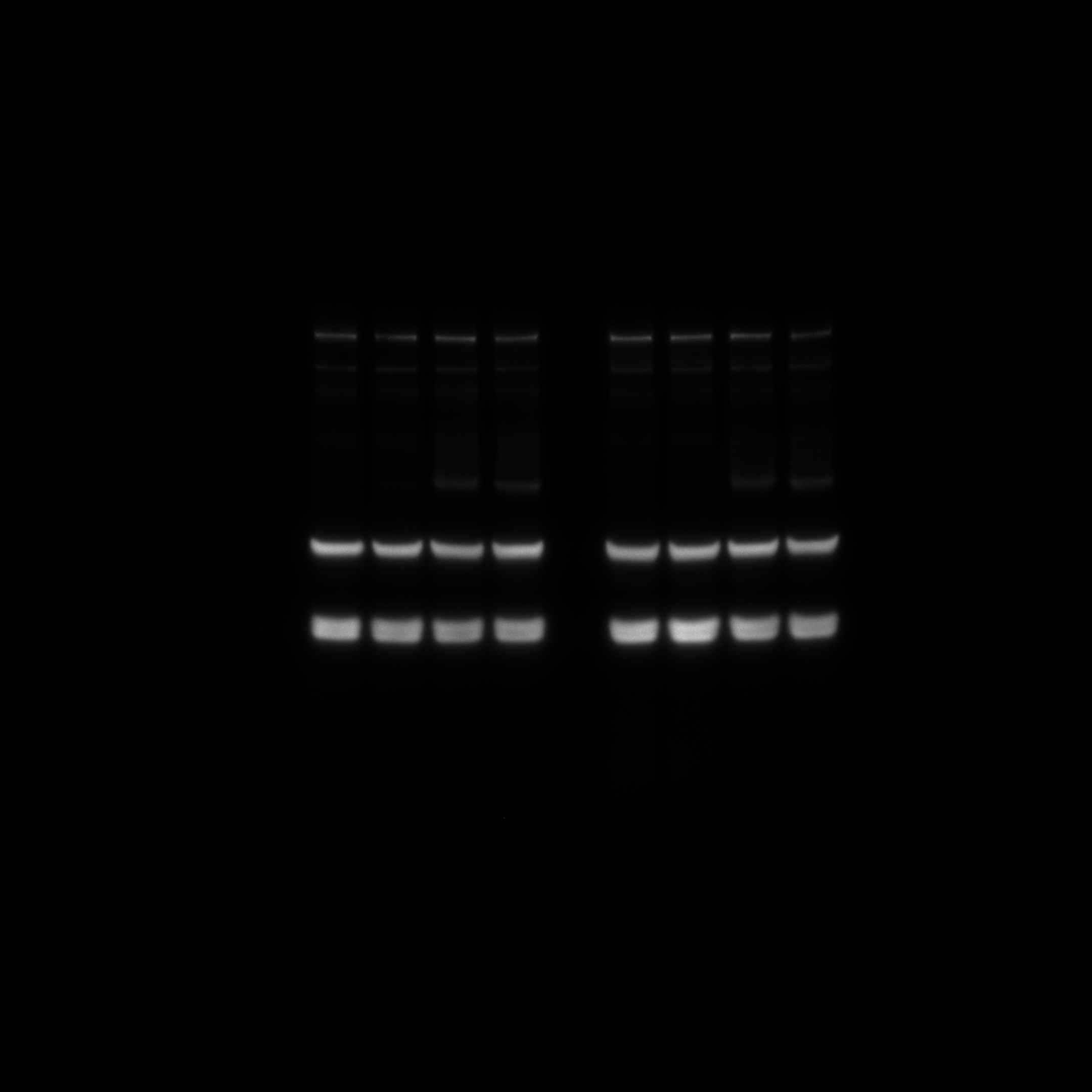

Supplement: Figure 4—figure supplement 1—source data 3. [file elife-106901-fig4-figsupp1-data3.zip › Figure4 figure supplement 1 source data 3/Figure S4F Tubulin.Tif]

Figure S4F

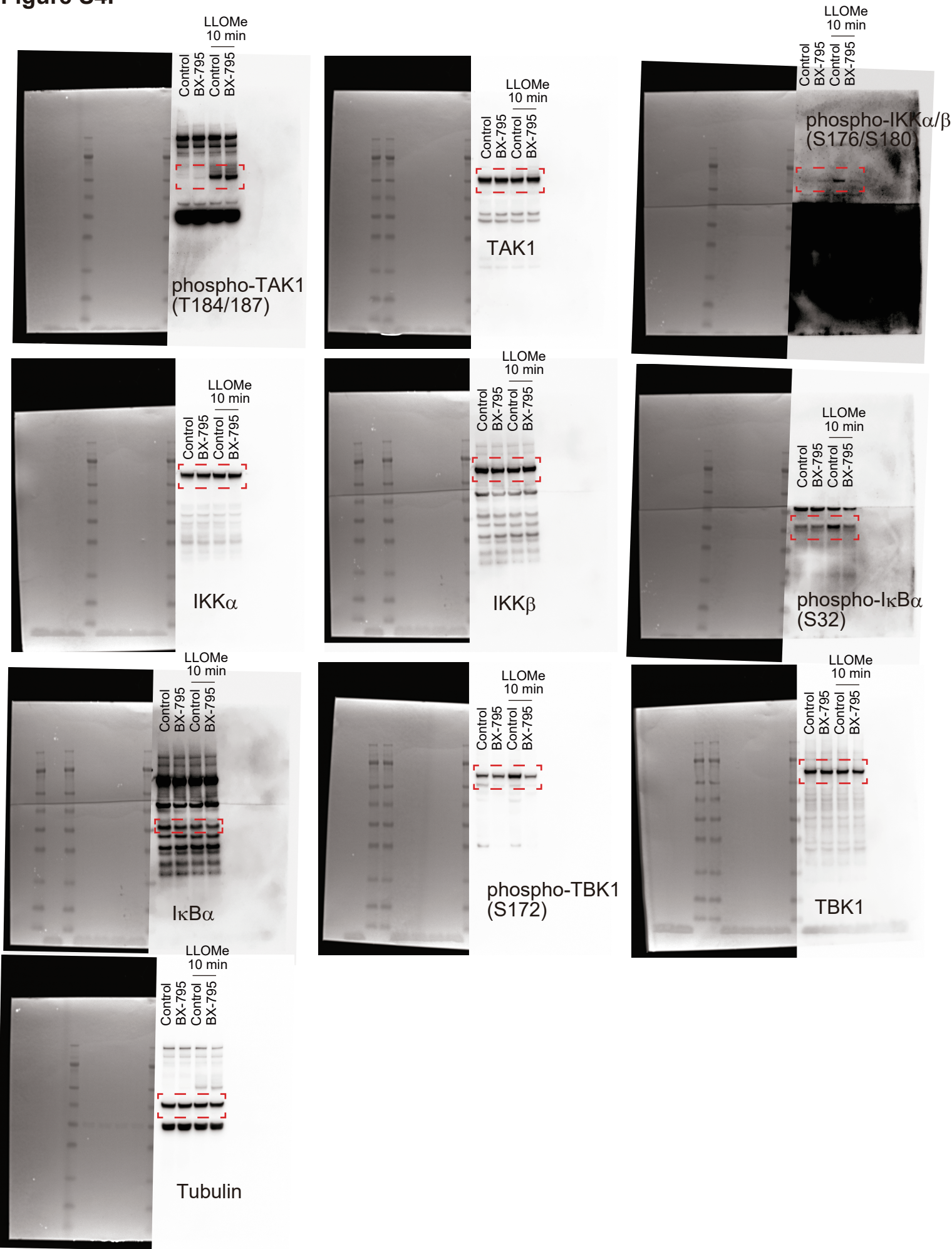

Supplement: Figure 4—figure supplement 1—source data 4. [file elife-106901-fig4-figsupp1-data4.pdf]

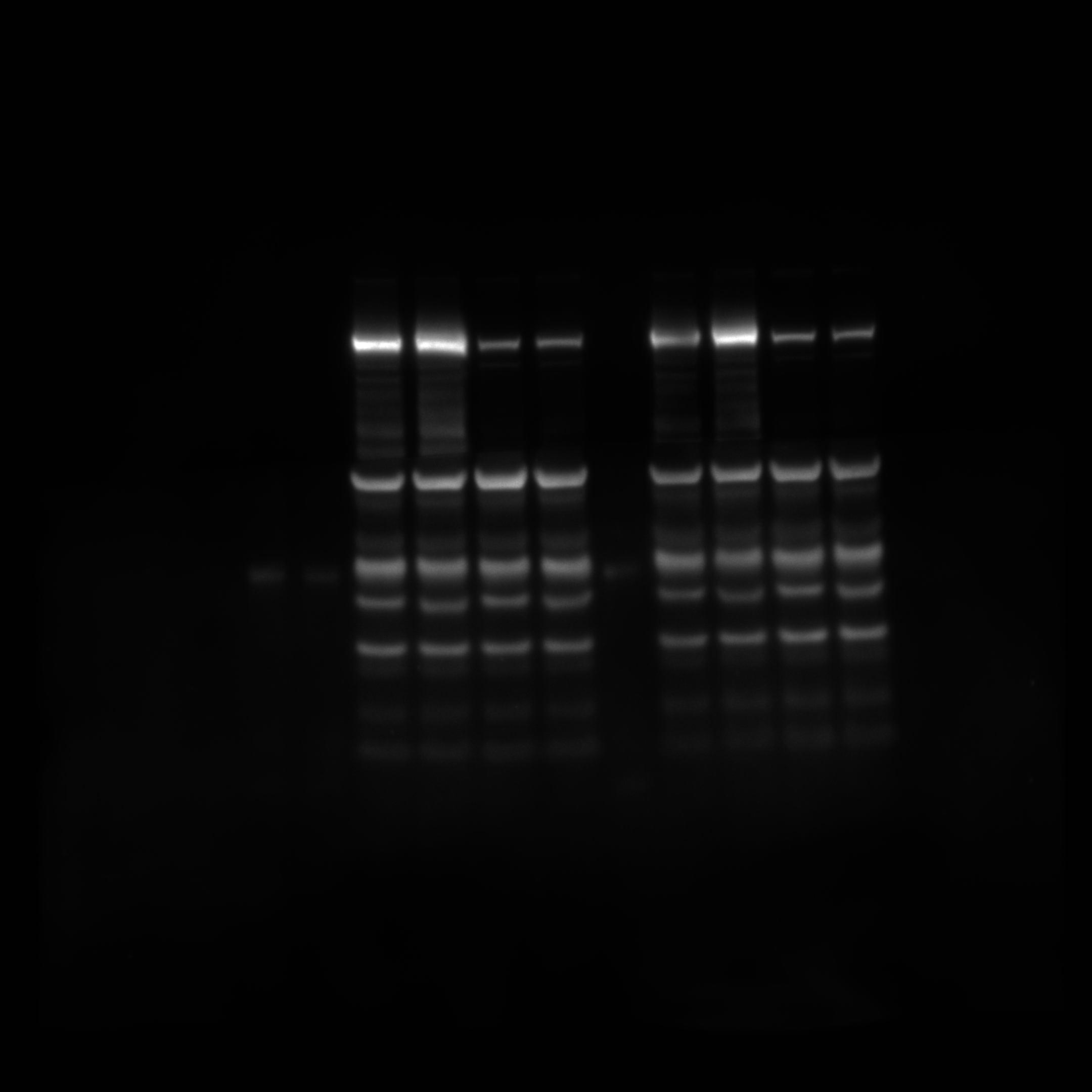

Supplement: Figure 4—figure supplement 1—source data 5. [file elife-106901-fig4-figsupp1-data5.zip › Figure4 figure supplement 1 source data 5/Figure S4G IkBa.Tif]

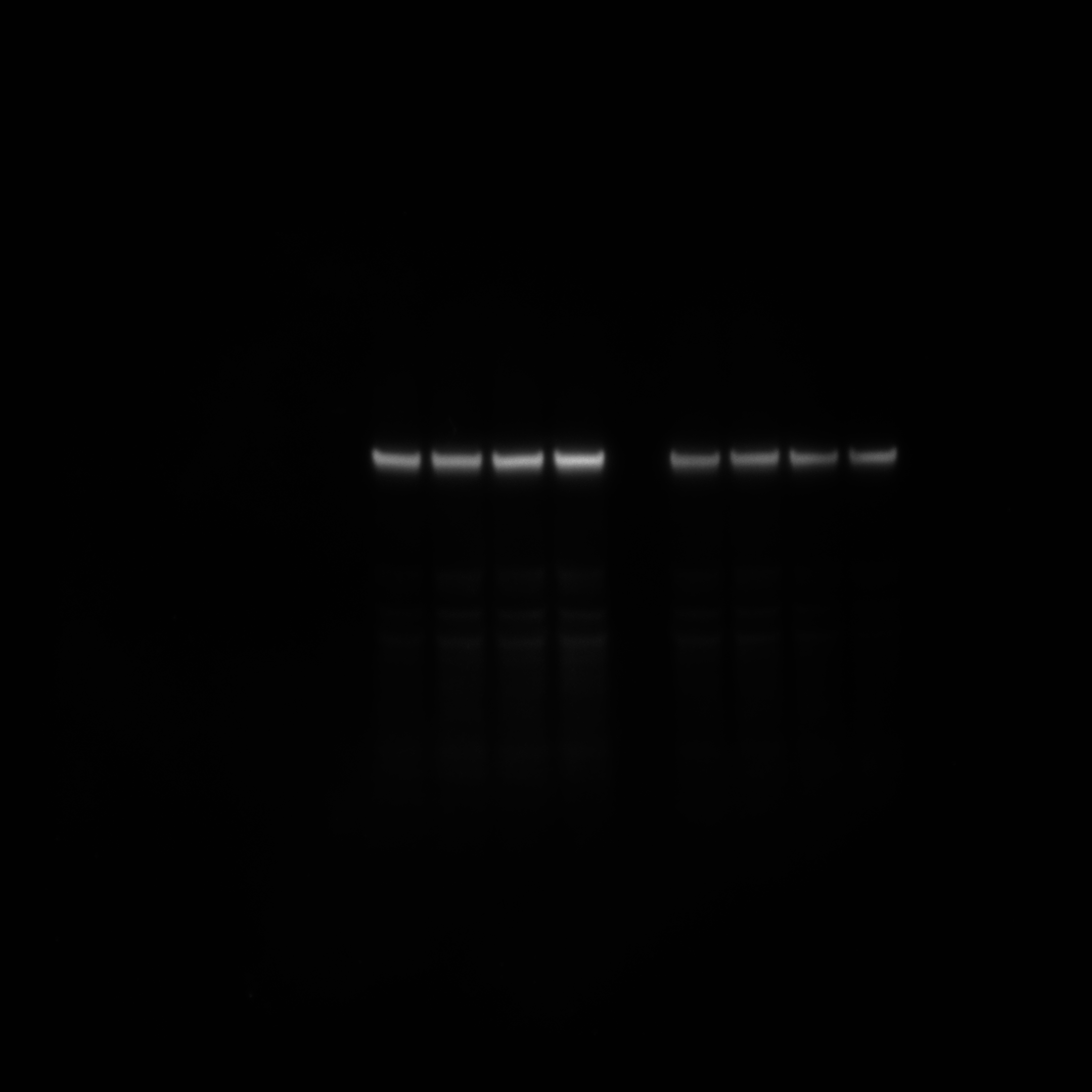

Supplement: Figure 4—figure supplement 1—source data 5. [file elife-106901-fig4-figsupp1-data5.zip › Figure4 figure supplement 1 source data 5/Figure S4G IKKa.Tif]

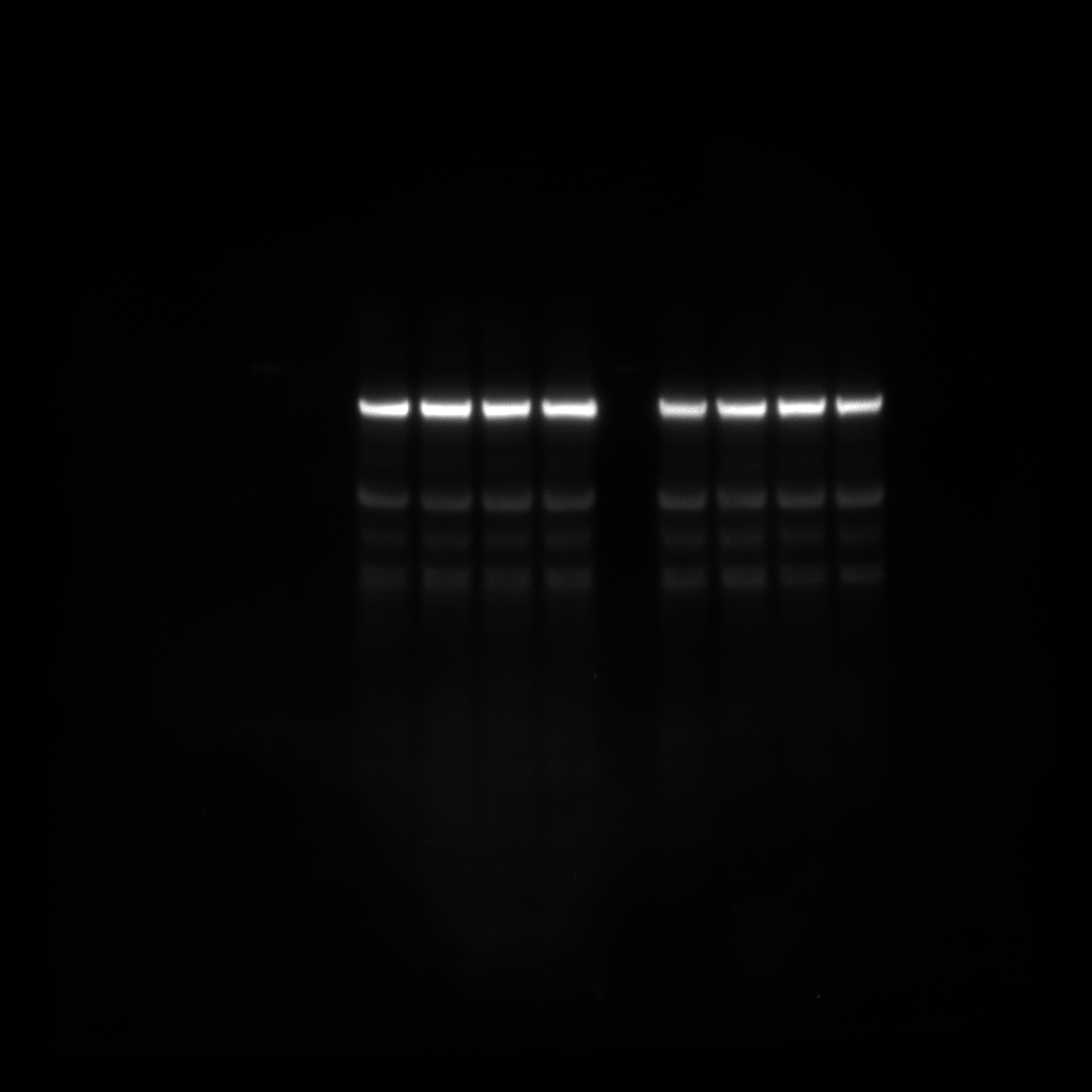

Supplement: Figure 4—figure supplement 1—source data 5. [file elife-106901-fig4-figsupp1-data5.zip › Figure4 figure supplement 1 source data 5/Figure S4G IKKb.Tif]

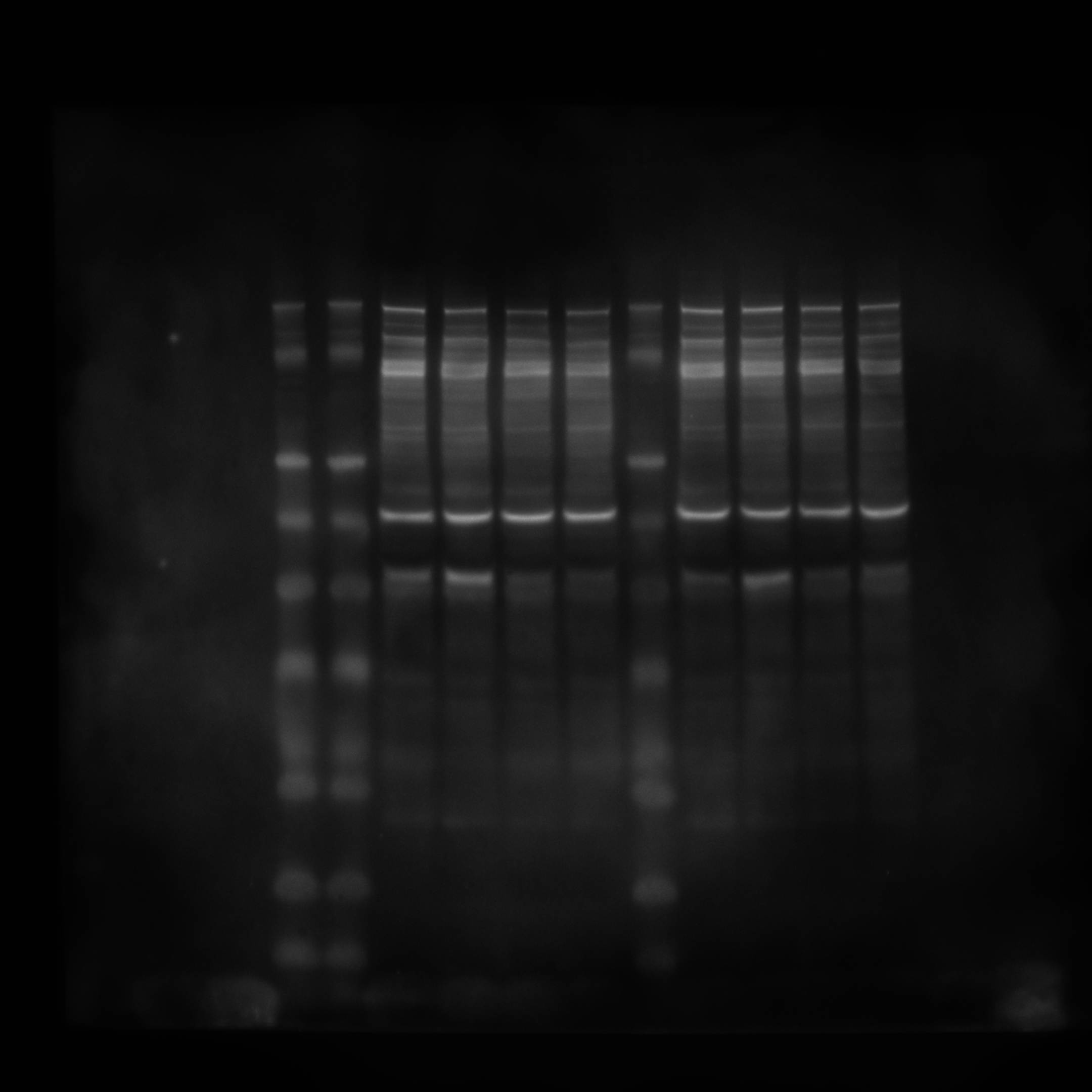

Supplement: Figure 4—figure supplement 1—source data 5. [file elife-106901-fig4-figsupp1-data5.zip › Figure4 figure supplement 1 source data 5/Figure S4G pIkBa.Tif]

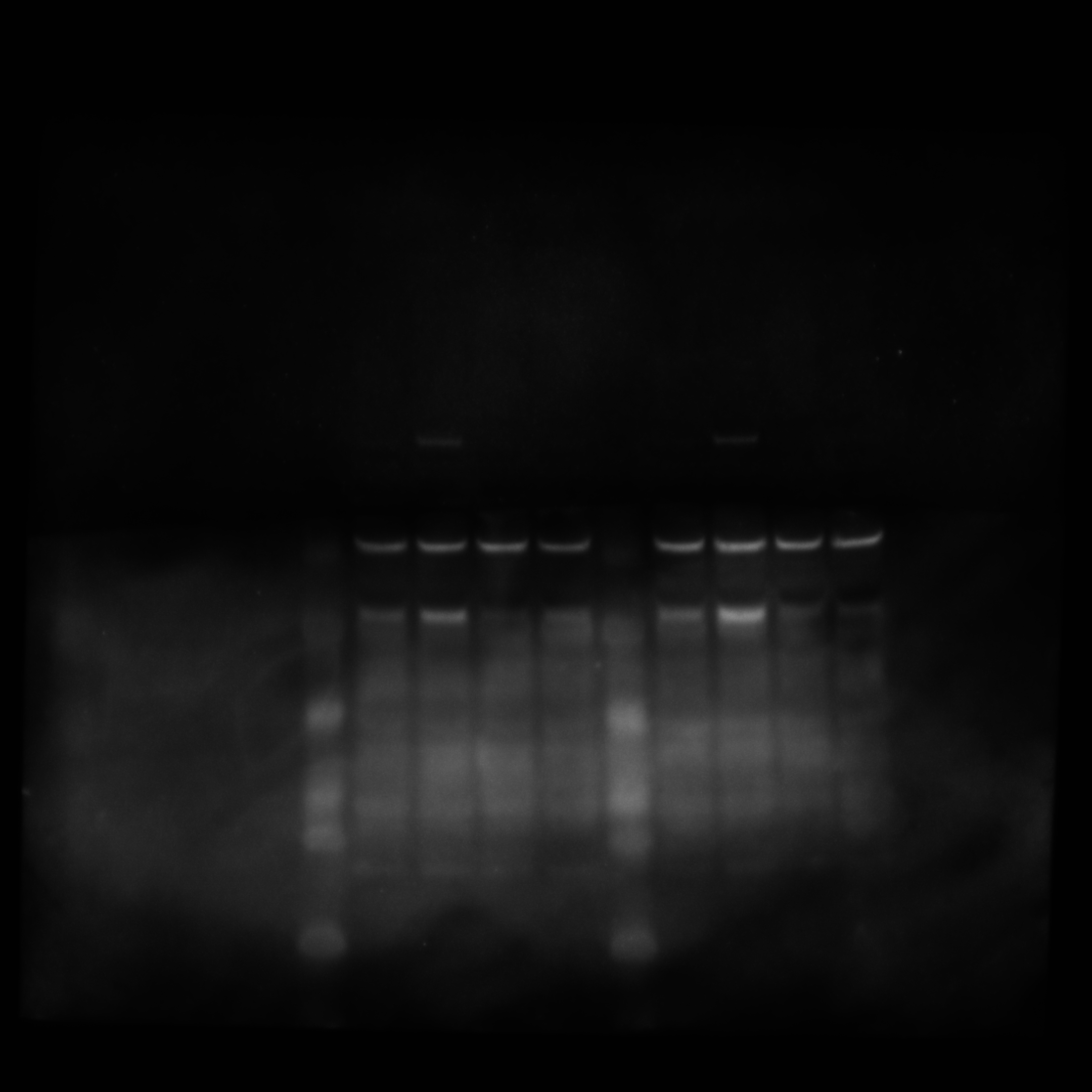

Supplement: Figure 4—figure supplement 1—source data 5. [file elife-106901-fig4-figsupp1-data5.zip › Figure4 figure supplement 1 source data 5/Figure S4G pIKKab.Tif]

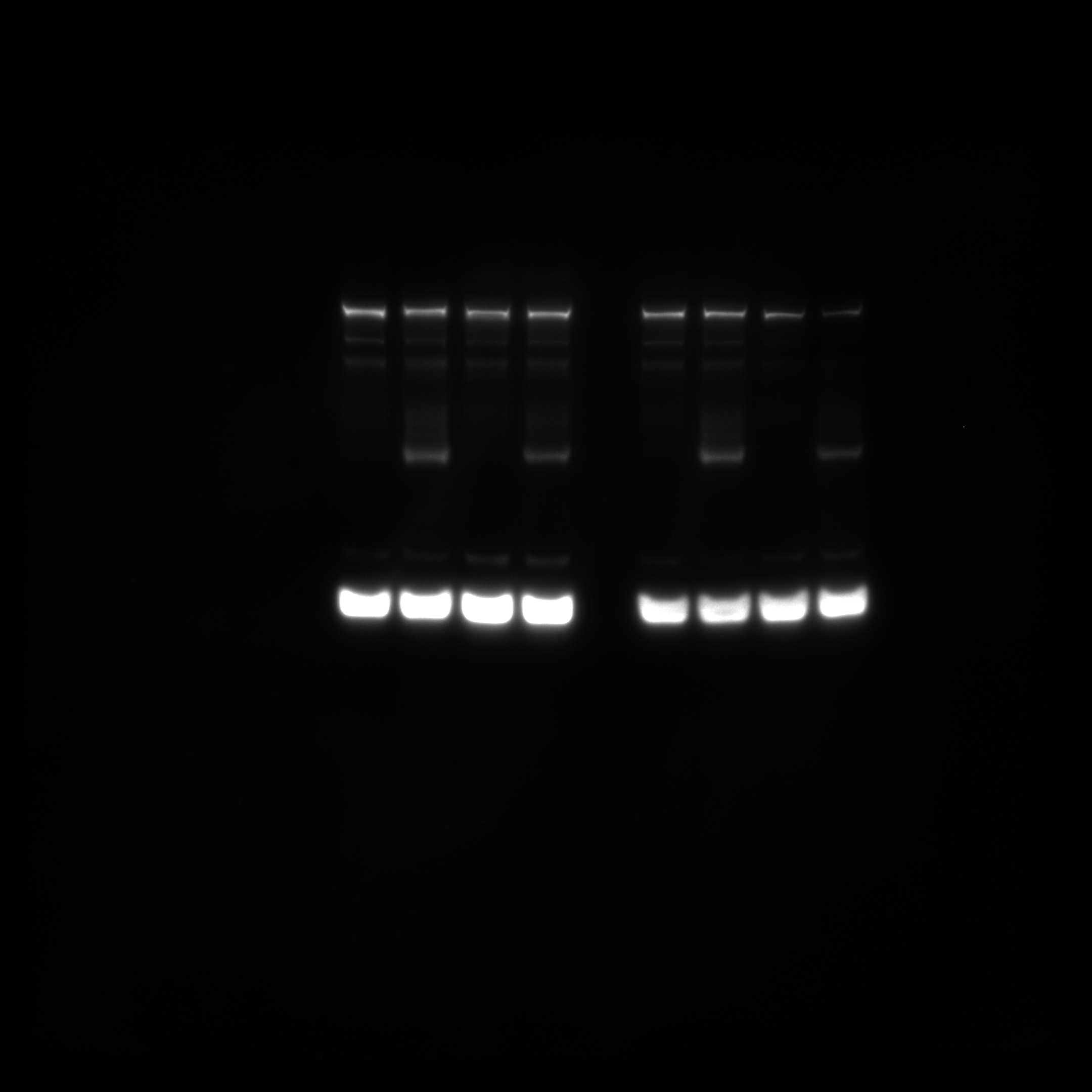

Supplement: Figure 4—figure supplement 1—source data 5. [file elife-106901-fig4-figsupp1-data5.zip › Figure4 figure supplement 1 source data 5/Figure S4G pTAK1.Tif]

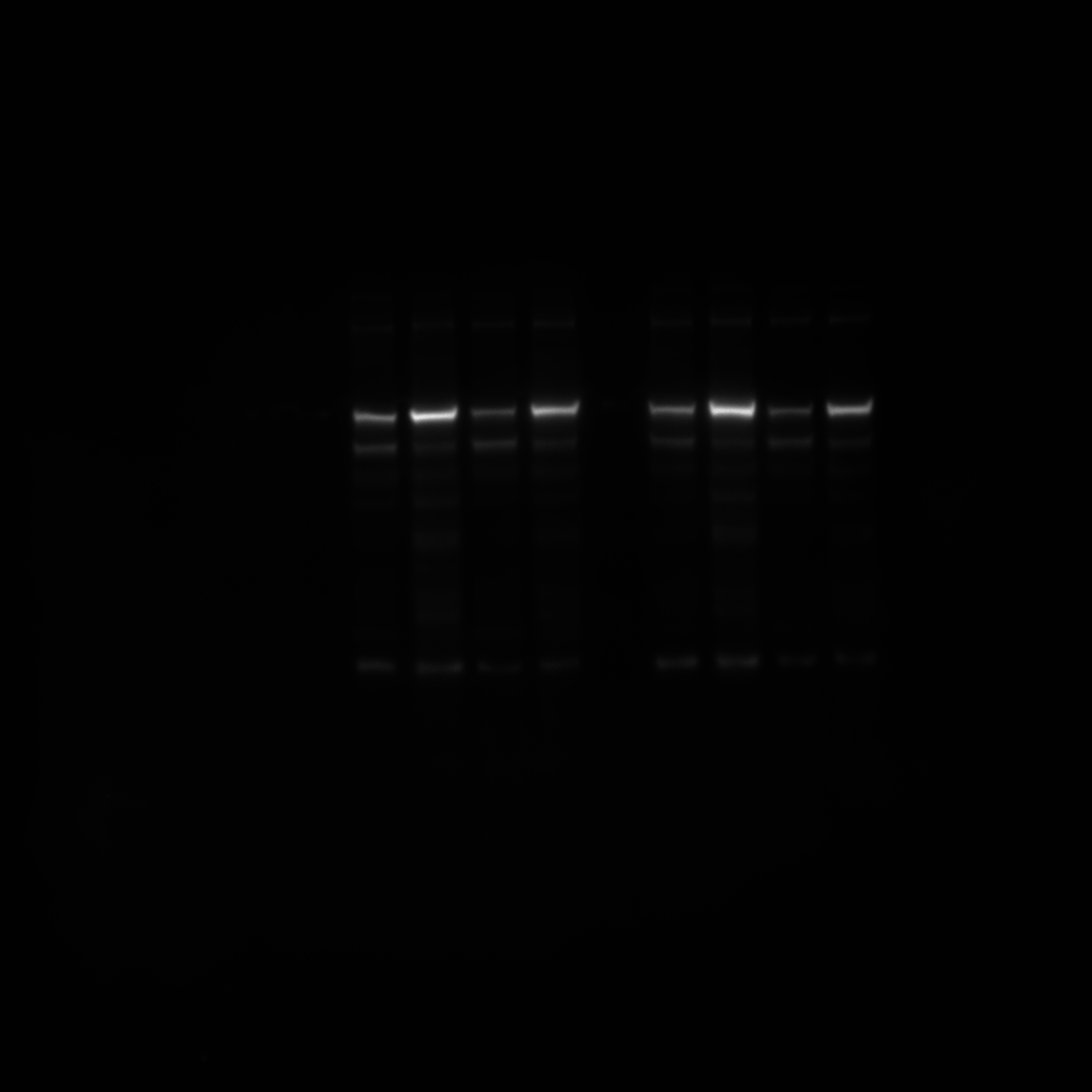

Supplement: Figure 4—figure supplement 1—source data 5. [file elife-106901-fig4-figsupp1-data5.zip › Figure4 figure supplement 1 source data 5/Figure S4G pTBK1.Tif]

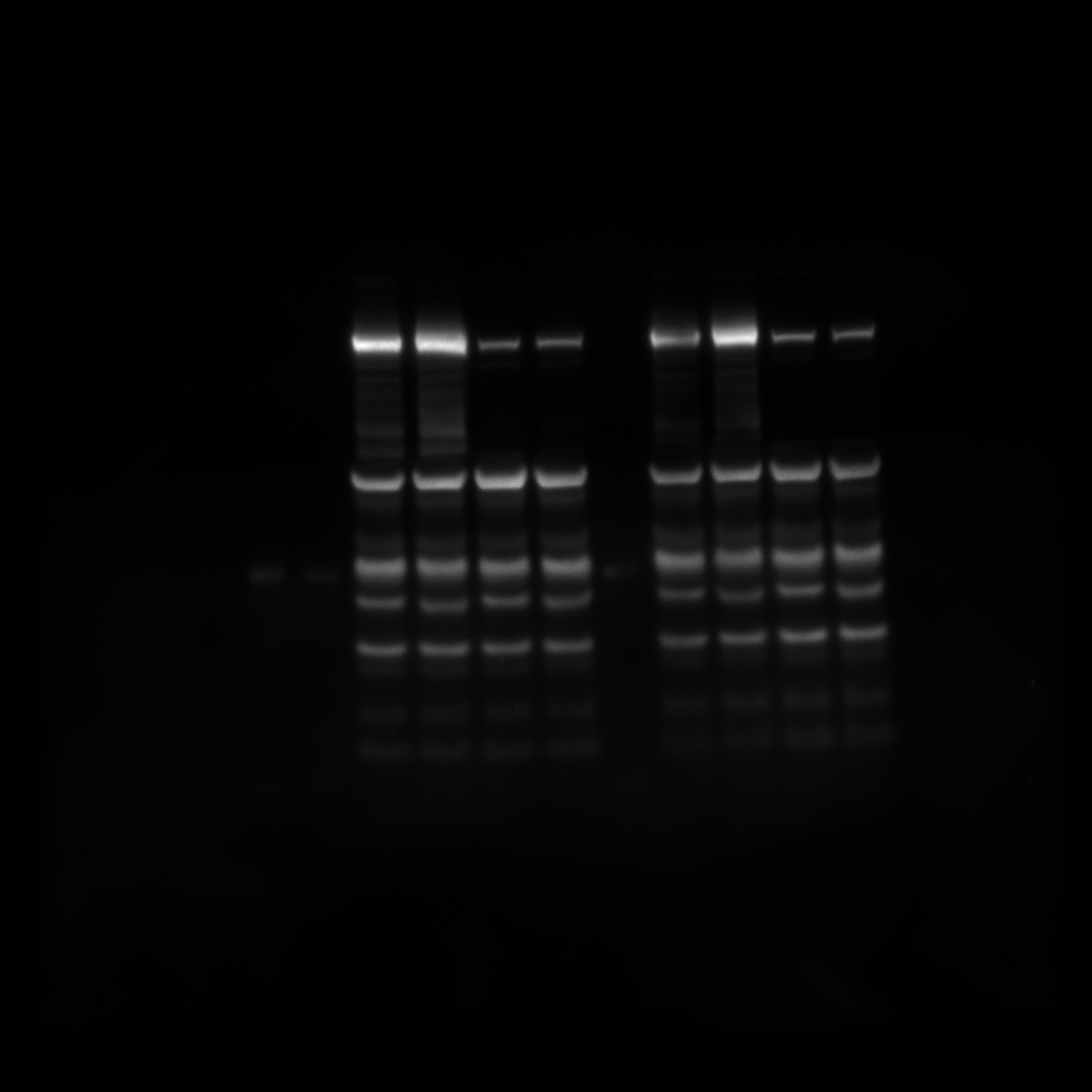

Supplement: Figure 4—figure supplement 1—source data 5. [file elife-106901-fig4-figsupp1-data5.zip › Figure4 figure supplement 1 source data 5/Figure S4G RNF31.Tif]

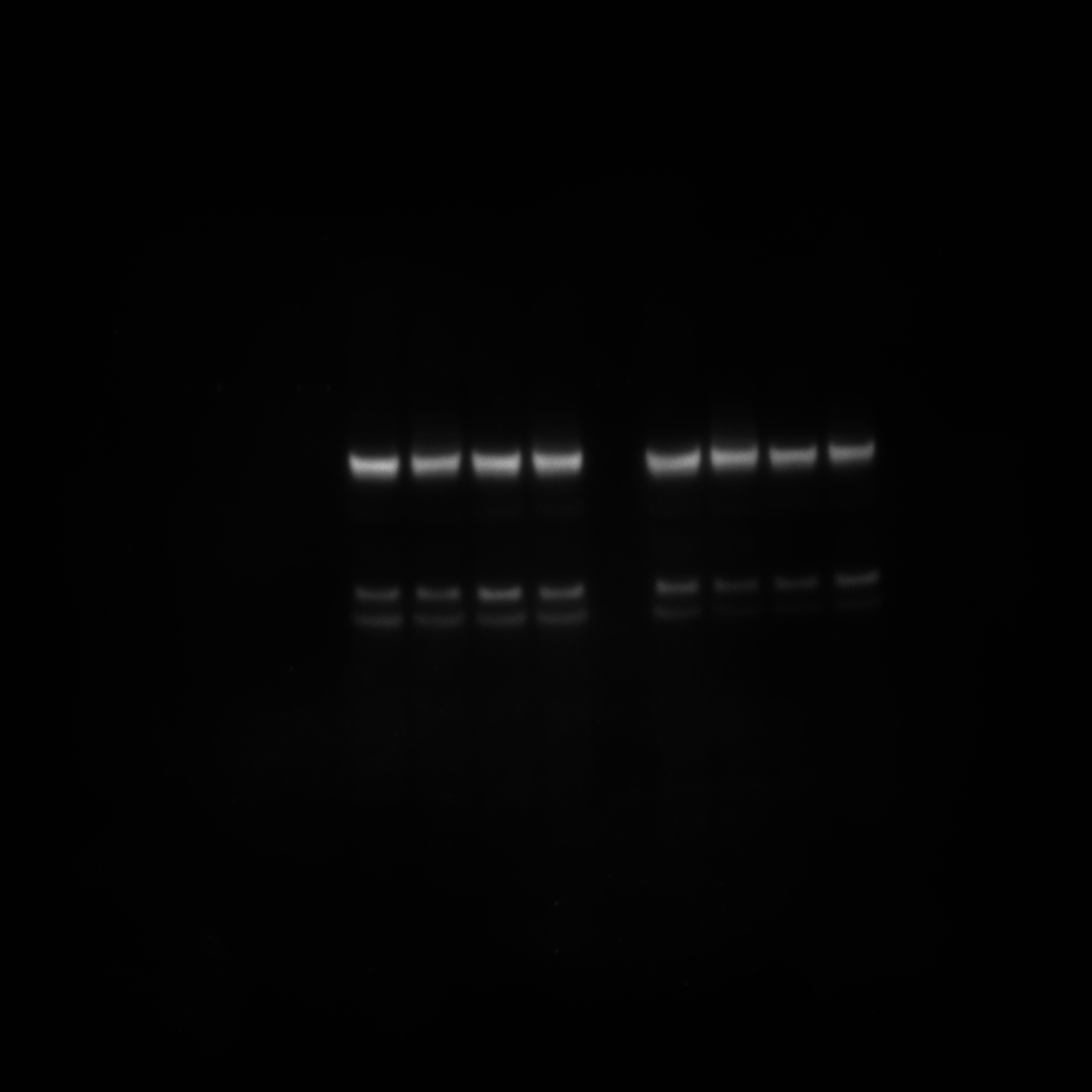

Supplement: Figure 4—figure supplement 1—source data 5. [file elife-106901-fig4-figsupp1-data5.zip › Figure4 figure supplement 1 source data 5/Figure S4G TAK1.Tif]

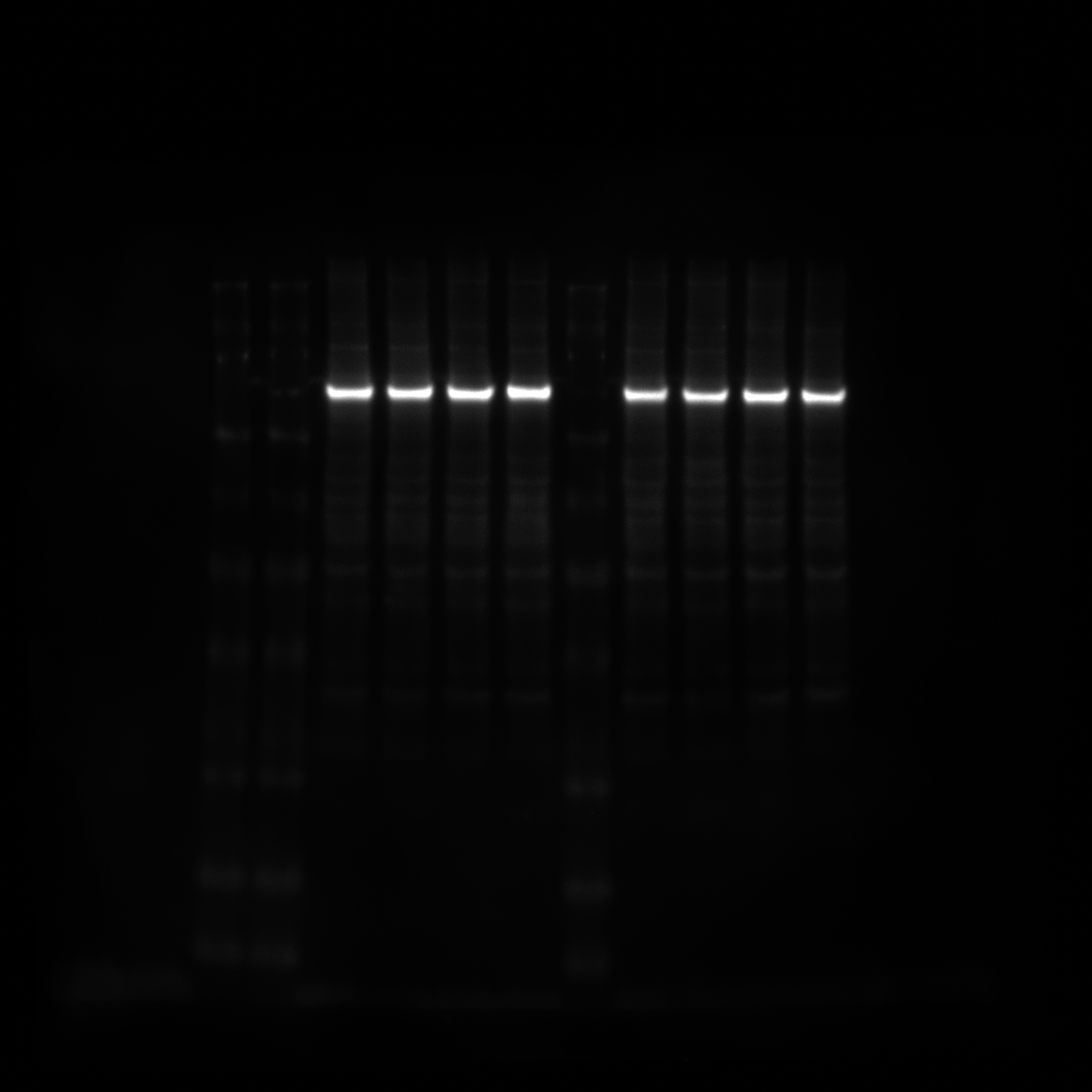

Supplement: Figure 4—figure supplement 1—source data 5. [file elife-106901-fig4-figsupp1-data5.zip › Figure4 figure supplement 1 source data 5/Figure S4G TBK1.Tif]

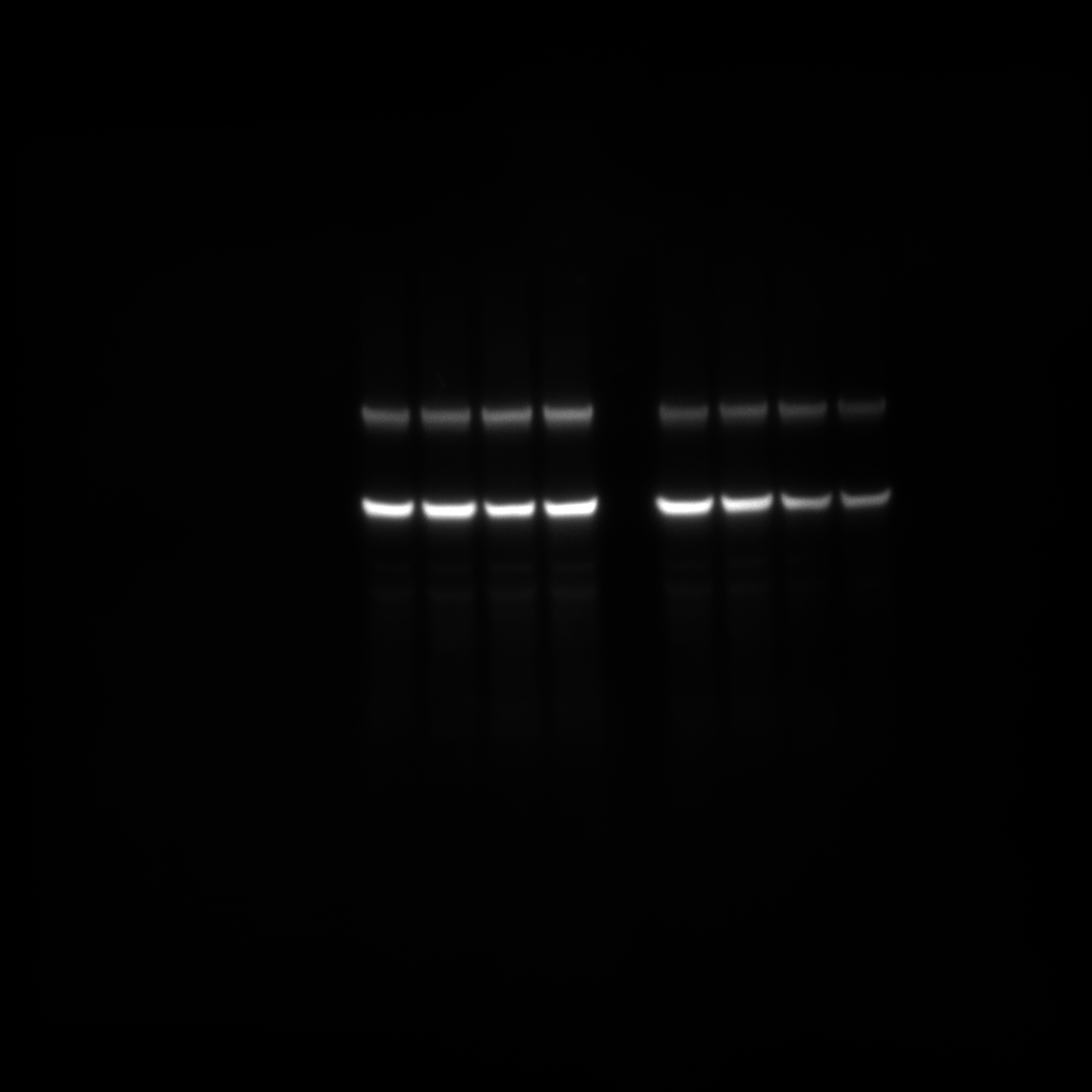

Supplement: Figure 4—figure supplement 1—source data 5. [file elife-106901-fig4-figsupp1-data5.zip › Figure4 figure supplement 1 source data 5/Figure S4G Tubulin.Tif]

Figure S4G

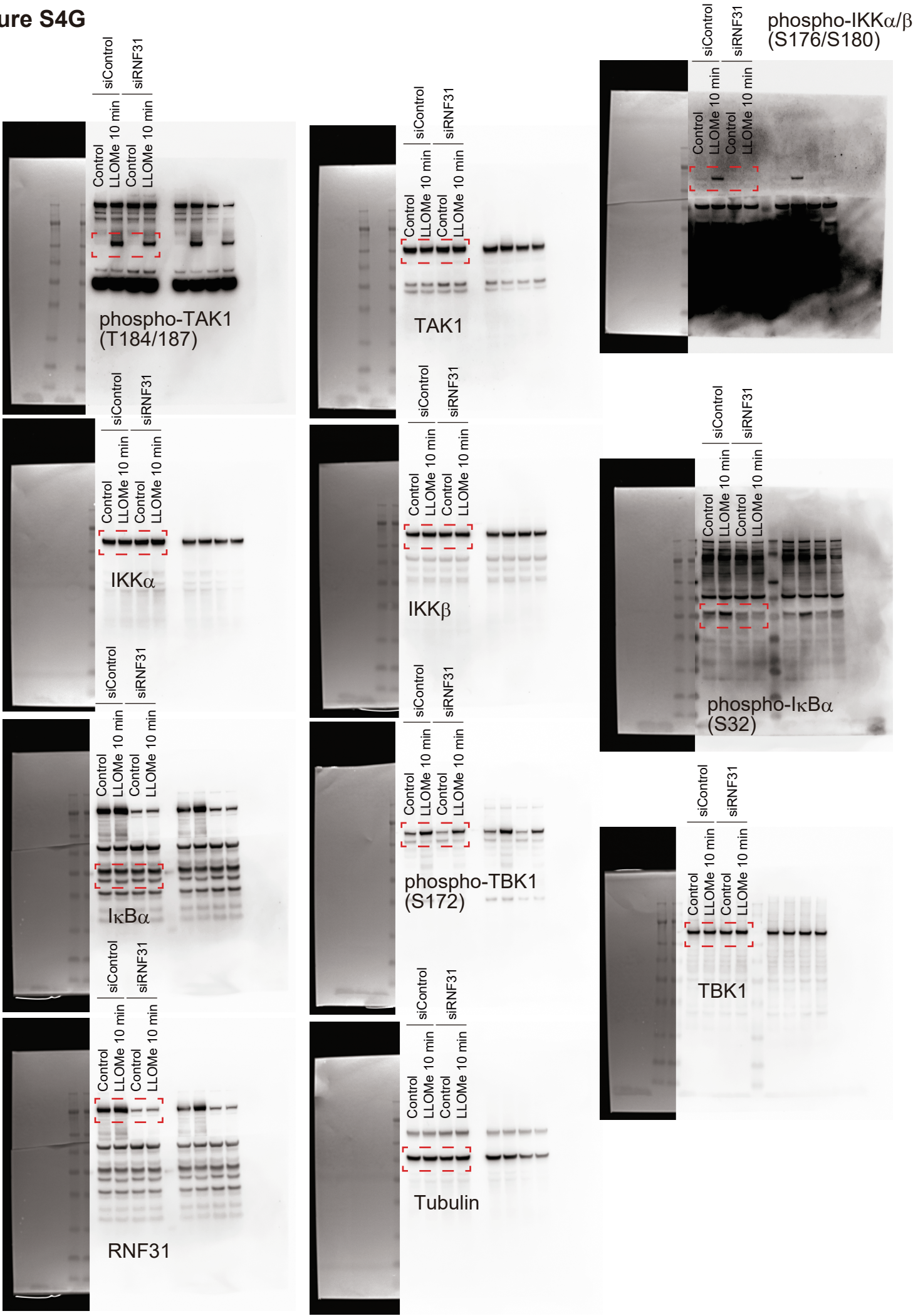

Supplement: Figure 4—figure supplement 1—source data 6. [file elife-106901-fig4-figsupp1-data6.pdf]

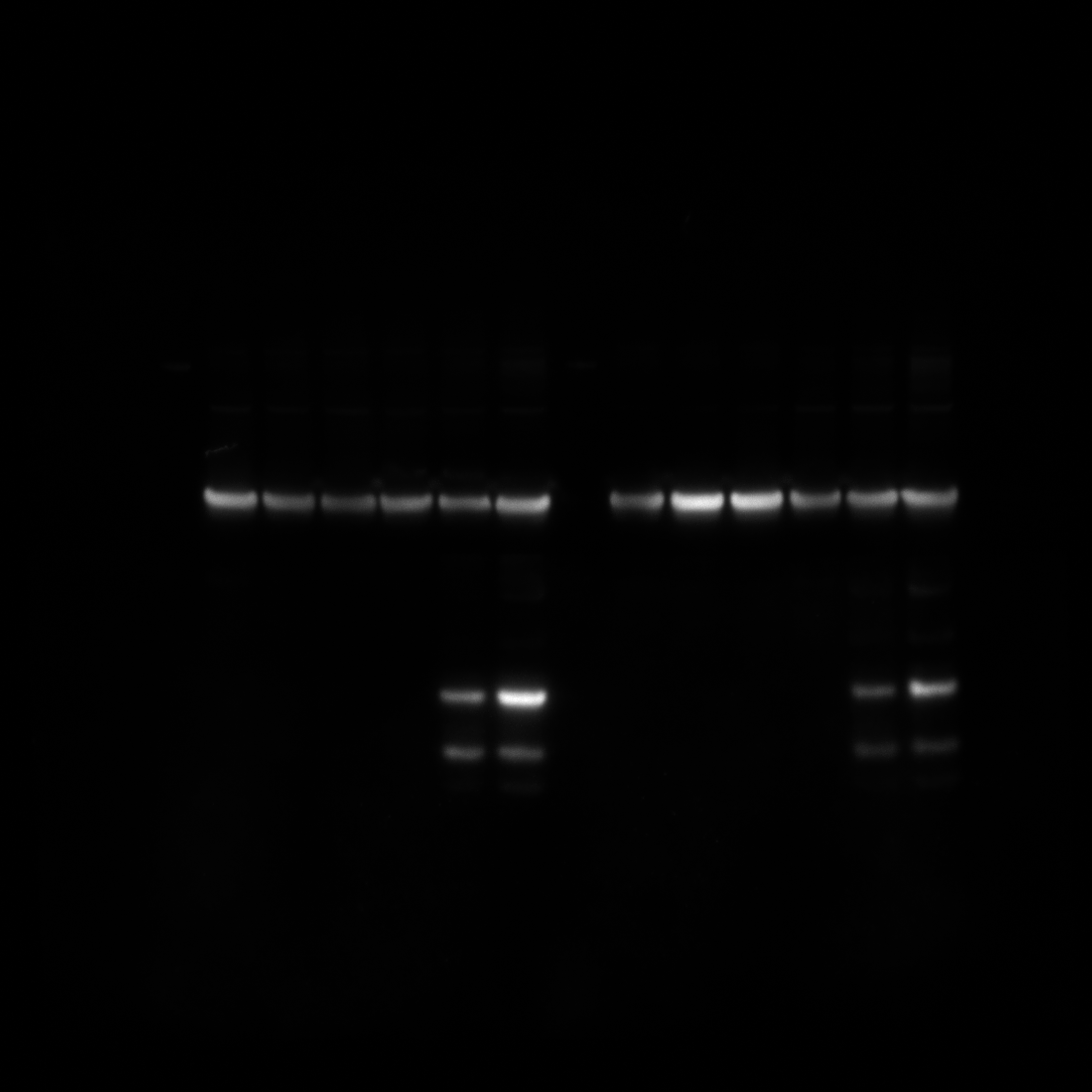

Supplement: Figure 5—source data 1. [file elife-106901-fig5-data1.zip › Figure5 source data 1/Figure 5D caspase3.Tif]

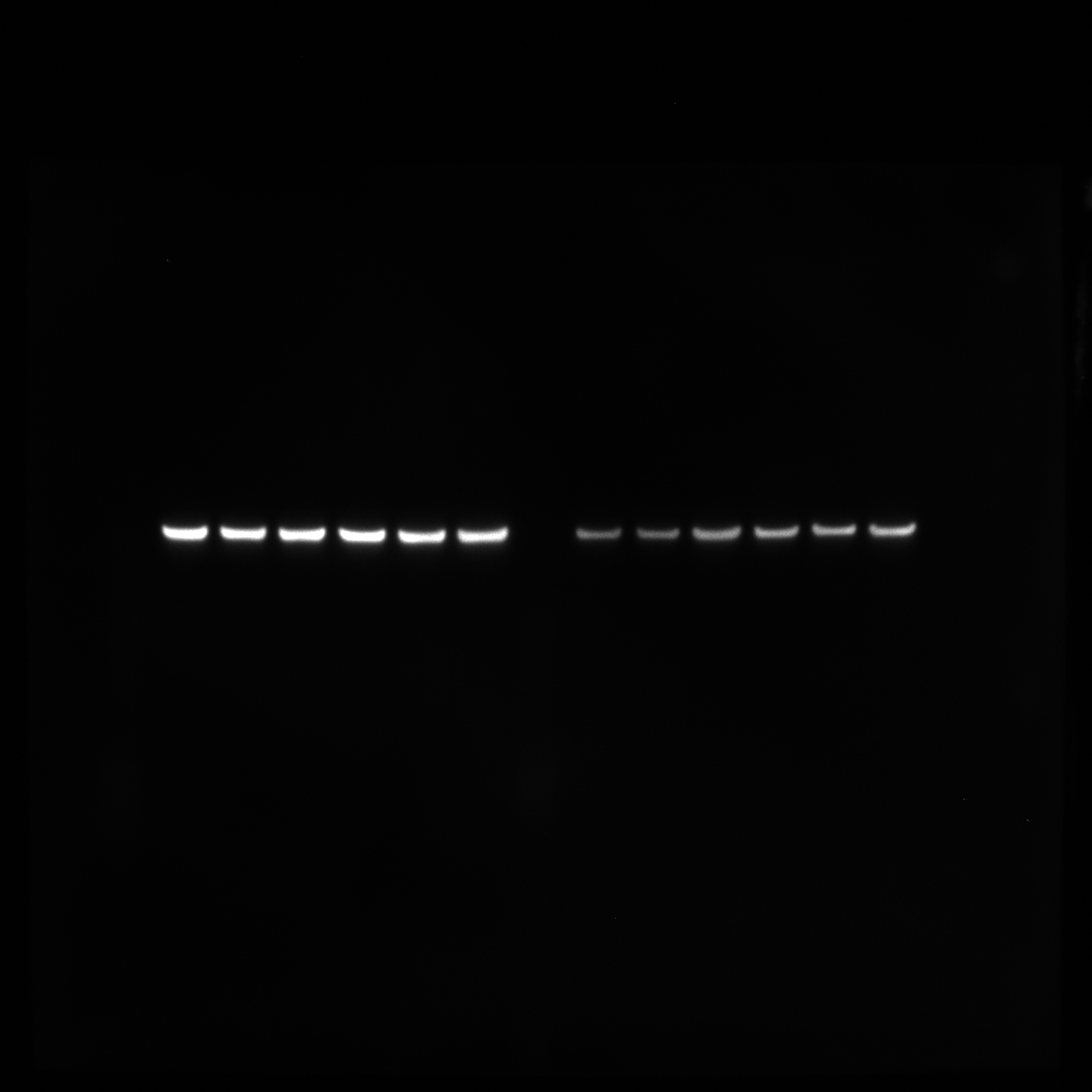

Supplement: Figure 5—source data 1. [file elife-106901-fig5-data1.zip › Figure5 source data 1/Figure 5D Tubulin.Tif]

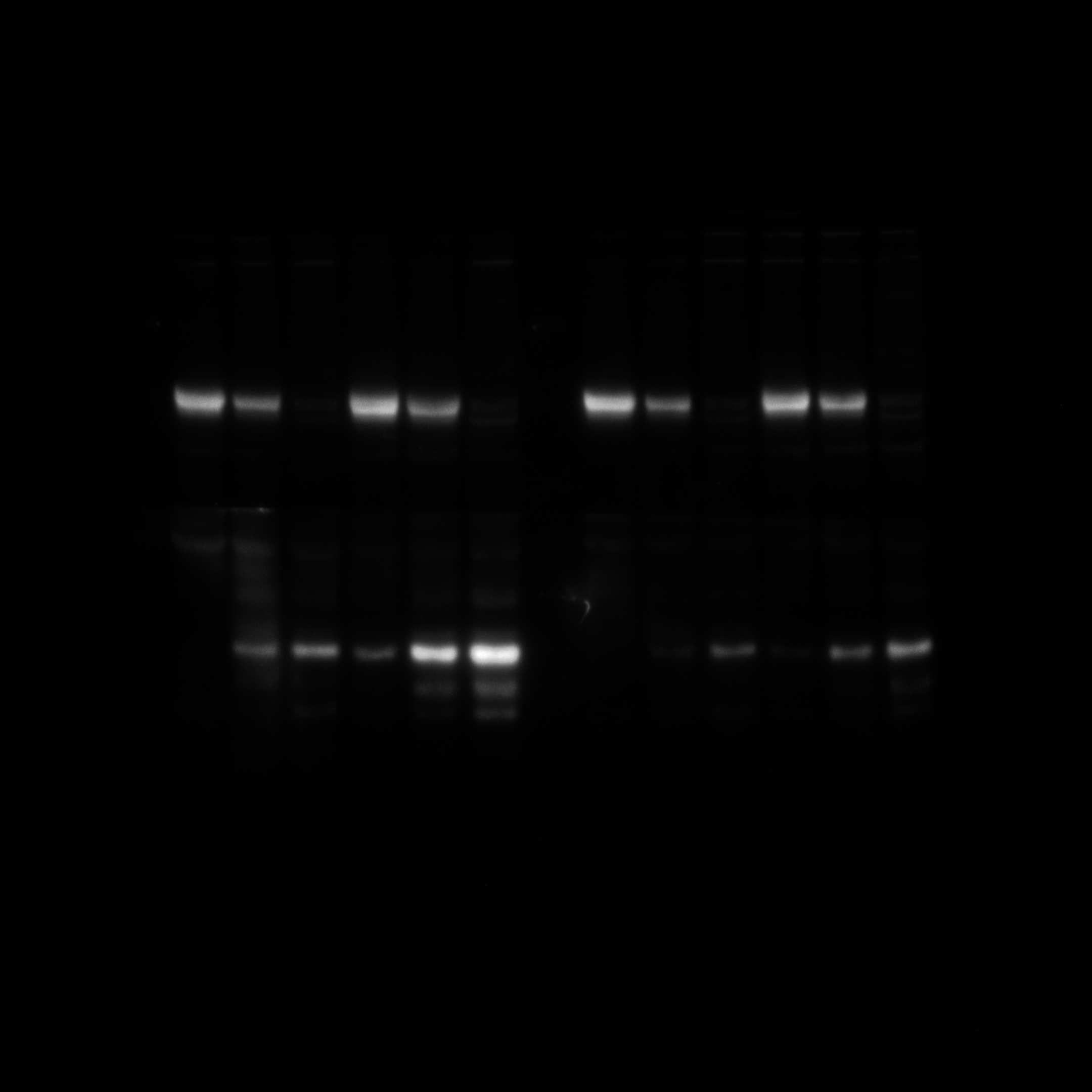

Supplement: Figure 5—source data 1. [file elife-106901-fig5-data1.zip › Figure5 source data 1/Figure 5F Caspase3.Tif]

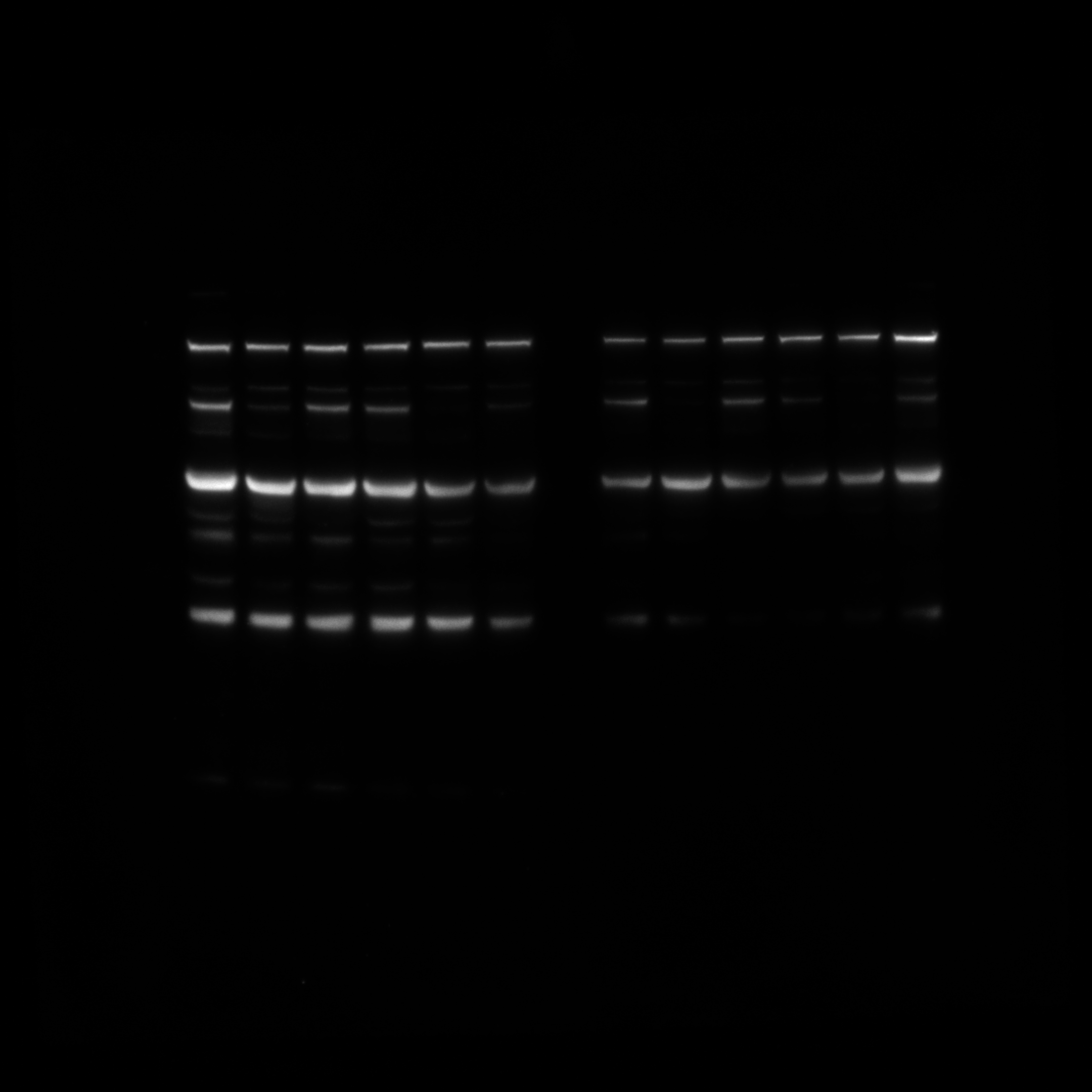

Supplement: Figure 5—source data 1. [file elife-106901-fig5-data1.zip › Figure5 source data 1/Figure 5F TAB2.Tif]

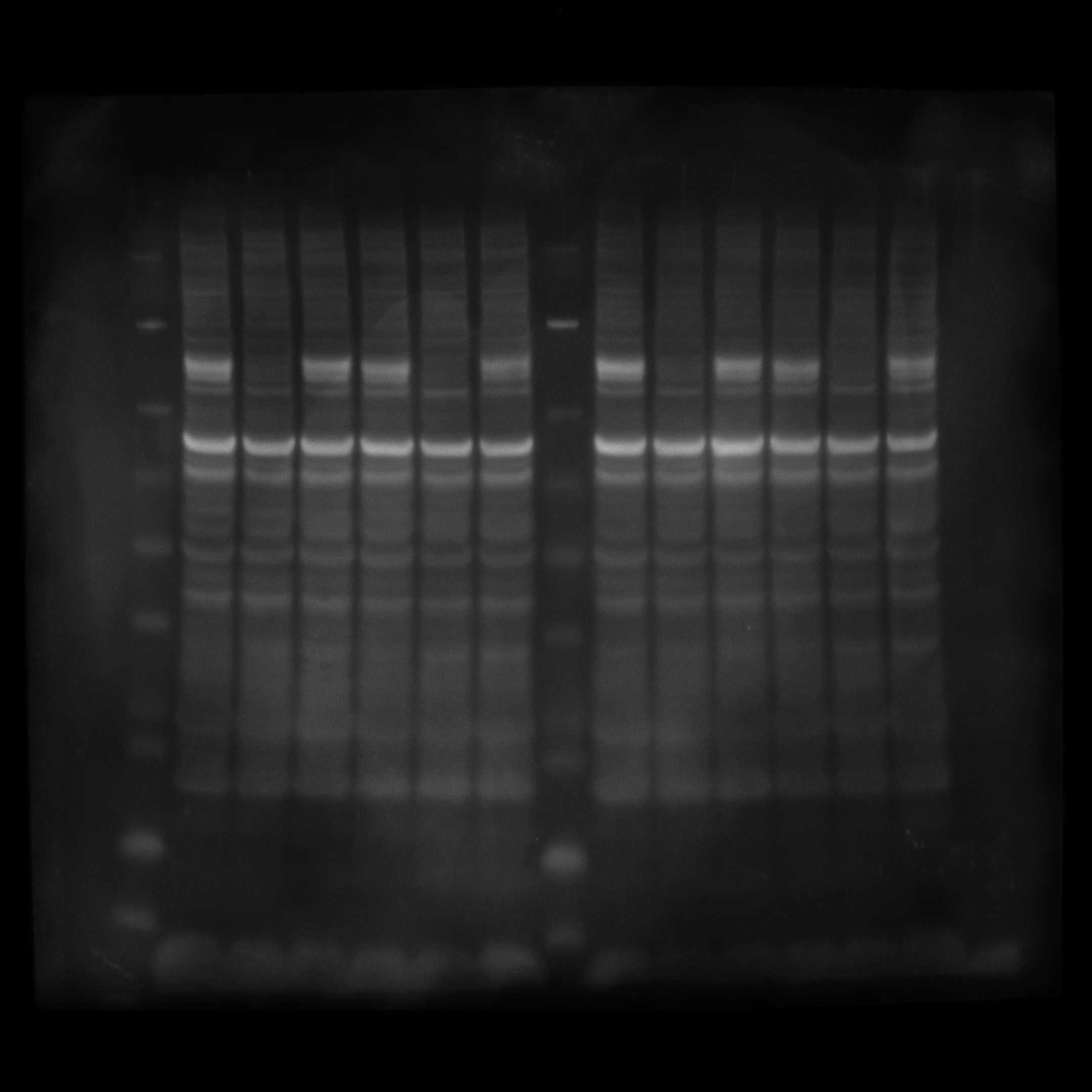

Supplement: Figure 5—source data 1. [file elife-106901-fig5-data1.zip › Figure5 source data 1/Figure 5F TAB3.Tif]

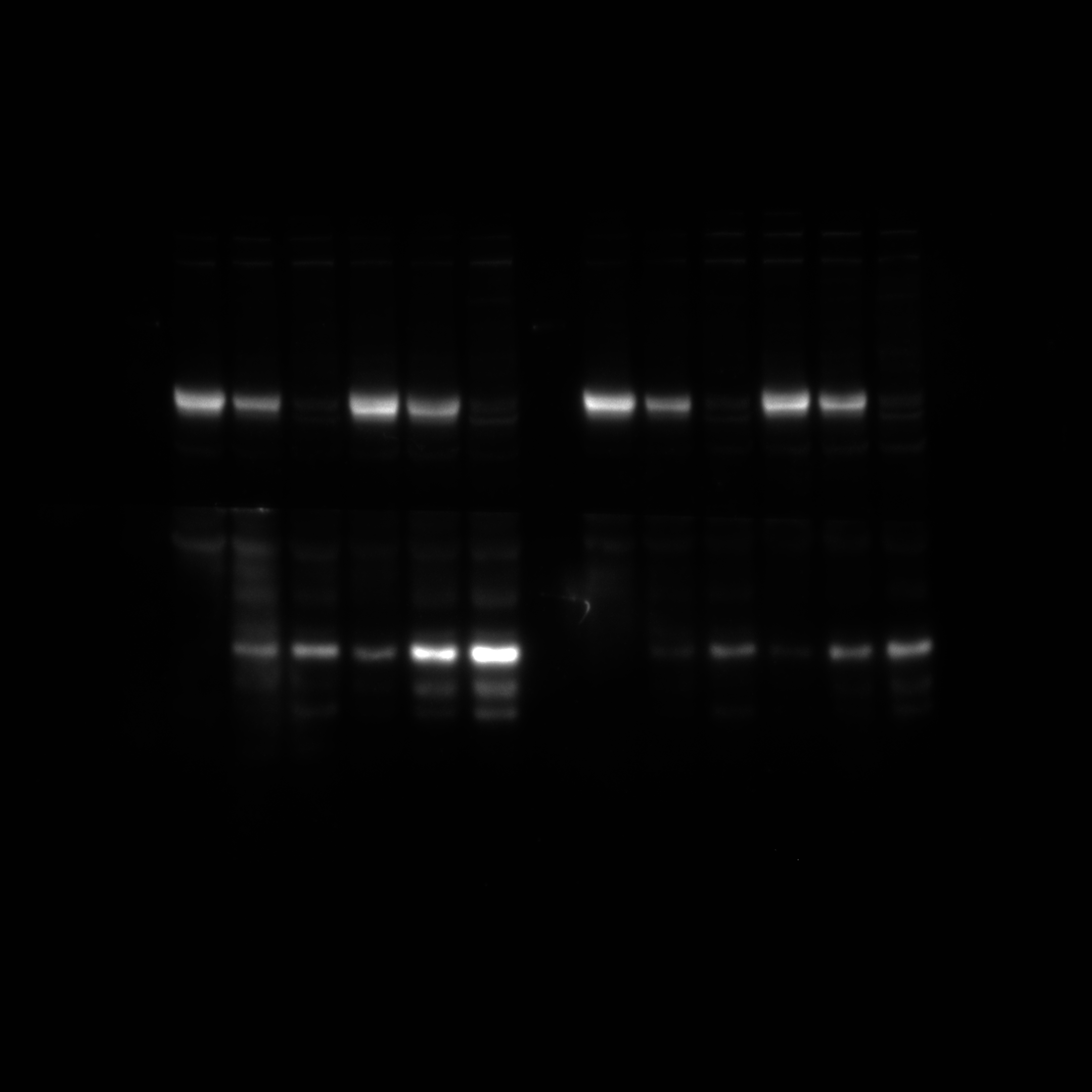

Supplement: Figure 5—source data 1. [file elife-106901-fig5-data1.zip › Figure5 source data 1/Figure 5F TAK1.Tif]

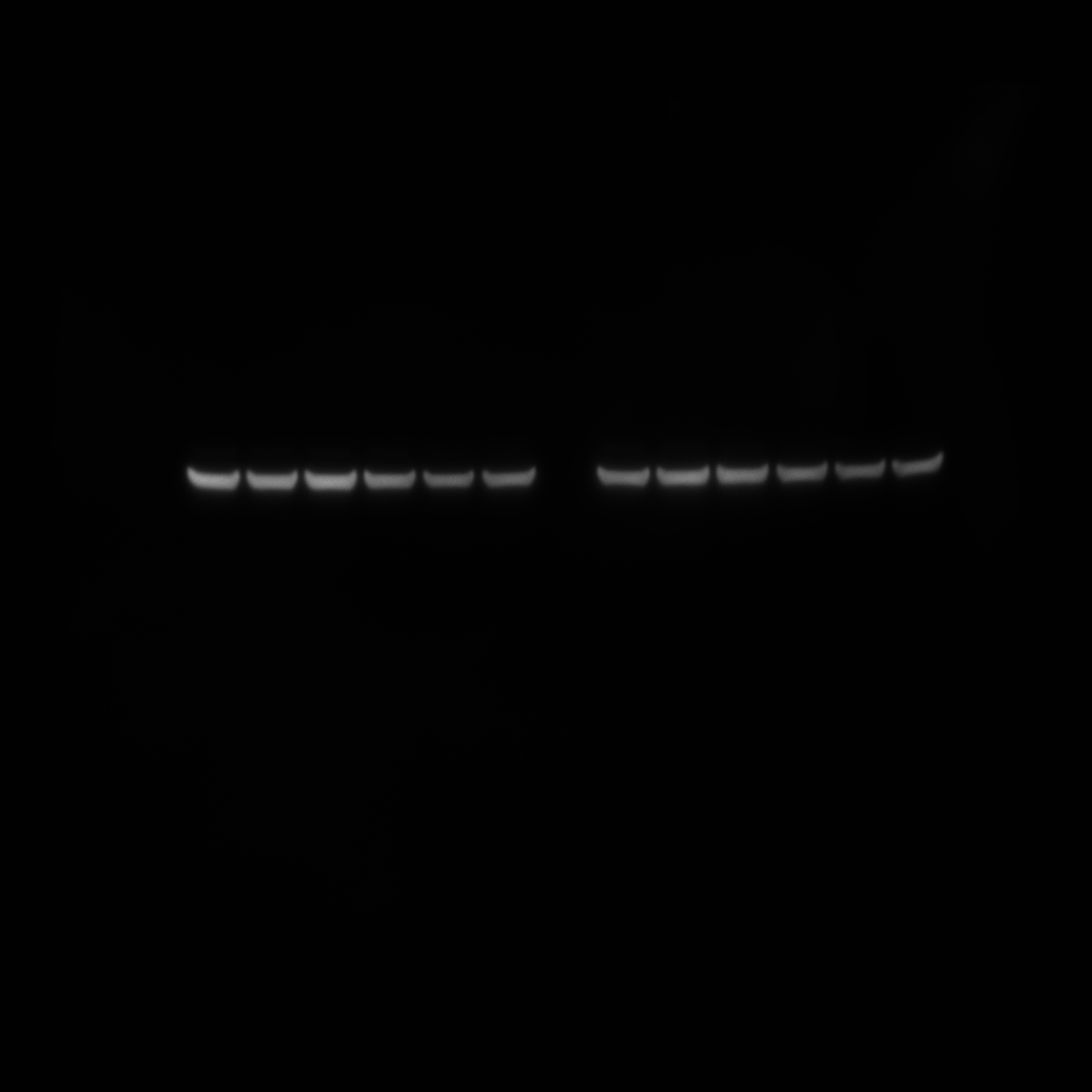

Supplement: Figure 5—source data 1. [file elife-106901-fig5-data1.zip › Figure5 source data 1/Figure 5F Tubulin.Tif]

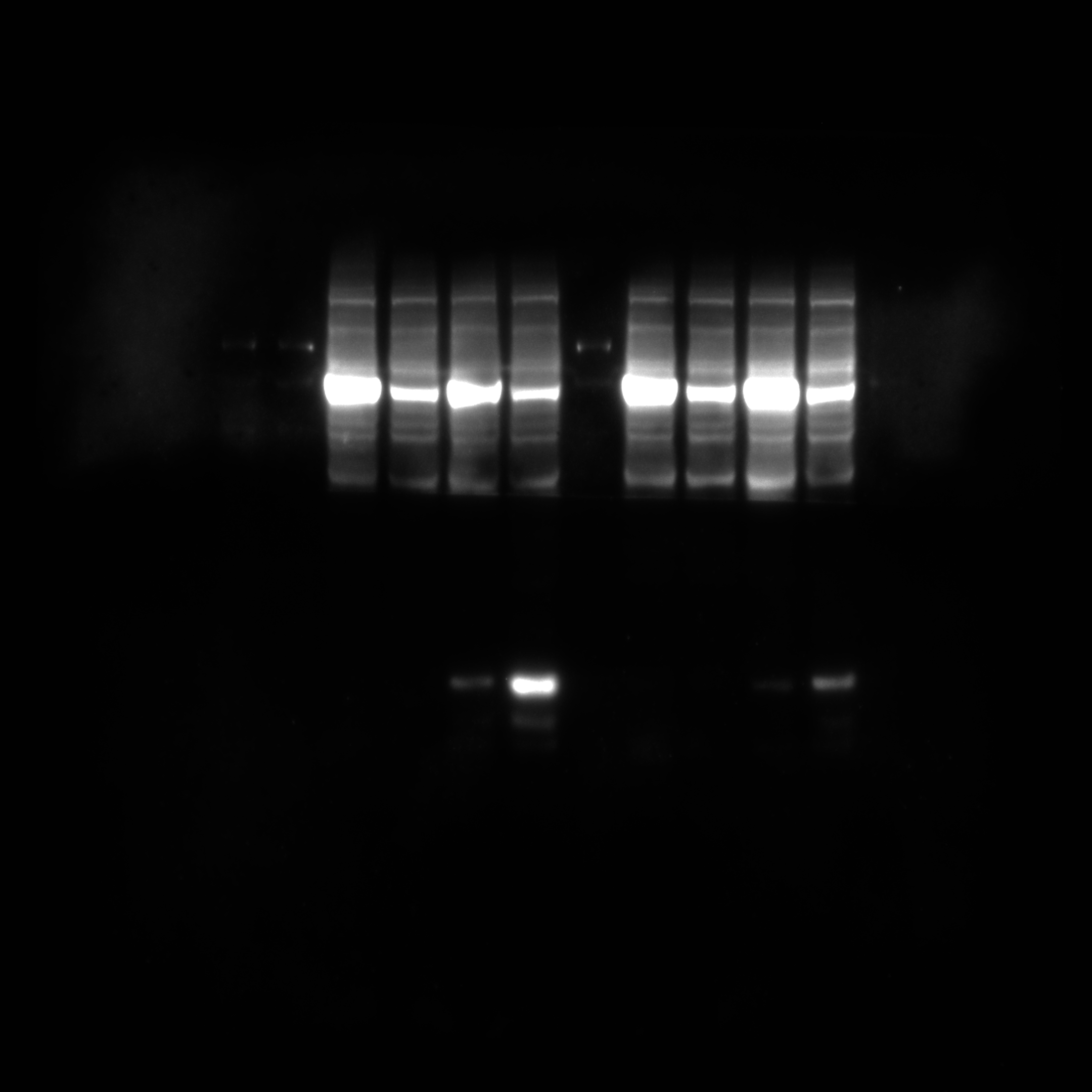

Supplement: Figure 5—source data 1. [file elife-106901-fig5-data1.zip › Figure5 source data 1/Figure 5G Caspase3.Tif]

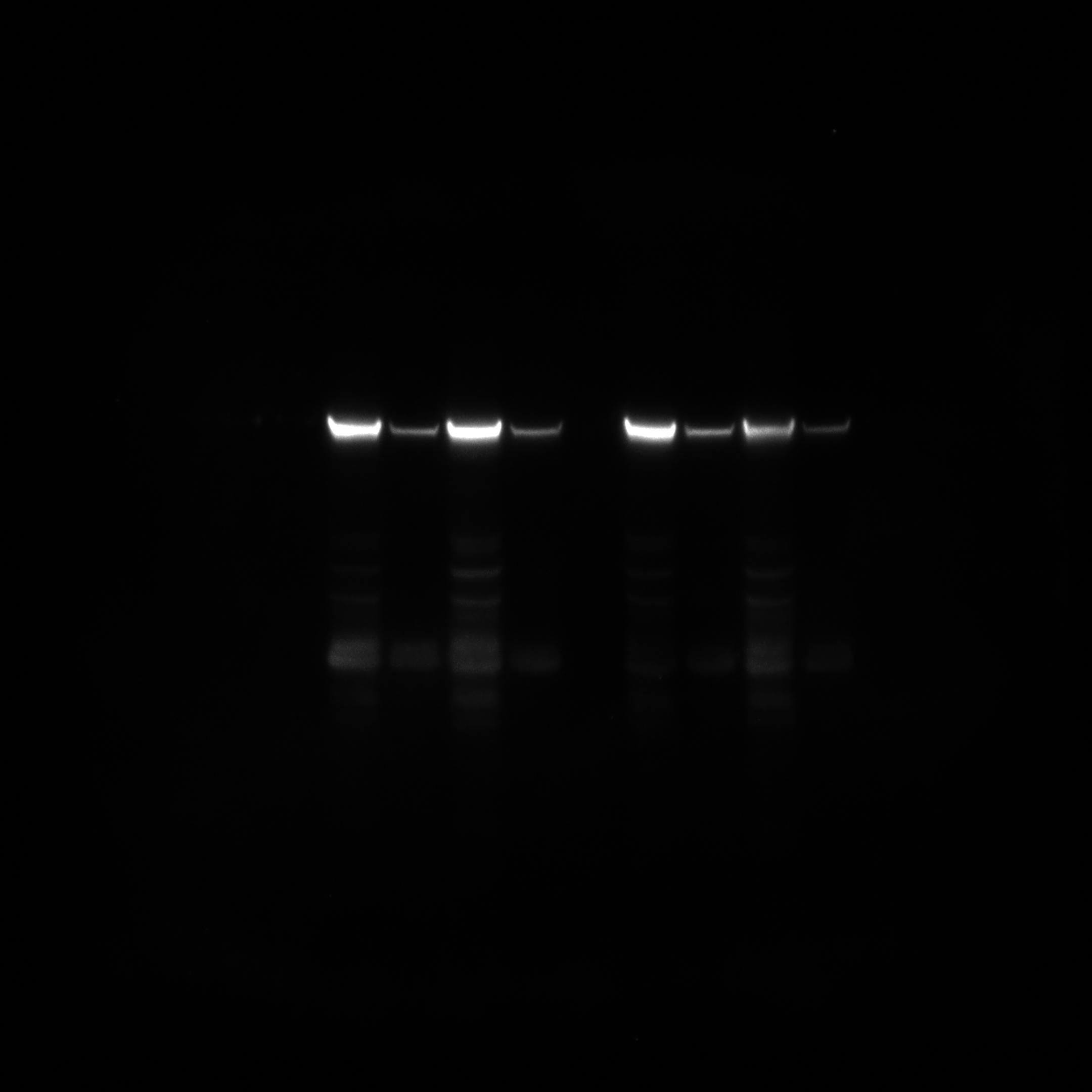

Supplement: Figure 5—source data 1. [file elife-106901-fig5-data1.zip › Figure5 source data 1/Figure 5G IKKa.Tif]

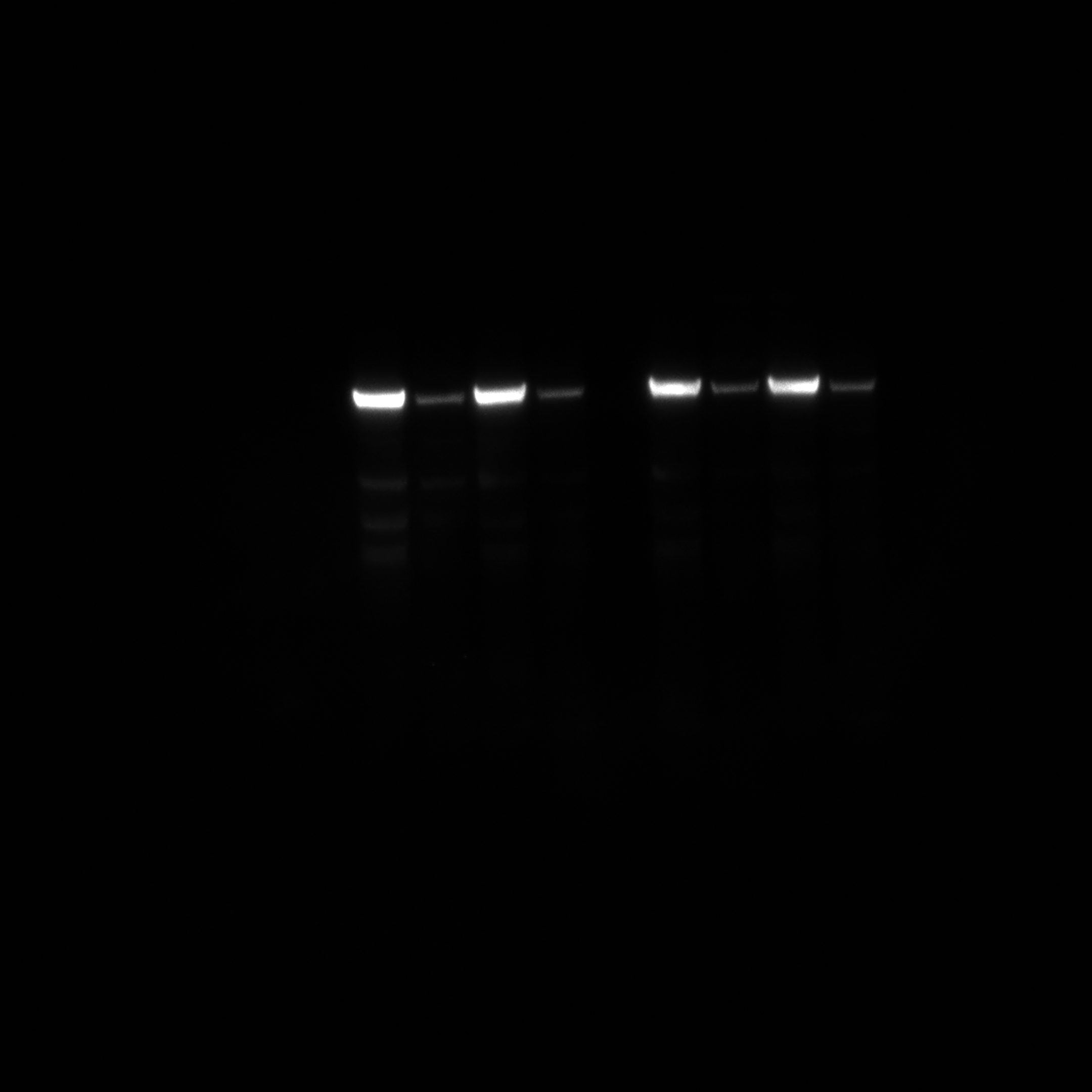

Supplement: Figure 5—source data 1. [file elife-106901-fig5-data1.zip › Figure5 source data 1/Figure 5G IKKb.Tif]

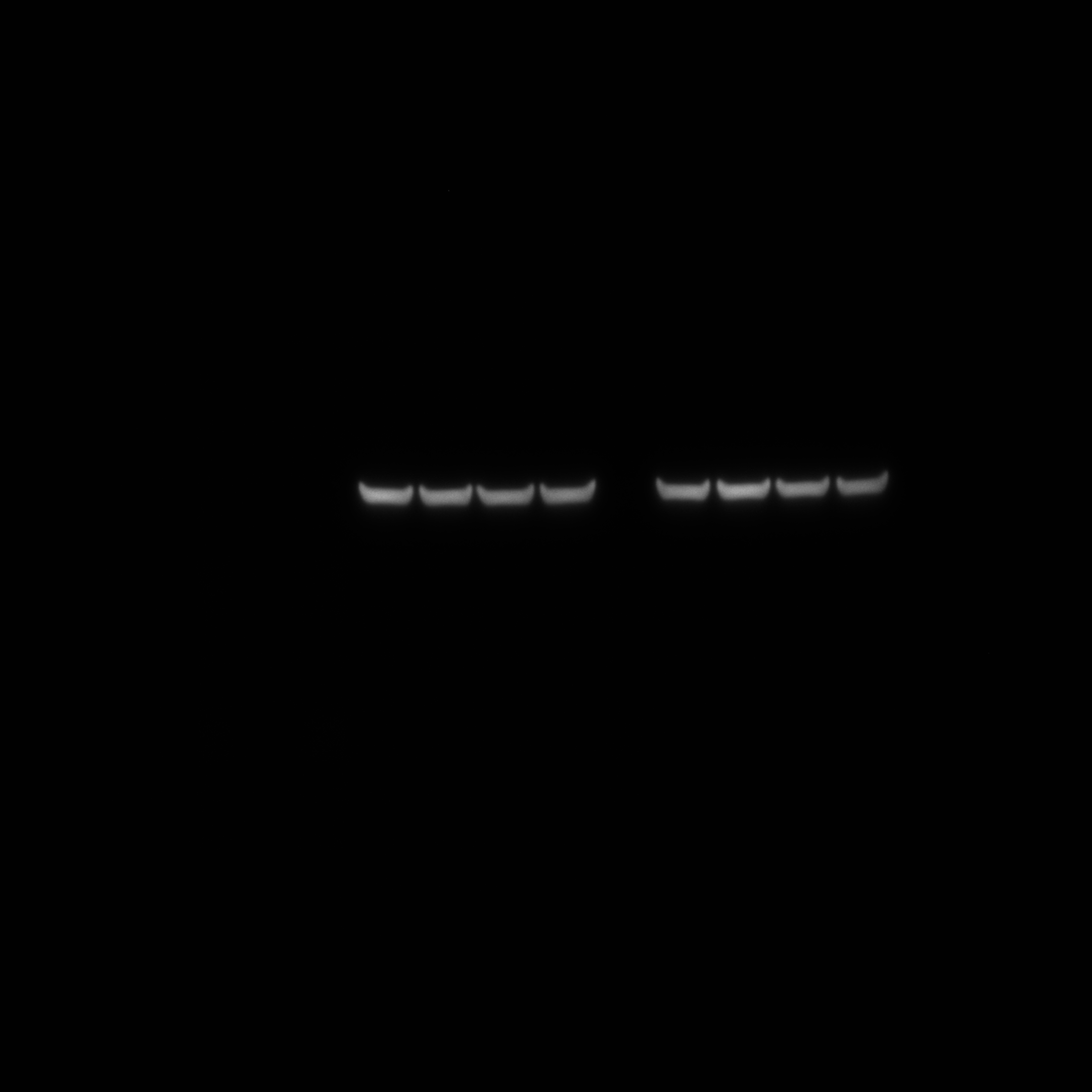

Supplement: Figure 5—source data 1. [file elife-106901-fig5-data1.zip › Figure5 source data 1/Figure 5G Tubulin.Tif]

**Figure 5D**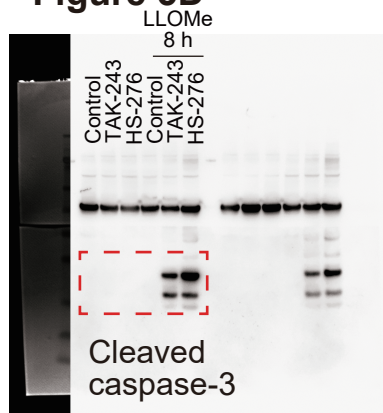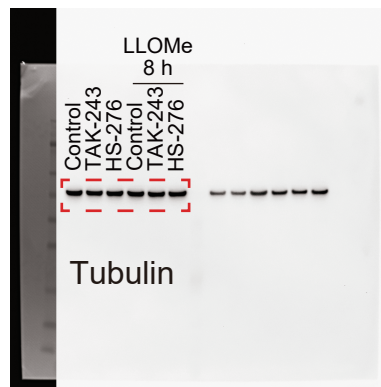**Figure 5F**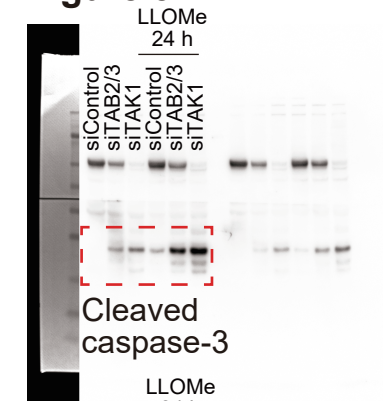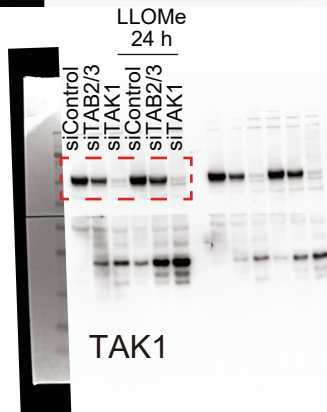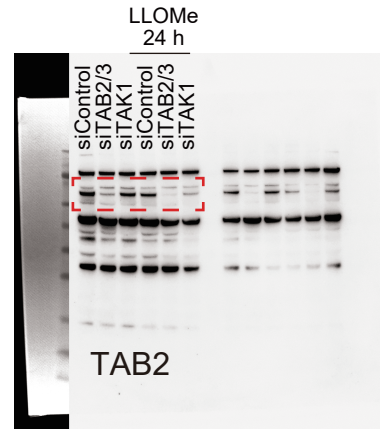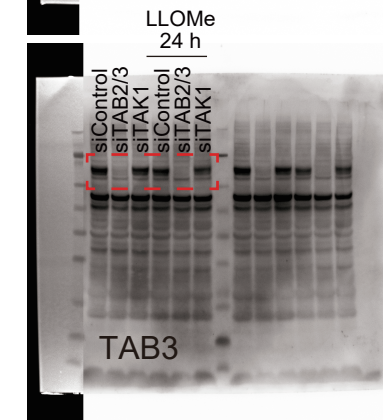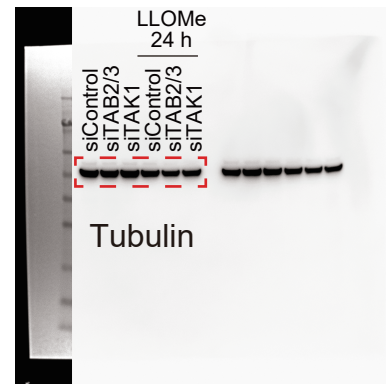**Figure 5G**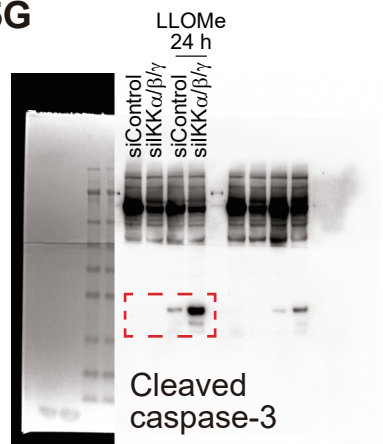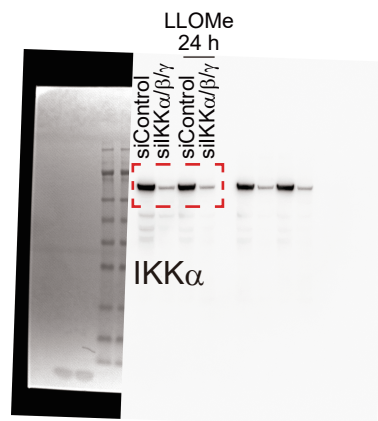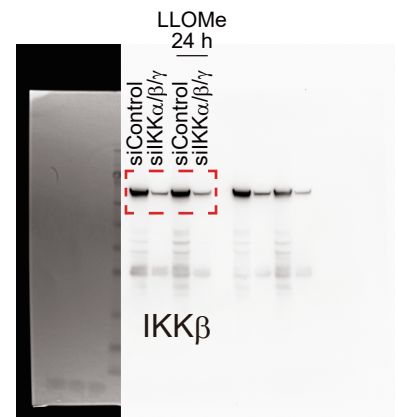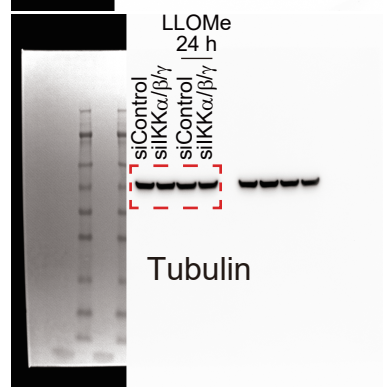

Supplement: Figure 5—source data 2. [file elife-106901-fig5-data2.pdf]

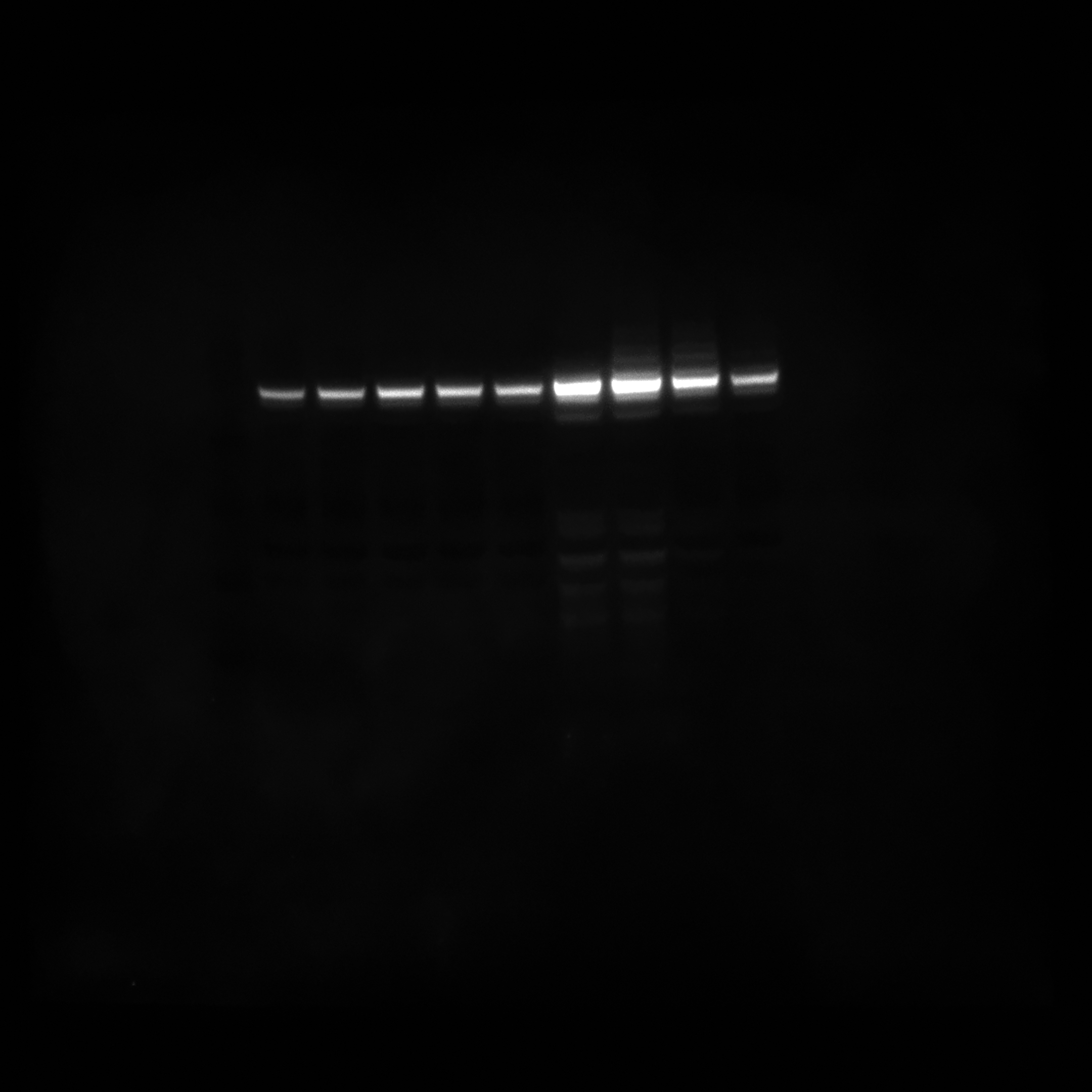

Supplement: Figure 5—source data 3. [file elife-106901-fig5-data3.zip › Figure5 source data 3/Figure 5H pSTAT3.Tif]

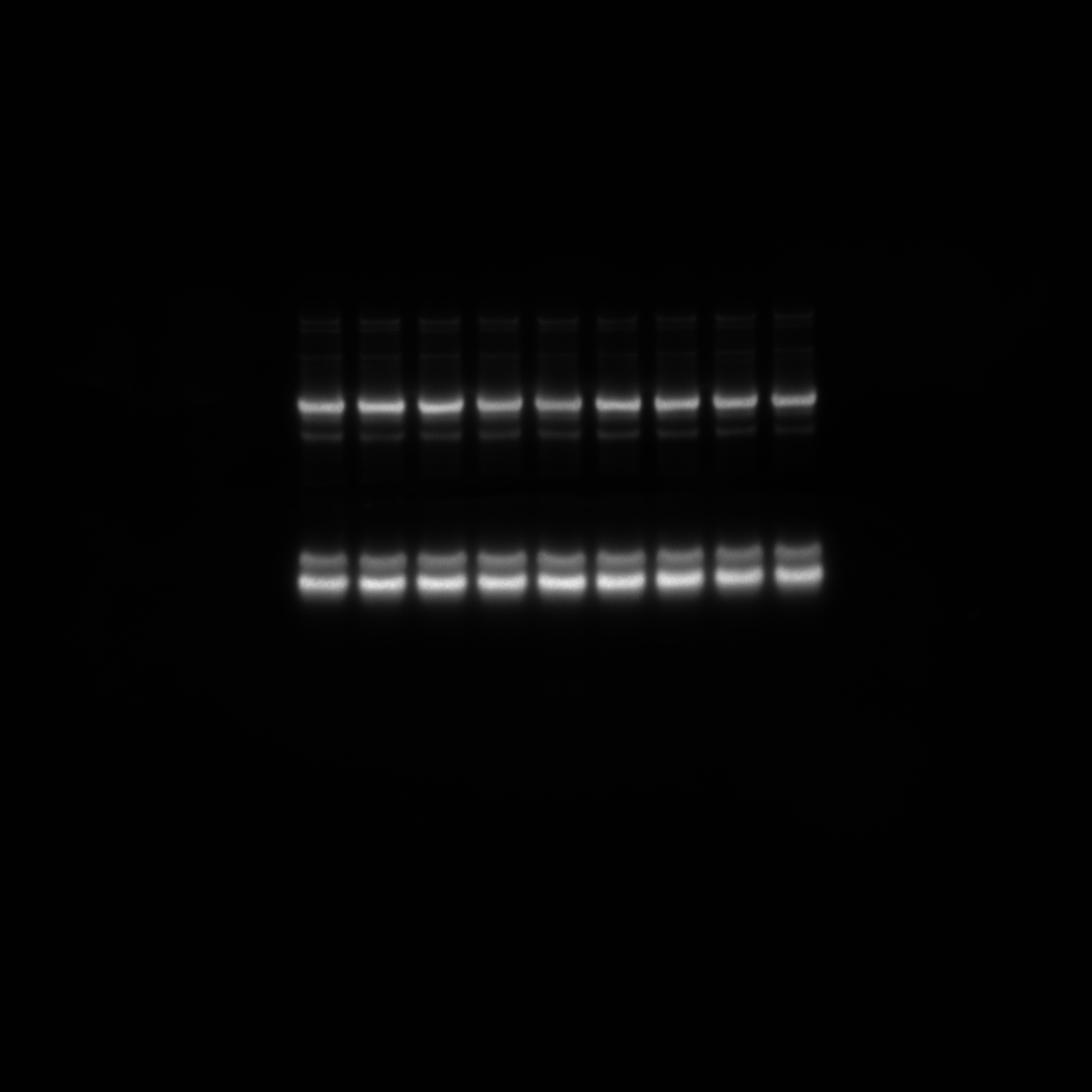

Supplement: Figure 5—source data 3. [file elife-106901-fig5-data3.zip › Figure5 source data 3/Figure 5H STAT3.Tif]

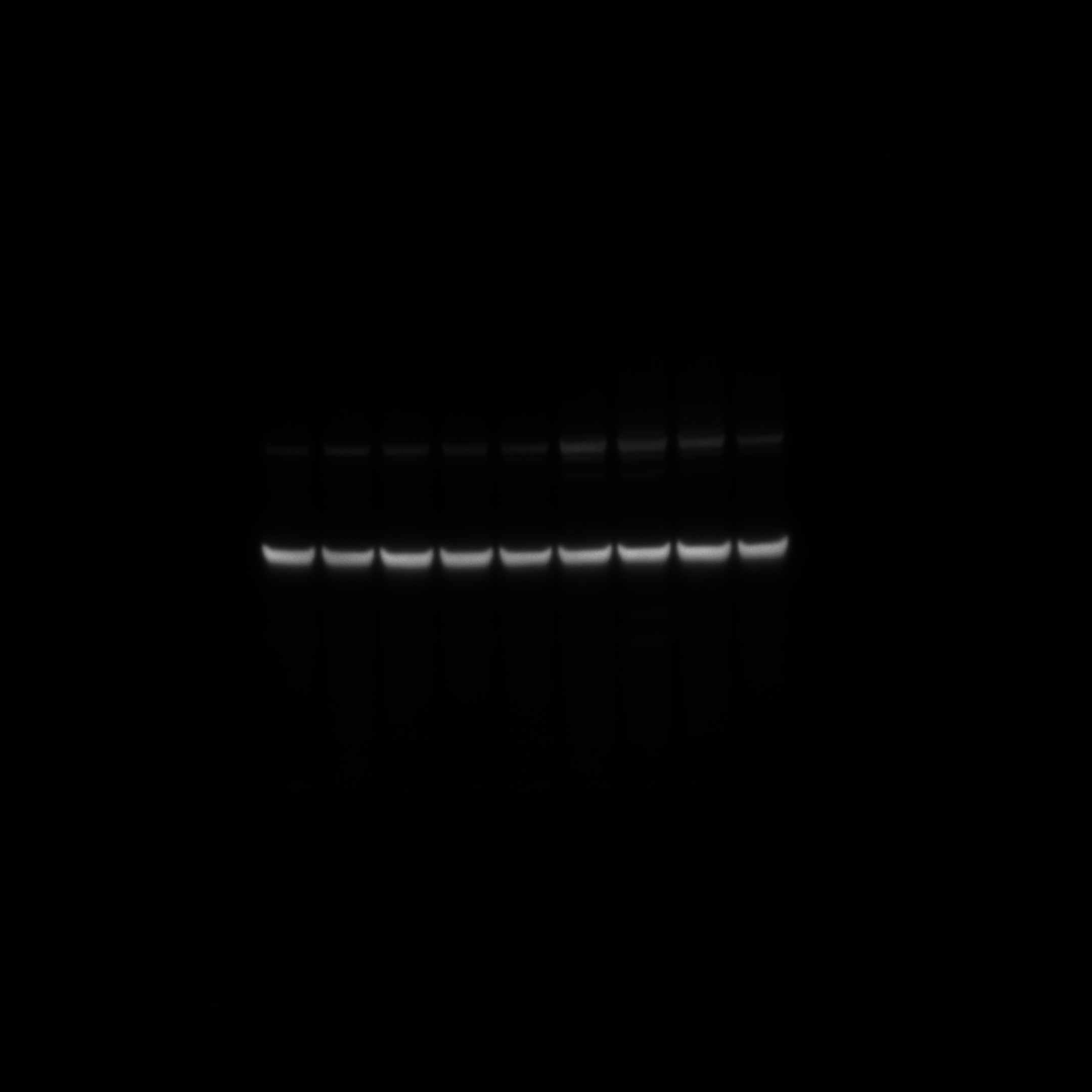

Supplement: Figure 5—source data 3. [file elife-106901-fig5-data3.zip › Figure5 source data 3/Figure 5H Tubulin.Tif]

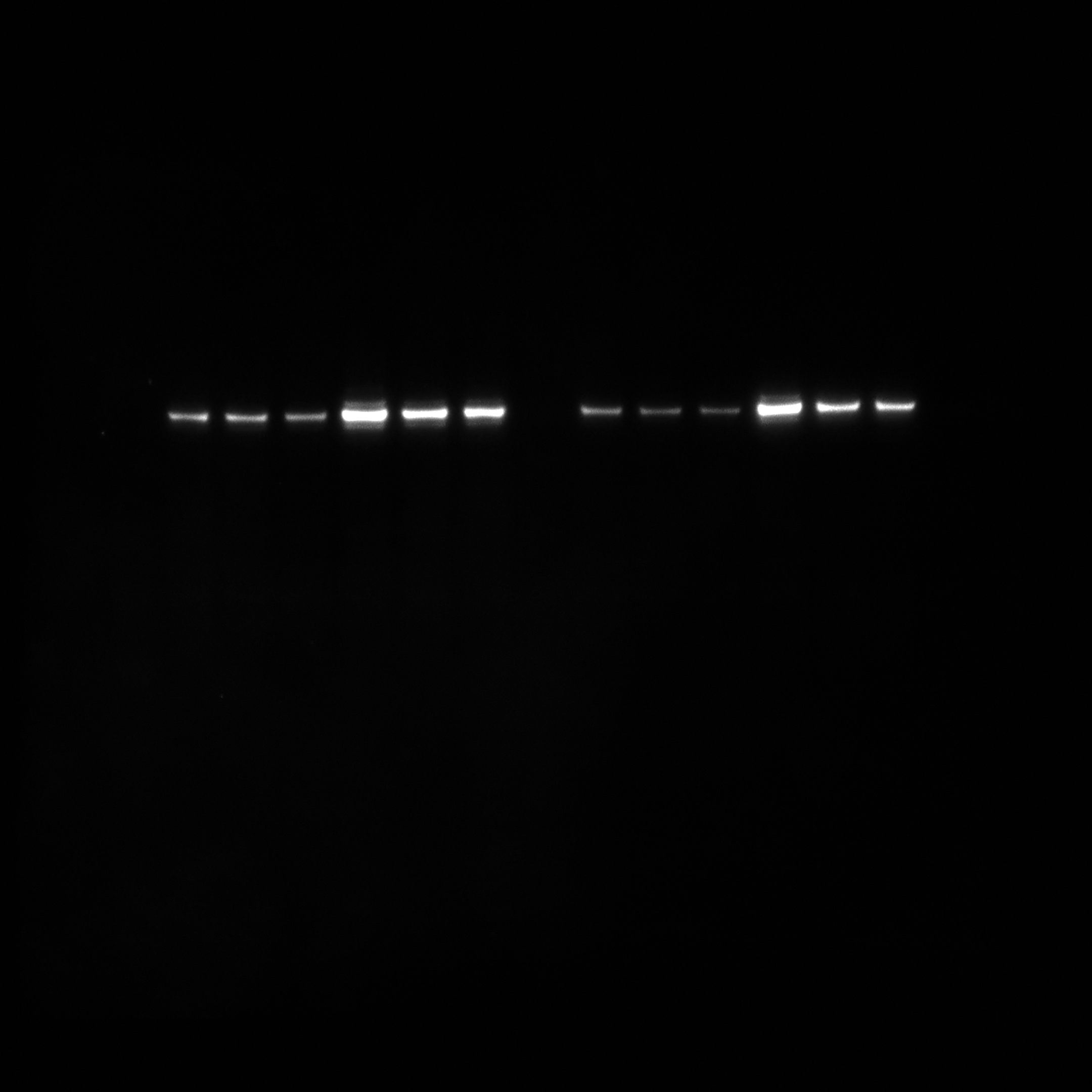

Supplement: Figure 5—source data 3. [file elife-106901-fig5-data3.zip › Figure5 source data 3/Figure 5I pSTAT3.Tif]

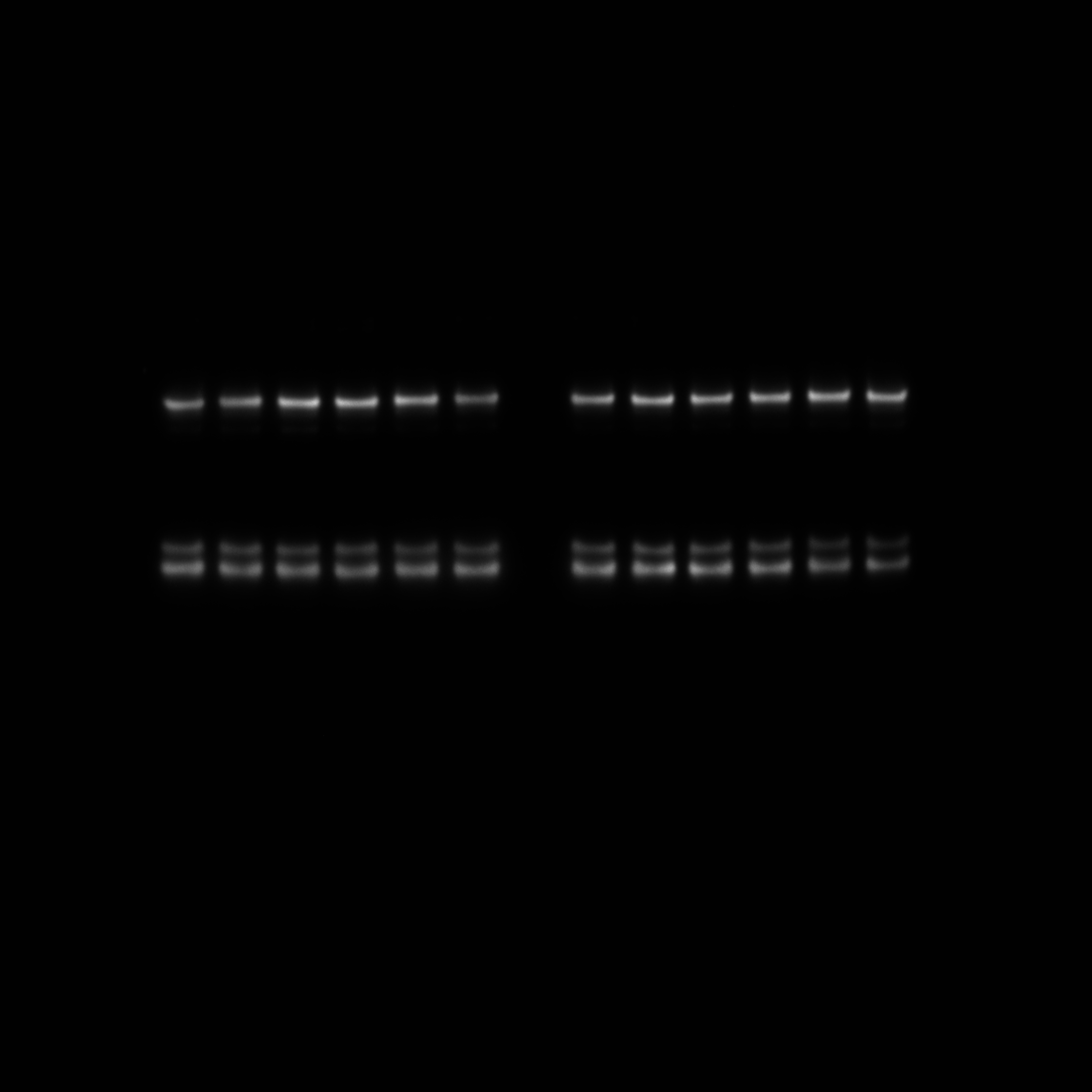

Supplement: Figure 5—source data 3. [file elife-106901-fig5-data3.zip › Figure5 source data 3/Figure 5I STAT3.Tif]

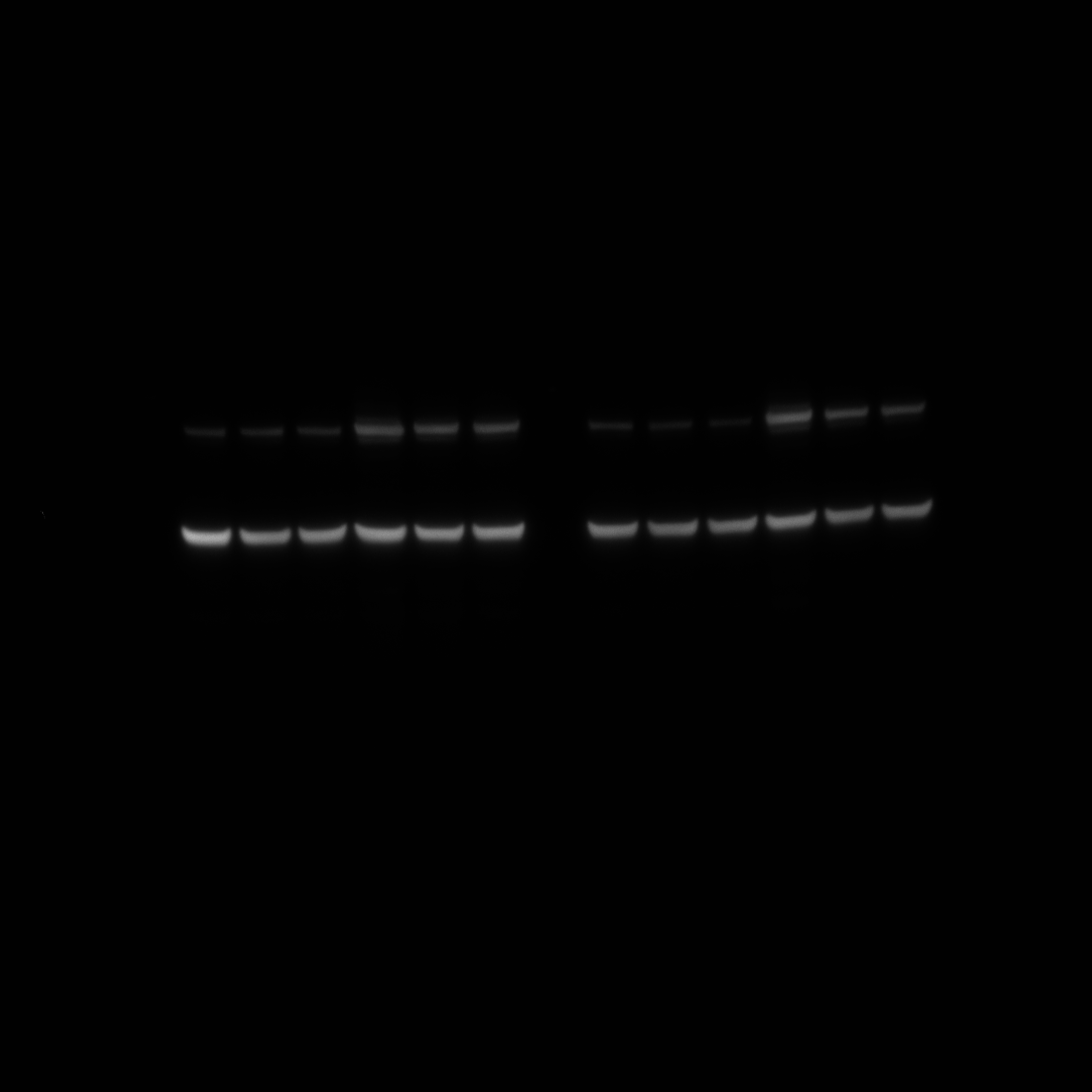

Supplement: Figure 5—source data 3. [file elife-106901-fig5-data3.zip › Figure5 source data 3/Figure 5I Tubulin.Tif]

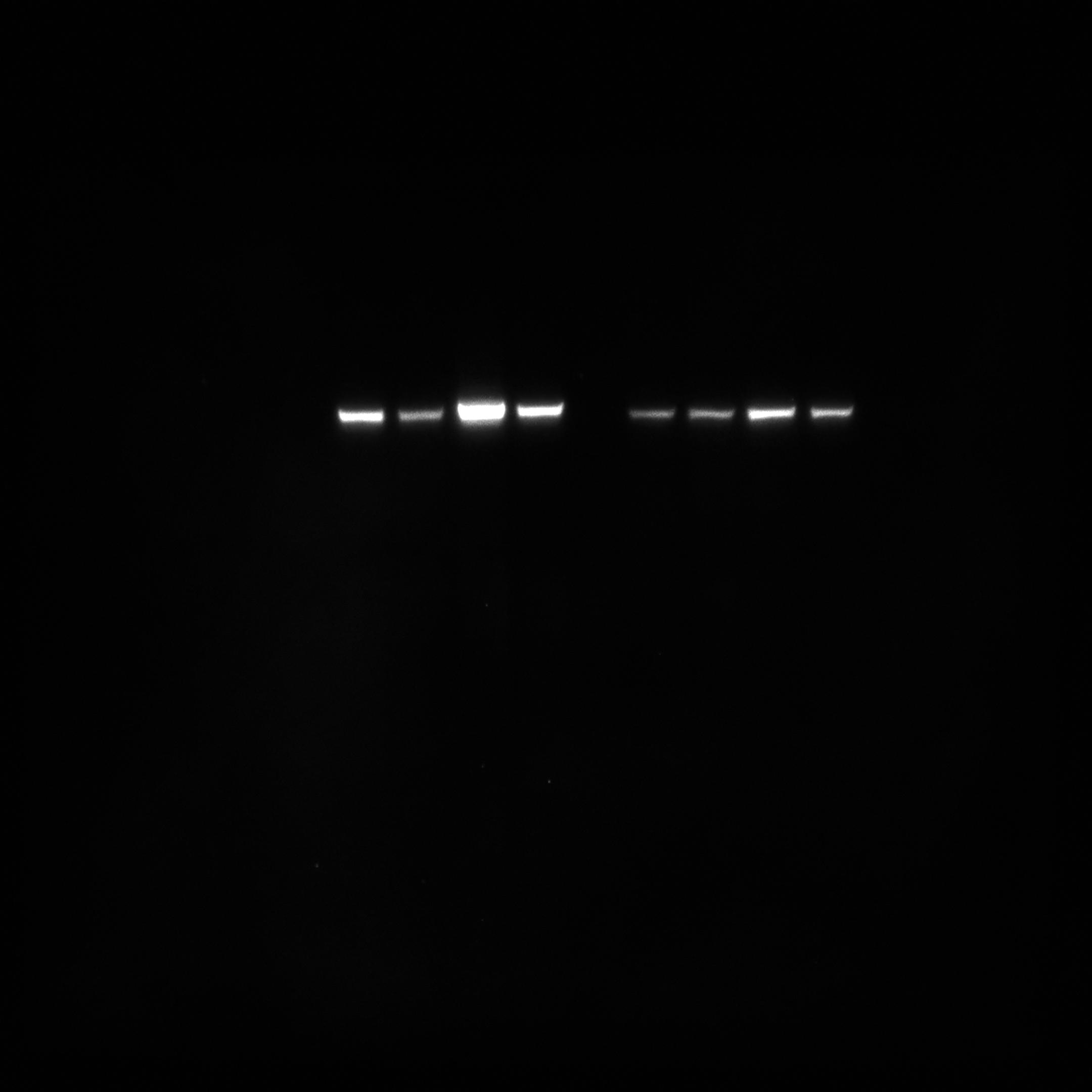

Supplement: Figure 5—source data 3. [file elife-106901-fig5-data3.zip › Figure5 source data 3/Figure 5J pSTAT3.Tif]

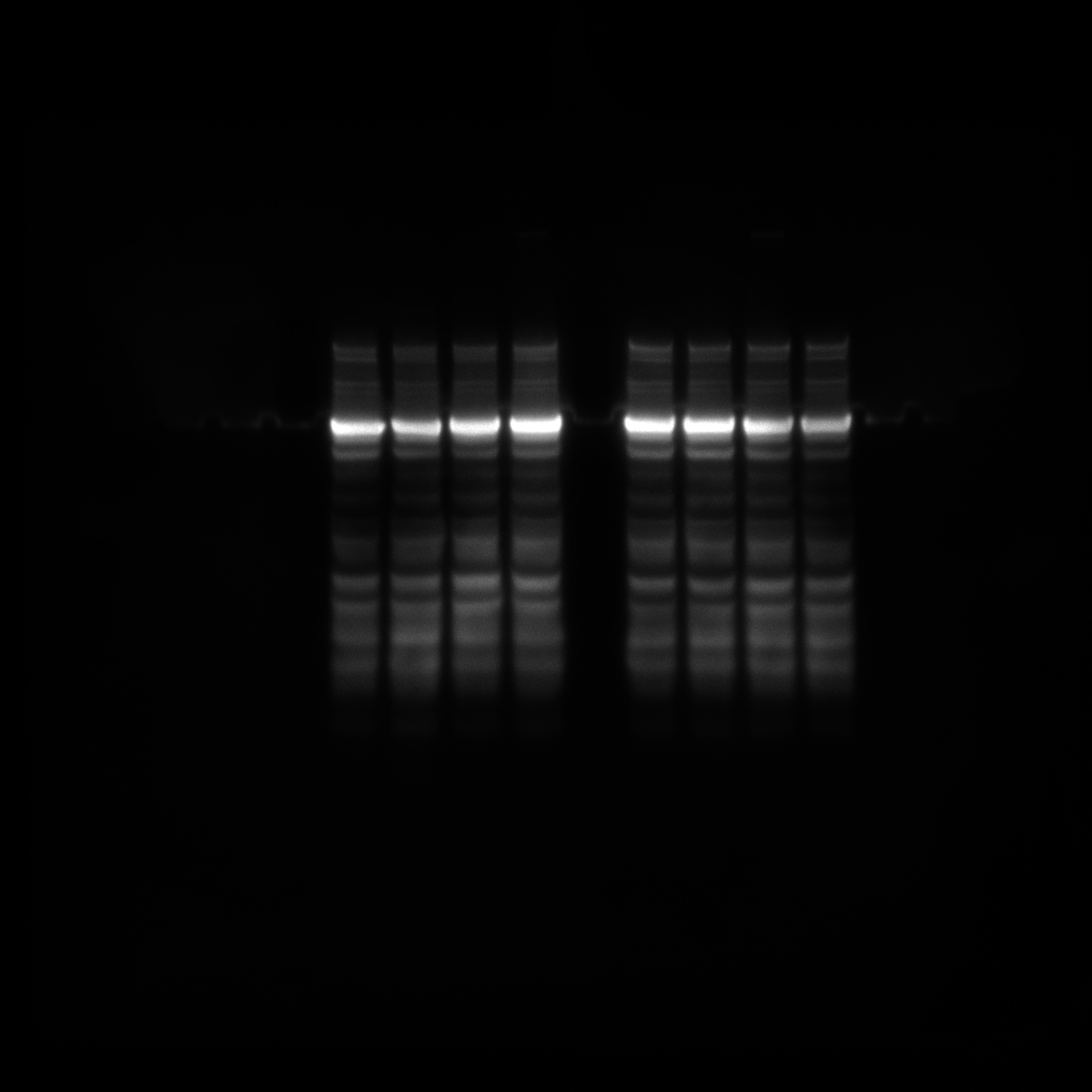

Supplement: Figure 5—source data 3. [file elife-106901-fig5-data3.zip › Figure5 source data 3/Figure 5J STAT3.Tif]

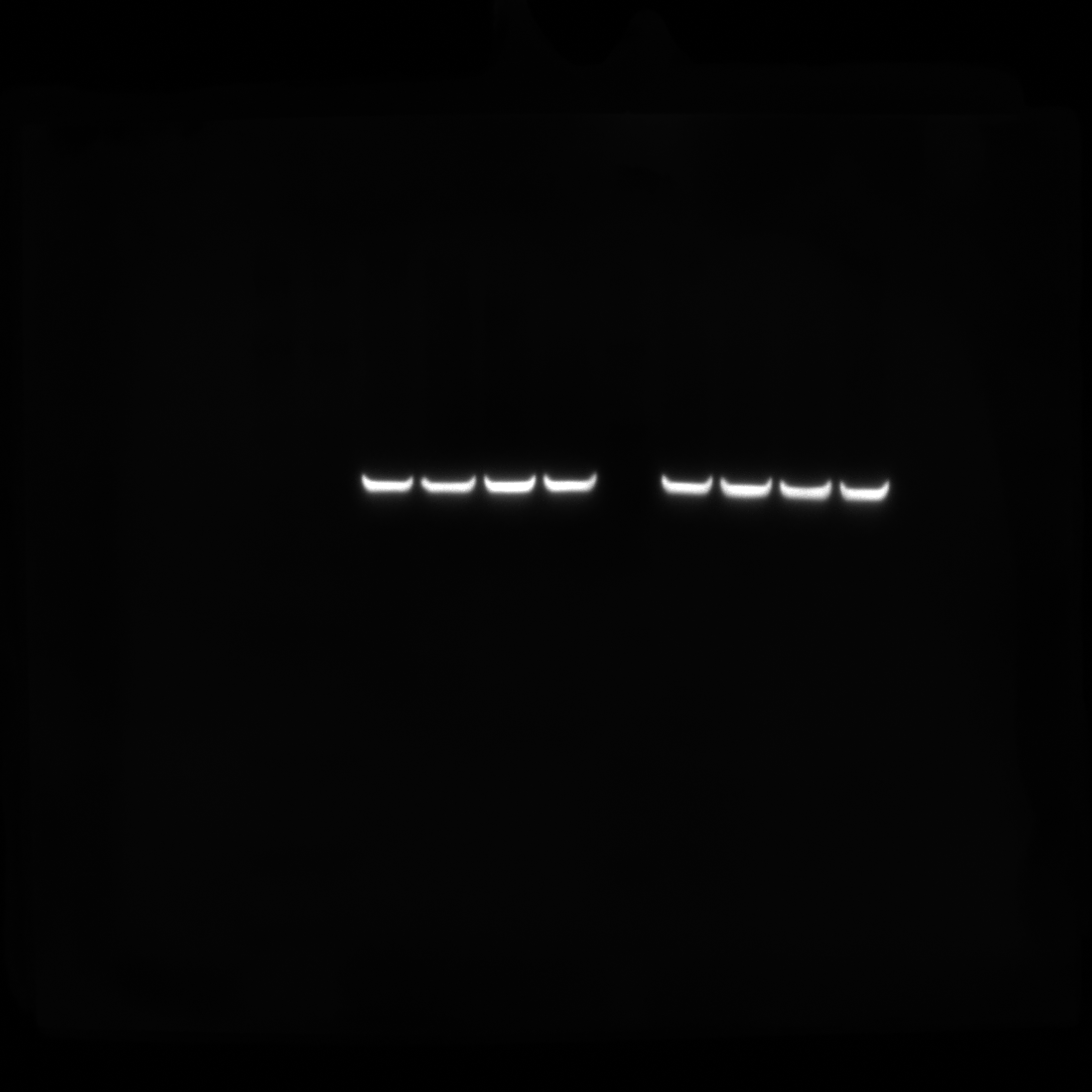

Supplement: Figure 5—source data 3. [file elife-106901-fig5-data3.zip › Figure5 source data 3/Figure 5J Tubulin.Tif]

Figure 5H

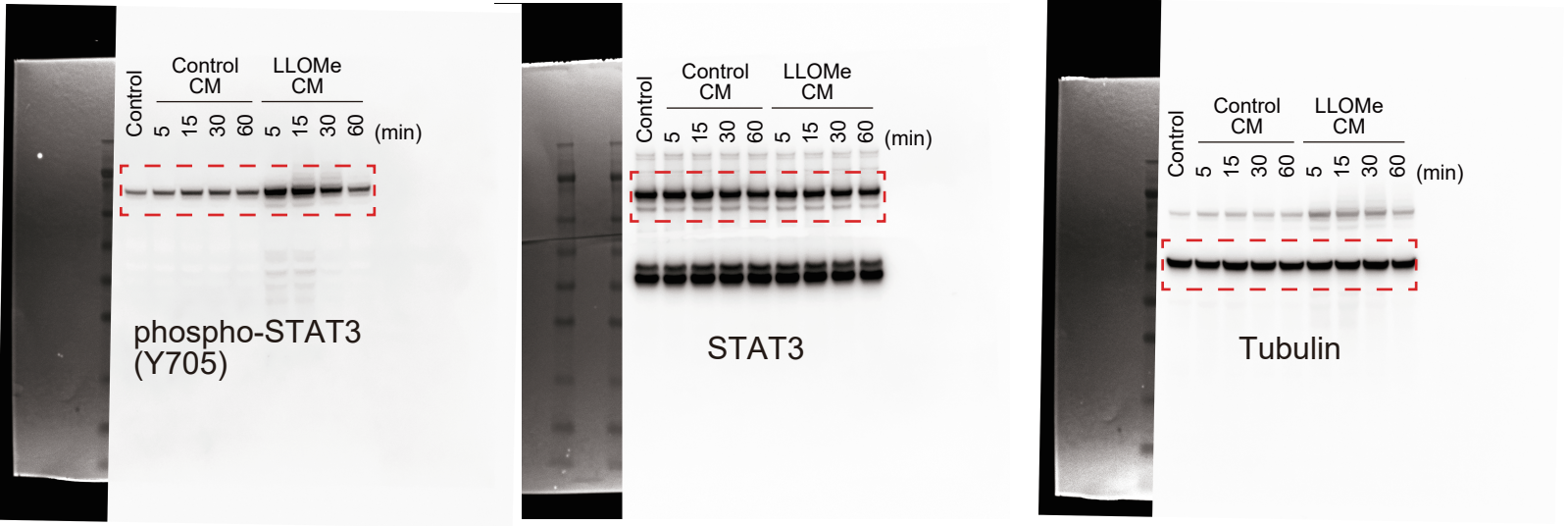

Figure 5I

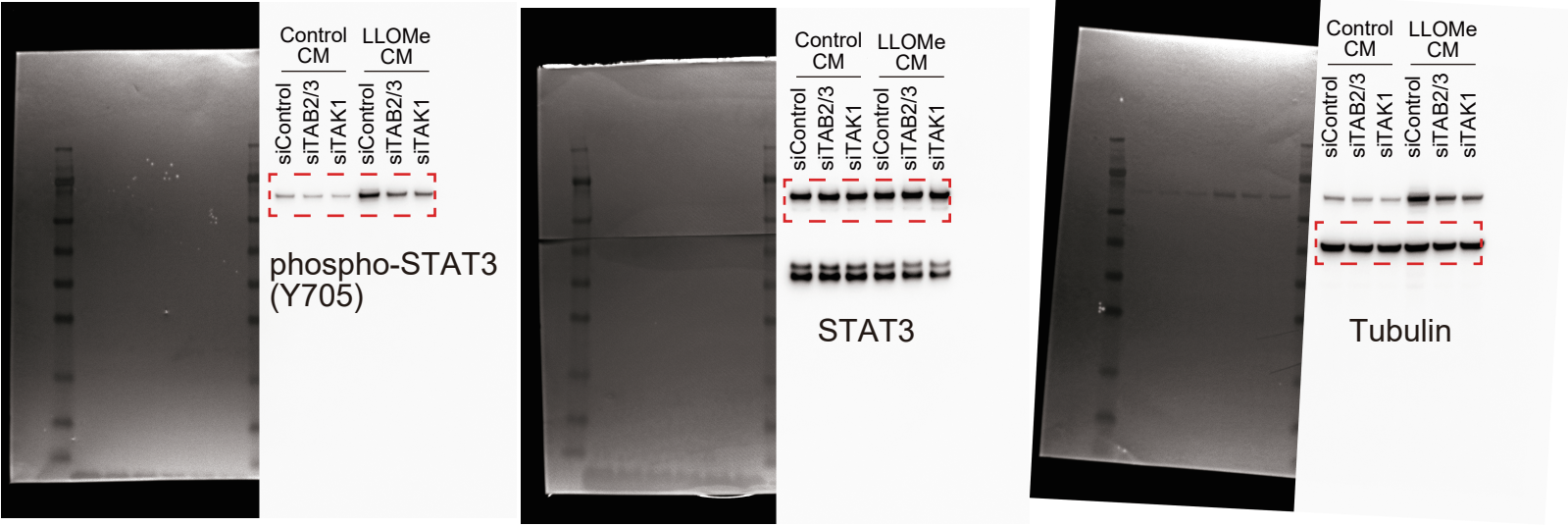

Figure 5J

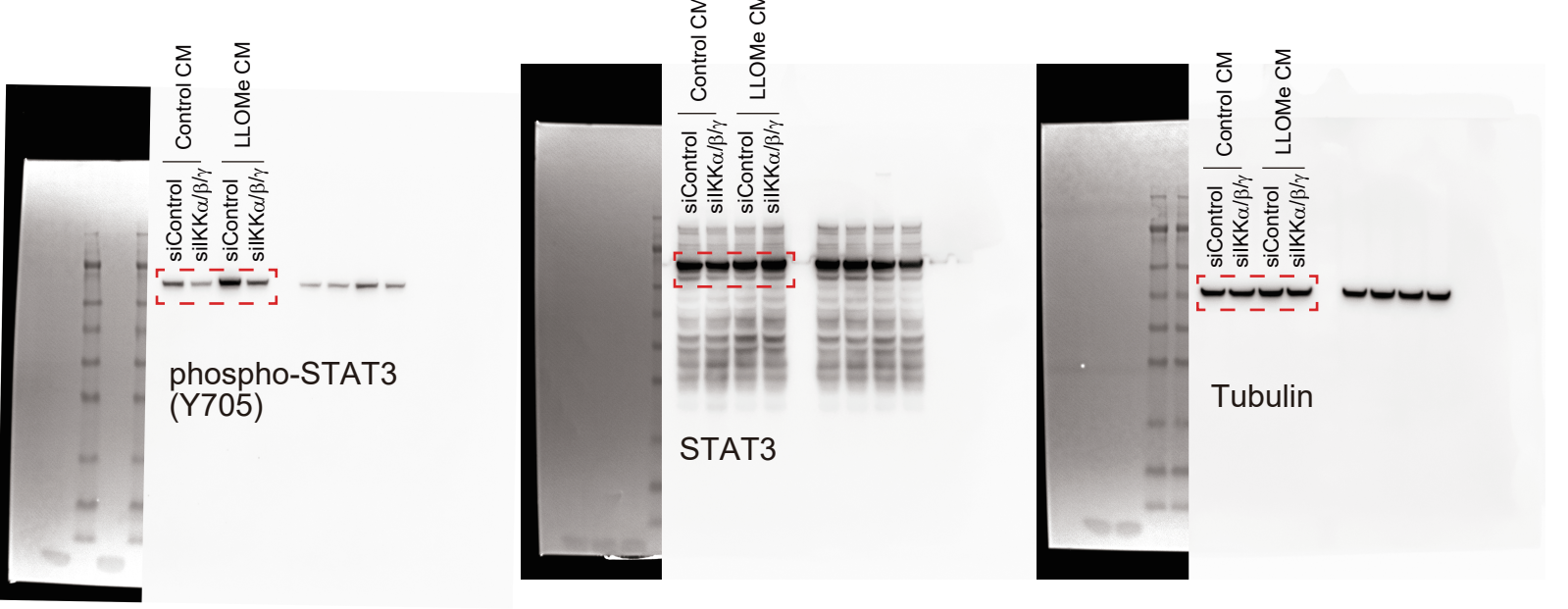

Supplement: Figure 5—source data 4. [file elife-106901-fig5-data4.pdf]

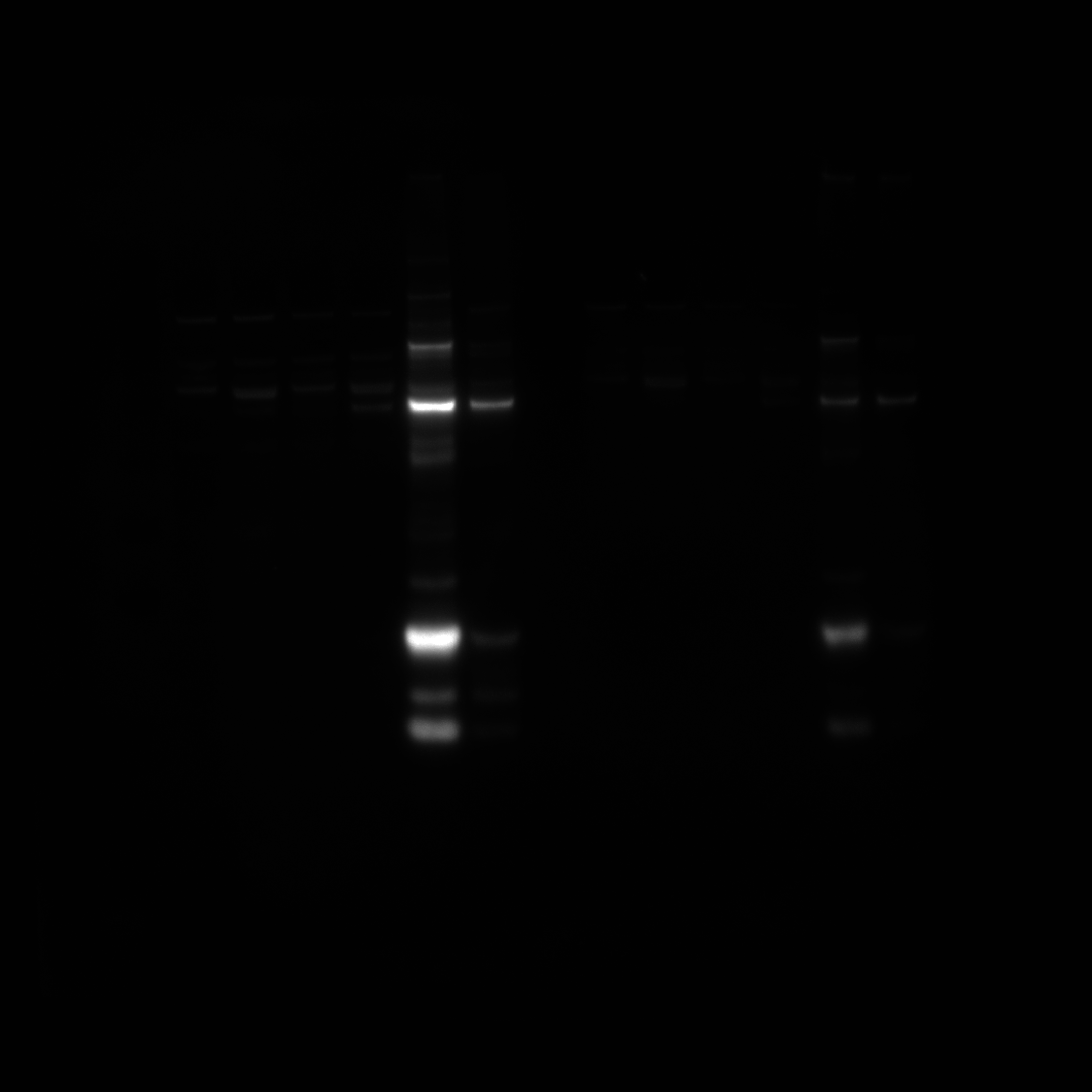

Supplement: Figure 5—figure supplement 1—source data 1. [file elife-106901-fig5-figsupp1-data1.zip › Figure5 figure supplement 1 source data 1/Figure S5A Caspase3.Tif]

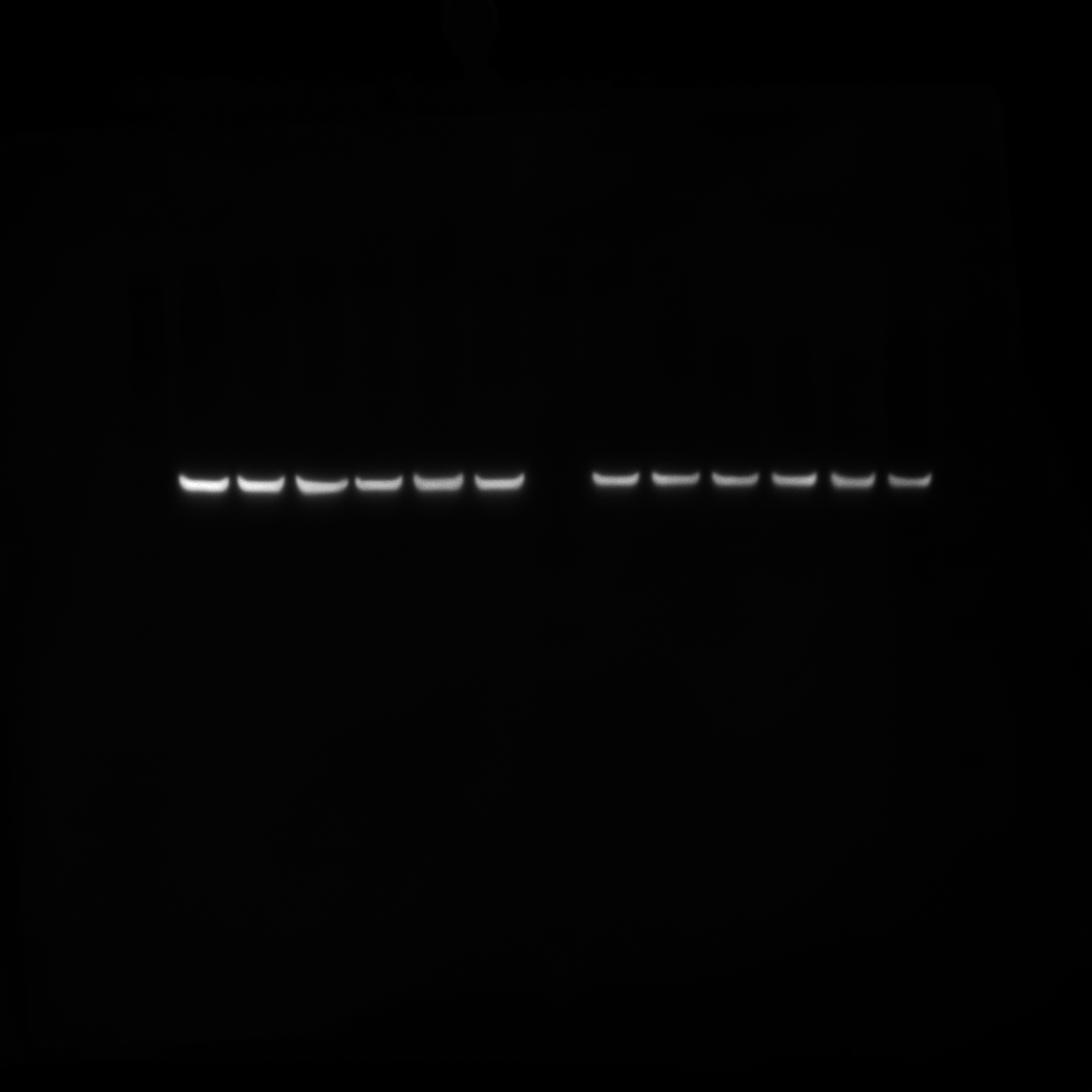

Supplement: Figure 5—figure supplement 1—source data 1. [file elife-106901-fig5-figsupp1-data1.zip › Figure5 figure supplement 1 source data 1/Figure S5A Tubulin.Tif]

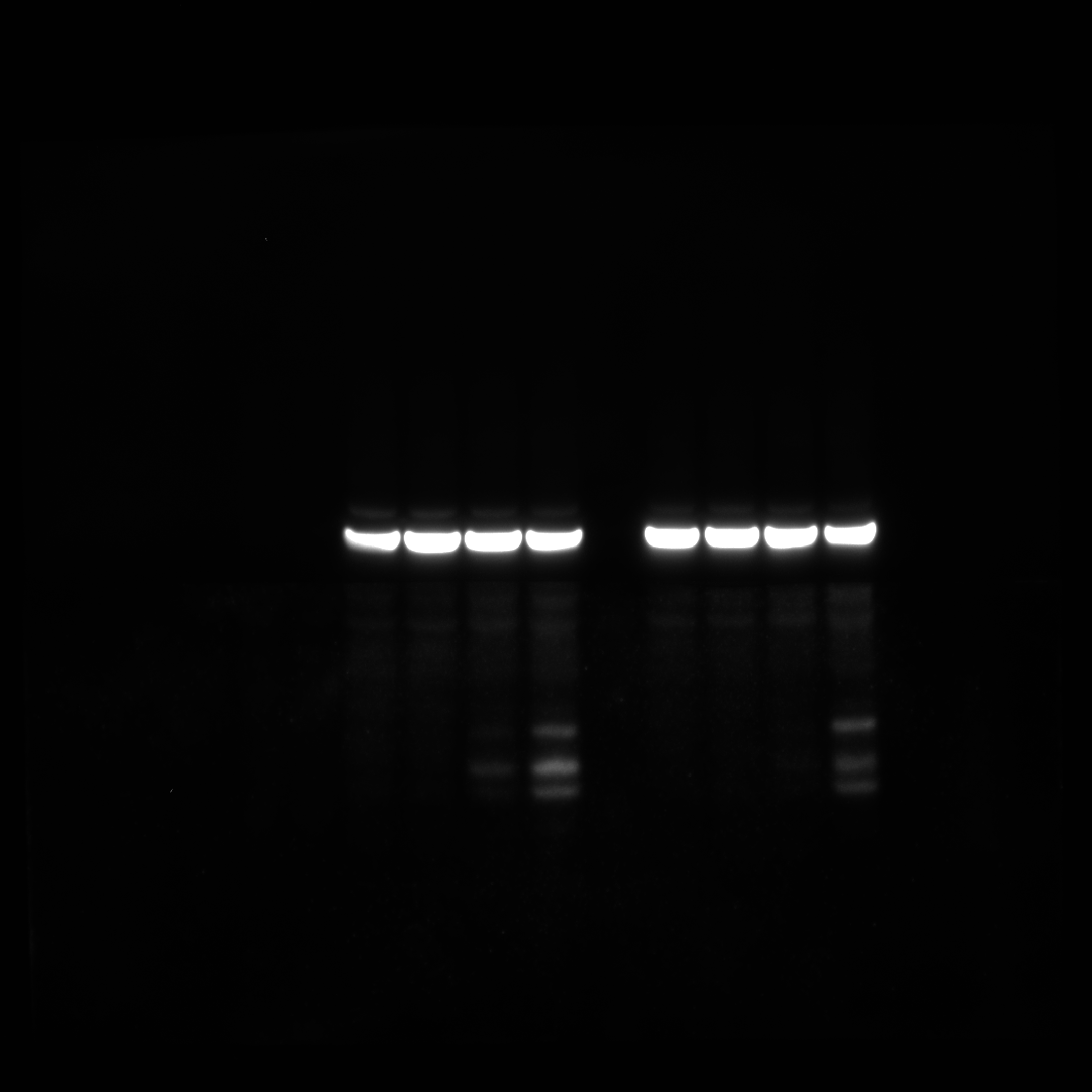

Supplement: Figure 5—figure supplement 1—source data 1. [file elife-106901-fig5-figsupp1-data1.zip › Figure5 figure supplement 1 source data 1/Figure S5B Caspase3.Tif]

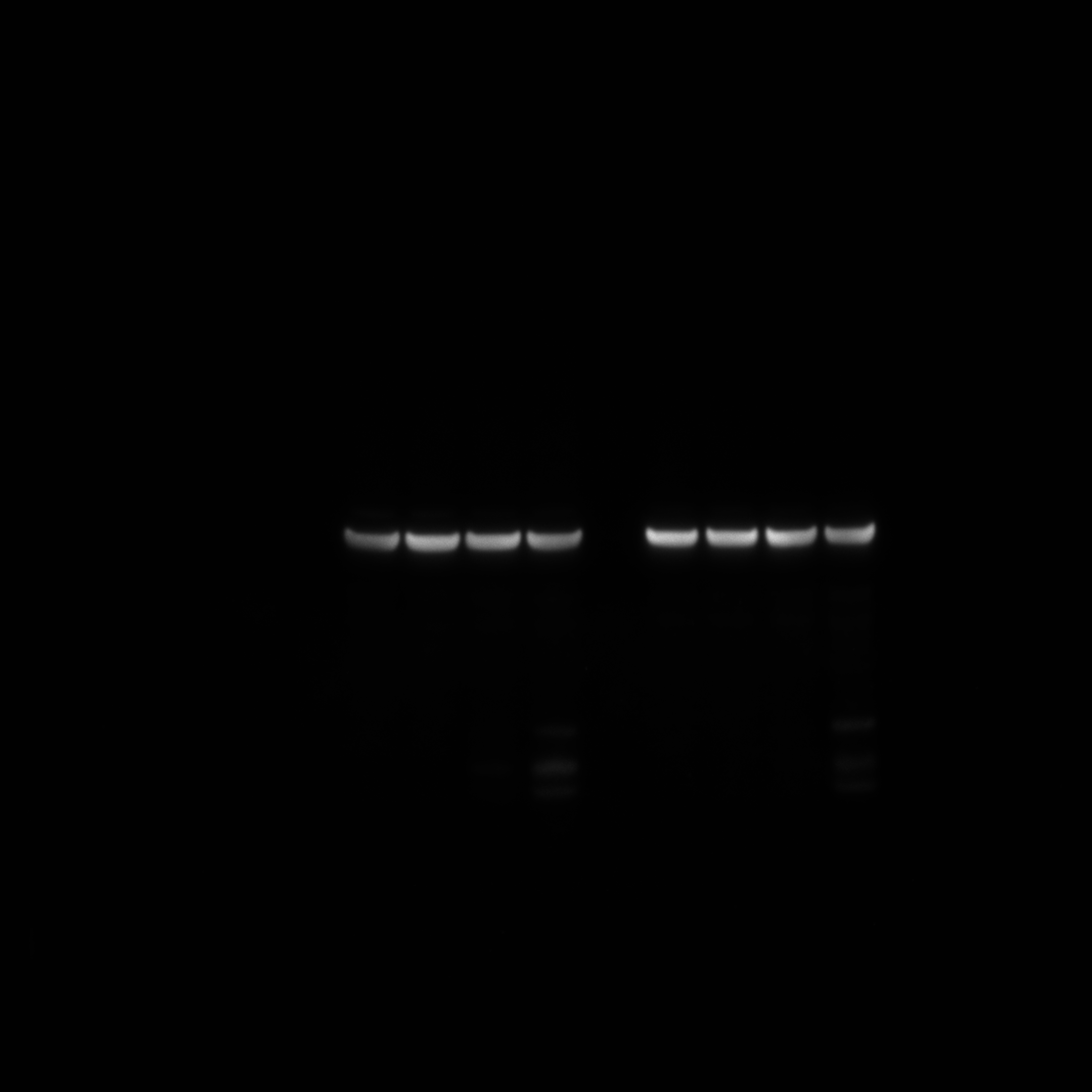

Supplement: Figure 5—figure supplement 1—source data 1. [file elife-106901-fig5-figsupp1-data1.zip › Figure5 figure supplement 1 source data 1/Figure S5B Tubulin.Tif]

Figure S5A

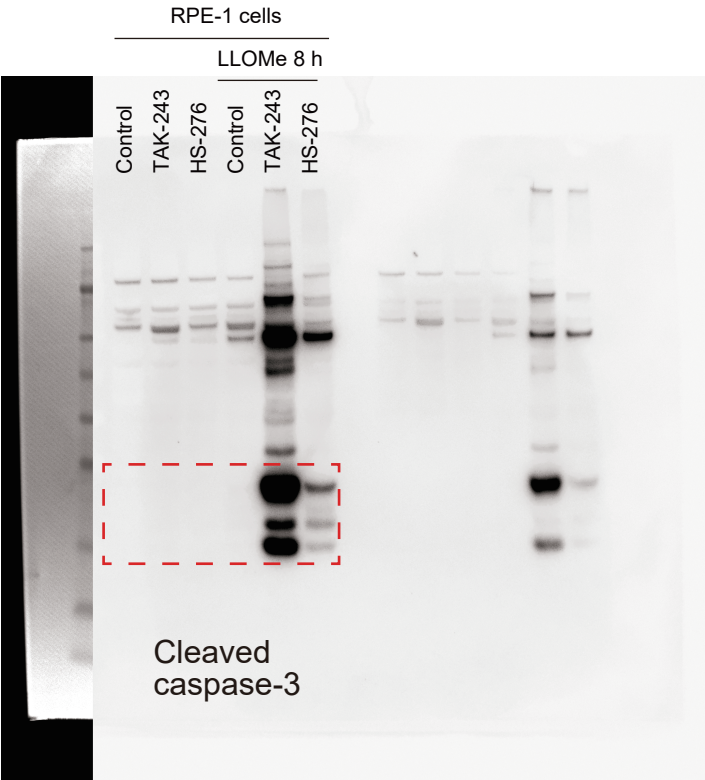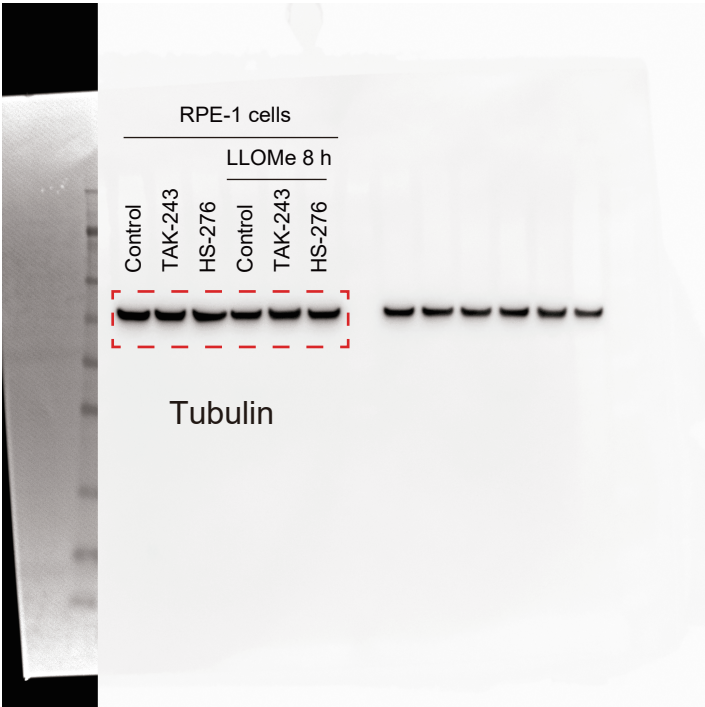

Figure S5A

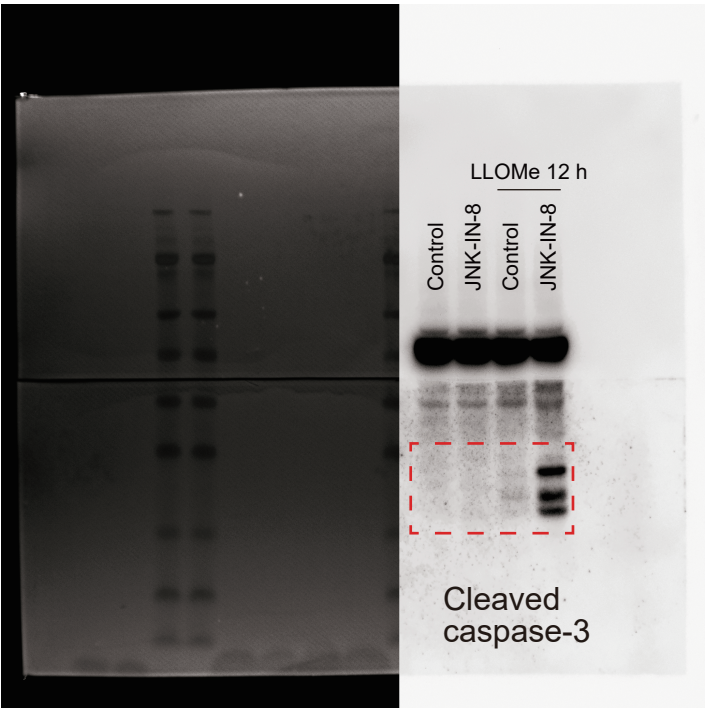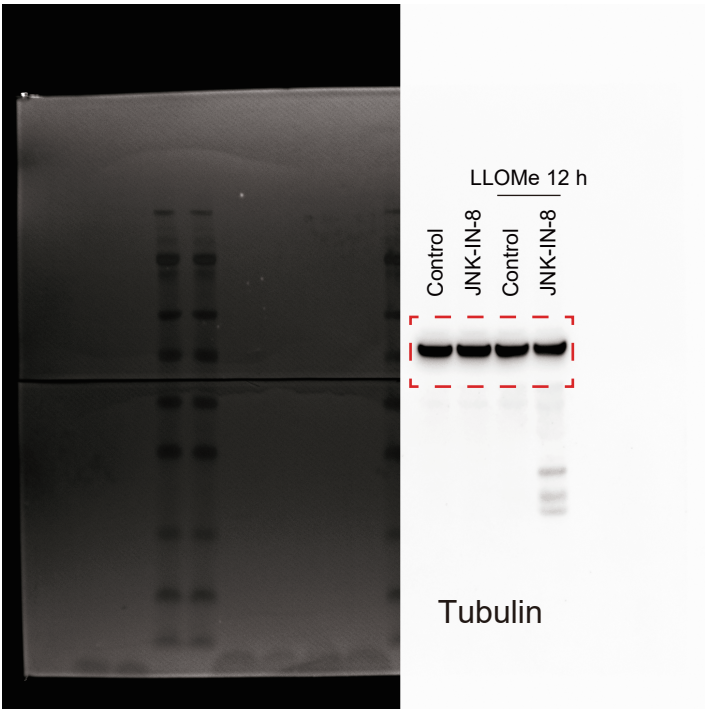

Supplement: Figure 5—figure supplement 1—source data 2. [file elife-106901-fig5-figsupp1-data2.pdf]
